# Revisiting the Modification Strategies of Alloy-Base Anode for Solid-State Lithium-Ion Batteries Through Deconstructing Anode-Interface-Solid Electrolyte

**DOI:** 10.1007/s40820-026-02157-0

**Published:** 2026-04-21

**Authors:** Yueying Chen, Hanyi Yu, Yuerui Lin, Cong Liu, Akif Zeb, Zijian Cai, Hongzhe Chu, Yuhong Luo, Xiaoming Lin, Jiaye Ye

**Affiliations:** 1 School of Chemistry, South China Normal University, Guangzhou, 510006 People’s Republic of China; 2 Institute of Materials Engineering, University of Siegen, No.9-11 Paul-Bonatz-Str., 57076 Siegen, Germany; 3 School of Chemistry and Physics, Faculty of Science, Queensland University of Technology, 2 George Street, Brisbane, QLD 4000 Australia

**Keywords:** Alloy anode, Solid electrolyte, Solid-state batteries, Solid-state lithium-ion batteries

## Abstract

In recent years, advanced battery systems based on solid electrolytes have become a research hotspot to replace traditional liquid lithium-ion batteries due to their significant advantages in energy storage performance and safety. The alloy anode materials (such as Si, Sn, and P) have attracted much attention due to their significantly higher theoretical capacity than graphite. This article systematically reviews the characteristics, key challenge and the latest progress of alloy-solid-state batteries at the anode and solid electrolyte levels. It is emphasized that through strategies such as structural design, material composite, surface engineering and overall electrode system optimization, the volume expansion problem of alloy materials during the cycling process can be alleviated. Meanwhile, in-depth analyses of the dynamic evolution of the interface of solid electrolytes, the kinetics of lithium-ion transport, and the failure mechanisms and innovative strategies in terms of mechanical properties have also been conducted. In addition, this paper introduces the in-depth analysis of the dynamic mechanism in the lithiation process through advanced in situ characterization techniques and multi-physics field simulation methods, thereby providing theoretical guidance for material design. Finally, the potential directions and future opportunities for promoting the development of solid-state batteries with alloy-based anodes are explored.

## Highlights

- The characteristics and key challenges of the anode and solid electrolyte levels in alloy-solid-state batteries are reviewed.
- The focus is on anode modification strategies such as interface modification, structural design, and composite electrolytes.
- Analysis of failure mechanisms and innovation strategies regarding solid-state electrolyte interfaces, lithium-ion transport dynamics, and mechanical properties.
- Looking forward to the potential directions and future opportunities of the development of alloy-based anode solid-state batteries.

## Graphical abstract

**Figure:**
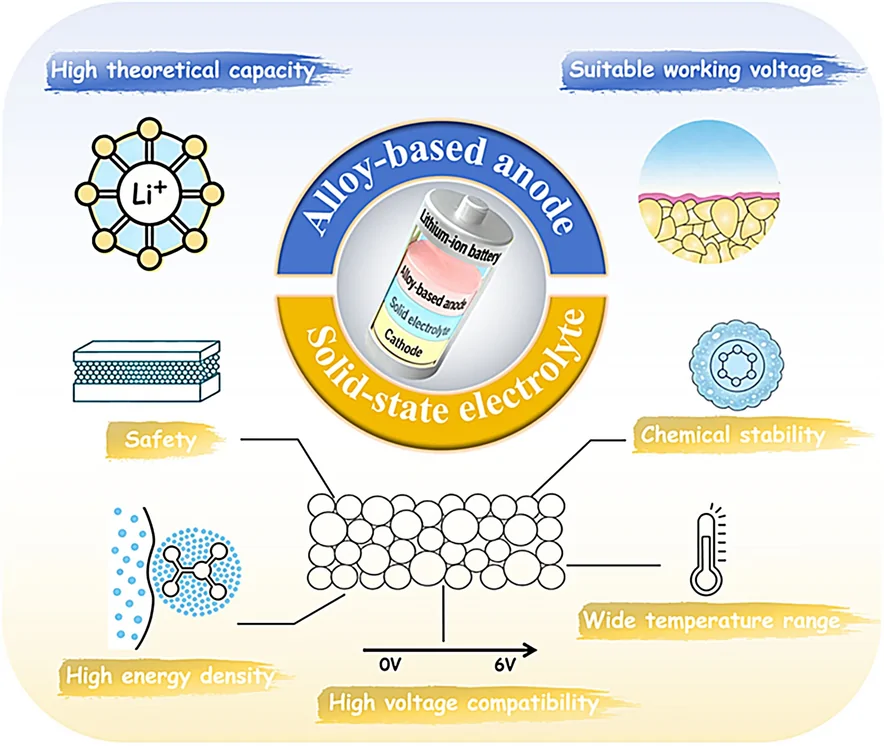

## Introduction

In 1991, lithium-ion batteries were successfully commercialized, being a technological breakthrough that brought about profound changes to the modern human society. Today, their applications have permeated every aspect of daily life. As an important technology in the field of energy storage, lithium-ion batteries have long attracted industry attention due to their significant advantages such as high energy density, excellent cycle stability, low environmental impact, and no memory effect. They were even once regarded as a highly promising energy storage solution because of their characteristic of no memory effect [1–4]. However, the development of the traditional lithium-ion batteries is facing severe challenges now. From the current technological level, the actual energy density of lithium-ion batteries is approximately 300 Wh kg^−1^ [5, 6]. Meanwhile, the global reserves of fossil fuels are constantly decreasing, while the demand for sustainable energy alternatives is increasing day by day. Developing energy conversion and storage technologies that combine cost advantages, environmental friendliness and excellent performance has become a core task in the current energy field. Especially, the rapid development of the electric vehicle industry and the continuous expansion of the grid energy storage field have further promoted the upgrading of rechargeable battery technology toward higher energy density and power density [7–9]. Against this backdrop, accelerating the research and development of lithium-ion batteries with higher energy density, longer cycle life, lower production costs and more complete safety mechanisms has become an urgent need for the industry's development.

However, traditional liquid lithium-ion batteries have inherent risks such as thermal runaway due to heat generation from long-term operation or internal structure damage [10]. Meanwhile, electrolyte leakage and lithium dendrite growth affect the reversible capacity and stability of the battery [11]. In contrast, the core advantage of solid-state lithium-ion batteries lies in the fundamental change of adopting solid-state electrolyte, thus achieving significant breakthroughs in safety, energy density and long-term stability [12–14]. The inherent high mechanical modulus of solid electrolytes can physically inhibit the puncture by lithium dendrites, greatly reducing the risk of internal short circuits and making the application of high-capacity anodes possible, providing a key path to break through the upper limit of theoretical energy density of traditional liquid systems. Their non-flammable and non-volatile characteristics can solve the thermal runaway safety hazard of liquid batteries, and at the same time expand the working temperature range of the battery [15, 16]. In terms of electrochemical stability, solid-state systems, especially those based on sulfides, have a higher lithium-ion migration number, which can reduce concentration polarization during the cycling process [16]. A wider electrochemical window also allows for matching with cathode materials of higher voltage, further enhancing the energy density of the entire battery. In addition, the structural design of solid-state batteries is expected to simplify the packaging system, enhance volumetric energy density and system integration efficiency [17]. Although solid-state batteries still face challenges such as interface impedance, solid-solid contact, and material cost at present, their inherent safety and high-performance potential make them a key development direction for the next-generation energy storage technology.

Lithium-ion batteries typically use graphite-based materials as the anode, and currently their energy density is gradually approaching the theoretical and technical upper limit. In addition, lithium metal anodes are regarded as one of the paths to enhance the energy density of solid-state lithium-ion batteries due to their specific capacity advantage [18, 19]. However, during the cycling process, due to the uneven deposition and exfoliation of lithium and the significant volume changes, the interfacial contact in the case of lithium metal anodes deteriorates significantly. This causes local current density concentration, which may induce the nucleation and growth of lithium dendrites even in solid electrolytes, and ultimately lead to mechanical failure of solid electrolytes [20]. In addition, there is thermodynamic instability between metallic lithium and most solid electrolytes, especially sulfide electrolytes, which can easily lead to continuous side reactions at the interface. Therefore, recently, high-capacity alloy anode materials without lithium richness have become a research hotspot due to their significant theoretical capacity advantages, especially the tetravalent (such as silicon, germanium, tin) and pentavalent (such as phosphorus, antimony, bismuth) alloy anode materials [21–23]. Compared to the commercially mature graphite anode, the alloy anode offers a theoretical specific capacity that is an order of magnitude higher. This characteristic directly and fundamentally enhances the energy density of the battery cell, meeting the urgent demand for range in electric vehicles and high-end electronic devices. Compared to the lithium metal anode with the highest theoretical capacity, the alloy anode avoids the fundamental safety hazard of lithium dendrite growth. The alloy anode accommodates lithium in an alloyed form through a solid-phase reaction, fundamentally eliminating the phase deposition process of metallic lithium and significantly reducing the thermodynamic driving force for dendrite formation. At the same time, its working potential is slightly higher than the lithium deposition potential. This moderate potential window can not only provide a high output voltage and energy density, but also provide a certain safety buffer for the unexpected precipitation of lithium in actual fast charging conditions. Secondly, the chemical compatibility of alloy-type anode materials with various solid electrolytes is superior to that of highly active metallic lithium, and they remain thermodynamically stable with most solid electrolyte materials [24]. In addition, most of these alloy materials are naturally abundant in reserves and environmentally friendly, which is more conducive to reducing the cost of industrial applications. However, such materials still face many challenges in practical applications, among which the most critical ones are the electrode structure damage and the instability of the solid electrolyte interface (SEI) layer caused by the huge volume change during charging and discharging [25]. During the dynamic cycling process, especially for alloys with highly expansive volume, the composition of the SEI constantly undergoes breakdown and reformation, forming complex multi-layer or gradient structures [26]. This structural evolution directly alters the activation energy distribution required for lithium ions to cross the interface, affecting ion transport. Additionally, the brittle SEI will rupture and flake off, causing the rupture site to expose new highly active surfaces, which will continuously consume the electrolyte and active lithium [27]. In terms of electrolytes, the intense periodic deformation of alloy materials mechanically destroys the interfacial intimacy between them and rigid solid electrolytes, leading to interfacial contact failure [28]. The problems of low lithium-ion conductivity, poor electronic conductivity and high interfacial resistance of solid electrolytes cause rapid capacity decline and deterioration of rate performance of batteries [11, 29, 30]. Therefore, the mechanical-electrochemical compatibility problem when solid electrolytes are coupled with alloy-type anodes restricts the actual cycle life and performance of the entire battery.

**Scheme 1:**
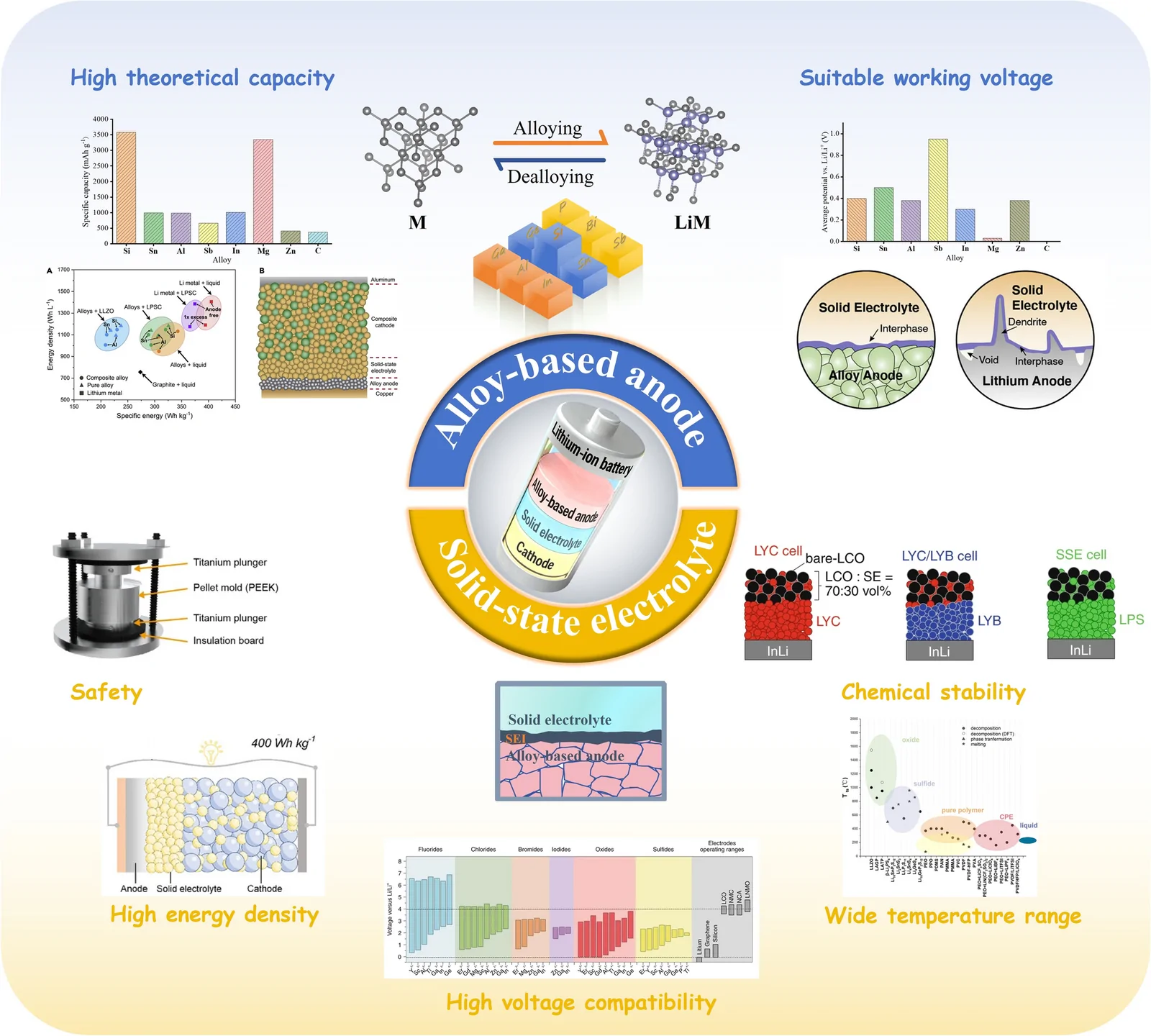
Advantages of alloy anode materials and solid electrolytes in solid-state lithium-ion batteries [31–35]. © 2022 Elsevier Inc. © The Author(s), under exclusive license to Korean Institute of Chemical Engineers, Seoul, Korea 2024. © 2023 Wiley–VCH GmbH. © Springer Nature Limited 2020. © 2021 The Authors. InfoMat published by UESTC and John Wiley & So

Herein, we systematically review the challenges and strategies of high-energy-density alloy-based solid-state batteries in terms of electrodes and solid electrolytes (Scheme 1) [31–35]. On the one hand, we will systematically review the characteristics, basic electrochemical mechanisms, and development status of various alloy-based electrodes, including those containing Si, Sn, Sb and P, etc. On the other hand, the key challenges of alloy-based electrodes in solid-state lithium-ion batteries will be analyzed vis a vis three major problems: ultimate volume expansion, slow electrochemical kinetics, and low area capacity. Subsequently, the optimization strategies and current development status of alloy-based anodes will be analyzed in the field of solid-state lithium-ion batteries in depth, with a focus on discussing the cutting-edge breakthroughs in battery engineering such as structural design, material composite, surface engineering, and new binders and conductive agents. In addition, the characteristics, advantages, and transport mechanisms of different solid electrolytes in solid-state lithium-ion batteries will be discussed. The adverse effects on alloy-based solid-state batteries have been analyzed from the perspectives of low ionic conductivity, inherent electrochemical instability, and mechanical failure caused by stress. Afterward, the review focuses on the methods to stabilize the interface between alloy-based materials and solid electrolytes through strategies such as interface modification, structural design, and composite electrolytes, used in the development of all-solid-state batteries with long cycle life and high energy density. In addition, advanced in situ/operational characterization techniques, further in-depth exploration and resolution of the key mechanisms of electrochemical reaction processes, fundamental structural evolution, and material degradation mechanisms have been discussed. Finally, we put forward forward-looking viewpoints and strategic suggestions, and point out the direction for subsequent scientific research, aiming to promote the development process of high-energy-density solid rechargeable batteries from the basic understanding of mechanisms to material optimization and application expansion using alloy anodes.

## Alloy-Based Anode in Solid-State Lithium-Ion Batteries

### Characteristics of Alloy Anode in Solid-State Lithium-Ion Battery

The core of high capacity in alloy-type anode materials lies in their unique alloying reaction mechanism [36]. Graphite can only bind about 1/6 lithium atoms per carbon atom through intercalation lithium storage. In contrast, metal atoms in alloy materials can chemically react with multiple lithium atoms to form lithium-rich intermetallic compounds. For example, a silicon atom can bind up to about 4.4 lithium atoms to form Li_4.4_Si. The high lithium coordination ratio, coupled with the relatively low atomic mass of these metal elements, jointly contributes to its theoretical specific capacity far exceeding that of graphite [37]. The theoretical capacity of silicon is as high as about 4200 mAh g^−1^, and tin is about 990 mAh g^−1^, far higher than graphite (372 mAh g^−1^) [38]. Therefore, high capacity directly stems from the essential characteristic of alloying reactions that allows a single metal atom to accommodate multiple lithium atoms. In solid-state batteries, silicon-based anode batteries using sulfide solid-state electrolytes can achieve energy densities close to lithium metal anodes, while avoiding the safety hazards posed by lithium dendrites [39]. The performance far exceeds traditional graphite anode liquid batteries, especially in designs with high active material loading and thin electrolyte separators. It is worth noting that when the specific capacity of alloy anodes exceeds about 1,000 mAh g^−1^, the marginal improvement effect on overall energy indicators will gradually weaken, which means that material selection needs to balance capacity and volume expansion rate. It is worth noting that alloy anodes, due to their high capacity characteristics and thinness, have a relatively small impact on overall battery energy even when solid-state electrolytes are added to the anode to enhance ion conduction [32].

In terms of reaction potential, alloy-type anodes demonstrate the characteristics of being both suitable and safe. Their lithiation reactions typically occur in the potential range of 0.1 to 0.6 V (relative to Li^+^/Li). This potential range has dual advantages: Firstly, it is significantly higher than the deposition potential of metallic lithium (0 V). It provides a crucial thermodynamic safety margin, meaning that under normal charge and discharge conditions, lithium ions tend to embed into the material for alloying reactions rather than reducing and depositing as metallic lithium on the material surface. Secondly, this potential is also lower than the significant reduction decomposition potential of most organic electrolytes, which usually lies between 0.8–1.5 V. Although a SEI is still formed in the first cycle, in subsequent cycles, as long as the potential is properly controlled, the risk of continuous electrolyte decomposition is relatively low [40, 41]. Furthermore, this platform which is above 0 V but below the decomposition potential of the electrolyte enables the entire battery to maintain a sufficiently high working voltage when paired with high-voltage cathodes (such as lithium cobalt oxide or ternary materials), ensuring energy density and avoiding the significant voltage sacrifice issue that occurs with high-potential anodes (such as lithium titanate).

The use of lithium metal anodes in solid-state batteries faces two core challenges, including internal short circuits caused by dendrites and contact failure at the electrode interface [42–44]. During the charging and discharging process, lithium metal is prone to form dendrites, which may penetrate solid electrolytes and cause internal short circuits. The problem is particularly prone to occur at the grain boundaries or pore locations of electrolytes, as lithium ions preferentially diffuse and deposit along these structural defects. On the other hand, when the lithiation process occurs, the shrinkage of lithium metal causes physical separation at the interface between it and the solid electrolyte, resulting in contact failure. The reduction in contact area leads to a sharp increase in local current density, which in turn further aggravates the growth risk of lithium dendrites [45]. Even if external pressure is applied in an attempt to maintain contact, in actual operation, it is still difficult to stabilize the control interface due to the low mechanical strength of lithium metal itself (only about 1 MPa). In contrast, alloy anode materials can avoid the above problems by serving as the "reservoir host" of lithium. For instance, alloys containing elements like silicon or tin can reversibly store lithium ions within the alloy lattice during charging and discharging, forming alloy phases and avoiding the direct deposition process of metallic lithium [46]. The approach not only provides stable electrochemical interface contact, but also fundamentally eliminates the growth mechanism of lithium dendrites. Although current research has attempted to add alloy components such as magnesium and silver to lithium metal anodes to improve interfacial diffusion, for instance, by taking advantage of the high lithium diffusion coefficient of magnesium to alleviate the problem of interfacial peeling. However, this essentially still relies on metallic lithium, and its degradation mechanism still exists. The use of pure alloy materials for the anode can solve the interface stability problem from a mechanistic perspective [32]. Although the huge volume expansion is the main obstacle to their commercial application, their high capacity and high safety potential make them important candidates for the next generation of high-energy-density lithium battery anode materials.

**Fig. 1:**
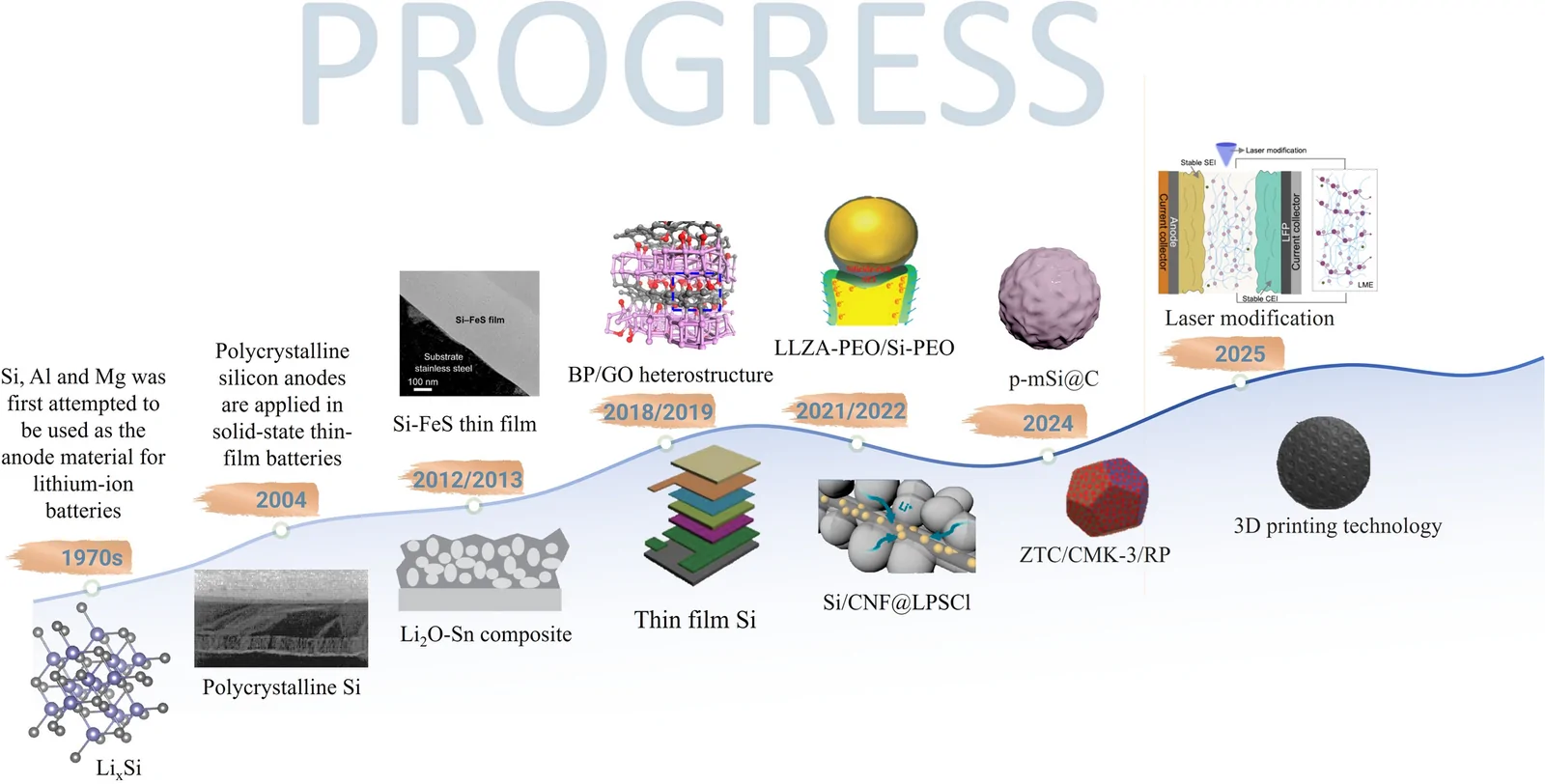
Development and progress of alloy anode materials in solid-state lithium-ion batteries [48–58]. © 2004 World Scientific Publishing Co Pte Ltd Powered by Atypon® Literatum. © 2012 The Japan Society of Applied Physics. © The Royal Society of Chemistry 2013. © 2018 WILEY–VCH Verlag GmbH & Co. KGaA, Weinheim. © 2019 American Chemical Society. © 2021 Elsevier B.V. All rights reserved. © 2021 The Authors. Advanced Science published by Wiley–VCH GmbH. © 2024 American Chemical Society. © 2024 Wiley–VCH. GmbH. © 2025 Science Press and Dalian Institute of Chemical Physics, Chinese Academy of Sciences. Published by Elsevier B.V. and Science Press. All rights are reserved. © 2025 Published by Elsevier B.V

The utilization of redox chemistry based on alloying reactions has brought significant improvements in energy density to solid-state lithium-ion batteries through the use of alloy-type anode materials made of group IVA and VA elements [47]. Silicon (Si) and phosphorus (P) are non-metals, while the rest are metals (such as antimony, germanium, and tin). Their ductility, tensile strength, and abundance of resources have shown great application prospects in the field of energy storage. The specific characteristics and progress of these anode materials are summarized below as well as in Fig. 1 [48–58]. In the early 2000s and before, early research focused on micron-sized phase materials of alloy elements such as tin and silicon through basic electrochemical tests and simple microscopic observations. In the 2010s, the research began to adopt sophisticated designs such as yolk-shell structures, porous frameworks, and hollow carbon spheres encapsulation as models. These structures created reserved expansion space at the nanoscale or sub-micron scale while ensuring continuous transmission paths for electrons and ions. Since the 2020s, new technologies and materials have been utilized to redesign the morphology and surface chemistry of alloy anodes to fit solid lithium-ion batteries, and to study the long-term electrochemical-mechanical behavior under external stacking pressure.

#### Si

Si has demonstrated great application potential in the field of anode materials for lithium-ion batteries (LIBs) due to a series of outstanding properties and has become one of the highly sought-after options. Silicon stands out particularly in terms of theoretical specific capacity. When Li_4.4_Si is formed, its theoretical specific capacity can be as high as 4200 mAh g^−1^, which is the highest theoretical specific capacity among the Li–Si alloys discovered so far. Even in a mild temperature environment, the specific capacity of Li_3.75_Si alloy can reach 3579 mAh g^−1^ [59]. Such a high specific capacity means that lithium-ion batteries using silicon as the anode material can store more energy under the same mass. The combination of stable solid-state interface chemistry makes silicon the key material to break through the energy density bottleneck of 500 Wh kg^−1^. Not only that, the working potential of silicon is relatively low, falling within the range of 0.2 to 0.3 V (relative to Li/Li^+^) [60]. The content of silicon on Earth is extremely considerable, and it is widely present in rocks, sand, and other substances. It provides a solid guarantee for the long-term stable supply of silicon as the anode material of lithium-ion batteries and avoids the problem of restricting its large-scale application due to resource shortage. In addition, low cost and non-toxicity are also its advantages [61]. According to Song et al.’s report, research on silicon-based anodes began in the 1970s, where elemental silicon was first attempted to be used as the anode material for lithium-ion batteries [62]. In 1971, Dey et al. discovered that various metal elements such as silicon, aluminum, and magnesium, could react with lithium ions to form alloys, which laid the foundation for silicon to be used as the anode material of batteries [63]. Subsequently, the research on silicon-based anodes entered a slow and initial exploration period. After the year 2000 CE, low-dimensional silicon nanomaterials have become a research hotspot. Zero-dimensional nanomaterials, alloys, films, and composite matrices have been applied to anodes to alleviate volume expansion problems and enhance cycling stability. With the development of solid-state electrolytes, researchers have begun to explore the compatibility of silicon anodes with solid-state systems. In 2006, Nava Ariel et al. carried out chemical mechanical polishing (CMP) treatment on polycrystalline silicon anodes, reducing their surface roughness from 8.06 to 0.53 nm, and applied it to the anodes of solid-state thin-film batteries [48]. CMP-treated batteries significantly enhanced the uniformity of the SiO_2_ electrolyte layer, achieve nanoscale interface flatness, and increase the charge recovery rate to 40% (non-CMP with only 14.5%). In 2013, Cervera et al. confirmed that solid electrolytes are a key strategy to unlock the high-performance potential of silicon anodes. They achieve rate capability and cycle durability by suppressing interfacial side reactions and material degradation mechanisms [50]. The unique advantage of the solid-state system lies in the fact that they only allow lithium ion migration, eliminating the decomposable components in the electrolyte and thus avoiding the formation of the SEI layer, which directly reveals the inherent potential of silicon anodes. Experimentally, Si-FeS thin film electrodes were fabricated by pulsed laser deposition (PLD) technology. The results showed that in high-performance solid-state batteries, silicon-based anodes can provide a capacity close to the theoretical value at a low discharge rate of 0.1 C. More significantly, the performance was also greatly enhanced at high rates of 10 and 100 C, where 3200 and 2300 mAh g^−1^ of discharge capacities were achieved respectively, far exceeding the previously reported nano-silicon materials. This improvement is attributed to the low interface impedance without the SEI layer in the solid-state environment, rather than the microscopic properties of the film. In addition, the addition of FeS optimized the electronic conductivity and synergistically enhanced the rapid kinetics of the reaction. In recent years, with the surge in demand for high-energy-density batteries in fields such as new energy vehicles, research on silicon anodes in liquid/solid lithium-ion batteries has entered an explosive period.

#### Sn

Tin also forms a rich Li intermetallic compound, Li_2_Sn_5_, through alloying reactions, theoretically achieving a high specific capacity of 990 mAh g^−1^ with a potential of ≈0.55 V. Metallic tin itself possesses high electronic conductivity (approximately 9.17 × 10^6^ S m^−1^), which facilitates charge transfer and alleviates the kinetic bottleneck commonly seen in alloy-type high-capacity materials. Tin is abundant in the earth's crust, is non-toxic, and less expensive than lithium and silicon, making it suitable for large-scale applications [64, 65]. Discussions surrounding tin alloy-based lithium-ion battery anodes date back to 1910, when alloy compounds such as Li_4_Sn, Li_3_Sn_2_, and Li_2_Sn_5_ were first reported [66]. Before 2000, it was in its infancy, with research focusing on the fundamental exploration of tin alloy-based anodes. Performance was improved by mixing active phase tin with inactive phases, such as Sn/SnSb composite anodes, which utilize a multiphase reaction mechanism to allow unreacted tin to act as a matrix to buffer volume expansion, resulting in superior cycling stability compared to pure tin anodes [67]. However, due to technological limitations, the overall electrochemical performance was poor, and both cycling stability and initial coulombic efficiency (ICE) needed improvement. From 2000 to 2006, it entered a preliminary development stage, with an increase in the types of tin alloys, such as various binary alloys (Sn–Ca, Sn–Cu, Sn–Ni, Sn–Ag) and ternary alloys. Performance improved compared to the previous stage, for example, in Sn–Cu alloys, copper acts as an inactive phase, mitigating the impact of volume expansion through excellent electrical properties and a low oxidation potential [68]. The 3D porous Cu_6_Sn_5_ alloy anodes can accelerate lithium-ion transport and electrochemical reactions, performing well at high current densities. From 2007 to 2011, it was a period of rapid development for tin, driven by nanotechnology. Nanoscale tin alloy anodes became a research focus, with nanoparticles, nanowires, and 3D porous structures prepared through reverse microemulsions, template methods, etc. At the same time, the application of carbon materials increased, and composite structures such as carbon coating and embedding in carbon matrices (e.g., Cu_6_Sn_5_@C, SnSb/C) further improved cycling performance and conductivity [69]. Since 2012, it has entered a stable development stage, with the construction of nano and microstructures and their combination with carbon materials remaining the mainstream strategy. More refined and complex structures have been synthesized, such as 3D nanowire networks, hollow structures, monodisperse nanoparticles. For example, the core–shell structure Sn-Co alloy electrode prepared using the Kirkendall effect has excellent cycling performance [70]. In addition, the exploration of phase transformation processes through in situ characterization techniques has deepened the study of reaction mechanisms, providing theoretical support for performance optimization. In 2012, Jee developed a stable Li_2_O-Sn composite anode material for all-solid-state batteries to improve cycling performance. Traditional lithium-based anodes have poor structural stability in thin-film microbatteries, while tin (Sn)-based anodes are highly processable. By depositing Sn and Li_2_O target materials at room temperature through co-sputtering, a structure was formed in which nano-Sn crystals were embedded in an amorphous Li_2_O matrix. Structural characterization showed that the SLC film had unique amorphous characteristics, with nano-sized Sn crystals (10–50 nm) uniformly dispersed in the Li_2_O matrix. This structure effectively avoided the common macroscopic crack defects of pure Sn films. Electrochemical testing results showed that the initial discharge capacity of the SLC material in a half-cell reached 420 mAh g^−1^, and the capacity retention rate at the second cycle was 51%, significantly better than that of pure Sn films (35%). In full-cell (SLC/LiPON/Li) testing, SLC exhibited superior cycling stability, with an initial capacity retention rate of up to 90%, compared to pure Sn (only 30%). The performance improvement is mainly attributed to the dual role of the Li_2_O matrix: on the one hand, it acts as a mechanical buffer layer to absorb volume strain during charging and discharging processes, and on the other hand, it maintains the integrity of the electrode structure by inhibiting crack propagation. Moreover, the introduction of the solid-state electrolyte LiPON (lithium phosphide oxynitride) further blocks the formation path of cracks on the SLC surface [49].

#### Sb

Antimony (Sb), as an anode material for LIBs, has also become an important alternative to traditional carbon-based materials due to its high capacity, suitable potential, good electrical conductivity and flexible material designability [71]. Antimony stores lithium through the alloying mechanism. During the reaction process, a stable Li_3_Sb phase is formed, with a capacity of up to 660 mAh g^−1^. What is more notable is that antimony-based materials offer flexibility in component design. By forming alloys or intermetallic compounds with different metals, the problems of capacity, cycling stability and volume expansion can be synergistically optimized. Antimony can form alloys with active metals such as Sn and Bi, maintaining high capacity while mutually supporting each other through the different potentials of the alloying steps of different active metal phases, significantly enhancing structural stability [72]. In addition, it forms intermetallic compounds with inert metals such as Cu and Ni. Inert metals do not react with Li^+^, but they can act as mechanical buffer phases and conductive skeletons, inhibiting the agglomeration of Sb particles, alleviating volume expansion, and enhancing the electronic conduction efficiency [73]. As early as the late 1990s, research began to focus on the electrochemical reaction between lithium and antimony alloys. Alloys such as Co-Sb, In-Sb, and Ti-Sb were all included in the research scope. These fundamental explorations laid the foundation for the subsequent application of antimony-based alloy anodes in lithium-ion batteries. In 2004, the Striebel and team fabricated Cu_2_Sb thin-film electrodes at room temperature using pulsed laser deposition technology and tested their electrochemical performance in lithium-ion batteries [74]. It was found that when cycled within a limited voltage window of 0.65–1.4 V (vs Li/Li^+^), the alloy anode exhibited relatively stable cycling performance within 50 cycles. However, before 2006, antimony alloy-based anodes mostly relied on the "active/inert" principle to alleviate the volume effect of antimony, and thus their overall performance was rather mediocre. In 2011, Yan's team synthesized Zn-Sb alloys with different nanostructures in one step through electrochemical deposition without the need for templates [75]. Among them, the nanosheets presented a porous morphology with pore diameters ranging from 100 to 800 nm. The open porous structure not only facilitated the diffusion of lithium ions, but also buffered the volume expansion of antimony during the lithiation process. As a result, the Zn-Sb nanosheet electrode exhibited a higher specific capacity and better cycling stability compared to the nanowire and nanoparticle electrodes. In 2018, the Prieto team investigated the relationship between the electrochemical performance of Cu-Sb alloy thin-film anodes and the composition of the films as well as the film-substrate interface [76]. To extend the cycle life, they constructed a variety of Cu_x_Sb (0 < x < 2) thin-film anodes and different film-substrate interfaces (with or without a Ni barrier layer) and tested their performance as anodes for lithium-ion batteries. The research found that when x = 1, the cycling stability of the Cu_x_Sb@Cu thin film anode was found to be the best. The non-stoichiometric composition was conducive to the formation of the Li-Cu-Sb ternary phase, thereby improving the cycling stability and reducing the excessive growth of the SEI caused by excessive Cu. In addition, the Cu_x_Sb@Ni@Cu thin film anode with a Ni barrier layer had better cycling performance than the Cu_x_Sb@Cu electrode, because the Ni barrier layer could prevent the mutual diffusion between the film and the substrate as well as the formation of voids, avoiding film delamination and thereby extending the cycling life. In 2019, Kumari and team investigated the efficient and stable performance of antimony (Sb) as the anode material in all-solid-state lithium-ion batteries using lithium borohydride (LiBH_4_) as the solid electrolyte [77]. LiBH_4_ has a high lithium-ion conductivity, as much as 10^−3^ S cm^−1^ in the 120 °C high-temperature phase. Although LiBH_4_ was previously mainly used in solid electrolytes of hydride materials, this study for the first time extended it to Sb anode materials. Composite anode materials containing acetylene black (AB) and LiBH_4_ were prepared by high-energy ball milling, and their high stability in multiple cycles and coulombic efficiency as high as 90%-99% were demonstrated. Li_2_Sb and Li_3_Sb were formed at the Sb anode at 0.86 and 0.82 V respectively. During the charging process, these reactions exhibited a high degree of reversibility, with a polarization voltage below 0.1 V, which was much lower than that of the liquid electrolyte system. The initial volume capacity of the Sb anode was 4393.4 mAh cm^−3^, and the capacity attenuation was only 5% after cycling. The synergistic effect of LiBH_4_ and AB not only alleviated the stress caused by volume expansion, but also enhanced ionic and electrical conductivity, thereby significantly improving cycling stability.

#### P

Similarly, phosphorus (P) has been widely studied as an anode material for lithium-ion batteries in recent years as well, with its core attraction being its extremely high theoretical specific capacity. Phosphorus is a somewhat special element, as it has three allotropes: red phosphorus, black phosphorus, and white phosphorus, each with significantly different electrochemical properties [78]. However, white phosphorus is highly toxic and unstable at room temperature, making it unsuitable for use in battery applications. Red phosphorus, due to its abundant resources, low cost, and minimal pollution, is used in battery applications. Red phosphorus (RP) is currently one of the research hotspots as an anode material for all-solid-state lithium batteries. After lithium ions are inserted into amorphous red phosphorus, an amorphous Li-P alloy is first formed, which eventually transforms into crystalline Li_3_P. However, in liquid electrolyte systems, the red phosphorus anode gradually dissolves during multiple cycles, resulting in loss of active material and capacity degradation [79, 80]. Solid-state nuclear magnetic resonance (NMR) studies have shown that there is incomplete symmetry (such as intermediate products LiP_7_, Li_3_P_7_, etc.) and the formation of irreversible phases (such as Li_3_PO_4_) during charging and discharging, leading to capacity loss. Solid-state electrolytes are solid barriers that can effectively confine the intermediate products of polyphosphide to the anode region, preventing their shuttling and greatly improving cycle stability [81]. Black phosphorus (BP), whereas, has a theoretical specific capacity of up to 2596 mAh g^−1^ (based on the formation of Li_3_P), which is much higher than that of commercial graphite (372 mAh g^−1^). Its thermal stability can be achieved at up to 450 °C in a nitrogen atmosphere, demonstrating the potential for high-energy-density batteries [82]. Due to its unique folded layered structure, black phosphorus provides two-dimensional interstitial space for the insertion and extraction of lithium ions, thereby exhibiting excellent lithium storage potential. Theoretical calculations show that the interaction between single-layered or few-layered black phosphorus and lithium ions is stronger than that between the graphene and lithium ions, which helps increase the open circuit voltage and enhance electrochemical performance. In addition, lithium ions have an ultrafast diffusion rate in the zigzag direction of black phosphorus, with a diffusion energy barrier of only 0.08 eV, much lower than that of graphene and molybdenum disulfide (MoS_2_), showing excellent rate performance. Studies have also found that even at a high lithium/phosphorus ratio, the structural distortion of black phosphorus during lithiation can self-recover, exhibiting good reversibility [81]. The development of phosphorus as an anode material for lithium-ion batteries has gone through multiple key stages, from early theoretical exploration to technological breakthroughs. In the early 2010s, researchers improved conductivity and buffered volume changes through nanocrystallization and carbon composites. For example, black phosphorus/graphite composites achieved structural stability through phosphorus-carbon covalent bonds, improving lithium-ion conduction efficiency. In laboratory tests, fast charging for 9 min restored 80% of the battery capacity, and the capacity remained at 90% after 2000 cycles [83]. After 2015, black phosphorus has become a research hotspot due to its layered structure and higher electronic conductivity. However, its phase transition process (P → LiP_7_ → Li_3_P_7_ → LiP → Li_3_P) leads to prominent issues of solvent co-intercalation and polyphosphide dissolution. Interface engineering has become a key breakthrough direction, such as inhibiting side-reactions through polymer gel coating or constructing organic/inorganic heterointerfaces, while accelerating electron/ion transfer [75].

#### Others

Additionally, other alloy-based anode materials can also form stable alloy compounds with a high proportion of lithium, providing a higher charging capacity than aluminum (990 mAh g^−1^) [84], bismuth (380 mAh g^−1^) [85], and germanium (1624 mAh g^−1^) [86], and have great potential as anode materials. As anode materials for lithium-ion batteries, their appeal lies in the fact that they offer a relatively good compromise point among capacity, volume expansion, conductivity, and safety. They are not like silicon, which pursues extreme capacity but faces huge expansion challenges, nor are they like graphite, which is safe but has limited capacity. In the research of lithium-ion batteries, bismuth (Bi) has shown significant advantages as an anode material due to its high volumetric capacity (3765 mAh cm^−3^) and low volumetric expansion rate (42%). Kumari also prepared the Bi-LiBH_4_-acetylene black (AB) composite anode material by high-energy ball milling. In this material, LiBH_4_ serves as both a solid electrolyte and participates in electrode reactions, while AB enhances conductivity and alleviates volume changes [77]. Electrochemical tests show that the composite material operates at a voltage window of 0.2–1.5 V (vs. Li^+^/Li) and a working temperature of 120 °C. The initial discharge/charge capacities were 4681.7 mAh cm^−3^ (478.7 mAh g^−1^) and 4510.5 mAh cm^−3^ (461.2 mAh g^−1^) respectively, and the first effect reached 96.3%. The charge–discharge curves showed two platforms: 0.82 V (Bi → LiBi) and 0.78 V (LiBi → Li_3_Bi), corresponding to reversible alloying reactions, with polarization voltages < 0.1 V, which is much lower than that of the liquid electrolyte system. After 100 cycles, the capacity remained at 3853.3 mAh cm^−3^ (394 mAh g^−1^), and the stability was significantly better than that of Bi nanoparticles in traditional liquid electrolytes (only 60 mAh g^−1^ after 100 cycles). The performance improvement was attributed to the ion conduction promotion of LiBH_4_ and the buffering effect of AB that worked together to inhibit the fracture of the electrode structure. Meanwhile, the high-temperature phase of LiBH_4_ (120 °C) optimized the reaction kinetics. Furthermore, although the theoretical capacity of Bi (380 mAh g^−1^) is lower than that of Sb (660 mAh g^−1^), its smaller volume expansion makes its cycling stability better, which is suitable for the high safety requirements of all-solid-state batteries. This research provided new ideas for the development of high-capacity and high-stability anode materials, especially by directly using bulk materials, which avoids the complexity of preparing nanomaterials. The current research hotspots and future development mainly focus on how to suppress the volume expansion effect to the greatest extent through means such as material nanoengineering, composite design and interface control, improve cycle stability and initial efficiency, optimize rate performance at the same time, and solve the cost problem of large-scale production. If significant breakthroughs can be made in cycle life and cost control, these alloy-based anodes are expected to become a supplementary or alternative option to graphite anodes, especially when there are high requirements for volumetric energy density [87, 88].

### Challenges of Alloy Anode in Solid-State Lithium-Ion Battery

**Fig. 2:**
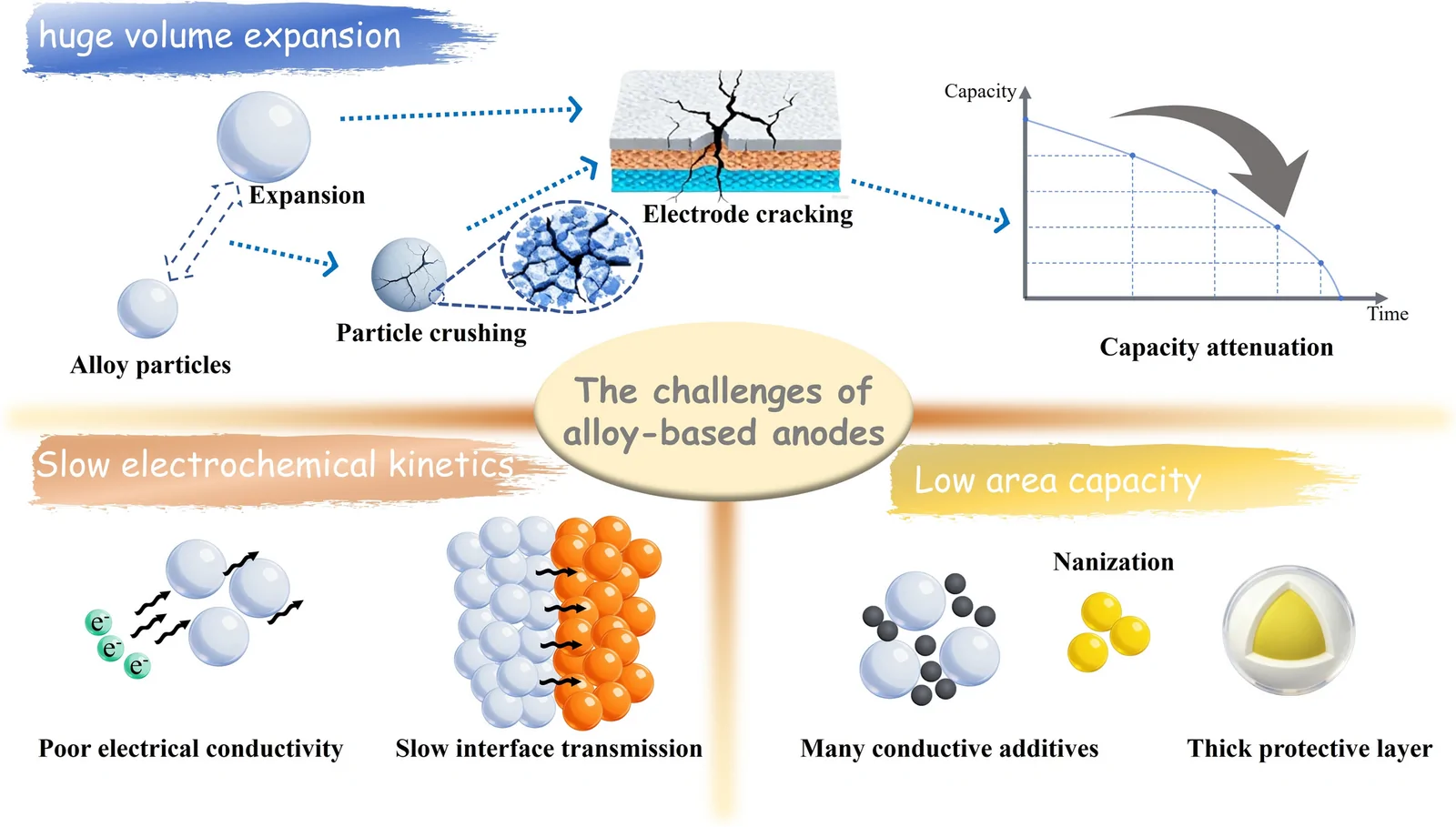
Schematic diagram of the challenges of alloy anodes in solid-state lithium-ion batteries

Although alloy anode materials represented by silicon and tin are regarded as key elements in building the next generation of high-energy-density solid-state batteries due to their theoretical specific capacity far exceeding that of graphite, their integration with solid-state electrolyte systems still faces a series of profound scientific bottlenecks (Fig. 2). There is a huge gap between the ideal high capacity and long-term stability and security in reality. These challenges are not isolated but stem from the fundamental contradiction between the intrinsic physicochemical properties of alloy materials during electrochemical cycling and the strict solid-solid interface requirements in solid-state batteries.

#### Huge Volume Expansion

Alloy-based anodes undergo significant expansion and contraction during charging and discharging, which can generate internal strain and stress at the interface between the electrode and solid electrolyte [89]. The repeated insertion and extraction of lithium ions can generate substantial mechanical stress within the alloy-type material, leading to the crushing of alloy particles and cracking of the electrode. The significant volume change can also cause severe expansion of the electrode thickness, ultimately resulting in detachment of the electrode from the current collector and a rapid decrease in battery capacity [90]. Unlike liquid electrolytes, solid electrolytes, due to their strong mechanical rigidity, cannot transmit stress quickly and uniformly, leading to stress concentration at the interface and triggering complex non-local strain effects [91]. This stress concentration can cause interface failure, such as delamination and cracks, reducing the effective interface contact area and manifesting macroscopically as a sudden decline in battery performance. It is worth noting that the mechanical properties of alloys dynamically change with lithiation state [92]. For example, the yield strength of silicon drops sharply from 2 GPa in the original state to 430 MPa after full lithiation, and this softening process may exacerbate plastic deformation and structural collapse of the material. In addition, composite alloy-type anodes usually contain a large amount of solid electrolyte and conductive agents to maintain rapid ion/electron transport, but the content of active materials is usually low (about 30%–80%). This low active material content can lead to electrode structural collapse and interruption of ion/electron transport under high stress [93]. Overall, stress-strain issues are prevalent within the electrode and at the interface, which adversely affects battery performance and urgently needs to be addressed.

#### Slow Electrochemical Kinetics

Alloy anodes have a relatively low intrinsic electronic conductivity due to their own semiconductor properties, which causes multiple kinetic limitations in solid-state batteries [94]. In terms of electron transport, although carbon-based conductive agents can construct electron pathways in electrodes, they can catalyze the early decomposition of electrolytes in sulfide solid-state batteries, especially in the low-voltage range. Meanwhile, the migration of ions in solid-state systems is also restricted. Since solid-state electrolyte cannot flow and permeate like liquid electrolytes, an efficient ion conduction network must be constructed inside the electrode. Unlike liquid electrolytes that can wet electrodes, the mechanical mixing of solid electrolyte particles and alloy particles usually forms a loose and porous structure, and ions need to diffuse along tortuous paths [95]. More seriously, the ionic conductivity drops sharply with the increase in thickness [96]. The diffusion time (τ) of lithium ions in electrode materials can be expressed by the formula τ = L^2^/D (where L is the diffusion length and D is the diffusion coefficient). Therefore, to shorten the diffusion time and improve the electrochemical performance, the key lies in reducing the ion/electron diffusion length (L) of the silicon-based anode and increasing its diffusion coefficient (D). To address these issues, current research mainly enhances the conductivity of alloy anode particles themselves through materials engineering, reduces the amount of carbon additives to avoid side effects, and improves the ion permeation path by optimizing the distribution network of solid electrolytes in the electrode.

#### Low Area Capacity

Although alloy-based electrode materials have a theoretical specific capacity far exceeding that of graphite, in practical applications, they often struggle to achieve the high areal capacity required for commercialization. In order to address the significant volume expansion of alloy during charging and discharging, researchers have developed various nanostructures such as nanoparticles, nanowires, and porous structures [97–99]. These structures, by providing internal space or relieving stress, indeed significantly enhance cycle stability. However, this nanoscale approach also brings about notable negative impacts. Nanomaterials, particularly nanoparticles, due to their tiny size and high specific surface area, cannot be densely packed when compressed into electrode sheets [100]. This results in a lower tap density, limiting the mass of active material that can be loaded per unit volume. In practical battery design, the thickness of the electrode coating is strictly limited. An overly thick coating can lead to difficulties in ion-diffusion and performance degradation. With limited thickness, a low tap density means that the total amount of alloy active material that can be loaded is limited [101]. Alloy-based materials inherently have poor conductivity and require strong binders to maintain structural stability in response to volume changes. Therefore, a large number of conductive additives and binders must be added to alloy-type electrodes. These inactive materials occupy space and weight in the electrode, further crowding out the proportion of active alloy-type materials [102]. Improving the tap density and conductivity of particles allows for higher active material loading and fewer non-active additives, ultimately enhancing the overall areal capacity and volumetric energy density of the electrode (Table 1).

**Table 1:** Alloy anode materials and their electrochemical performance in SSBs

Anode	Solid electrolyte	Voltage range (V)	ICE (%)	Initial capacity (mAh g^−1^)	Cycling stability (RC/CD/CN) (mA g^−1^)	Rate capability (DC/CD)	References
FeSn_2_	Li_6_PS_5_Cl	0.6–1.4	81.5	934.6	818.8/0.93/200	–	[2]
LiSH46	Li_6_PS_5_Cl	2.5–4.2	~ 83.2	3.6 mAh cm^−2^	2.6/1 C/5000	3.4/2 C	[15]
GaSb/C	LiBH_4_	0.6–1.6	97.2	660	650/1.0/400	166/12.8	[31]
SLC	LiPON	0–1.2	51	823	420/0.00196/500	–	[49]
BP/GO	PVDF-HFP	0.005–3.0	–	737	477/0.5/500	158/1.0	[52]
Si(55)/CNF (45)@LPSCl	Li_6_PS_5_Cl	–0.615–0.88	–	1172	728/1.79/50	466/3.57	[53]
P@ZTC	Li_6_PS_5_Cl + Li_3_InCl_6_	0.01–2.0	87.44	1823	1260/0.2/400	1000/2.0	[55]
Bi-LiBH_4_-AB	LiBH_4_	0.2–1.5	96.3	478.7	394/0.1 C/100	–	[77]
nano Si	Li_6_PS_5_Cl	0–1.5	89.3	2412	2375/0.1 mA cm^−2^/10	220/2.0	[104]
Si@SiO_2_@LPO@C	PEO@LATP	0.005–1.5	88.7	2482.1	1012.4/0.5/200	882/1.0	[105]
Si–N-MXene	PEO@LATP	0.005−1.5	82.02	2305	881/0.4/90	304/1.0	[106]
Cu/C/Si	Li_5.5_PS_4.5_Cl_1.5_	2.0−4.5	–	217.4	174.4/0.8 C/ 450	55/2 C	[107]
μSi/SWCT/LPSCl	Li_6_SP_5_Cl	0−1.5	85.4	2974	1596/1 C/400	–	[108]
Si@C@C-10%	PDOL-SN	0.01−3.0	79.8	105.2	86.7/1.0/300	–	[110]
Si-200	Li_6_SP_5_Cl + Li_3_InCl_6_	2.0−4.2	78.12	–	47.88/1 C/150	12.80/4 C	[115]
nSi:mSi = 7:3	Li_2_S-P_2_S_5_	0.02 − 1.2	–	~ 3000	1230/0.12/200	–	[116]
Sn powder	80Li_2_S-20P_2_S_5_	0.01–1.5	78	760	600/0.03 C /100	150/0.15 C	[117]
Si/C fibers	77.5Li_2_S-22.5P_2_S_5_	0.005–1.5	84.2	–	709/0.1/70	–	[122]
col-Si	LPSCl	0.01–2.0	84	3459	2400/0.336/100	1700/2.1	[123]
SiN_0.92_	70Li_2_S-30P_2_S_5_	0.01–2.5	> 80	1800	1300/0.2 C/100	–	[126]
amorphous Si thin films	LiPONB	0.05–1.0	99.9	40 μAh cm^−2^	40 μAh cm^−2^/2 C/1500	20/17 C	[127]
Si(a-Si) film	70Li_2_S-30P_2_S_5_	0.01–1.2	99.0	~ 2800	2000/0.1/100	2400/34 C	[128]
Li_21_Si_5_/Si-Li_21_Si_5_	LPSCl//Li_3_InCl_6_	2.0–4.2	97.7	2.8 mAh cm^−2^	1.54 mAh cm^−2^/2.5 mA cm^−2^/1000	14.5 mAh cm^−2^/9 mA cm^−2^	[130]
a-SiO_0.4_	70Li_2_S-30P_2_S_5_	0.01–1.2	86.2	2835	2657/0.1 mA cm^−2^/100	2500/35 C	[135]
μm-Si	Li_6_SP_5_Cl	0.01–1.5	–	800	570.4/0.15 mA cm^−2^/50	638.4/1.4	[139]
Porous In	Li_10_SnP_2_S_12_	0.01–2.0	80.0	350	300/0.05 C/40	–	[141]
Sn/Graphite-Type 1	Li_3_PS_4_	0.01–1.2	74	470	170/0.03/50	59/0.12	[147]
SiC20	Li_5.5_PS_4.5_Cl_1.5_	− 0.59–0.40	~ 80	902	722 C/20 /50	382/1 C	[148]
Gr/n-Si 90/10	Li_6_PS_5_Cl	0–0.3	~ 83.2	553	2.04/0.5 C/20	–	[149]
PL-DDE 03	Li_6_PS_5_Cl + Li_3_InCl_6_	0–0.8	~ 87.3	602.1 (3.30mAh cm^−2^)	2.81/0.5 C/200	1.90/1 C	[152]
Si/CNTs/C	Li_6_PS_5_Cl	0.01–3.0	59.7	2125	1226/50/50	158/1000	[158]
Sn/PEO-LiClO_4_	PEO-LiClO_4_	0.01–1.5	57.8	–	–	–	[159]
Sn(nano)/C	LiI-Li_2_S-P_2_S_5_	0.01–2.0	56	1266	74/214/100	–	[160]
P@AC	Li_6_PS_5_Cl	0.01–2.0	91.86	1438	962/0.2/400	891/2.0	[163]
3D Ge/Gr	PVDF-HFP/LATP	0.005–1.5	–	1135	860/1.0/2000	805/10.0	[165]
Si@B-C/rGO	LPSC	0.01–2.5	85.6	–	956/0.1 mA cm^−2^/100	–	[167]
Sn-Si/SSE	77.5Li_2_S-22.5P_2_S_5_	0–1.5	–	1000	700/0.08/50	–	[172]
Si@O@Al	LiPON	0–4.2	–	32.8 μAh cm^−2^	–	–	[173]
cAl-Si_LB	LBHI	− 0.595–0.9	94.5	5.3mAh cm^−2^	4.76/0.5 C/300	3.86/1 C	[176]
μSi/GIS-LMA55	LPSCl	0.01–1.5	85.6	3984.7	860.4/1.32/150	2910/2.64	[178]
N_4.4_Sn_4_	Li_6_PS_5_Cl	0.2–2.0	~ 70.6	170	90/0.005/20	–	[180]
Si-Ti-Ni	Li_2_S-P_2_S_5_	0.01–1.0	77.7	483	286/0.04/100	–	[182]
Ag-coated Si	LPSCl	0.01–1.0	79.1	2430	1390/0.2 C/100	1215/1C	[190]
Si@O@Al	LiPON	0–5	–	–	–	–	[191]
BP/NG	LiBH_4_	0–1.6	66.3	1908	50/0.5/100	300/1.0	[195]
Si@LLZTO	LLZTO	0.005–1.5	90.4	2760	1363/1.0/200	1565/2.0	[196]
Si@LiAlO_2_	LPSCl	− 0.595–0.88	80.1	2045	1164/1.17/150	1243/3.5	[198]
PD@Si	LiTFSI/NMA-PEGMA	0.01–2.0	76	2904	1200/0.5/100	1000/1.0	[199]
Si-Li_3_PS_4_-AB	Li_3_PS_4_	− 0.58–0.88	64	2766	2071/0.3 mA cm^−2^/50	–	[201]
n-Si/MWCNT	77.5Li_2_S-22.5P_2_S_5_	0.005–1.5	99	2013	1290/0.12/50	–	[203]
Si-C2mpyrFSI	C2mpyrFSI: LiFSI	0.07–1.0	65.2	1000	961/0.05/60	–	[204]
Si-PAN	77.5Li_2_S-22.5P_2_S_5_	0.1–1.0	~ 84	~ 3579	1606/0.36/200	1500/3.58	[206]
μm-Si/r-LPS/AB	Li_3_PS_4_	− 0.62–0.88	95	3405	1700/0.3/375	550/10 mA cm^−2^	[207]
Si-CNT	LLZAO	0.001–1.5	83.2	2685	2103/0.0075/2000	–	[246]
Si@VG	PEO	0.01–1.5	83.4	1465	444.9/0.5/200	1116.4/1.0	[248]
MSi-C	PVDF-HFP/LATP	0.005–1.5	83.2	2137	1135/1.0/500	1793/3.0	[249]
Amorphous Si	PPCL-SPE	0.05–1.5	68.0	2520	2220/0.1 C/200	1342/1 C	[250]
Si-graphite	Li_6_PS_5_Cl	0.01–2.0	–	5.83mAh cm^−2^	–	4.59/3.13	[251]
SNF@PAN-E	PAN-E	0.01–3.0	–	308.0	429.0/1.0/1200	194.1/1.0	[252]
Si-SHDSE	SHDSE	0.01–1.5	83.2	3304.1	1772.4/1.0/500	816.4/10.0	[253]
thin silicon wafers	LPSCl	0–1.0	–	10 mAh cm^−2^	10 mAh cm^−2^/0.5 mA cm^−2^/100	8 mAh cm^−2^/1.0 mA cm^−2^	[254]
99.9wt% mSi	LPSCl	2.0–4.3	76	2890	1000/5 mA cm^−2^/500	1250/5 mA cm^−2^	[255]
Si@MgO@C	PEO-LATP-NCF	0.005–1.5	81.4	3224.6	1658.9/0.3/100	1299.2/1.0	[256]
μ-Si	Li_6_PS_5_Cl	− 0.6–0.8	86	3412	–	–	[257]
μ-Si	Li_6_PS_5_Cl	− 0.61–0.88	86.34	3752.2	335.1/0.5/50	604.5/1.0	[258]
LPSCl-infiltrated m-Si	Li_3_PS_4_	0.01–1.2	88.7	3246	2300/0.2 C/30	–	[259]
SiMg5.0	75Li_2_S- 25P_2_S_5_	− 0.58–0.88	71	1729	1038/0.026/200	–	[281]
μm-Si-elastic electrolyte	poly-DMAM/poly-AM	0.02–2.0	87.1	2973	1039.7/1.43/300	890.5/1.88	[283]
Si-SE-C	Li_5.4_PS_4.4_Cl_1.6_	0–1.5	88.7	2917	1137/1.42/50	1985/1.42	[285]
Si thin-film	LiPON	3.0–4.55	89.4	42 μAh cm^−2^	35/0.028/30	–	[290]
Li_2_SiS_3_ + FeS thin film	70Li_2_S-30P_2_S_5_	0–2.5	~ 100	1300	–	–	[291]
Si film	LiPON	0.5–4.55	89.4	32.8 μAh cm^−2^	7.7 μAh cm^−2^/0.3 μA/30	–	[298]
Si@MOF	PPG	0.01–1.5	72.0	1416	1442/0.2/50	–	[304]
n-Si/LPSCl	LPSCl	− 0.595 to 0.9	88.11	3554.5	903/0.3 C/100	75.4/2 C	[307]
Li_4.4_Si	Li_2_S-P_2_S_5_	1.0–2.5	71	210	150/130 μA cm^−2^/10	90/260 μA cm^−2^	[310]
SP-MSi/C	LPSCl	0.01–1.5	87.8	3423	864/2.1/700	–	[315]
SiNWs	LPSCl	− 0.6 to 1.0	78	2600	425 C/10/50	–	[317]
Ga−In liquid metal	Li_6_PS_5_Cl	0.01–2.0	–	389	198/0.4175/500	51/1.670	[319]
Bi@Li_3_PO_4_	Li_2_S-P_2_S_5_	0.4–2.5	–	246	211.6/0.025/150	157.4/0.2	[321]
Si/SiCO	LiAlPON	1.6–3.2	–	190.1	50/0.1 C/100	120/1 C	[322]
Si/C	PEO-LiTFSI	0.01–1.2	–	2750	1000/0.8/100	1300/0.8	[325]
Micro-Si@ Li_3_PO_4_@C	3D-PPLLP	2.8–4.3	83.3	129.1	127.2/0.2 C/100	92.5/2.0	[329]
Si@MHF	SPE	0.01–1.5	–	1500	1266/0.2/200	770/5.0	[332]
Li_13_Si_4_	LPS	0.5–3.7	–	1179	1179/130 μA cm^−2^/80	–	[337]
Porous Amorphous Si Film	80Li_2_S- 20P_2_S_5_	0.01–1.2	–	~ 3200	2962/2.19 mAh cm^−2^/100	3000/17 C	[344]
SnMAG	LPSI	0.01–2.0	88.9	455.6	380/0.033 C/30	215/0.1 C	[347]
Si–C	SSE-PIB-PP	0.01–2.0	80.5	1305	0.83mAh cm^−2^/0.1 C/400	0.57 mAh cm^−2^/2 C	[349]
Si–C	77.5Li_2_S-22.5P_2_S_5_	0.005–1.5	83	1858.7	1089.2/0.38/100	–	[353]
SiO_x_@C	PVDF/LLZO	0.005–1.5	74	1531	1224/0.2/50	612/1.0	[354]

### Modification Strategies of Alloy Anode in Solid-State Lithium-Ion Battery

**Fig. 3:**
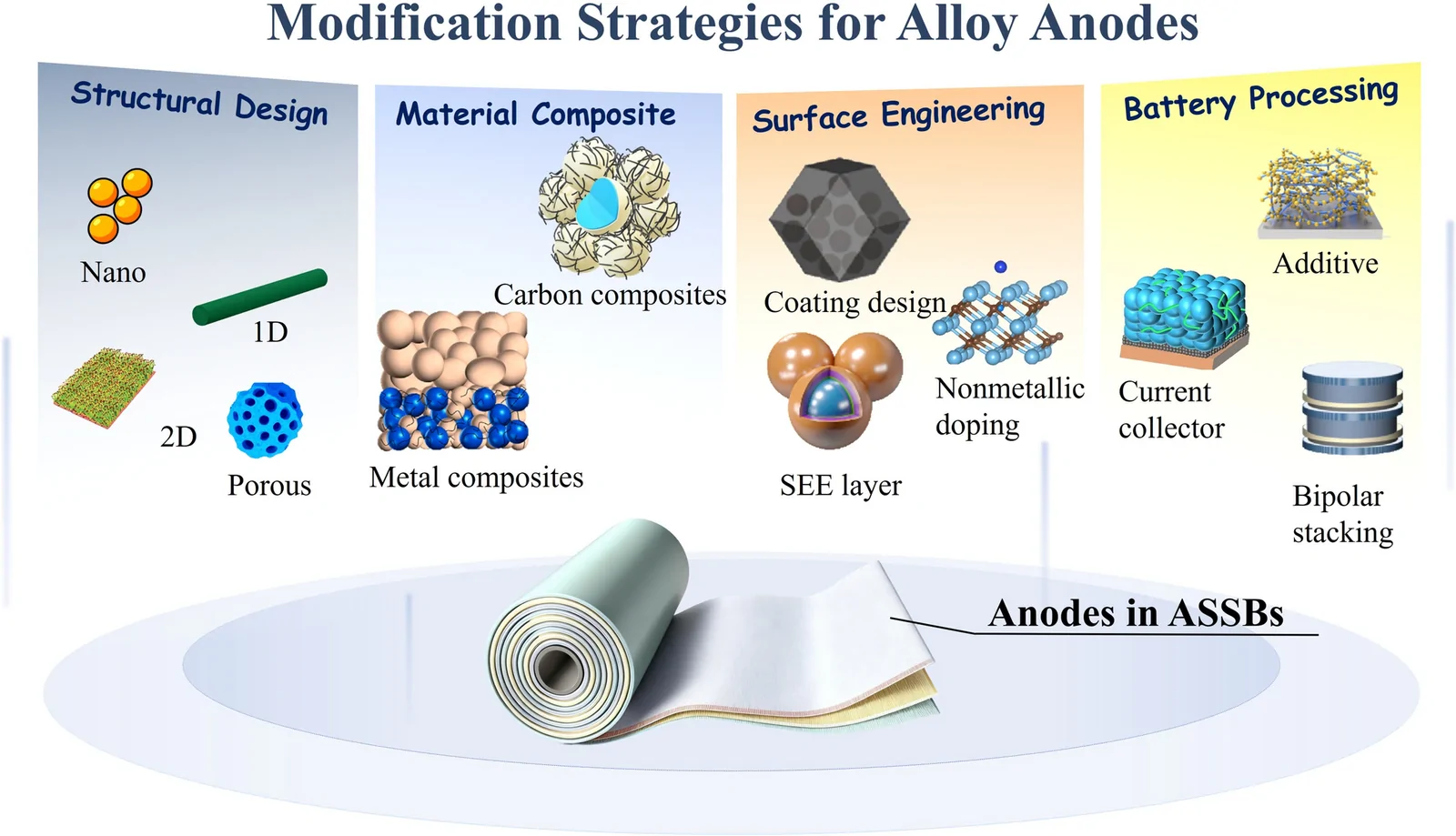
Schematic diagram of modification strategies for alloy anodes of solid-state lithium-ion batteries [2, 103–110]. © 2020 WILEY–VCH Verlag GmbH & Co. KGaA, Weinheim. © 2024 Elsevier Inc. © 2025 American Chemical Society. © 2024 Elsevier Ltd. All rights are reserved. © 2021 Science Press and Dalian Institute of Chemical Physics, Chinese Academy of Sciences. Published by ELSEVIER B.V. and Science Press. All rights reserved. © 2022 Elsevier B.V. All rights reserved. © 2021 Elsevier B.V. All rights reserved. © 2024 Elsevier B.V. All rights are reserved. © 2022 Elsevier B.V. All rights reserved

To realize the practical application value of its ultra-high theoretical capacity, researchers have invested a lot of effort in solving the inherent fatal defects of alloy-type anodes and improving their electrochemical performance. Through modification methods, the three core problems of alloy-type anodes, namely volume expansion, poor conductivity, and low areal capacity, have been systematically solved, achieving a balance of high capacity, long life, and fast charging. Currently, mainstream modification strategies include structural design, material compositing, surface engineering, and the regulation of other characteristics such as battery composition (Fig. 3) [2, 103–110]. Without these in-depth and systematic modification strategies, alloy-based anodes cannot be applied in practice. The significance of these strategies is not only reflected in improving the performance of silicon anodes, but also has a revolutionary impact on promoting the commercialization process of next-generation high-energy-density lithium-ion batteries.

#### Structural Design

##### Size Effect

**Fig. 4:**
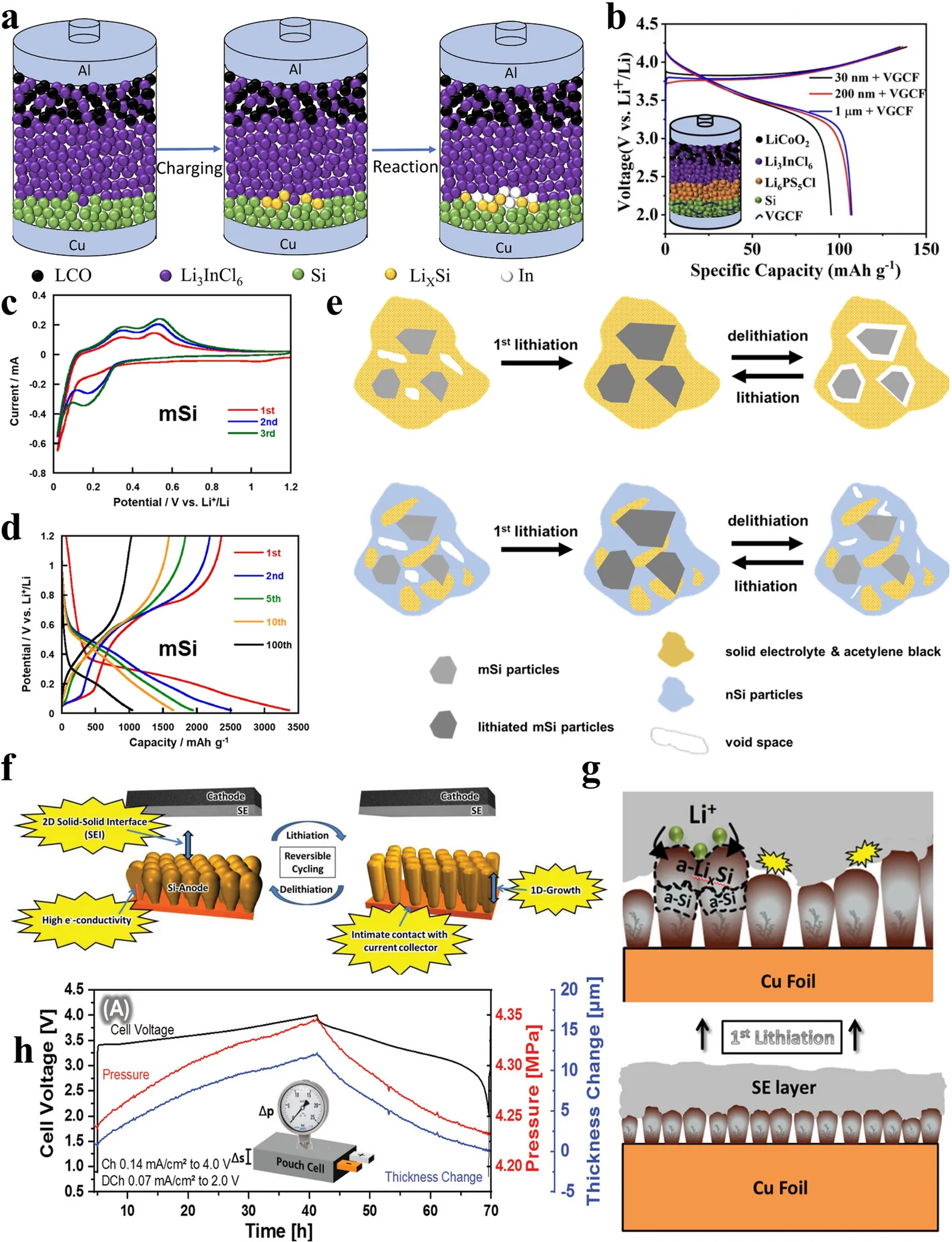
**a** Schematic diagram of products after the reaction of Li_3_InCl_6_ and with Li_4.2_Si. **b** Charge-discharge curves at 0.1 C of different particle sizes (Si-30, Si-200, Si-1000) [115]. © The Author(s), under exclusive licence to Springer-Verlag GmbH Germany, part of Springer Nature 2024. **c** CV and **d** charge/discharge curve of mSi electrodes. **e** Schematic image of the after-cycling with mixed micro-sized and nano-sized silicon particles [116]. © 2024 Elsevier Ltd. All rights are reserved. **f** Schematic diagram of the mechanism of col-Si. **g** Illustration of morphological and phase changes in col-Si. **h** The first cycle test was conducted on NCM/SE/col-Si pouch cells that were cycled in a special test device [123]. © 2020 The Authors. Published by WILEY–VCH Verlag GmbH & Co. KGaA, Weinheim

The particle size of alloy-type materials determines their electrochemical performance and is the physical basis for overcoming severe volume expansion [111, 112]. When the particle size is reduced to the nanometer level, the internal stress of the material significantly decreases during lithium intercalation expansion. Large particles of silicon rapidly pulverize and crack due to repeated expansion and contraction during the cycle [113]. Nanoparticles can release stress and inhibit crack propagation through surface energy, thus avoiding structural collapse. Secondly, compressing the particles from the micrometer level to the nanometer level lead to shortening the diffusion path and increasing the migration speed of lithium ions [114]. In addition, nanosizing causes the specific surface area to increase by orders of magnitude, exposing a large number of reaction interfaces and significantly enhancing the reaction kinetics of lithium-ion intercalation/deintercalation. Li team reported the key impact of the selection of Si anode particle size on the conductivity and overall performance of ASSBs (Fig. 4a) [115]. The surface of small-sized silicon particles (30 nm) forms a more uniform and denser layer of silicon oxide (SiO_2_). Although this oxide film has a certain protective effect, it mainly plays the role of an insulating layer that hinders the transmission of lithium ions, thereby increasing the interface impedance. The oxide layer on the surface of large-sized silicon particles (1 μm) is generally uneven, which hinders ion conduction and shows good electrochemical reversibility (Fig. 4b). The properties of the surface oxide layer of medium-sized silicon particles (200 nm) lie between the two. In addition, 30-nm-sized nano-silicon particles exhibit a relatively low tapped density due to their large specific surface area and numerous voids between particles. Although large-sized micrometer particles have a large individual volume, due to their irregular shapes, their tapped density is also not ideal. Medium-sized silicon particles at 200 nm have better particle filling properties, and thus have the highest tapped density. Comprehensive research has found that in ASSBs, 200-nm sub-micron silicon particles exhibit the most outstanding performance due to their moderate tap density, appropriate surface oxide layer characteristics, and low volume expansion rate. The ICE of Si (200 nm) reached 78.37%, which was much higher than that of Si (30 nm) and Si (1 μm). The cycle stability was also the best, and its volume retention rate after 150 cycles was also much higher than that of the other two. This research provided an important theoretical basis and practical reference for designing the particle size of high-performance all-solid-state alloy-based anode materials. In addition to alloy material particles of different sizes, recent research has reported an innovative strategy based on mixtures of different sizes for all-solid-state lithium batteries [116]. The study found that two single-sized silicon particles exhibited almost opposite characteristics. Micron-sized silicon particles (mSi) exhibit a very high initial discharge capacity (~ 2400 mAh g^−1^), but as the number of charge/discharge cycles increases, their discharge capacity significantly decreases. Conversely, nano-sized silicon particles (nSi) have a lower initial discharge capacity (~ 1000 mAh g^−1^), but surprisingly, their capacity shows an upward trend during the first approximately 20 cycles, and then exhibits better capacity retention over a longer period of 200 cycles. Therefore, in order to effectively alleviate the sharp capacity changes of single components during the initial stages of cycling and simultaneously achieve high initial capacity and good cycling stability, researchers attempted to mix nSi and mSi in different proportions. The results showed that when the mass ratio was nSi: mSi = 7: 3, the anode exhibited the best balanced performance (Fig. 4c, d). It not only had a high initial capacity, but also showed the highest final retained capacity (72%) after up to 200 cycles of testing. This happened because in the mixed structure, micron-sized mSi particles still provided the main high-capacity foundation, while nano-sized nSi particles and conductive agents (AB) surrounding them jointly formed a relatively dense matrix or buffer layer. This buffer layer played a key role in effectively absorbing the stress generated by the dramatic volume changes of mSi particles during charging and discharging. It reduced the formation of destructive voids and cracks around the mSi particles, thus maintaining good ionic and electronic conduction paths. Therefore, the mixed anode not only utilized the high-capacity potential of mSi, but also improved the mechanical stability and interfacial contact continuity of the structure with the help of nSi, ultimately achieving superior comprehensive electrochemical performance compared to the single-sized silicon particle anodes (Fig. 4e).

Interestingly, it has been found that in solid-state electrolyte systems, the Sn anode exhibits cycle stability that is difficult to achieve in traditional liquid electrolyte systems [117]. The fundamental mechanism behind this stability lies in the unique volume adaptability behavior of the Sn/solid-state electrolyte composite material. During the charge-discharge cycle, the electrode spontaneously densifies, effectively maintaining the integrity of the interface contact. Unlike the hollow structure design designed to buffer volume changes in liquid systems, the original Sn powder in solid-state batteries can achieve stable cycling, breaking through the trade-off limitations between net capacity and cycle life of traditional high-capacity anode materials. In addition, experiments have confirmed that the charge transfer impedance at the electrode/electrolyte interface is negligible, but bulk diffusion becomes the main rate-limiting step. The Sn thin-film electrode exhibits excellent rate performance in the solid-state system, while conventional micron-sized Sn powder rapidly degrades to 150 mAh g^−1^ at a rate of 0.15 C, revealing the decisive influence of material size effect on lithium-ion diffusion kinetics. Through comparison, it was found that the ion migration impedance of the solid-state electrolyte itself dominates the overall overpotential, but the transport limitation of lithium ions within the particles is the main cause of capacity degradation, providing an optimization direction for high-rate anode design. This reveals the dual nature of electrode material volume changes in solid-state battery systems. Macroscopic expansion/contraction is not only non-maleficent, but also forms a stable interface through a self-adjusting densification mechanism, providing a new idea for the application of high-capacity alloy anodes.

##### 1D Structure

Fibrous structure design is an important strategy to enhance the performance of lithium-ion battery anode materials. The fibrous structure can exert mechanical flexibility and conductive penetration, forming a skeleton like support advantage for the electrode, enabling the alloy anode to maintain the integrity of the electrochemically active network during repeated expansion [118–120]. In addition, designing the fiber diameter size has significant advantages in regulating the interface, suppressing volume effects, and optimizing ion diffusion balance [121]. Kim and team explored the performance of nanostructured silicon-carbon composite fibers as anode materials for all-solid-state lithium-ion batteries [122]. Although electrode A with a fiber diameter of about 2 μm exhibited the highest discharge/charge capacity during the initial cycle, its capacity rapidly decayed. In contrast, electrode B with a fiber diameter of about 1 μm and electrode C with a fiber diameter of about 0.1 μm showed significantly improved electrochemical stability with decreasing fiber diameter under the same composition conditions. Electrode C exhibited the best cycling performance: after 70 cycles, its discharge capacity reached 714 mAh g^−1^, charge capacity reached 709 mAh g^−1^, coulombic efficiency was as high as 99.2%, and it maintained 80% capacity after 5 cycles. This performance is attributed to the multiple advantages brought by small-diameter fibers. Small-diameter fibers effectively limit the agglomeration behavior of silicon nanoparticles during cycling, which greatly reduced the cracking, crushing, and subsequent electrochemical isolation of active materials due to volume expansion. Small fibers helped maintain the stability of the discharge platform voltage, with a phase change peak (about 0.4 V) voltage drift of only 0.004 V, much smaller than the 0.031 V shift of electrode A, thus maintaining the stability of the voltage platform. Although electrode C achieved a high reversible capacity of over 700 mAh g^−1^, there was still a slow capacity decay mechanism during 70 cycles, which is related to its high silicon content ratio and the possible evolution of silicon particles into long-chain structures after cycling. Therefore, focusing on deeply understanding the evolution mechanism of silicon microstructure during long-term cycling is the research focus for future solid-state energy storage anode materials.

In addition, the volume effect of silicon is transformed into a controllable one-dimensional deformation through geometric constraints and pressure management, while the intrinsic stability of solid electrolytes is utilized to construct highly reliable interfaces. In Cangaz's research, columnar silicon anodes were prepared by physical vapor deposition (PVD), demonstrating a unique columnar microstructure (Fig. 4f) [123]. This work demonstrates for the first time the feasibility of columnar silicon anodes in practical high-load (3.5 mAh cm^−2^) solid-state batteries. The design alleviates the inherent volume expansion problem of silicon materials through a "one-dimensional breathing mechanism" that expands and contracts only in the vertical direction (Fig. 4g). When coupled with thiophosphate electrolytes and nickel-rich cathodes, a stable two-dimensional solid-solid interface was formed. External mechanical pressure (20 MPa) maintained close contact at the interface and limits lateral deformation. At an industrial-grade surface capacity of 3.5 mAh cm^−2^, after 100 cycles, the capacity retention rate was 92.8%, and the coulombic efficiency reached 99.7%–99.9%, supporting a charging current of 0.9 mA cm^−2^ at room temperature (Fig. 4h). Compared with liquid systems, solid-state design avoided the depletion of electrolyte and the continuous formation of SEI during the cycle. In situ monitoring confirmed that a constant pressure of 20–25 MPa can suppress interface delamination, thereby increasing the capacity retention rate of pouch cells to 83–95% after 50 cycles.

##### 2D Structure

The two-dimensional thin-film structure can also convert cyclic stress into reversible bending deformation through a wavy deformation mechanism driven by geometric mechanical mismatch, thus alleviating the stress failure of the alloy-based electrode [124]. Two-dimensional thin film materials form mechanically stable layered stacks through van der Waals forces or chemical bonds, thereby preventing electrode cracks under high loading conditions. In all-solid-state lithium batteries, thin-film electrodes break through the kinetic and mechanical performance bottlenecks of traditional electrodes through nanoscale structures, gradient designs, and composite materials [125]. Suzuki and team fabricated a 200 nm non-stoichiometric silicon nitride thin film (SiN_0.92_) using pulsed laser deposition (PLD) technology [126]. Electrochemical tests showed that this material exhibited significant redox activity in the potential range below 0.5 V (vs. Li^+^/Li), with an initial charge capacity of up to 1800 mAh g^−1^. After 100 cycles, it still maintained 1300 mAh g^−1^, demonstrating a theoretical capacity far exceeding that of graphite anodes and showed excellent cycling stability. During the lithiation process of SiN_0.92_ material, it did not generate the traditional conversion reaction products Si and Li_3_N, but achieved reversible conversion by forming a ternary covalent compound of lithium, silicon and nitrogen (such as Li_2_SiN_2_) instead. The synergistic effect of this intermediate phase with the subsequent silicon-lithium alloying reaction provided a high specific capacity. Meanwhile, the rigid matrix of the ternary compound effectively buffered the volumetric strain during the alloying process and inhibited electrode powdering. SEM (scanning electron microscopy) observation confirmed that there were no obvious cracks on the electrode surface after cycling, which corroborated the structural stability. In addition, the application of sulfide solid electrolyte solid-state battery systems further suppressed interfacial side reactions and enhanced cycling performance. In summary, nitrogen-rich silicon-based thin films have solved the bottleneck of capacity attenuation of silicon anodes through a reversible conversion-alloying coupling mechanism, while they possess both high energy density and long cycle life. The continuous development of microelectronic systems has put forward higher requirements for the performance of all-solid-state thin-film lithium microbatteries. Phan et al. innovatively constructed a Li/LiPONB/Si all-solid-state battery structure by using physical vapor deposition technology [127]. Within the optimized 50 mV–1 V potential window, this system achieved a breakthrough effect of zero capacity attenuation after 1,500 cycles, and the coulombic efficiency remained consistently above 99.99%. SEM analysis confirmed that despite the significant volume change caused by the lithiation process, no cracks or structural damage occurred at the silicon film or at its interface with the LiPONB solid electrolyte. This extraordinary stability stemmed from three key mechanisms: (1) The isotropic expansion property of amorphous silicon thin films; (2) Vertical displacement mechanism of the electrode/electrolyte interface in uniaxial expansion mode; (3) The strong interfacial bonding force between the LiPONB electrolyte, whereas the silicon film effectively inhibited crack initiation and simultaneously avoided the negative impact of side reaction products in the liquid electrolyte. Solid electrolytes have achieved a revolutionary breakthrough in the application of silicon anodes in micro-energy storage devices by eliminating the erosion of electrode structures by liquid reaction by-products, combined with optimized LiPONB film thickness, voltage threshold control and film geometry design.

As early as 2014, the first realization of intrinsic high-performance output from additive-free pure silicon thin films provided a new paradigm for all-solid-state battery design. Miyazaki et al. prepared high-purity amorphous silicon (a-Si) thin films with a density of 2.3 g cm^−3^ through radio frequency magnetron sputtering technology [128]. Combined with 70Li_2_S-30P_2_S_5_ sulfide glass ceramic solid-state electrolyte, the excellent electrochemical performance and inherent mechanism of the additive-free pure silicon system were revealed. At a current density of 10 mA cm^−2^, the discharge capacity remained at 2400 mAh g^−1^, which was much higher than that of traditional liquid electrolyte systems. The performance was attributed to the ultra-low impedance of the solid-state interface, with the electrolyte bulk impedance being the dominant factor, while the interfacial charge transfer impedance was negligible. Compared with composite systems containing FeS additives, pure a-Si exhibited equally excellent rate performance, confirming that the intrinsic high electronic conductivity of lithium-silicon alloy was the core factor, without the need for additional conductive agents for reinforcement. The 50 nm thin film maintained a capacity of 2500 mAh g^−1^ (initial capacity of 2800 mAh g^−1^) after 100 cycles at 0.1 mA cm^−2^, with a coulombic efficiency of > 99%. The solid-state electrolyte effectively avoided the continuous capacity fading and impedance increase caused by the repeated formation of SEI in liquid systems, highlighting the advantage of electrochemical stability at the solid-state interface. Subsequently, researchers developed amorphous silicon carbon composite films (aSi/C) using co-sputtering technology [129]. By adjusting the sputtering power ratio (Si_63_C_37_, etc.), the composite film formed a uniformly distributed amorphous nanocomposite structure. The structure exhibited the combined effect of the high theoretical capacity of silicon and the buffering function of the carbon matrix during electrochemical cycling. The carbon phase effectively buffered the volume change during insertion and extraction of lithium through physical confinement effects, while improving overall conductivity. Compared with pure silicon electrodes and pure carbon electrodes, the preferred composition Si_63_C_37_ exhibited the best long-term cycle stability (capacity retention rate of 85% after 100 cycles), excellent rate performance (capacity retention rate of 89% at a current density change of 160 μA cm^−2^), and significant inhibition of initial irreversible capacity loss.

Compared to pure alloy anodes, lithium-alloy compounds can effectively mitigate initial irreversible capacity loss, reduce voltage rise at the end of discharge, decrease capacity fading, and prolong cycle performance [130, 131]. However, the structure of lithium-alloy compounds prepared by traditional electrochemical lithium insertion methods is complex and difficult to control. Shifting to physical deposition methods seems to be a more suitable approach to achieve precise synthesis of amorphous thin films. For this reason, the Strauß's team developed lithium silicon (Li–Si) compound thin films using reactive ion beam co-sputtering technology [132]. Experimental results showed that the obtained thin films exhibited significant amorphous structural characteristics. Further investigation using depth profiling secondary ion mass spectrometry (SIMS) and X-ray photoelectron spectroscopy (XPS) revealed complex layered compositions. A lithium silicon alloy layer (Li_x_Si) with a thickness of approximately 120 nm was observed near the interface, which was enriched in silicon but contained low lithium (lithium-silicon molar ratio x ≈ 0.4). Its surface was covered with a graded oxide layer (Li_x_SiO_y_) with a thickness of approximately 10 nm. The oxide layer formed after exposure to air exhibited lithium-rich characteristics, attributed to lithium diffusion from the oxygen-rich surface to the inner layer to reduce the chemical potential gradient. Its XPS analyses showed that the layers were dominated by Li–O bonds (binding energy 57.0 eV), accompanied by weak SiO_y_ signals. The inner Li_x_Si region showed stable coexistence of Li–Si bonds and Si–Si bonds. The oxide layer may act as an in situ SEI precursor, thus enhancing stability during electrochemical cycling. Meanwhile, lithium concentration gradients induced diffusion at room temperature, indicating that the Li–Si system is kinetically prone to ion migration, which is crucial for designing high-capacity anodes.

Alloy anode materials generally face the challenge of insufficient cycle stability. An effective solution is to introduce a convertible electrode system. The system uses oxide MO (where M represents a metal element that can form an alloy with lithium) in the initial state of the battery. During the first charging process, MO undergoes an electrochemical reaction, generating the elemental metal M and the lithium compound Li_2_O [133]. In the subsequent charge-discharge cycles, the elemental M undertakes the main electrode function through alloying/dealloying reactions with lithium, while Li_2_O exists as an inactive supporting skeleton. The key advantage of this in situ transformation reaction lies in the fact that it transforms the active metal M into nanoscale particles and uniformly disperses them in the inert matrix Li_2_O. The unique nanocomposite structure greatly alleviates the inherent problem of cycling performance degradation caused by drastic volume changes in alloying reactions [134]. For example, using SiO_x_ anode instead of Si anode is an effective way to improve the cycling performance of alloy anodes. Miyazaki et al. reported silicon-enriched amorphous silicon oxide (a-SiO_x_) films based on the previous a-SiO_x_ films, which were applied as anodes in all-solid-state lithium batteries [135]. The electrochemical behavior of a-SiO_x_ thin films in solid-state environments benefits from a unique transformation reaction mechanism. During the initial charging process, an irreversible transformation reaction of a-SiO_x_ occurs, generating electrochemically inert scaffolds of nano-silicon particles and Li_2_O as the main lithium oxide/silicate composite phase. This bracket, on the one hand, provides mechanical support, alleviates the stress concentration caused by the volume expansion of silicon particles during charging and discharging, and inhibits material pulverization. On the other hand, the synergistic effect with solid electrolytes significantly reduces interfacial side reactions, keeping the electrode in an efficient ion/electron transport channel. Therefore, the first-cycle discharge capacity of the a-SiO_0.4_ film reached 2835 mAh g^−1^, and the capacity retention rate after 100 cycles was as high as 94%, with an average capacity attenuation rate of only 0.06% per cycle. At a high current density of 10 mA cm^−2^, it could still release a capacity of approximately 2500 mAh g^−1^, which was comparable to pure amorphous silicon, confirming that the inert scaffold did not impede the ion conduction kinetics.

##### 3D Structure

Although nanostructures can greatly overcome the particle powdering and electrode failure caused by volume expansion, the preparation cost of nanoalloy materials is high. The low tapped density and extremely high specific surface area result in low initial coulombic efficiency, which limits their industrial-scale application in SSBs. The main advantages of micron alloy materials over nano-alloy materials are reflected in production cost, processing performance and volume energy density [136, 137]. For instance, micron silicon can be directly obtained through mechanical crushing without the need for complex nanoscale processes. The specific surface area of micron silicon is much lower than that of nanometer silicon, and the particles of micron silicon are packed more closely [138]. This means reducing the irreversible lithium consumption of the SEI film during the first charge and discharge, and having better dispersion to avoid agglomeration. Poetke et al. reported micrometer-sized silicon particles (μm-Si) as anode materials for high-energy solid-state lithium-ion batteries and proposed a "partial lithiation" solution [139]. The lithium intercalation capacity of silicon particles was controlled at 800 mAh g^−1^, approximately 22.3% of the theoretical specific capacity. Compared with complete lithiation (3579 mAh g^−1^), this strategy could significantly reduce the volume expansion rate from 300 to 66%. By analyzing the nickel-rich layered oxide cathode (LiNi_0.9_Co_0.05_Mn_0.05_O_2_) and the NCM/SE/μm-Si full cell composed of the sulfur silver germanium mineral type solid electrolyte (Li_6_PS_5_Cl), it was found that the edge regions of partially lithiated silicon particles preferentially formed amorphous a-Li_x_Si. This system also maintained a reversible lithium-ion intercalation/deintercalation process in subsequent cycles. Research has found that reducing the full battery charging cut-off voltage (from 4.25 to 4.00 V) has a crucial impact on battery stability. The regulation reduced the charging cut-off potential of the cathode from 4.375 to 4.126 V, effectively suppressing the structural distortion and phase transition of the nickel-rich cathode at high voltages, and simultaneously reducing interfacial side reactions. Experiments have proved that when the charging cut-off voltage is 4.00 V, after 50 cycles of the battery without active pressure control, the capacity retention rate can reach 71%, which is much higher than 32% in the high cut-off voltage system. In addition, this μM-Si-based solid-state battery system demonstrates a significant advantage in energy density. Compared with traditional graphite anodes, its volume energy density increased by 28% (766 Wh L^−1^), and its mass energy density reached 258 Wh kg^−1^. This technical route ensured cycle stability while taking into account industrial-grade cost-effectiveness and energy density improvement through the electrochemical process of synergistically optimizing the material design of micron-scale silicon and partial lithiation and regulating the charging cut-off voltage.

**Fig. 5:**
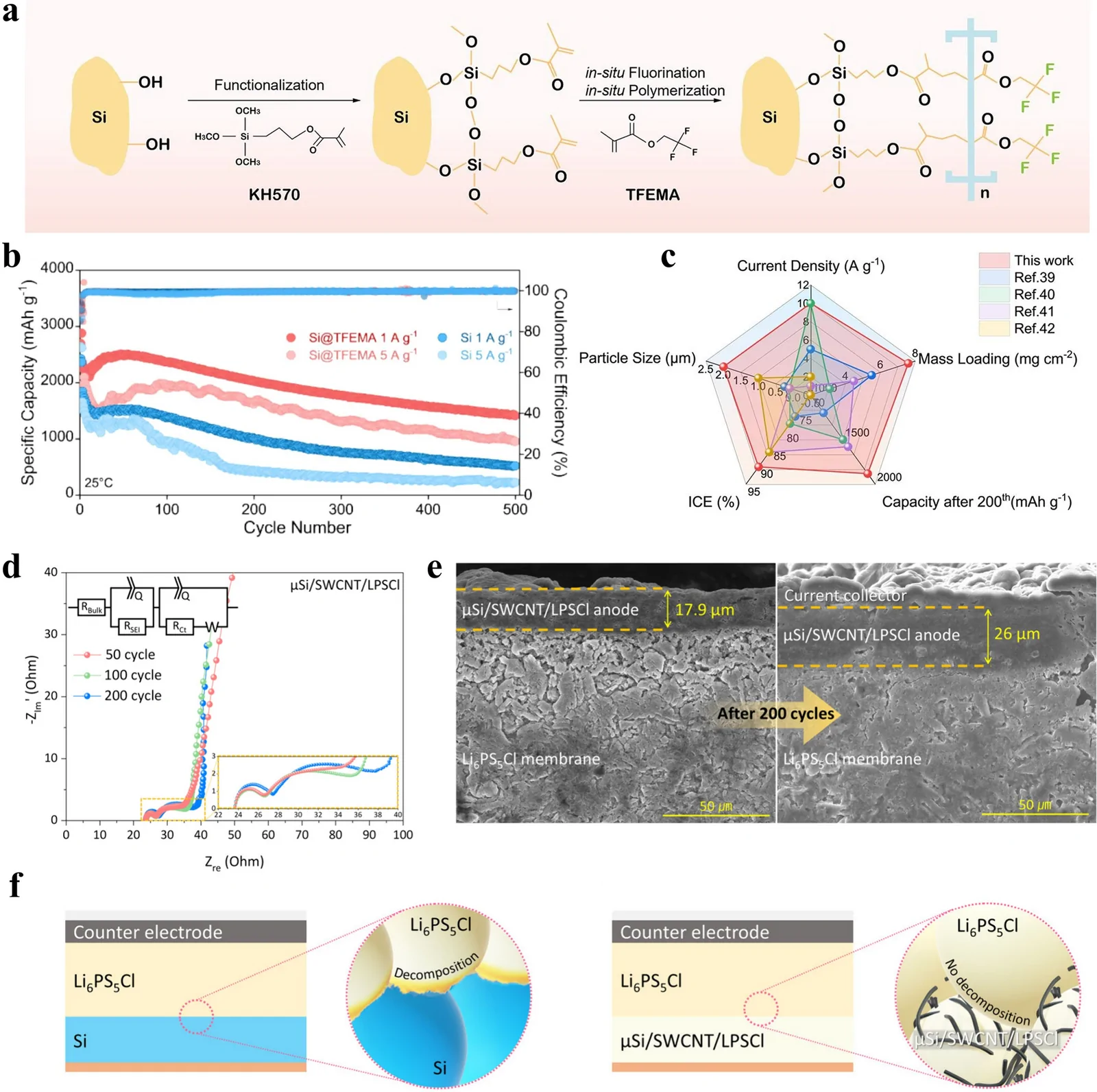
**a** Schematic diagram of the synthetic route of Si@TFEMA. **b** Cyclic performance and **c** comparison of the performance of Si@TFEMA with other Si materials [140]. © 2025 Elsevier B.V. All rights are reserved. **d** Representative Nyquist plots and **e** cross-sectional SEM images of μSi/SWCNT/LPSCI. **f** Schematic of the unstable interface between Li6PS5Cl SE and Si or μSi/SWCNT/LPSCl anode [108]. © 2025 American Chemical Society

In addition, with the rapid development of photovoltaic energy, the global photovoltaic industry generates a large amount of silicon waste. The traditional landfill treatment method not only occupies land but also pollutes groundwater. Recycling and reusing silicon waste generated by the photovoltaic industry can alleviate the raw material anxiety of lithium-ion batteries and the waste anxiety of photovoltaic waste, eliminate pollution sources, as well as promote the low-carbonization of the new energy industry. Recently, the Ma and team proposed an innovative surface modification strategy for the recycling and utilization of micron-sized silicon waste generated by the photovoltaic industry [140]. The surface of silicon particles was pre-treated with silane coupling agent KH570, and then ethyl trifluoromethyl acrylate (TFEMA) was grafted in situ by radical polymerization to form an organic-inorganic composite coating with a cross-linked network structure (Si@TFEMA), as shown in Fig. 5a. After modification, the initial effect was increased to 90.2%, and the capacity remained at 1647 mAh g^−1^ after several cycles at the current density of 1 A g^−1^, which was 2.4 times that of the original silicon (Fig. 5b). The pouch battery assembled with LiFePO_4_ achieved a capacity retention rate of 89.7% after 100 cycles at 0.2 C, and the rate test tolerated a current of 3 C. For the first time, it was matched with PVDF(polyvinylidene fluoride)/LITFSi-based polymer solid electrolytes, outputting a specific capacity of 1000 mAh g^−1^ and stably cycling for 100 times, demonstrating high safety potential. This is because the design integrates multiple advantages: 1) The elastic polymer layer can effectively buffer the volume expansion of silicon during the cycling process, increasing the DMT modulus to 703 MPa. 2) Cross-linked networks confine silicon particles within the polymer matrix, thus reducing the generation of "dead silicon" caused by the shedding of active substances. 3) The fluorine-terminated–CF_3_ groups of TFEMA induce the preferential decomposition of fluoroethylene carbonate in the electrolyte at the interface, promoting the formation of a stable SEI film rich in LiF. Compared with other photovoltaic silicon waste (Fig. 5c), Si@TFEMA shows superior electrochemical performance. The technology converts silicon waste into high-performance anodes through low-cost and scalable surface engineering, providing an environmentally friendly and economically valuable solution to the problems of solid waste pollution in the photovoltaic industry and interface failure of silicon-based anodes in lithium batteries. In Kim's research, they proposed a ternary composite anode material (μSi/SWCNT/LPSCl), the core of which is to composite microsilicon particles (μSi), single-walled carbon nanotubes (SWCNTs), and sulfide solid electrolyte (Li_6_PS_5_Cl, LPSCl) together through a simple dispersion-calcination process [108]. This design combined the high electronic conductivity of SWCNTs with the high ionic conductivity of LPSCl, while the LPSCl layer suppressed the side reactions at the interface between silicon and the electrolyte (Fig. 5d-f). The discharge/charge capacity of the first cycle at 0.1 C reached 3483/2974 mAh g^−1^ (ICE = 85.4%), and the capacity retention rate was 54% after 400 cycles. It still maintained a high reversible capacity of 1271 mAh g^−1^ at a high rate of 1 C. The volume expansion rate dropped from 68% of pure silicon to 45.2%, and no cracks occurred, confirming that LPSCl coating effectively alleviated mechanical stress (Fig. 5e, f). Micron alloy anodes have overwhelming advantages in terms of cost, processing performance and volumetric energy density. Therefore, micron alloy anodes have become the ideal choice for the industrialization and commercialization of all-solid-state lithium-ion batteries in the short term. The requirements for its preparation and synthesis processes are low cost and scalable. At the same time, it is necessary to further solve the volume effect and interface problems of the micron-structured anode in ASSLIBs through structural modification and surface engineering, and promote the practical application process of high-energy-density solid-state batteries.

Porous structures, on the other hand, have a unique mechanism in alleviating interfacial strain, maintaining structural integrity and enhancing energy density in solid-state batteries. For example, Han et al. successfully prepared indium (In) and tin (Sn) metal foils with a double continuous ligament-pore structure [141]. Chemical dealloying of lithium-rich alloys (Li-In/Li-Sn) was carried out at 60 ℃ using dry methanol solvent, and a controllable nano-micron-scale pore structure could be achieved without an external potential. Porous indium foil exhibited significantly superior electrochemical performance compared to dense indium foil in solid-state batteries. On the one hand, it provides a buffer space for volume changes. Its porosity (68%) and micron-scale ligament structure provided an effective buffer space for volume expansion, solving the problem of chemical and mechanical degradation caused by electrode volume changes in solid-state batteries. On the other hand, it only forms a planar contact interface with the solid electrolyte, avoiding the continuous SEI growth problem caused by the high specific surface area in liquid batteries. In contrast, in liquid batteries, the porous electrodes experience rapid capacity degradation due to the continuous accumulation of SEI. In addition, the porous structure strengthened the electronic conduction network, enabling the electrode to maintain a higher capacity consistently in rate performance tests. High pore connectivity and low charge transfer resistance jointly promoted the kinetics of lithium-ion migration. The discharge capacity of the first cycle increased to 350 mAh g^−1^. After 40 cycles, the porous electrode still maintained a capacity of 300 mAh g^−1^. It is worth noting that the cycling stability of the porous structure in the solid system is superior to that in the liquid system. This is due to its high specific surface area, which avoids continuous electrolyte side reactions under solid interface conditions and only requires the formation of a stable interface layer at the electrode/electrolyte plane interface.

#### Composite Materials

##### Carbon Composite

**Fig. 6:**
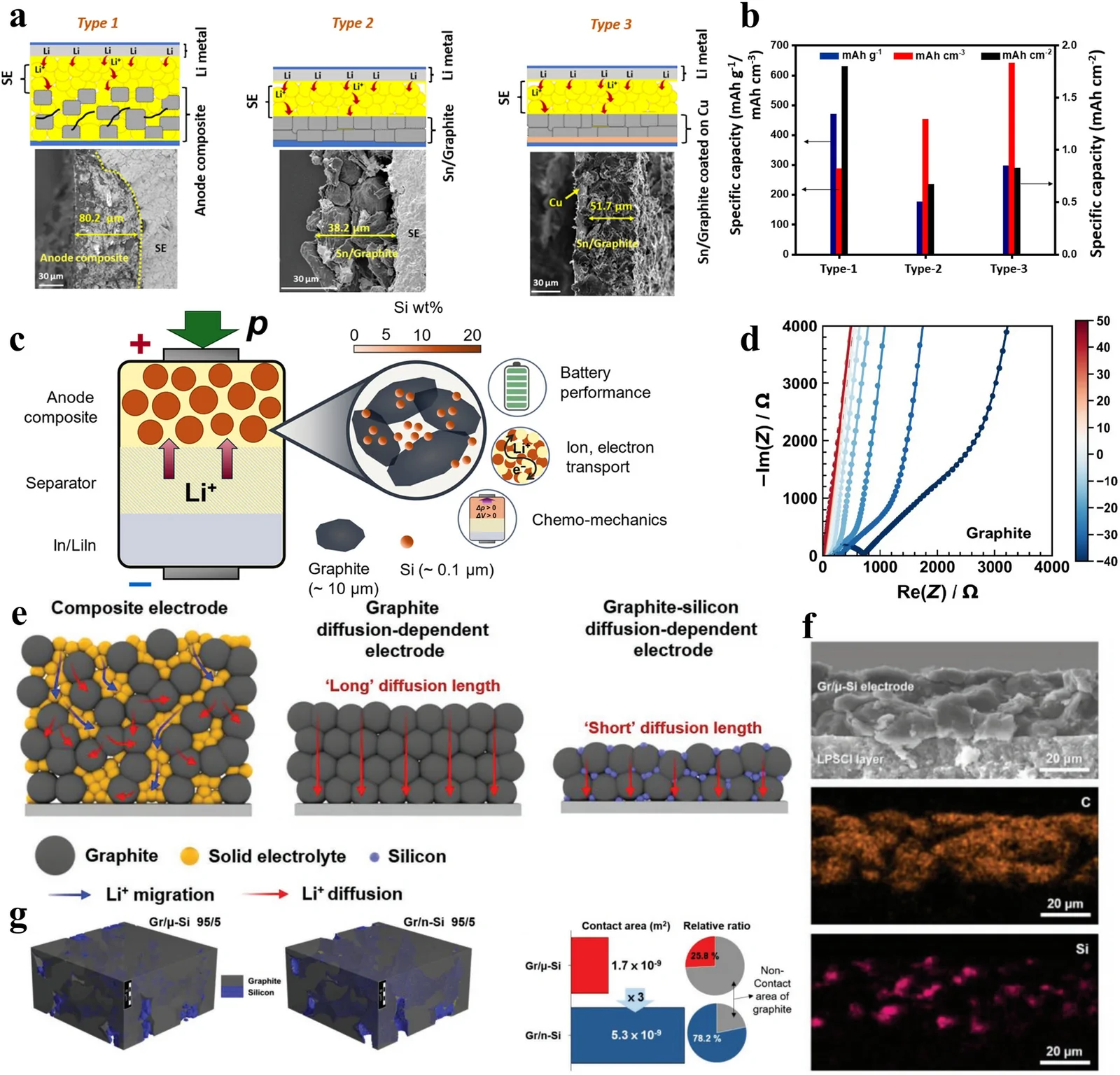
**a** Schematic diagrams of three different types of tin/graphite-based SSB electrodes under cyclic current and comparison of scanning electron microscope images. **b** A comparison of the weight capacity, volume capacity and area capacity provided by the three electrodes [147]. © 2022 The Authors. Published by American Chemical Society. **c** Schematic diagram of the half-cell structure of silicon/graphite composite material [148]. © 2025 The Authors. **d** The EIS curve of impedance spectra of graphite from − 40 to 50 °C. Published by American Chemical Society. **e** Schematic illustration of the structure and lithium-ion transport of the three different composite electrodes. **f** Cross-sectional SEM image and the corresponding EDS results of the Gr/µ-Si. **g** Digital twin-driven 3D structures, Contact area (m^2^) and Relative ratio of µ-Si and n-Si [149]. © 2021 Wiley–VCH GmbH

Carbon plays a multi-dimensional key role in alloy anode materials, such as silicon-based, tin-based, germanium-based, etc. Through the synergy of multiple mechanisms such as physical confinement, interface regulation, and structural stability, it has become the core material for solving the volume expansion and cycle failure of alloy anodes. Carbon materials construct a multi-level buffer system through gradient modulus design and stress dissipation topology [142]. When lithium-ion intercalation causes lattice expansion in alloy-type materials, the carbon layer coating the particle surface is the first to bear compressive stress, and the sp^2^ hybrid carbon bond undergoes bond angle distortion rather than fracture. The inner amorphous carbon absorbs deformation energy through the collapse of micropores and the sliding of carbon atoms, converting expansion potential energy into the internal frictional heat of the carbon layer [143, 144]. In addition, when carbon materials encapsulate alloy-type particles, the π electron cloud of carbon atoms forms p-π conjugation with the suspension bonds on the surface of the alloy materials, reducing the interfacial contact resistance. Importantly, the type and structure of carbon also have a significant impact on the performance of alloy-based/carbon composites. For instance, as the best commercialized anode material, graphite has the advantages of mature technology, low cost, good electrical conductivity, and easy processing [145, 146]. The layered structure of graphite has a certain degree of elasticity and can absorb some stress. In common alloy-based/C composites, nano-alloy-based particles are embedded in the pores of the graphite skeleton. The expansion squeezes the graphite layer, and the stress is shared by the entire graphite skeleton, thereby suppressing the macroscopic expansion of the electrode as a whole. Meanwhile, graphite itself is an excellent conductor, forming the base conductive network of the electrode. Palaniselvam et al. prepared tin-graphite composites (17 wt% nano-tin, 83 wt% graphite) as high-capacity anodes for all-solid-state lithium-ion batteries, demonstrating remarkable electrochemical properties (Fig. 6a) [147]. Electrochemical tests indicated that at a low current density of 0.02 mA cm^−2^, the electrode could release a specific capacity of 470 mAh g^−1^ (Fig. 6b). In addition, researchers found that the content of the solid electrolyte (SE) Li_3_PS_4_ in the electrode significantly affected the performance. The electrode containing 65 wt% SE achieved high utilization by optimizing the ion conduction path. However, a high SE ratio increased the weight and volume of the electrode and reduced the volumetric energy density. On the contrary, the electrode without SE had a higher volumetric capacity, reaching 642 mAh cm^−3^. However, the mass and area capacity significantly decreased, highlighting the problem of limited ion transport. Therefore, tin-graphite composites achieved a specific capacity close to the theoretical value through the synergistic effect of nano-tin and graphite, but their practical application still faced challenges, such as interface stability, electrode structure design, and kinetic optimization. In the actual design of solid-state batteries, it is necessary to control the silicon content to achieve the coordinated optimization of performance and mechanical stability. Pham et al. focused on exploring the influence mechanism of silicon content on electrochemical performance and chemomechanical behavior (Fig. 6c) [148]. When the silicon content was ≥ 10 wt%, the material could still maintain excellent discharge capacity at high rates (1 C), which is significantly better than that of pure graphite anodes. This is attributed to the high theoretical capacity and low working potential of silicon. However, the ICE of all composite materials was approximately 80%, mainly due to the decomposition reaction of the Li_5.5_PS_4.5_C_l1.5_ sulfide solid electrolyte at low potentials. In long-term cycling, an increase in silicon content can improve cycling stability, but the coulombic efficiency is slightly lower than that of pure graphite. In addition, due to the high electrical conductivity of graphite, the addition of silicon did not significantly change the transmission path (Fig. 6d). This indicates that the electrochemical differences of the composite materials mainly stem from the intrinsic properties of the active substances rather than the limitations of interfacial ion/electron transport. Although a silicon content of ≥ 20 wt% significantly increases capacity, the sharp increase in internal pressure may cause contact failure at the electrode/electrolyte interface, especially in full cells matched with high-expansion cathodes, the risk is even higher. Therefore, to achieve breakthroughs in silicon content and battery capacity, more innovative modification methods are still needed. In Kim's research, they proposed a novel design strategy for diffusion-dependent graphite-silicon composite electrodes [149]. Nanosilicon particles formed a lamellar structure between graphite particles, significantly enhancing the contact area of the two-phase interface (Fig. 6e, f). Three-dimensional digital twin simulation confirmed that the contact area increased by 200%, and the interface coverage rate rose from 25.8% to 78.2% (Fig. 6g). The structural design compressed the thickness of the silicon agglomeration region to the nanoscale, thereby successfully overcoming the defect of the intrinsic lithium-ion diffusion coefficient of silicon materials being too low. Synchrotron radiation and electrochemical impedance spectroscopy analysis further confirmed that an efficient cross-particle diffusion channel for lithium ions was formed in the composite electrode, achieving uniform transmission of lithium ions throughout the electrode interior and maintaining a capacity retention rate of 93.8% at a 0.5 C rate. This design ultimately demonstrated outstanding performance in the LiCoO_2_//graphite-silicon full cell, especially achieving stable cycling at room temperature and high-rate conditions.

**Fig. 7:**
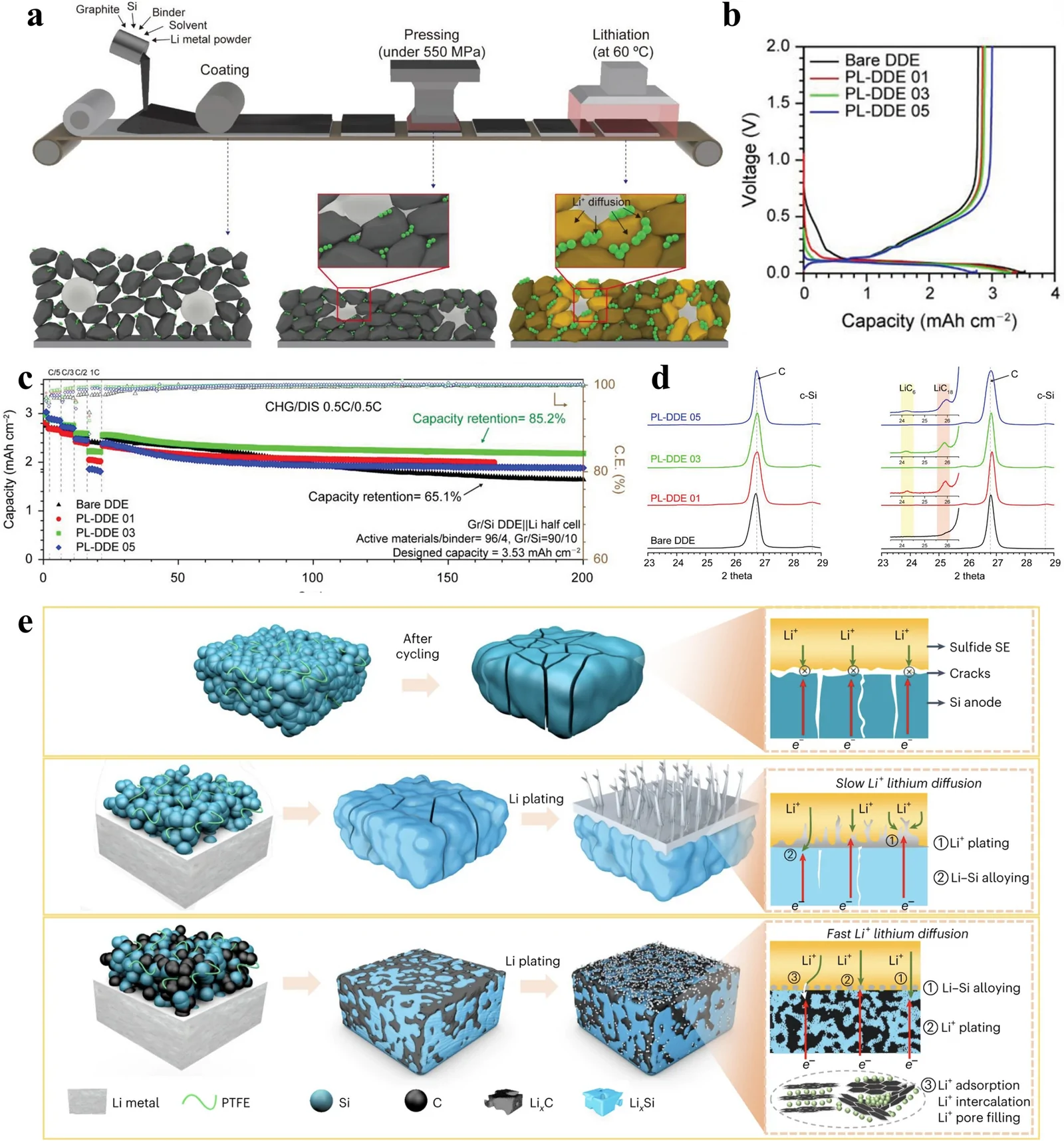
**a** Schematic diagram of the preparation process and lithiation process of PL-DDE. **b** First charge-discharge voltage profiles. **c** Rate capability and cycle performance of the Gr/Si||Li half-cells with DDEs. **d** XRD patterns of DDEs with different amounts of Li metal powder before and after pre-lithiation [152]. © 2023 The Authors. Advanced Energy Materials published by Wiley–VCH GmbH. **e** Schematic illustration of mechanisms for Si, LiSi and LiSH46 anodes in all-solid-state batteries [154]. © under exclusive licence to Springer Nature Limited 2023

The pre-lithiation of anode materials is a core and key technology to solve the problem of low initial efficiency of high-capacity anodes, especially silicon-based, tin-based and other alloy anodes [150, 151]. It provides an additional lithium source for the anode in advance before battery assembly to form an SEI film, thereby compensating for the initial lithium loss. Pre-lithiation promotes the formation of a dense and stable SEI film before the cycle begins. This high-quality initial SEI film can effectively prevent the continuous decomposition of the electrolyte and the repeated growth of the SEI film during the cycling process, thereby reducing the further consumption of active lithium in subsequent cycles. Lee et al. reported on the research based on the dry pre-lithiation technology of graphite-silicon composite anodes in all-solid-state batteries (Fig. 7a) [152]. The pre-lithiation of diffusion-dependent electrodes (Ddes) was achieved under dry conditions by introducing lithium metal powder (Fig. 7d). Specifically, lithium metal powder breaks the surface Li_2_CO_3_ coating layer under a high pressure of 550 MPa and a temperature rise of 60 °C , directly contacting graphite and silicon particles, whereas lithium-ion transmission was achieved through ion diffusion between particles. This process did not require solvents or additives, thus avoiding the possible side reactions that may be caused by traditional wet pre-lithiation, while ensuring the chemical stability of the electrode components. In addition, the residual lithium metal that was not fully reacted after pre-lithiation played a key role in the cycling process. It promoted the deep lithiation of active substances and enhances the kinetics of electrode reactions. It continuously compensated for the loss of active lithium caused by the formation of SEI and the expansion of silicon volume, thus increasing the initial efficiency of the half-cell from 75.9% to 108.9% and that of the full-cell from 73.3% to 84.8% (Fig. 7b). As shown in Fig. 7c, the half-cell exhibited excellent rate performance and long cycle performance. The capacity retention rate of the LiCoO_2_/ Li_3_InCl_6_/Gr/Si full battery was 88.1% after 100 cycles.

Apart from graphite, hard carbon is also one of the most core and commercially valuable anode materials at present, widely used in commercial sodium-ion batteries. The lithium storage mechanism of hard carbon is surface adsorption and short-range diffusion, with a short ion migration path and low resistance [153]. The interlayer intercalation of graphite requires long-range diffusion of ions and is prone to lithium precipitation under extremely large currents. In addition, its surface-driven reaction mechanism is less affected by temperature, and its disordered structure has a lower energy barrier for ion migration. Yan and team introduced a hard carbon-stabilized Li–Si alloy anode material for high-performance all-solid-state lithium-ion batteries (Fig. 7e) [154]. On the one hand, silicon particles undergo sintering effects during the first charge and discharge process, achieving the transformation from micron-sized particles to a dense continuous phase. On the other hand, hard carbon materials play a role as key stabilizing components. When the optimized mass ratio of Si to hard carbon was kept at 4:6, an ideal structure was formed. The plastic Li_15_Si_4_ phase and LiC_6_ phase effectively buffered the cyclic stress through the deformation mechanism. The three-dimensional framework of hard carbon significantly enhanced the lithium-ion conductivity due to its large interplanar spacing and special defect structure, and induced uniform deposition of lithium at the interface of hard carbon. When the anode system was coupled with the LiNi_0.8_Co_0.1_Mn_0.1_O_2_ cathode, it could withstand a high discharge current density of 155.3 mA cm^−2^ and achieved 5000 stable cycles with a capacity of 5.86 mAh cm^−2^, with a capacity retention rate of 61.5%. When matched with the LiCoO_2_ cathode, 30,000 cycles were completed at a current density of 14.64 mA cm^−2^ (20 C), with an initial capacity retention rate of 72.1%. It is notable that the battery assembled with a 20 mAh cm^−2^ high-load electrode achieved a device-level energy density of 263 Wh kg^−1^. Its performance advantage stemmed from the synergistic enhancement of electrode kinetics and structural integrity by the three-dimensional lithium-rich conductive network.

Carbon nanotubes can also serve as a multifunctional nanoscale additive and supporting framework, optimizing next-generation high-capacity and high-power anode materials by constructing a superconducting mechanically enhanced network [155]. When carbon nanotubes are introduced into battery anodes, they first intertwine to form a continuous, highly conductive, and porous network structure, greatly improving the conductivity of the electrode. This is crucial for active materials such as silicon and red phosphorus, which have poor conductivity themselves. More importantly, this three-dimensional network has excellent mechanical flexibility and strength [156, 157]. When faced with high-capacity materials such as silicon or tin that undergo significant volume expansion, the elastic skeleton composed of carbon nanotubes can absorb stress like a spring, buffering volume changes through its own bending and stretching, thus ensuring the structural integrity of the battery during long-cycle cycling. Hu's team innovatively developed a silicon/carbon nanotubes/carbon (Si/CNTs/C) composite anode material with a "reinforced concrete" microstructure [158]. Using ferrocene as a catalyst, the in situ reaction of magnesium-silicon alloy with CaCO_3_ simultaneously achieved the generation of silicon particles and the catalytic growth of carbon nanotubes (CNTs), formed a three-dimensional interpenetrating network structure. The tight physical contact constructed through in situ growth maintained stable interfacial contact between silicon active particles, CNT conductive network, and lithium sulfide electrolyte. The three-dimensional interconnected CNT network provided efficient electronic conduction paths while maintaining the integrity of lithium ion diffusion channels in the sulfide electrolyte. After cycling 50 times at a current density of 50 mA g^−1^, the composite anode still maintained a reversible specific capacity of 1226 mAh g^−1^. Even when increased to 200 mA g^−1^, it maintained a stable capacity of 395 mAh g^−1^ after 200 cycles. Structural characterization and electrochemical analysis jointly demonstrated that the CNT skeleton alleviated the significant volume effect and low conductivity issues of silicon-based anodes in sulfur-based solid-state batteries by inhibiting electrode cracking and interfacial degradation. The in situ growth of carbon nanotubes forms a continuous conductive network from the inside to the surface of the sample, which is more conducive to improving solid-solid contact interfaces and ionic conduction performance. The network structure of fibers provided continuous paths for electron and ion conduction, maintaining conductivity even in areas where some interfaces are detached, significantly improving the utilization rate of silicon. In addition, SEM observations revealed that silicon fibers underwent plastic deformation during cycling, forming amoeboid shapes and tight binding with the surrounding solid electrolyte, constructing an interconnected network structure that extended throughout the entire electrode. The study found that carbon nanotubes not only serve as "reinforcing steel" to tightly fix silicon active particles, but also maintain close contact with silicon particles, carbon layers, and Li_6_PS_5_Cl. This tight structural design precisely alleviates the volume expansion problem of silicon materials during cycling, while preventing the destruction of Li_6_PS_5_Cl as an ion channel. In terms of electrochemical performance, the synergistic effect of the high ionic conductivity of the sulfide solid electrolyte and the carbon nanotube network produces significant performance improvement.

The carbon nanofiber matrix not only provides excellent mechanical properties to release the stress generated by the volume change of silicon, but also constructs stable electronic conduction channels. In addition, Hafner et al. developed a novel synchronous electrospinning and electrospraying technology for directly manufacturing all-solid-state lithium-ion batteries in ambient air, solving the problems of high interfacial resistance, air and moisture sensitivity, and large-scale production in traditional manufacturing processes [159]. High-voltage electric fields are used to drive electrospinning to generate solid polymer electrolyte fibers and electrospray deposition of electrode active materials. This technology achieves direct stacking deposition of cathode, solid electrolyte, and anode for the first time, forming a dense, pore-free electrode structure, and enhances interfacial contact and electrode density through subsequent low-temperature pressurization (about 375 MPa). LiFePO_4_ was selected as the cathode material for lithium-ion batteries and Sn nanoparticles as the anode material in this system. Overall, this work demonstrated the potential of synchronous technology at the laboratory scale. However, the first cycle coulombic efficiency of the solid-state full battery was low (57.8%), and irreversible capacity loss and voltage anomalies were observed. SEM revealed voids in the electrolyte layer and changes in the electrode structure after cycling, indicating that interfacial degradation was a performance-limiting factor. Similarly, Maroni and team used a mixed polymer solution of polyacrylonitrile (PAN) and polymethylmethacrylate (PMMA), combined with tin nanoparticles, to prepare a composite film composed of micro/nanofibers [160]. The mixture was first thermally treated at a specific temperature in air, followed by high-temperature annealing and carbonization in an argon/hydrogen mixed atmosphere. During the high-temperature treatment process, the polymer was carbonized to form a conductive porous carbon fiber matrix, where the tin nanoparticles were found to be uniformly dispersed within it. This porous structure benefited from the decomposition of PMMA into gas emissions during the first step of treatment. When this material was applied to all-solid-state lithium batteries composed of sulfide-based solid electrolytes, it exhibits significant initial capacity, reasonable cycle stability, and good interfacial stability.

**Fig. 8:**
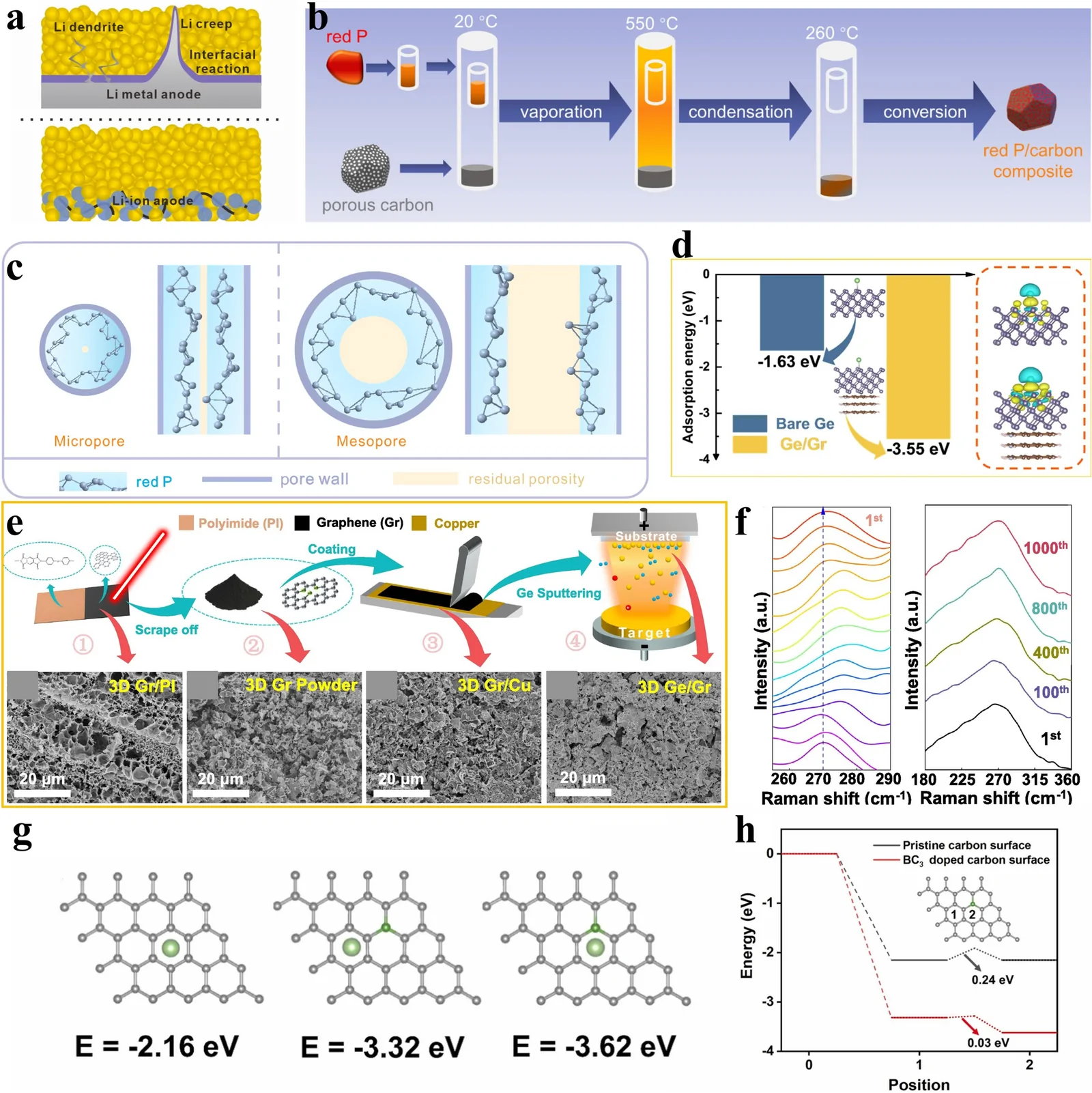
**a** Schematic of Li metal and Li-ion anodes in all-solid-state batteries [163]. © 2024 Elsevier B.V. All rights reserved. **b** Synthesis schematic of red P/carbon composite. **c** Schematic of red P deposition inside the micropore and mesopore [55]. © 2024 American Chemical Society. **d** Adsorption energy and differential charge density in the bare Ge and Ge/Gr surfaces. **e** Schematic of the preparation process of the 3D Ge/Gr electrode and corresponding SEM images. **f** in situ Raman pattern and ex-situ Raman pattern of 3D Ge/Gr anode [165]. © 2025 Elsevier Inc. All rights are reserved. **g** Adsorption energy of different models; and **h** the adsorption energy and diffusion energy barrier [167]. © 2024 Elsevier B.V. All rights reserved

Compared with tin nanoparticles embedded in carbon fibers, activated carbon as a high-performance host or carrier material is currently a more concerned research and application direction. Porous carbon plays a crucial role in the alloy-based anode of solid-state batteries [161]. The pores in carbon materials provide valuable space for the volume expansion of alloy-type materials, greatly suppressing macroscopic volume changes and preventing structural collapse. Defects at the edges and surfaces of the channels or introduced heteroatoms (such as N, O, S) can provide active sites to store more lithium ions through the Faraday reaction (pseudocapacitance). When combined with red phosphorus, several improvements can be observed, especially when the insulating properties and significant volume expansion of red phosphorus limit its practical application [162]. Activated carbon constructs a three-dimensional high-speed electronic conduction network for phosphorus-based materials with poor inherent electrical conductivity, improving the rate performance. By confining phosphorus nanoparticles in pores or encapsulating them within carbon layers to prevent their agglomeration during circulation. Compared with the lithium dendrites that are prone to form during the cycling process of metal Li anodes (Fig. 8a), red phosphorus is an anode material with greater development potential. The Sun team effectively activated the electrical insulation of red phosphorus by loading sub-nanoscale red phosphorus clusters into the micropores of commercial activated carbon (AC) (forming a P@AC composite material), achieving its efficient operation in room-temperature ASSB in half-cell tests [163]. The P@AC electrode containing 53 wt% red phosphorusprovided a high initial lithiation capacity of up to 1438 mAh g^−1^ at a current density of 0.2 A g^−1^, and maintained a capacity retention rate of 66.9% after 400 cycles. Through symmetrical battery tests, the P@AC anode demonstrated an ultra-high critical current density exceeding 18 mA cm^−2^, with no risk of lithium dendrite formation, significantly superior to metallic lithium anodes. In the full battery configuration, the initial surface capacity reached 2.98 mAh cm^−2^. It could still operate stably after 200 cycles and had a practical surface capacity retention rate under high loads. Compared with the P@CNT composite material formed by depositing red phosphorus on the surface of carbon nanotubes (CNT), P@AC provided a closer electronic contact interface due to the micropore loading strategy, significantly reducing electrode polarization and accelerating the lithium-ion diffusion kinetics.

The huge specific surface area and well-developed pore system of carbon matrix makes it an ideal platform for loading other active substances. It is used for highly dispersive and fixing nanoscale active material particles, using its pores to buffer volume expansion and its conductive network to enhance the overall electrical conductivity. By precisely controlling the size, distribution, and surface chemistry of the microporous structure and its interaction with red phosphorus, its comprehensive electrochemical performance can be further enhanced. To address this issue, Wang et al. employed a vapor deposition-condensation strategy to encapsulate red phosphorus in the nanoporous pores of the carbon matrix, thereby activating its electrochemical reaction interface and suppressing volume changes (Fig. 8b) [55]. The core role of the microporous structure of the carbon matrix in optimizing the red phosphorus loading was confirmed by comparing two model carbon matrices with clear pore structures. Zeolite template carbon (ZTC) mainly contained monodisperse micropores with an average pore size of approximately 0.93 nm, whereas the ordered mesoporous carbon (CMK-3) mainly contained uniform mesoporous structure, with an average pore size of approximately 4.91 nm. The micropores of ZTC were significantly superior to the mesoporous pores of CMK-3 in terms of pore utilization. The loading rate of red phosphorus in ZTC was as high as 65.0 wt%, while it was only 46.2 wt% in CMK-3. This difference mainly stemmed from the higher adsorption potential energy within the micropores, which enhanced the interaction between red phosphorus molecules and the carbon pore walls, thereby improving the filling efficiency (Fig. 8c). Furthermore, residual pore analysis revealed that the surface area of the P@ZTC complex after loading red phosphorus dropped sharply to almost zero, approximately 2 m^2^ g^−1^, while P@CMK-3 still retained a relatively high surface area, approximating to ~ 98 m^2^ g^−1^. This difference in surface area had a crucial impact on the electrochemical performance. In the electrochemical performance test, the anode of P@ZTC reached a capacity of 1823 mAh g^−1^ at the first charge of the half-cell, and the ICE was 87.44%, which was much higher than 1200 mAh g^−1^ and 73.80% efficiency of P@CMK-3. More notably, P@ZTC demonstrates outstanding long-cycle stability. After 400 cycles at A current density of 0.2 A g^−1^, the reversible capacity remained at 1260 mAh g^−1^, corresponding to a capacity retention rate of 99.92% per cycle. The full battery composed of P@ZTC and NCM811 cathode maintained a capacity of 67.26% after 1000 cycles at 0.5 C. Therefore, micropores, through high porosity utilization and low residual surface area, were found to enhance the utilization of active materials, suppress interfacial side reactions, and ultimately achieve high-capacity and long-life anodes.

The application research of graphene in the anode of solid-state lithium-ion batteries demonstrates its immense potential as well. Its value stems from its intrinsic two-dimensional sp^2^ hybridized carbon lattice structure, which endows it with excellent electron mobility, extremely high theoretical specific surface area, superior mechanical strength, and flexibility [164]. In the anode system, the function of graphene far exceeds that of traditional conductive additives. By constructing a multi-dimensional functionalized structure, it achieves synergistic enhancement of the electrochemical performance of electrode materials. Its flexible layered structure can absorb stress through its elastic deformation, inhibiting the pulverization of active particles. Through in situ composite or self-assembly strategies, graphene nanosheets can be uniformly coated on the surface of active nanoparticles, forming a stable core–shell structure. This encapsulation not only provides a continuous electron transport path but also promotes the formation of a more stable and denser solid-state electrolyte interfacial film. Fang et al. utilized laser-induced graphene (Gr) to enhance the physical and electrochemical properties of germanium (Ge) anodes in lithium-ion batteries (Fig. 8d, e). By laser ablation of polyimide (PI) substrates, a 3D interconnected graphene skeleton with a high specific surface area (approximately 169.0 m^2^ g^−1^) and microporous structure (pore size approximately 4.3 nm) was prepared [165]. Subsequently, an amorphous germanium thin film was deposited on its surface using radio frequency magnetron sputtering technology. A series of in situ and ex situ characterizations showed that this 3D structural design not only provided ample space to buffer the volume changes of the germanium electrode during lithiation/delithiation, but also significantly shortened the transport distance of electrons and ions, thereby optimizing the electrochemical kinetic performance (Fig. 8f). The 3D Ge/Gr composite structure exhibited higher lithium adsorption energy and improved charge density distribution, indicating that the graphene support layer effectively promotes charge transfer and electrochemical reactions. An all-solid-state Ge/Gr||Li battery was also constructed using polyvinylidene fluoride-hexafluoropropylene/Li_1.3_Al_0.3_Ti_1.7_(PO_4_)_3_ (PVDF-HFP/LATP) solid polymer electrolyte. The results showed that under the solid-solid interface, the 3D Ge/Gr electrode maintained efficient electrochemical behavior. It provided a discharge capacity of approximately 1010 mAh g^−1^ at a high current of 5.0 A g^−1^ and achieved excellent rate reversibility.

More importantly, the non-metal sites in the non-metal-doped carbon framework can serve as active sites for lithium ion adsorption [166]. The lone pair electrons of non-metal atoms form weak coordination bonds with Li^+^, enabling directional tunneling transmission of Li^+^ driven by an electric field and enhancing the lithium ion diffusion coefficient at the interface. This mechanism intertwines electron and ion conduction paths at the nanoscale, allowing electrons to migrate rapidly along the graphene lattice while Li^+^ undergoes hopping transmission at carbon layer defects. For example, Zhang et al. constructed a boron-doped carbon shell in situ on the surface of silicon nanoparticles using a sol–gel method and high-temperature annealing process, and then loaded it onto a reduced graphene oxide (rGO) substrate to form a core–shell-carrier composite structure [167]. Boron atoms were doped into the carbon layer in the form of B-C bonds, where their atomic radius (0.082 nm) was larger than that of carbon (0.077 nm), thereby widening the spacing between carbon layers. At the same time, boron due to a stronger affinity for lithium ions, reduced the diffusion energy barrier of lithium ions from 0.24 to 0.03 eV, thereby significantly promoting lithium ion insertion/extraction kinetics (Fig. 8g, h). The carbon shell effectively inhibited the volume expansion of silicon particles during cycling and enhanced the initial coulombic efficiency. rGO provided a three-dimensional conductive network and mechanical support, thus inhibiting particle agglomeration, and its layered structure reserved buffer space for silicon volume changes. It is particularly important to state that this design still performed well in all-solid-state battery systems, with a stable capacity of 956 mAh g^−1^ after 100 cycles when using sulfide solid-state electrolytes, hence demonstrating the stability of the solid-solid interface. In short, the boron-doped carbon shell enhanced conductivity and lithium ion migration ability, while the rGO carrier optimized charge transmission and mechanical stability. The triple synergistic effect provided innovative ideas and technical paths for the development of practical silicon-based anodes.

##### Metal Composite

In addition to the widely studied strategies of compounding with carbon materials, compounding with metals or metal matrices does indeed constitute another important and promising technological path. By using the metal matrix as a robust and highly conductive continuous phase, this design provides strong mechanical support and an efficient electronic transmission network for alloy active substances. Unlike carbon materials, which mainly respond to volume changes through elastic buffering and physical confinement, the metal matrix composite strategy focuses more on taking advantage of the excellent ductility, strength and low irreversible capacity of metals themselves to construct a composite structure that combines rigidity and flexibility [168, 169]. This metal matrix plays multiple key roles. As a continuous skeleton, it can effectively restrain the expansion and contraction of active particles during the lithiation/delithiation process, prevent them from falling off the current collector, and thus maintain the structural integrity of the electrode [170]. Secondly, the inherent high electronic conductivity of metals ensures efficient charge transfer within the electrode, significantly improving the rate performance of the material [171].

Active metals can undergo alloying reactions with lithium at appropriate potentials, but this alloying behavior is usually accompanied by relatively mild volume changes and good reversibility. This, together with the alloy-type material, constructs a composite system with coordinated energy storage and adaptive stability functions. In addition, the introduction of active metals helps to optimize the kinetic process of electrode reactions. Their inherent high electronic conductivity ensures efficient charge transfer, and the alloying reaction of certain metals may have a certain pseudo capacitive behavior, which helps to improve rate performance. For example, Whiteley et al. constructed a Si-SN composite anode material by embedding high-capacity Si into a Sn matrix with good ductility [172]. The design took advantage of the characteristic of Sn, where it preferentially lithiates at a voltage higher than that of Si. The volume expansion generated during the lithiation process exerted in situ and uniform hydrostatic pressure on adjacent Si particles. The isotropic stress field significantly improved the reversibility of Si during the cyclic process and suppressed its inherent huge volume expansion. The composite electrode achieved a specific capacity of up to 1000 mAh g^−1^ in solid-state battery systems, and still stably provided a reversible capacity of 700 mAh g^−1^ after 50 cycles, which was far superior to pure Sn anodes. It is worth noting that under high-pressure stress conditions, Si abnormally forms the high-temperature stable phase Li_7_Si_3_. This phenomenon is attributed to the strain energy provided by Sn expansion, which reduces the crystal nucleation barrier and promotes the formation of thermodynamically metastable crystalline phases, replacing the traditional amorphous Li_x_Si alloy. Compared with the unidirectional mechanical compression strategy, the in situ uniform stress provided by the Sn matrix more effectively maintains the structural integrity of Si, thereby significantly enhancing the cycling stability. This study not only verified the synergistic enhancement mechanism of Sn as an active matrix with both conductivity and buffering functions on Si.

In addition, these characteristics of semiconductor electrodes significantly affect the evaluation of battery characteristics. Firstly, dynamic changes in contact resistance and bulk resistance during charging introduce additional internal resistance fluctuations, such as barrier manifestation caused by conductivity type transition and resistance peaks induced by doping compensation effect. Secondly, in impedance measurements, contact nonlinearity and voltage sensitivity effects distort the parameter extraction of traditional equivalent circuit models, leading to an overestimation of electrode/interface impedance. These effects collectively contribute to uncertainty in battery state of charge (SoC) evaluation, posing new challenges for accurate modeling of semiconductor-based SSLIB. Therefore, when developing new semiconductor electrode systems, it is necessary to simultaneously consider the influence mechanisms of contact physics and material nonlinearity to optimize battery design and state monitoring strategies. Rudy's research group found that the anode composed of silicon-aluminum nanocomposite material (Si@O@Al) forms a characteristic voltage step phenomenon on the charging curve during charging when lithium ions are inserted, causing the material's conductivity type to transition from hole-dominated to electron-dominated [173]. The physical mechanism of this phenomenon stems from the role of the aluminum element as an acceptor impurity in the material composition. In the unlithiated state, aluminum doping renders the silicon matrix P-type semiconductor characteristics, forming an ohmic contact with the titanium current collector. As the degree of lithiation increases, the material gradually transitions to an electron-dominated N-type semiconductor, forming a Schottky barrier at the metal–semiconductor interface. Additional voltage is required to maintain constant current charging, resulting in the experimentally observed voltage transition. The experimental value of barrier height is lower than theoretical predictions, which can be attributed to factors such as the Schottky effect caused by image force, interface state influence, and interface double layer.

**Fig. 9:**
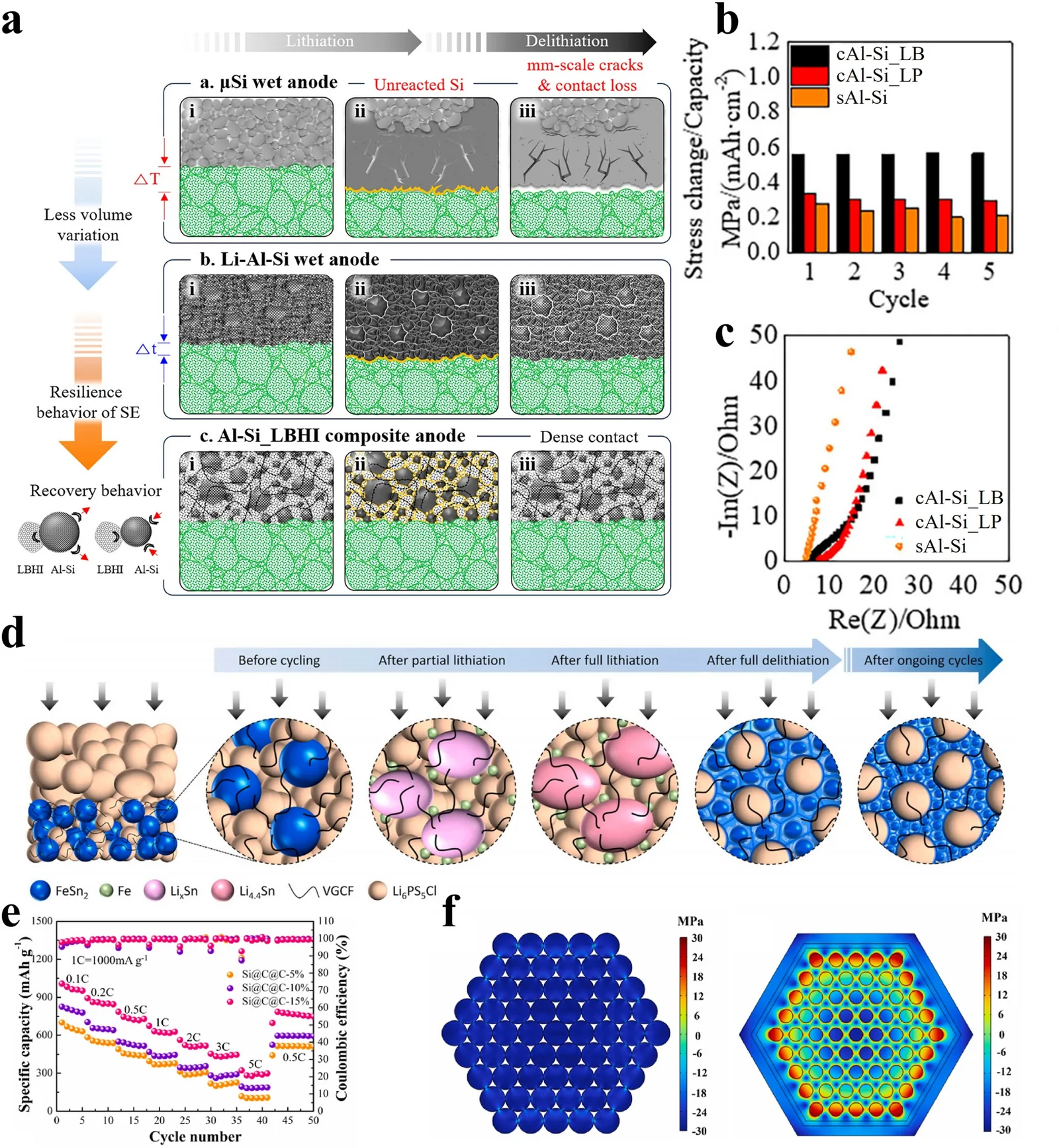
**a** Schematic diagram of the structural evolution of aluminum–silicon-lithium-boron hydride composite anode (Al-Si_LBHI) and its comparative samples. **b** Schematic diagram of internal stress distribution and **c** the EIS in Al-Si anode full cell [176]. © 2025 Advanced Functional Materials published by Wiley–VCH GmbH. **d** Schematic diagram of morphological changes in Sn anode during cycling in ASSLIBs [2]. © 2024 Elsevier Inc. **e** Rate capability performance of Si@C@C-5%, Si@C@C-10% and Si@C@C-15% electrodes. **f** Stress distributions of the models of pure Si NP and Si@C@C-10% composites [110]. © 2024 Elsevier Ltd

Theoretical calculations play an indispensable role in guiding and analyzing the research on solid-state lithium-ion battery anodes. The impact extends across multiple levels, from atomic-scale electronic structures to mesoscopic-scale phase field evolution, significantly complementing and surpassing the limitations of traditional experimental methods [174]. By utilizing multiscale computational approaches such as first-principles calculations based on density functional theory (DFT), molecular dynamics (MD) simulations, phase field methods, and machine learning potential functions, it is possible to reveal the complex ion transport mechanisms, the essence of interface stability, and the dynamic processes of microstructural evolution in solid-state systems from a physical perspective. Their value is firstly reflected in the precise prediction of intrinsic physical properties and the elucidation of mechanisms. First-principles calculations can accurately calculate the electronic structure, thermodynamic stability, lithium-ion migration barriers, and intrinsic bulk conductivity of anode materials and solid electrolytes, providing a theoretical basis for the screening and design of high-performance materials. Bhimineni and team explained the mechanism of aluminum doping on the cycling stability of polycrystalline silicon anode materials in lithium-ion batteries through a combination of first-principles calculations and experimental verification [175]. Theoretical calculations showed that the segregation of trace aluminum elements at silicon grain boundaries (GBs) can significantly reduce the energy barrier of grain boundary sliding. Density functional theory simulations revealed that the displacement energy of aluminum at grain boundaries is 0.9 eV, which is lower than its 0.99 eV in the silicon bulk phase. Aluminum tends to accumulate in the grain boundary region and form weaker Al-Si bonds than Si–Si bonds, thus acting as a "bonding lubricant". When the amount of aluminum doping is 4 atoms per grain boundary, the energy barrier of grain boundary sliding can be reduced to 0.0385 eV Å^−2^, which is only 12% of the pure silicon system. This reduction allows the material's volume deformation stress during lithiation/delithiation to be effectively released through grain boundary sliding, avoiding material cracking caused by stress accumulation. The study also found that there is a concentration threshold for the optimization effect of aluminum doping. When the aluminum content is > 3 atoms/grain boundary, the energy barrier decreases slowly, with an increase of only 0.3%, but the grain boundary formation energy continues to increase. This explains why optimal performance can be achieved with only 5 wt% trace doping in experiments. In addition, the study revealed the synergistic effect of oxygen elements. The oxygen introduced during the ball milling process promotes stable segregation of aluminum at grain boundaries. However, the first cycle discharge reaction reduces oxides to metallic aluminum and Li_2_O, ultimately leading to the metastable distribution of aluminum at grain boundaries. This work provides a new strategy for developing high-stability silicon-based anodes through the linkage verification of atomic-scale theoretical models and macroscopic electrochemical properties. Recently, Song et al. first achieved the application of Al-Si alloy in sulfide batteries, focusing on the synergistic mechanism between silicon and electrolyte to achieve contact loss suppression [176]. They proposed and verified a composite anode architecture combining aluminum–silicon (Al-Si) alloy anode material with elastically recoverable borohydride-based electrolyte (3LiBH_4_-LiI, LBHI), revealing the electrochemical-mechanical coupling relationship between material microstructure and interfacial behavior (Fig. 9a). The unique dual-phase structure of Al-Si alloy effectively adjusts volume expansion behavior by embedding a silicon skeleton in the tough aluminum matrix. After the first cycle of lithiation, the volume expansion quickly saturates, and only minor deformation occurs during delithiation, significantly different from pure silicon (Fig. 9b). The silicon skeleton forms a stable network after the first expansion fracture, while the aluminum matrix maintains electrode integrity through plastic deformation. The two cooperate to suppress the volume change to 37.5%. Pre-lithiation treatment increases the coulombic efficiency of the material to 84.7% in the first cycle, and reduces the cycle expansion rate by 93%, laying the foundation for practical application. And using the "elastic recovery" characteristic of LBHI electrolyte, it forms a sharp contrast with the plastic deformation of Li_6_PS_5_Cl sulfide electrolyte. Its three-dimensional network structure can adapt to the volume expansion and contraction during charging and discharging, maintain the close contact of Al-Si particles, and reduce impedance (Fig. 9c). The multi-level collaborative mechanism of aluminum matrix dissipating silicon expansion stress at the microscale, elastically recovering electrolyte constructing adaptive interface at the mesoscale, and pre-lithiation fixing volume framework at the macroscale provides a material-structure-process trinity solution for high energy density all-solid-state batteries.

The successful application of Al-Si anode solid-state batteries has broken through the limitations of a single alloy type material. The composite design not only does not rely on nanostructures, avoiding the problem of preparation complexity, but also significantly improves capacity stability through the buffering effect of solid electrolytes. This provides a new path for developing high-capacity and high-safety solid-state lithium batteries, such as expanding to other alloy anode materials, like SiGe. The Loaiza^,^s team successfully synthesized a solid solution silicon–germanium alloy (Si_0.5_Ge_0.5_) with a face-centered cubic structure through mechanical ball milling [177]. Its unit cell parameter (a = 5.54 Å) lies between that of pure silicon (5.43 Å) and pure germanium (5.64 Å), conforming to Vegard's law and confirming the formation of solid solutions. During the charging process, the crystalline phase Li_15_(Si_0.5_Ge_0.5_)_4_ gradually decrystallizes into a-Li_x_ (Si/Ge), and the final delithiation product is an amorphous SiGe alloy. The important finding is that both XAS (X-ray Absorption Spectroscopy) analysis and Raman spectroscopy confirmed that silicon and germanium elements remained atomically uniformly mixed during the cycling process, without phase separation or element segregation. During discharge, the Ge–Ge bond breaks preferentially (compared to the Si–Ge bond), but synergistic lithium storage is achieved by forming the ternary lithium compound Li_15_(Si_0.5_Ge_0.5_)_4_ and reconstructing the Si–Ge bonding network during charging. Uniform Si–Ge chemical bonding is maintained throughout the charge-discharge cycle, rather than stepwise formation of binary lithiates (Li_x_Si/Li_x_Ge). This unique structural reversibility is the essential reason why the Si_0.5_Ge_0.5_ alloy has better cycling stability compared to monomer silicon/germanium or their physical mixtures.

In addition, the multiphase alloy strategy provides key support for the practical design of solid-state batteries by offering reversible self-healing mechanisms and enhanced ion/electron conduction networks. However, there are very few reports on the application of solid alloy anodes. Until recently, Kim et al. introduced the Ga-In-Sn liquid metal alloy (GIS-LMA), which penetrates into the pores of μSi anodes through its liquid property at room temperature [178]. During the lithiation process, this alloy reacts with Li to solidify into alloys such as Li_2_Ga, Li_2_In and LiSn, forming a robust ion and electron conduction network. It is particularly worth noting that GIS-LMA exhibits reversible solid-liquid phase changes during the cycling process. It transitions from liquid to solid during lithiation and returns to liquid during delithiation. This phase transformation behavior endows the μSi anode with self-healing capability. The liquid alloy can dynamically fill the microcracks and pores generated during the cycle, thereby maintaining continuous particle contact and low-resistance pathways. The experimental results show that the μSi anode doped with GIS-LMA (μSi/GIS-LMA) has a conductivity increase of 90,000 times compared with the pure μSi anode, and significantly reduces the internal resistance by reducing the stress caused by volume expansion. The improvement is attributed to the fact that GIS-LMA optimizes the conductive network and alleviates the structural deformation of the electrode. In both half-cell and full-cell configurations, μSi/GIS-LMA anodes exhibit excellent electrochemical performance. For instance, at high current density (1C), LiNi_0.8_Co_0.1_Mn_0.1_O_2_ (NCM) /μSi/GIS-LMA full cells achieved stable cycling and high coulombic efficiency, while a 28.9% reduction in electrode swelling was observed. It is worth noting that although GIS-LMA can solve the cumulative problem of interface contact loss caused by volume expansion, the usage needs to be further optimized to help overcome cost limitations and promote industrial applications.

Furthermore, metals such as Fe and Cu can form miscible solid solutions or intermetallic compounds with active substances like Sn and Sb, enhancing the interfacial bonding force through strong metallic bonds, which is more stable than physical contact [179]. Tout et al. evaluated the performance of alloyed type anode material Ni_3.4_Sn_4_ in all-solid-state lithium-ion batteries based on mercury chlorite Li_6_PS_5_Cl electrolyte [180]. The lithium intercalation capacity in the first week is 170 mAh g^−1^, and the reversible capacity is 120 mAh g^−1^, but it has excellent cycling stability. Its smaller volume change and lower carbon content reduce the side reactions at the electrolyte interface, thus maintaining its structural integrity. However, the core reason for the performance limitations of solid-state batteries lies in the electrode structure design level: (1) The agglomeration of active substances forms isolated regions, hindering the ion/electron transport network; (2) The plasticity of the electrolyte is insufficient to buffer the drastic volume changes of high-capacity materials, resulting in contact failure. (3) An excessive proportion of carbon additives accelerate the decomposition of the electrolyte interface.

Tin anodes are prone to particle agglomeration and cracking during high-current cycling, which is attributed to their high ductility and low elastic recovery ability, thereby disrupting the interface stability with solid electrolytes. Lee and team innovatively introduced iron-tin compounds (FeSn_2_) as self-stabilizing anode materials. The mechanism lies in the unique electrochemical cycle-induced size reduction mechanism and excellent mechanical properties (Fig. 9d) [2]. During the charging/discharging process, FeSn_2_ generates nanoscale iron and lithium-tin interphases through conversion reactions. Among them, iron particles effectively inhibit the agglomeration of tin and, through cycling, reduce the grain size of FeSn_2_ to below 5 nm, forming a uniform and dense structure. In addition, the moderate ductility and brittleness ratio of FeSn_2_ and its significant elastic–plastic deformation energy storage capacity enable it to resist deformation and avoid interfacial contact loss in high heap pressure environments. The results show that the FeSn_2_ anode exhibits high reversible capacity, excellent cycling stability, and wide temperature range adaptability in the half-cell configuration. In the Li_6_PS_5_Cl solid electrolyte full battery system, this anode achieves high surface capacity and high rate performance, while the energy density remains significant at ultra-high current density. The performance is attributed to the self-stabilizing mechanism of the FeSn_2_ anode, which effectively alleviates the volume change problem and enhances the interfacial ion/electron conduction.

To address the polarization effect and lithium deposition issues of traditional graphite anodes during high current charging, Lu et al. constructed an intermetallic compound Cu_6_Sn_5_ network as an efficient solid-state lithium ion transport channel [181]. They designed a copper tin alloy nanowire (Cu@CuSn). The network structure consisted of copper nanowires (Cu NWs) as the core, and a surface coating containing a lithium inert layer Cu_3_Sn and a lithium active layer Cu_6_Sn_5_. The crystal structure of Cu_6_Sn_5_ underwent lithiation and transformed into the Li_x_Cu_6_Sn_5_ phase, thus exhibiting superior high-speed lithium transport properties. Through defect structure analysis, it was found that tin atoms migrated during lithiation to form a zinc mineral symmetric structure. The open channel of this structure could accommodate rapid migration of lithium ions, and its dynamic process exhibited capacitance-like behavior, thereby achieving non-diffusion-controlled rapid lithium insertion. The experiment showed that the diffusion coefficient of the Li_x_Cu_6_Sn_5_ phase was significantly improved, and its effective diffusion coefficient was verified to be twice that of traditional copper nanowires through polarization and interrupted diffusion tests. The high load 2 wt% Cu@CuSn electrode design of NWs ensured uniform dispersion of the network through ball milling technology, forming a three-dimensional electron/ion conduction pathway across the entire electrode range. In the full battery test, the G-SLTC electrode achieved a capacity retention rate of 96.6% (145 mAh g^−1^) at a high-speed charging rate of 6 °C , which is much better than the 78% of traditional graphite anodes. Through in situ XPS mapping and expansion monitoring, it was shown that lithium ions were evenly distributed across the electrode cross-section, avoiding interface failure. At the same time, the energy utilization rate remained at 95% after 100 cycles, reflecting a significant improvement in electrochemical stability.

In addition, carefully selected metal substrates such as Ni and Mo exhibit negligible alloying reactions with lithium within their working potential window, where they act as electrochemical inert phases that help reduce the irreversible capacity of the entire electrode and improve initial coulombic efficiency. The Yersak team reported an innovative research on an all-solid-state lithium-ion battery using pre-lithiated silicon titanium nickel (Si Ti Ni, STN) alloy anode [182]. After the first electrochemical activation, the alloy formed the mixed conductive matrix of Li_3.2_Ti_4_Ni_4_Si_7_, that had an ion conductivity of 2.0 × 10^−5^ S cm^−1^ and an electronic conductivity of 0.24 S cm^−1^. This unique mixed conductive characteristic significantly increased the loading capacity of silicon materials in solid-state electrodes. It is worth noting that the team had for the first time applied the pre-lithiation technology of stabilized lithium metal powder (SLMP) to an all-solid-state battery system, successfully solving the matching problem between lithium-free cathode (FeS + S composite cathode) and lithium-free anode. The final FeS + S//pre lithiated STN all-solid-state battery exhibited excellent cycling stability at 60 °C : the first cycle discharge specific capacity reached 295 mAh g^−1^ (based on the cathode), and after 100 cycles, it still maintained a capacity of 355 mAh g^−1^. More importantly, the battery had an energy density of 225 mWh g^−1^ calculated based on the total mass of the dual electrodes, breaking through the traditional solid-state battery's dependence on lithium metal anodes and providing a new technological path to avoid the risk of dendrite penetration.

By precisely regulating the deposition kinetics and electrochemical activation window, a new type of anode with high mechanical stability and tunable electrochemical performance can be designed for miniaturized/solid-state energy storage devices, which is another innovative optimization strategy. In addition, the active material can be deposited on the substrate surface through ion-assisted grazing angle electron beam co-evaporation technology to achieve multiphase coexistence [183]. The high-density interface layer formed by ion-assisted deposition ensures stable contact between the electrode and the current collector. The interface inclined structure induced by argon ion bombardment further enhances the adhesion strength. Karahan et al. designed and studied a unique three-dimensional helical nanostructured silicon–molybdenum-oxygen (Si–Mo–O) composite system through this technology [184]. This method can achieve the co-deposition of molybdenum, silicon, and oxygen atoms, and form a composite structure containing various metal silicides and silicon/molybdenum oxidation states. The silicon phase reaction was mainly activated in the 0.2–1.2 V window to achieve good cycling stability. In the 0.2–3.0 V window, molybdenum oxide is simultaneously excited to participate in the transformation reaction, increasing the initial capacity but causing interfacial kinetic lag. The 5 mV–3.0 V window achieves a balance between high capacity and rate characteristics by deeply lithiating the silicon phase and maintaining a wide potential range. The phenomenon of "voltage-activated component selection" stems from the lithiation differences of various components in the electrode at specific potentials. The high specific surface area structure in the material significantly shortens the lithium-ion diffusion path, while the voids between the helices effectively buffer the stress and strain of the electrode during the lithiation/delithiation process. After cycling 200 times in a wide voltage window at a current density of 5 μA cm^−2^, the electrode demonstrated an excellent capacity retention rate. It is worth noting that this structure still maintained stable cycling capability at a high rate as high as 0.7 mA cm^−2^, which has a practical significance for the application of micro-solid-state batteries. The mechanism can be attributed to the fact that at a lower depth of discharge, lithium ions can rapidly diffuse through a high specific surface area contact. Silicon active particles participate in lithiation to provide high capacity, while inert molybdenum atoms act as conductive skeletons to maintain structural integrity. Meanwhile, copper-doped particles enhance the overall conductivity of the electrode.

As one of the products in the first lithiation process of alloy-type anode materials, Li_2_O captures active lithium ions, causing severe irreversible capacity loss. However, due to the in situ formation of Li_2_O nanoparticles, they can serve as a microstructure buffer phase. They possess a certain mechanical strength and can, to a certain extent, disperse and suppress the volumetric strain of active substances during cycling, which is conducive to maintaining the structural integrity of the electrode. In addition, the decomposition products of Li_2_O, especially during interfacial reaction process, have a significant impact on the microstructure and interfacial chemistry of the electrode. The lithium and oxygen species produced by decomposition may participate in the composition of the interfacial film, which helps to form an inorganic-enriched SEI layer that is denser, more stable and has a higher ionic conductivity. Jee and team fabricated such SLC composite films with nanocrystalline tin particles embedded in an amorphous Li_2_O matrix by co-sputtering technology [49]. In the all-solid-state half-cell equipped with LiPON electrolyte, the SLC anode had a secondary discharge capacity of 420 mAh g^−1^ at a voltage window of 0–1.2 V and a current density of 1.96 mA^−1^, and the capacity retention rate was increased to 51%. In the full battery test, the initial capacity retention rate of SLC in the 0–0.3 V window was as high as 90%, which was far superior to that of pure tin electrodes. This was mainly because of the fact that the Li_2_O matrix effectively absorbs the volume change stress of tin particles during the charging and discharging process, inhibiting the initiation and propagation of grain boundary cracks. The presence of Li_2_O reduced the interfacial impedance between the electrode and the LiPON solid electrolyte and enhanced the surface mechanical strength through the LiPON deposition layer, thereby effectively hindering crack formation.

In the design of alloy-type anodes for solid-state batteries, constructing a multiphase composite system composed of active metals and inert metals represents an advanced materials engineering strategy, with the core lying in leveraging the complementary and synergistic functions of the two types of metal components. The formed nano-active metal domain not only provides additional reversible capacity, but more importantly, its relatively mild volume change behavior complements the intense expansion and contraction of the main alloy phase, essentially modulating the volume strain response of the entire complex, making it more uniform and controllable. The inert metal component mainly serves as a stable mechanical framework and an efficient electronic channel. The synergistic effect of active and inert metal components is reflected in the dynamic optimization of interface engineering and structural evolution. The active metal adaptively regulates local stress through its reversible volume change, while the inert metal frame provides global rigid constraints, jointly maintaining the macroscopic integrity of the electrode. However, the design of this system still faces challenges, including the precise control of the multiphase interface structure, the establishment of the optimal ratio and distribution of the two, and the cost control of complex preparation processes. This leaves a broad space for future theoretical research and material innovation.

#### Surface Engineering

The surface engineering strategy plays a crucial role in the optimization design of alloy-based anode materials, with its core being to address the key challenges faced during electrochemical cycling by regulating the surface interface structure and chemical properties of the materials. Surface engineering can effectively suppress mechanical stress caused by volume changes and enhance the structural integrity of materials by constructing surface structures with specific functions [80, 185]. For instance, introducing a carbon coating or a conductive polymer coating on the surface of the active material. This strategy not only enables the construction of a continuous and efficient electron transmission network, significantly enhancing the electronic conductivity of the electrode and accelerating the interface charge transfer kinetics process. It can also, by virtue of the flexibility and elasticity characteristics of the carbon-based or conductive polymer itself, act as an effective elastic buffer layer during the charging and discharging cycles, to accommodate the intense volume deformation caused by the active alloy materials during lithiumization/delithiumization processes. Moreover, this surface modification layer can serve as an artificial interface layer, guiding the formation of a stable and dense SEI film, reducing the side reactions of the electrolyte and the irreversible consumption of active lithium. It is worth noting that the thickness of the carbon coating needs to be precisely controlled. An overly thin coating may lead to insufficient mechanical protection, while an overly thick coating will increase electrode polarization and reduce the specific capacity ratio [186]. By surface passivation, element doping, or constructing core–shell structures, the interface ion migration barrier can be optimized, and dendrite growth can be suppressed, thereby improving Coulomb efficiency and long-term cycling performance. Surface engineering can also enhance the wettability of material surfaces to electrolytes, promote uniform diffusion of lithium ions, and further improve the kinetic process of electrochemical reactions [187]. Therefore, surface engineering is not only an effective interface control method, but also a key technical approach for the practical application of alloy-based anode materials, providing important support for their performance improvement in high-energy density solid-state lithium-ion batteries.

##### MOF Modification

The surface engineering strategy derived from metal organic frameworks (MOFs) has opened up a highly promising path for improving the electrochemical performance of alloy-based anode materials [188]. By utilizing the unique porous structure, high specific surface area, and uniform distribution of metal ions and organic ligands at the molecular scale of MOF precursors, highly functionalized modified layers or unique multi-level structures can be constructed in situ on the surface of alloy active materials through precise thermal conversion processes. It derived surface layer typically appears as a highly graphitized porous carbon matrix, in which nanoactive sites such as metal oxides, carbides, or phosphides derived from metal nodes in MOFs can also be embedded [189]. The unique structure provides multiple solutions to address the significant volume expansion and interface instability of alloy anodes during cycling. Recently, Qiu and team constructed a double-layer carbon encapsulation structure Si@C@C composite material, as an anode for high-performance all-solid-state lithium-ion batteries [110]. By using freeze-drying technology, citric acid is uniformly attached to the surface of silicon nanoparticles (Si NPs), and then calcined to form a single-layer carbon-coated material Si@C compound material. This single-layer carbon not only promoted the adsorption of zinc ions (Zn^2+^) through high negative charge density, but also used it as a template to guide the epitaxial growth of ZIF-8 crystals, ultimately resulting in thermal decomposition in an inert atmosphere Si@C@C composite material. Secondly, a chemical pre-lithiation method was introduced to compensate for irreversible lithium loss during the first charge and discharge. By pre-lithiation treatment in lithium coupling reagents, pre-lithiation Si@C@C-10% electrode ICE increased to 92.7% while reducing the charge transfer impedance. Finally, a polymer electrolyte based on in situ polymerization technology (PDOL-SN) was developed. The electrolyte used LiPF_6_ as the initiator, 1,3-dioxolane (DOL) as the monomer, and succinonitrile (SN) as the key additive. During the polymerization process, PF_5_ initiated the ring-opening polymerization of DOL to form polyDOL, while SN coordinated with lithium ions through cyanide groups to regulate the sol structure, significantly improving ion conductivity and oxidation stability. Therefore, Si@C@The C-10% electrode exhibited excellent rate performance and uniform stress distribution (Fig. 9e, f). The electrolyte was applied to the assembly of all-solid-state batteries, and the results showed that the initial ICE of the battery was 79.5%. After 300 cycles, the capacity retention rate reached 82.4%, demonstrating excellent cycling stability. The MOF-derived surface engineering strategy goes beyond the simple concept of traditional surface coating, achieving integrated and precise control of material surface chemical composition, electronic structure, pore structure, and mechanical properties, providing a powerful design platform for constructing the next generation of high-performance alloy-based anode materials.

##### Metal Modification

**Fig. 10:**
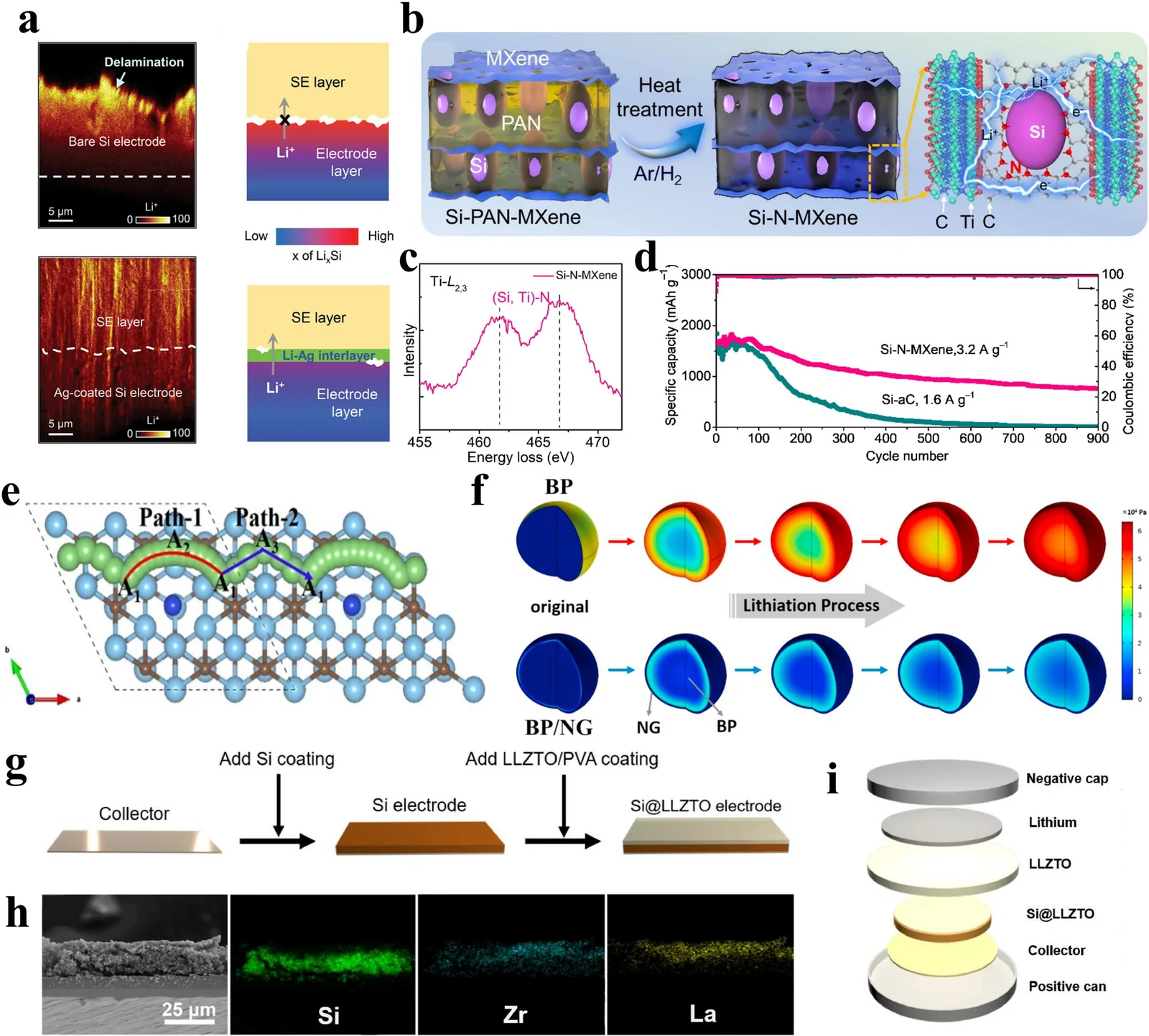
**a** TOFSIMS results after delithiation and schematic diagrams depicting the variations in state of charge across the depth of the SE layer and bare Si or Ag-coated Si electrodes [190]. © 2024 Wiley–VCH GmbH. **b** Synthesis process and schematic of the Si–N-MXene. **c** EELS analysis of the nitrogen interface: Ti-L2,3. **d** Cycling performance of Si–N-MXene and Si-aC electrodes. **e** Li ions diffusion pathway of Si–N-MXene [106]. © 2021 Science Press and Dalian Institute of Chemical Physics, Chinese Academy of Sciences. Published by ELSEVIER B.V. and Science Press. All rights reserved. **f** Evolution of residual stress in the BP particle and BP/NG particle during lithiation [195]. © 2021 Elsevier Ltd. All rights reserved. **g** Schematic illustration of the preparation process of the Si@LLZTO composite electrode. **h** Cross-sectional SEM image and EDS spectrum of the Si@LLZTO. **i** Schematic diagram of Si@LLZTO||LLZTO||Li all-solid-state battery [196]. © 2023 Elsevier B.V. All rights reserved

Metal nanoparticles can be introduced and anchored onto the surface of alloy materials through physical modification or chemical bonding as well, thereby constructing a composite interface structure with synergistic effects. Highly dispersed metal nanoparticles, as excellent electronic conductors, can significantly reduce the interfacial contact resistance of electrode materials. In addition, some metal nanoparticles exhibit excellent hydrophilicity and a high interfacial lithium diffusion coefficient toward certain alloy phases, such as Li–Si and Li–Sn. This guiding effect helps promote a more uniform lithiation process. Jun et al. innovatively introduced silver (Ag) interlayer engineering and pre-lithiation strategy, successfully solving the challenge of low-pressure operation [190]. The specific strategy included introducing a layer of Ag interlayer with a thickness of about 40 nm between the Si composite electrode and the solid electrolyte Li_6_PS_5_Cl (LPSCl) interface. During the charging and discharging process, Ag was transformed in situ into lithium silver alloy (Li-Ag), which exhibited outstanding deformability and adhesion, thereby significantly improving interfacial mechanical contact and ensuring efficient transport of Li, especially at a low pressure of 15 MPa. Experimental results showed that the initial discharge capacity of the Si electrode coated with Ag reached 2430 mAh g^−1^, which was much higher than that of uncoated Si. At the same time, the Ag interlayer acted as a barrier to suppress the harmful side reaction of Li_2_S formation between LPSCl and CNTs, and improve the chemical stability of the interface (Fig. 10a). In addition, pre-lithiation of the Si electrode with lithium metal deposited by thermal evaporation provided additional lithium reserves, compensating for the loss of active lithium during cycling. The pre-lithiated Ag-Si electrode exhibited excellent cycling performance in Si||Li in half cells. After 100 cycles, the capacity retention rate was about 67%, while it was 58% without pre-lithiation. In Si||NCM full battery, the capacity retention rate reached 73%. This method provided a practical strategy for the design of high-energy density ASSBs operating at low voltage, significantly reducing reliance on high-voltage equipment and improving system-level energy density.

In addition, the coupling mechanism of semiconductor physical-electrochemistry provides a key reference for the design of silicon-based anodes in miniaturized solid-state energy storage devices. In Rudy's research, the composite anode material Si@O@Al was prepared by magnetron sputtering technology, and its conductive matrix was an aluminum-doped amorphous silicon solid solution (a-Si(Al)), which initially exhibited p-type semiconductor properties [191]. Aluminum atoms introduce acceptor energy levels by replacing the lattice positions of silicon, significantly increasing the hole concentration. Meanwhile, oxygen elements are dispersed in the amorphous silicon network in the form of molecular clusters, passivating suspended bonds and forming percolation channels. The structural characteristic simultaneously optimizes the material's electronic conductivity and lithium-ion diffusion capacity. More importantly, the researchers have revealed the physical mechanism of the voltage platform rise in the charging curve and its correlation with the semiconductor properties of the electrode. During the lithiation process, lithium atoms, as donor impurities, gradually compensate for aluminum acceptor impurities, leading to a continuous transformation of the semiconductor carrier type. In the initial stage, P-type silicon and titanium current collectors form an ohmic contact, and the current is maintained by a composite current dominated by holes. With the increase of lithium concentration, a-Si(Al) underwent an intrinsic state transition and eventually transformed into an N-type semiconductor (a-Si(Li)). At this point, the Ti/Si@O@Al contact interface evolved into a reverse-biased Schottky junction due to the Fermi level shift, and the depletion layer formed, thereby significantly increasing the interface resistance [192]. In the constant current charging mode, to maintain the current density, the external voltage must be forcibly increased, which is manifested as a characteristic voltage "rise" phenomenon on the charging curve. The mechanism is particularly prominent in solid-state battery systems, as the LiPON solid-state electrolyte method penetrates the current collector/electrode interface like a liquid electrolyte, thereby shielding this semiconductor effect. Experimental data show that the critical concentration of lithium that triggers this transformation in the solid system is two orders of magnitude lower than that in the liquid system.

##### Non-Metallic Doping Modification

The surface engineering strategy of non-metallic doping is a profound and efficient method for regulating the interface properties and bulk electronic structure of alloy-type anode materials. Non-metallic elements are introduced into the protective layer (such as the carbon coating layer) or the shallow surface lattice structure of alloy materials through chemical means. This process is not merely a simple surface physical modification, but rather a reconstruction of the material's surface electronic density of states and charge distribution through the formation of chemical bonds, thereby essentially optimizing its electrochemical behavior [193]. The introduction of non-metallic elements significantly enhance the electronic conductivity of the surface modification layer. For example, the p electrons provided by nitrogen atoms can be delocalized into the π system of carbon, increasing the concentration of charge carriers and reducing the Fermi level, thereby significantly improving the kinetics of interfacial charge transport, ensuring efficient electron injection and extraction at high magnification, and alleviating electrode polarization [194]. More importantly, doped atoms can actively regulate the electrode/electrolyte interface reaction through their unique electronegativity and bonding characteristics. These doping sites, as functionalized active centers, can preferentially adsorb the components of the electrolyte and catalyze the formation of a more stable, denser, and mechanically excellent SEI film. Han and team designed a self-integrating monolithic silicon/two-dimensional layered MXene (Ti_3_C_2_T_x_) composite structure and introduced nitrogen functional group engineering treatment at the interface (Fig. 10b) [106]. Through the 700 °C heat treatment of PAN in a protective atmosphere, this process induced the transformation of PAN into amorphous carbon (A-C) layer coated silicon particles. More crucially, covalent chemical bonds of silicon-nitrogen (Si–N) and titanium-nitrogen (Ti-N) were generated in situ, achieving a robust anchoring connection between the silicon core and the Ti_3_C_2_T_x_ carrier (Fig. 10c). In solid-state battery applications, this design maintained a specific capacity of 880 mAh g^−1^ after 100 cycles at a current density of 0.4 A g^−1^, and the interface impedance was significantly reduced due to the fast ion channel (Fig. 10d). Furthermore, when Si–N-MXene was combined with a high-nickel NMC811 cathode to construct a full-cell structure that maintained a capacity retention rate of 80.5% after 200 cycles, with a specific capacity of 1811 mAh g^−1^, highlighting the practical value of this design in balancing energy density and cycle life. This was because of the fact that the formation of Si–N and Ti-N bonds significantly enhanced the mechanical adhesion between silicon and MXene. Nitrogen doping reduced the silicon adsorption energy on the surface of the Ti_3_C_2_T_x_ interface from −0.64 to −0.70 eV, thus enhancing the interfacial bonding stability. Meanwhile, the migration barrier of Li^+^ at the nitrided MXene interface was reduced to 0.15 eV, thereby effectively accelerating the diffusion rate of lithium ions within the electrode (Fig. 10e). This unique structural optimization not only maintained the structural integrity of the electrode under cyclic volume changes, but also promoted the formation of a stable SEI as well as suppressed the continuous electrolyte degradation side reactions caused by the fracture of silicon particles.

Yang et al. also designed an innovative BP/natural graphene (NG) composite electrode material to address the issues of low intrinsic electronic conductivity and significant volume changes during charge and discharge processes of black phosphorus (BP) as an anode material in all ASSLIBs [195]. The material constructed a covalently bonded C-P chemical bridge through an efficient ball milling process, significantly enhancing the interfacial bonding and charge transfer kinetics between BP and NG. Experimental characterization confirmed that the formation of C-P bonds not only effectively enhanced the migration rate of lithium ions and electrons in the composite, but also the flexible network structure of NG could buffer the huge mechanical stress generated by BP during lithiation/delithiation processes (Fig. 10f). The composite anode maintained a reversible specific capacity of up to 750 mAh g^−1^ after 100 cycles at a current density of 0.5 A g^−1^, while the coulombic efficiency remained stable at over 99%. Especially under high rate conditions of 1 A g^−1^, its discharge capacity still reached 300 mAh g^−1^, which was significantly better than the rate performance of uncomplicated pure BP material. Furthermore, the ASSLIB full battery assembled with BP/NG as the anode and TiS_2_ as the cathode maintained a reversible capacity of 400 mAh g^−1^ after 30 cycles, verifying the engineering application potential of this material system. This study, for the first time, achieved the highly reversible and fast charge-discharge lithium storage performance of black phosphorus in all-solid-state batteries through a synergistic strategy of chemical bonding and structural regulation.

##### Solid Electrolyte Modification

Furthermore, the in situ construction or introduction of a solid electrolyte protective layer on the surface of alloy materials through surface engineering strategies can effectively serve as an artificial buffering interface, such as LiPON, Li_7_La_3_Zr_2_O_12_ (LLZO), and sulfide electrolytes. The high mechanical modulus of solid electrolyte protective layer can suppress the volume expansion of the alloy, maintain a tight interface, and ensure the continuity of the ion transport pathway. By constructing a functional interface layer that combines high ionic conductivity, excellent mechanical strength, and good chemical stability, a tight physical contact and stable chemical compatibility between the anode and the solid electrolyte can be achieved. Zeng et al. coated a layer of solid electrolyte Li_6.4_La_3_Zr_1.4_Ta_0.6_O_12_ (LLZTO) with excellent lithium ion conductivity on the surface of silicon electrodes, combined with the synergistic effect of adhesive chemical bonds, which is used to engineer the solid-solid interface (Fig. 10g) [196]. They utilized the chemical bonding interaction between polyvinyl alcohol (PVA) and polyacrylic acid (PAA). During the preparation process, Si electrodes were first deposited on copper foils using conventional techniques, including nanosilicon powder, conductive carbon, and PAA binder. Subsequently, the slurry formed by mixing LLZTO powder with PVA/NMP solution was uniformly coated on the surface of the Si electrode. After drying treatment, the LLZTO coating formed a stable network through chemical cross-linking of PAA and PVA. This mechanism enhanced the interfacial adhesion, ensuring that the coating and silicon particles remained in close contact during volume expansion during lithiation (Fig. 10h). The high ionic conductivity (approximately 2.0 × 10^−3^ S cm^−1^) and mechanical strength of LLZTO not only promoted rapid transport of lithium ions at the interface, but also provided physical constraints to suppress the structural disintegration of silicon. More noteworthy is that when assembled into a fully solid-state battery (Fig. 10i), it maintained a specific capacity of 386 mAh g^−1^ after 1000 cycles at a high current density of 1.2 A g^−1^, with an average coulombic efficiency stable at 99.89%. They attributed these results to the promoting effect of the LLZTO coating on the stability of the SEI layer. This engineering interface method not only solves the problem of cyclic degradation of silicon-based electrodes, but also simplifies the process flow, with potential large-scale commercial value.

**Fig. 11:**
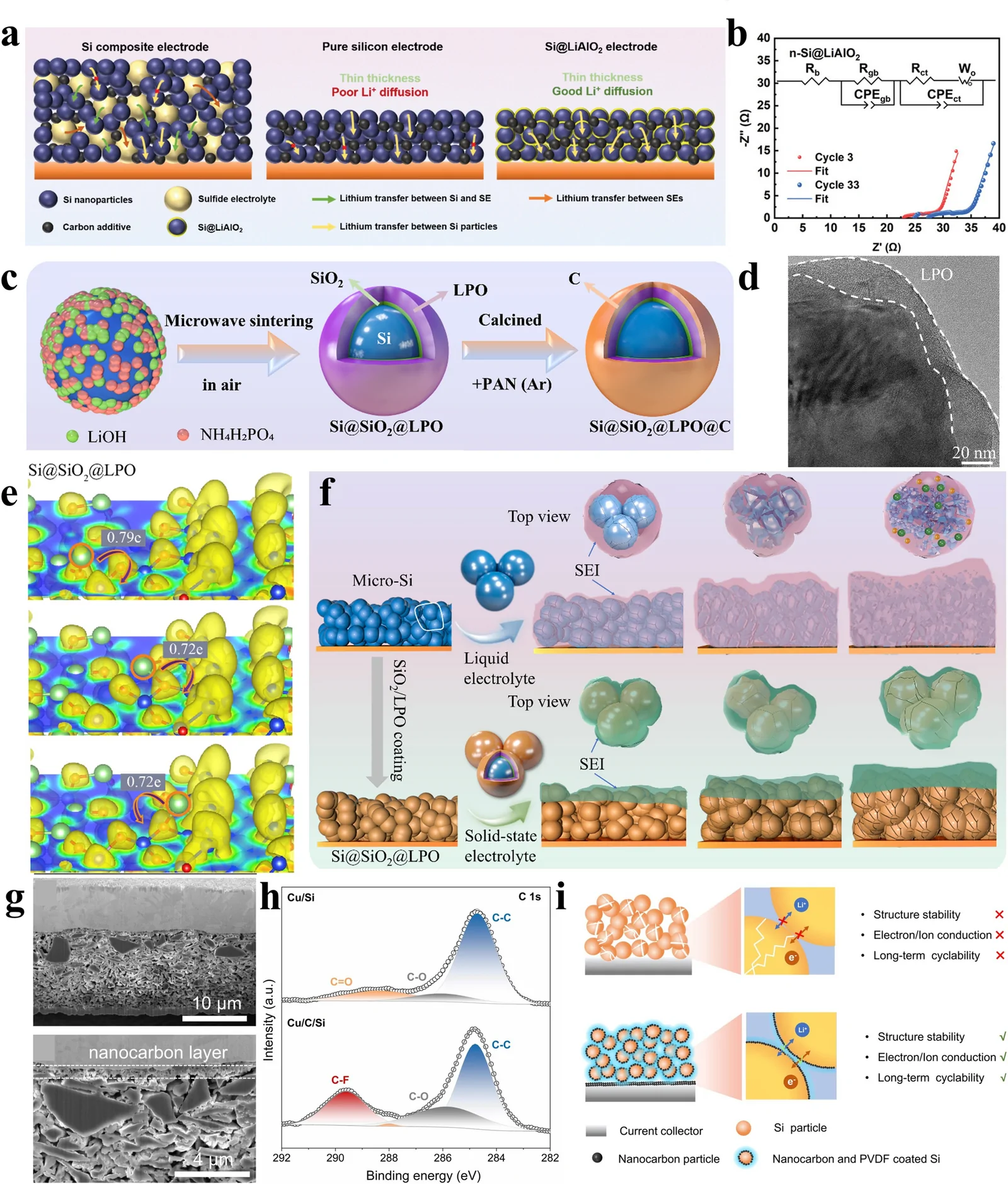
**a** Schematic diagrams of three different Si electrodes. **b** EIS results of Si@LiAlO_2_ after cycling [198]. © 2023 Wiley–VCH GmbH. **c** Synthesis processes of Si@SiO_2_@LPO@C material. **d** High-resolution TEM (Transmission Electron Microscopy) image of Si@SiO_2_@LPO particle. **e** Calculated electron localization functions (ELFs) for Si@SiO_2_@LPO surfaces. **f** Schematic illustration of the Micro-Si and Si@SiO_2_@LPO@C [105]. © 2022 Elsevier B.V. All rights reserved. **g** Cross-section images of Cu/C/Si after cycling. **h** The C 1*s* spectra of Cu/Si and Cu/C/Si ASSBs after cycling. **i** Schematic illustration of structural evolution of Cu/Si and Cu/C/Si after cycling [107]. © 2024 Elsevier B.V. All rights are reserved

In the research and development of alloy-type anode solid-state batteries, the traditional strategy of physically blending sulfide solid-state electrolyte coatings within the electrode is a common approach. However, the poor chemical and electrochemical stability of sulfide electrolytes themselves lead to inherent scientific challenges. These decomposition products usually have low ionic conductivity and poor electronic insulation, which not only significantly increases the interfacial impedance, but also leads to severe polarization and capacity attenuation. Secondly, sulfide materials are extremely sensitive to moisture in the air, and the preparation process of their mixing with electrode materials and slurry coating faces severe challenges [197]. Furthermore, from the perspective of mechanical properties, the modulus of sulfide electrolytes is relatively low, and their mechanical strength is insufficient. Abandoning the traditional practice of adding sulfide electrolytes to electrodes and instead directly coating the surface of alloy-type nanoparticles with lithium-ion conductive coatings might be a better approach. Xu et al. developed a novel sheet-like nano-silicon anode with a LiAlO_2_ coating (Fig. 11a) [198]. Specifically, the LiAlO_2_ coating was synthesized through the sol–gel method and subsequent heat treatment processes. Its properties include high mechanical strength and good ionic conductivity. The coating not only inhibited the free expansion of silicon particles but also provided a low-energy barrier channel for the diffusion of Li^+^ within the electrode. The method not only avoided the side reactions between sulfides and polar solvents, but also increased the volumetric capacity. The silicon anode coated with LiAlO_2_ significantly improved cycling stability and rate performance, and reduced impedance (Fig. 11b). At a rate of 0.33 C, after 150 cycles, the specific capacity remained at 1205 mAh g^−1^, and the initial value of the coulombic efficiency exceeded 80%, maintaining a relatively high level throughout the long-term cycle. These outstanding properties stem from the coating's effective restraint on the volume expansion of silicon. This study proposed a new strategy for the design of scalable sheet-like silicon anodes, breaking through the limitations of silicon electrodes in sulfide batteries.

In the field of silicon-based all-solid-state batteries, traditional designs require an external pressure of up to 370 MPa to maintain solid-solid contact and inhibit lithium dendrite growth. However, this external pressure demand increases the complexity of the system and hinders practical application. To address this challenge, researchers have proposed an innovative double-layer anode design composed of Li_21_Si_5_/ Si-Li_21_Si_5_, which generated a top Li_21_Si_5_ layer and a bottom Si-Li_21_Si_5_ layer through a cold-pressing sintering (600 MPa) process [130]. The upper Li_21_Si_5_ layer homogenized the electric field distribution on the anode surface, promoting the uniform injection of lithium ions. The lower Si-Li_21_Si_5_ layer provided an efficient ion/electron transport path, enabling silicon particles to be uniformly alloyed through the pre-formed Li_21_Si_5_ network. The collaborative mechanism effectively dispersed the stress generated by the expansion of silicon particles under the condition of no external pressure, stabilizing the interface between the anode block and the electrolyte. The self-discharge effect formed a 30–60 nm Li_x_Si coating layer on the surface of silicon particles, ensuring the rapid and uniform diffusion of lithium ions and preventing particle breakage and the formation of interface cracks. This double-layer anode demonstrated outstanding performance at 45 ℃ and zero external pressure. The solid-state full battery achieved an ICE of 97 ± 0.7% and a surface capacity of 2.8 mAh cm^−2^ at 0.25 mA cm^−2^. In terms of cycling stability, after 1000 cycles at 2.5 mA cm^−2^, the volume retention rate reached 54.9%, and the volume expansion rate was only 14.5%.

Therefore, the multi-level encapsulation strategy significantly improves the structural stability and interfacial electrochemical performance of silicon-based materials in solid-state lithium-ion batteries, which is an effective strategy. Similarly, Gu et al. reported an innovative micro silicon anode composite structure design (Si@SiO_2_@LPO@C), and constructed a three-layer functional structure on the surface of micro silicon particles, including an amorphous SiO_2_ intermediate layer, a Li_3_PO_4_ ion conductive layer, and a carbon protective layer (Fig. 11c-f) [105]. In terms of material design, the research team has for the first time constructed a multi-layer encapsulation structure with mechanical buffering and mixed ionic-electronic conductive properties. Among them, the introduction of the amorphous SiO_2_ intermediate layer significantly reduces the energy barrier for the transmission of Li^+^ from the Li_3_PO_4_ shell layer to the Si core. Density functional theory calculations further clarify that the amorphous SiO_2_ intermediate layer effectively promotes the interface transmission of lithium ions by regulating the interface charge distribution and providing P–O/Si–O bonding sites. The carbon-coated network provides a continuous electron conduction path, forming a three-dimensional interconnected dual continuous conductive network. This structural design enables the material to exhibit excellent cycling stability and rate performance in a liquid electrolyte system. In the application of solid-state batteries, it was found that the PEO(polyethylene oxide)/LiTFSI-based solid-state electrolyte formed a stable SEI layer rich in LiF on the surface of Si@SiO_2_@LPO@C. Different from the porous Li_2_CO_3_-based SEI formed in traditional liquid electrolytes, the SEI formed by the solid-state electrolyte only grows on the outer surface and has higher mechanical strength. The collaborative strategy of material design and electrolyte optimization reveals the mechanism of "limiting SEI growth at the outer layer" and the principle of enhanced interface ion transmission. This discovery can be extended to the research and application of other alloy electrode systems.

Similarly, Suebsing et al. achieved dual optimization of material bulk protection and interface stability through the synergistic effect of surface coatings and quasi-solid-state deep eutectic electrolytes [199]. At the material design end, polydopamine (PD) -coated micron silicon particles (PD@Si) were prepared by a wet chemical method. High-resolution transmission electron microscopy confirmed the acquisition of uniform and continuous amorphous PD coating layers at 5–7 nm. The characteristic nitrogen peak at 400–402 eV in XPS confirmed the chemical composition of the PD layer. This organic coating layer effectively suppressed the volume expansion effect of micron silicon by improving the kinetics of charge transfer and alleviating mechanical stress. At the electrolyte construction end, a quasi-solid gel electrolyte (GE) based on lithium bis (trifluoromethanesulfonimide)/N-methylacetamide (NMA) deep eutectic solvent and polyethylene glycol methacrylate (PEGMA)/polyethylene glycol dimethacrylate (PEGDMA) was developed. The system achieved a high ionic conductivity of 1.63 × 10^−3^ S cm^−1^ at 30 °C. Activation energy analysis indicated that it had an effective lithium-ion transport channel. Fluoroethylene carbonate (FEC) additives further optimized the stability of the electrode/electrolyte interface. PD@Si the anode maintained a reversible capacity of 1000 mAh g^−1^ after 100 cycles at 1 A g^−1^ in GE, which was four times higher than that of the liquid electrolyte system under the same conditions. The interface stability was improved by forming a stable SEI rich in LiF/C=O, inhibiting the formation of by-products such as Li_2_CO_3_, and effectively buffering the volume change mechanism of silicon through the gel network.

In the performance optimization of alloy-type anode materials, although the above-mentioned surface engineering strategies show diversity in implementation paths and material selection, their core themes focused on solving the inherent bottleneck problems such as volume effect, interface instability and slow transport kinetics by constructing a functionalized interface layer in a coordinated manner. These strategies achieve precise regulation of the thermodynamics and kinetics of electrode/electrolyte interface reactions by artificially intervening in the atomic arrangement, chemical composition and electronic structure of the surface and interface. Specifically, the surface modification layer physically acts as a mechanical barrier to buffer volumetric strain, effectively maintaining the structural integrity of the electrode and preventing the powdering of active substances, such as MOF-derived carbon matrices, metal nanoparticles, non-metallic doped carbon layers, or solid electrolyte protective layers. In terms of chemical interfaces, by providing preferred decomposition sites or serving as a stable phase itself, the protective layer induces the formation of a SEI film with high ionic conductivity, low impedance and mechanical stability, significantly suppressing continuous side reactions and the irreversible dissipation of active lithium. Therefore, surface engineering goes beyond the modification of a single function and presents multi-dimensional integrated advantages, providing a solid scientific and technological foundation for the application of alloy-type anode materials in solid-state battery systems. The core idea lies in transforming unstable and dynamic solid-solid or solid-liquid interfaces into controllable, stable, and efficient functional interfaces for ion transport. Ultimately, the simultaneous improvement of energy density and cycle stability is achieved.

#### Battery Processing

In solid-state battery systems, the synergistic optimization of electrode components such as binders, conductive agents, and current collectors is crucial for constructing stable and efficient electrode structures. Their role goes far beyond the simple auxiliary functions in traditional lithium-ion batteries, and has become a core strategy for solving the challenges of solid-solid interface contact, stress management, and multi-scale transmission. Therefore, the optimization of the overall electrode system is a multi-objective and multi-scale system integration problem. Through the functional design and collaborative interaction of each component, the structural integrity, interface stability, and efficient electron and ion transport capabilities of the electrode are jointly guaranteed, providing crucial basic support for achieving high-performance solid-state batteries.

##### Conductive Agents

The optimization selection and spatial distribution of conductive agents are directly related to the completeness and stability of the electronic conduction network inside the electrode [200]. Compared to traditional systems, more attention should be paid to the compatibility and dispensability between the electronic conduction network inside the electrode and solid electrolytes and active substances to avoid local polarization caused by uneven electronic conductivity. Sometimes it is even necessary to balance the competitive relationship between electronic conductivity and ion transport pathways in order to seek the optimal conductive network structure. In the study on the performance optimization of nano-porous silicon composite anode in all solid state lithium-ion batteries, Okuno et al. systematically explored the effect of acetylene black as a conductive additive (CA) on the electrochemical performance of the battery [201]. They designed a composite anode structure consisting of nano-porous silicon particles, Li_3_PS_4_ solid electrolyte, and acetylene black in a fixed weight ratio. The experimental results revealed the positive effect of conductive additives. They found out that the conductivity significantly increased with the increase of CA content, from 4.1 × 10^−4^ S cm^−1^ (x = 1) to 6.8 × 10^−4^ S cm^−1^ (x = 4), with an increase of about 1.7 times. This indicates that the addition of acetylene black forms a new conductive pathway and improves the electronic transport network. The charging capacity also increased proportionally after the addition of CA, from 2700 to 3015 mAh g^−1^, approaching the theoretical value of Li_15_Si_4_ (3579 mAh g^−1^). But it was still below the intrinsic potential Li_15_Si_4_, partly due to the irreversible consumption of lithium ions by the oxide layer of nanoporous silicon particles, resulting in an ICE of less than 70%. However, the amount of CA added is not necessarily better. After 100 cycles, x = 2 exhibited the highest discharge capacity (2071 mAh g^−1^) and optimal capacity retention rate (91%), far higher than the 17% of non-nanoporous silicon anodes. The nonlinear behavior was attributed to microcracks caused by excessive addition of CA (x ≥ 3), which weakened the elastic buffering mechanism of the solid electrolyte Li_3_PS_4_, thereby exacerbating the structural stress caused by the volume change of silicon.

Although conductive carbon black is often used as a conductive additive, combining one-dimensional carbon nanomaterials to enhance the continuous conductive network is an effective way to improve the electrochemical performance and structural stability of solid-state battery composite electrodes at the microstructure design level. The use of multi-walled carbon nanotubes (MWCNTs) as conductive additives is a highly promising electrode structure optimization strategy [202]. Its unique one-dimensional hollow tubular structure and excellent intrinsic physicochemical properties provide a multifunctional solution for addressing the challenges of ion/electron transport and mechanical stability in solid-state electrodes. Compared with traditional zero-dimensional or two-dimensional conductive agents, multi-walled carbon nanotubes, with their high aspect ratio and excellent mechanical toughness, can interweave within the electrode to form a stable three-dimensional interconnected conductive network. This network not only provides an efficient and low-resistance path for electron transmission. Trevey and team investigated the performance improvement mechanism of multi-walled carbon nanotubes (MWCNTs) as conductive additives in all-solid-state lithium-ion batteries based on nano-silicon (n-Si) active materials [203]. In one of the experimental studies, the composite electrodes were assembled into all-solid-state batteries using n-Si, solid electrolytes and MWCNTs with a weight ratio of 1:5:1. The results showed that MWCNTs, due to their higher specific surface area and mechanical flexibility, effectively buffer the lithiation/delithiation deformation of silicon particles and maintain the electronic conduction path. Compared with AB additive batteries, n-Si/MWCNTs batteries showed an approximately 100% increase in specific capacity. The reversible specific capacity exceeded 900 mAh g^−1^ in over 100 cycles, and the initial discharge capacity reached 2013 mAh g^−1^. It is worth noting that cyclic tests within different voltage ranges have revealed optimization strategies. Although the 0.05–1.0 V narrow window reduces the initial capacity, it significantly increases the capacity retention rate. This is attributed to the avoidance of the formation of harmful crystal phases of c-Li_15_Si_4_ in the low-voltage region. On the contrary, although large blocks of silicon have a relatively high initial capacity in the high-voltage range, their capacity decays to 200 mAh g^−1^ within 10 cycles due to severe expansion. The n-Si undergoes a phase transition from crystalline to amorphous silicon during the cycle, while MWCNTs reduce the decomposition kinetics problem of solid electrolytes by providing an extended interface. It indicates that the combination of MWCNTs and nano-silicon not only enhances the cycle life but also provides a feasible path for the commercialization of high-energy-density solid-state batteries.

##### Current Collector

As a carrier for electron collection and transmission, the surface modification of the current collector can also significantly enhance the mechanical interlocking and electrochemical coupling with the composite electrode layer, such as constructing a three-dimensional porous structure and applying lithium-friendly or ion-conductive coatings. Meanwhile, this reduces the interfacial contact resistance and effectively guides the uniform distribution of lithium ion flow, inhibiting dendrite formation. Huang et al. prepared microsilicon (μSi) electrodes on copper foils coated with nano-carbon layers. During the charging and discharging process, nano-carbon particles diffuse into the interior of silicon particles, forming a carbon-coated structure and activating the carbon-PVDF cross-linked network [107]. This structure had the following functions: 1) The carbon–fluorine bond enhanced the interfacial bonding force, alleviating the volume expansion stress of silicon particles, and inhibited particle breakage, agglomeration and microcrack formation caused by repeated expansion and contraction (Fig. 11g, h); 2) It homogenized the distribution of lithium ion flow within the electrode and reduces local stress concentration at the interface; 3) The carbon-coated structure maintained the electronic path at the current collector interface through the residual carbon layer on the surface to reduce the phenomenon of interface peeling. Therefore, solid-state full batteries exhibited excellent electrochemical performance in this case. The 2 C discharge specific capacity reached 55 mAh g^−1^. The capacity retention rate was 80.2% after 450 cycles at 0.8 C and 73.1% after 1340 cycles at 2 C. Even at 60 ℃, after 100 cycles at 0.8 C current, the capacity retention rate was 86.3%. The amorphous carbon layer formed by the reaction during the electrochemical cycle and the binder PVDF were used to co-construct a three-dimensional lithium-ion/electron dual continuous transport network, achieving the structural stability and interface kinetics optimization of the silicon anode (Fig. 11i).

##### Binder

The lack of sufficient wetting and penetration of liquid electrolyte in the electrode of solid-state batteries makes ion transport highly dependent on close physical contact. Therefore, the binder should have strong mechanical bonding force and certain flexibility or self-healing characteristics to maintain the lasting contact between active substances, solid electrolyte particles and conductive agents during the repeated volume expansion and contraction of the alloy anode material, thereby preventing the electrode structure from pulverizing and interface contact failure, and at the same time, it should have certain ionic conductivity to assist local ion transmission. Sadati et al. developed a functional binder system based on organic ionic plastic crystal (oipc), which significantly improved the electrochemical stability of solid silicon anode [204]. The composite silicon anode (Si-C2mpyrFSI) was constructed by introducing an oipc electrolyte composed of n-ethyl-n-methylpyrrolidine bis (fluorosulfonyl) imide (C2mpyrFSI) and lithium bis (fluorosulfonyl) imide (LiFSi) in a molar ratio of 50:50 into carboxymethyl cellulose (CMC) binder. The introduction of oipc significantly improved the ionic conductivity in the electrode, which was three orders of magnitude higher than that of the silicon anode with a traditional CMC binder. Its property promoted the uniform diffusion of lithium ions between silicon particles, and inhibited the formation of local superlithified phase through the in situ ion conduction network. Electrochemical tests showed that after 60 cycles of limiting the lithium insertion capacity to 1000 mAh g^−1^, the lithium removal capacity retention rate of the Si-C2mpyrFSI anode reached 96.1% (961 mAh g^−1^), while the capacity of the traditional silicon anode decreased significantly. The plasticization effect of oipc on CMC significantly improved the flexibility of the binder. FTIR spectra detected the Si-OOC covalent bond formed by the esterification reaction between the silanol group on the surface of silicon particles and the carboxyl group of CMC at 1633 cm^−1^, which confirmed that the composite binder enhanced the interfacial adhesion through chemical bonding. Differential capacity analysis further revealed that there was an additional reduction peak near 0.24 V in the process of lithium insertion at the composite electrode, which confirmed that the oipc mediated lithium-ion diffusion path optimized the phase transition kinetics. The oipc-cmc composite binder system provides an effective strategy for solving the capacity attenuation and mechanical failure of solid silicon anode by synergistically improving the homogeneity of ion conduction, regulating the mechanical stress distribution and strengthening the interface. In addition, Vieira et al. used titanium oxide (TiO_x_) instead of traditional metal titanium as the adhesive layer, which effectively solved the interface failure problem of all solid-state lithium battery multilayer structure (Si/Si_3_N_4_/Ti/Pt/LiCoO_2_) after high temperature annealing (700 ℃) [205]. Three kinds of titanium oxide layers with thicknesses of 25, 35, and 45 nm were prepared by thermal oxidation deposition. It is found that the migration of titanium oxide induced by high temperature annealing is significantly thickness-dependent. The thicker titanium oxide layer caused an increase in residual free titanium atoms due to incomplete oxidation, which intensified the migration of TiO-species to the lithium cobalt oxide positive layer, and caused lithium ions to penetrate the silicon nitride layer and diffuse to the silicon substrate, damaging the structural stability. The 25 nm titanium oxide layer showed the best performance. It not only effectively blocked the diffusion of titanium atoms along the grain boundary of the platinum layer and maintained the structural integrity of Pt/TiO_x_/Si_3_N_4_, but also retained the inhibitory effect of silicon nitride barrier on lithium diffusion. This optimization scheme provides a key interface engineering strategy for the preparation of alloy solid-state batteries, and is expected to improve the thermal stability and cycle life of thin-film lithium batteries.

**Fig. 12:**
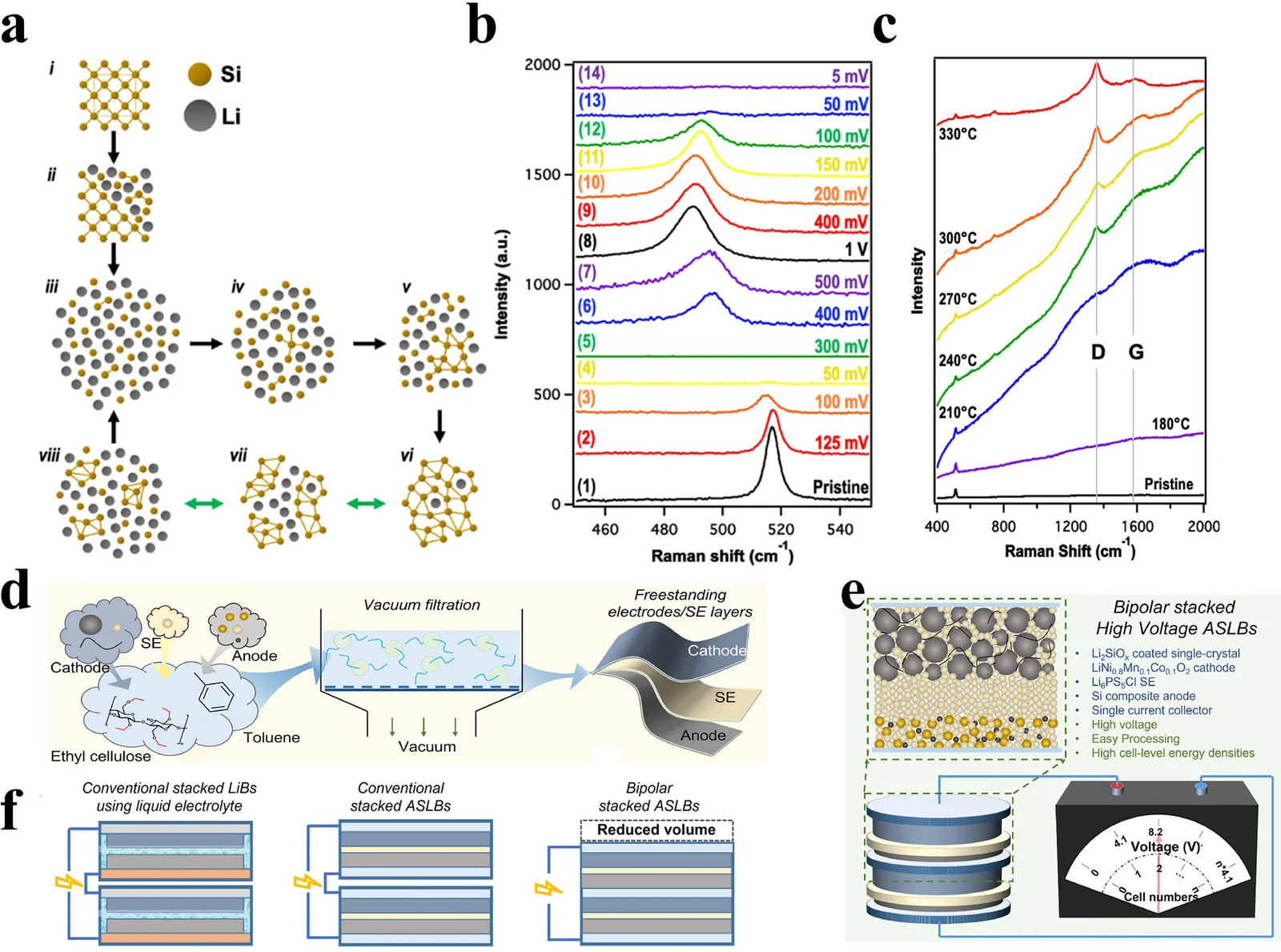
**a** Schematic of Si-PAN materials upon discharging/charging processes. **b, c** Ex situ Raman spectra of Si-PAN electrodes [206]. © 2020 The Electrochemical Society (“ECS”). Published on behalf of ECS by IOP Publishing Limited. **d** Fabrication process via vacuum filtration. **e** Merits of the high voltage bipolar-stacked ASLBs and composition distribution in each layer. **f** Schematic illustration of conventional stacked LiBs using liquid electrolyte, conventional stacked ASLBs, bipolar stacked ASLBs [104]. © 2022 Elsevier B.V. All rights reserved

Through innovative material design and structural regulation, a high-performance anode system that can be prepared on a large scale can be developed to further solve the inherent problems of alloy anode solid-state batteries. Dunlap and team used the mixed conductive network formed by the thermal-induced cyclization of Pan matrix to replace the independent function of binder and conductive agent in the traditional electrode (Fig. 12a) [206]. This structure design makes the electrode with silicon active material content up to 70 wt% show significant electrochemical performance. The uniform coating of silicon nanoparticles in Polyacrylonitrile not only provided mechanical support, but also maintained the structural integrity of the electrode in the process of deep lithiation through chemical bonding effect (Fig. 12b, c). The experimental data showed that the electrode after 270 °C heat treatment can stably output a reversible capacity of about 1500 mAh g^−1^ at 1 C rate, and still maintained a high capacity of 1122 mAh g^−1^ after 200 cycles. Especially, by accurately adjusting the discharge depth, the irreversible structural changes of silicon particles to completely disintegrate into isolated anions/dimers was successfully avoided. The four coordinated silicon cluster structure (Si_4_ tetrahedral unit) is always maintained in the silicon particles, so as to realize the topological reversible phase transition in the process of lithiation/delithiation. It was also found that limiting the lithium insertion depth (≥ 100 mV) could significantly inhibit the internal stress accumulation caused by deep lithium. This research creatively revealed that the cyclic degradation of the silicon anode is not only due to the mechanical pulverization effect, but also directly related to the destruction of local tetrahedral bonding structure under the condition of deep discharge.

##### Slurry Engineering

The application bottleneck of alloy-type materials in solid-state batteries has been broken through slurry engineering. The characteristics of this method, such as the low cost and bringing high specific capacity have significantly advanced the industrialization process of all-solid-state batteries. Yamamoto et al. reported an innovative slurry mixing process for the preparation of silicon-based composite electrodes in all-solid-state batteries [207]. The process constructed an optimized ion/electron conduction network in a composite system of silicon particles, 75Li_2_S_25_P_2_S_5_ glass solid electrolyte and conductive carbon black by using zirconia (ZrO_2_) ball-assisted wet mixing. During the electrode preparation process, the mechanical force mediated by ZrO_2_ spheres achieved controllable crushing of solid electrolytes, thereby reducing the particle size to the sub-micron level and significantly enhancing the contact interface between solid electrolytes and active material silicon. When the proportion of conductive carbon black was optimized to 10 wt%, the binder electrode exhibited an ICE as high as 9% and a reversible capacity of 3058 mAh g^−1^. Most importantly, the electrode maintained a capacity of 1700 mAh g^−1^ after 375 cycles at a current density of 0.3 mA cm^−2^, and its coulombic efficiency remained stable at over 99% after 64 cycles, effectively suppressing the volume expansion effect of micron silicon. In addition, this electrode exhibited a surface capacity as high as 4.4 mAh cm^−2^. In the all-cell design, the energy density of the Si/SE/NCM system constructed by coating a thin layer of electrolyte on the surface of the silicon composite electrode reached 212 Wh kg^−1^.

##### Stacking Design

Compared with traditional liquid lithium-ion batteries, ASLBs using solid electrolyte have higher safety and thermal stability, but their performance optimization is limited due to the difficulty of electrode interface integration. In the solid-state battery system, the bipolar stack design is used as an integrated optimization strategy at the system level [208]. By innovating the multi-cell series architecture inside the battery, the energy density and power characteristics of the whole battery system are significantly improved, and the mechanical integration and interface management are synchronously optimized. This multi-cell series architecture has greatly reduced the mass and volume ratio of inactive substances, and achieved a fundamental breakthrough in the energy density at the battery system level. Cao and team achieved the goal of improving the energy density of ASLBS through an innovative bipolar stacking design, which significantly reduced the use of inactive materials (Fig. 12d) [104]. Researchers have proposed a new strategy to prepare a self-supporting electrode and electrolytic layer by the vacuum filtration method. Ethyl cellulose was used as an amphiphilic adhesive to ensure its stable dispersion in toluene solvent, and a thin film with uniform thickness and high mechanical strength was formed by the hot-pressing process (Fig. 12e). The key materials included single crystal LiNi_0.8_Mn_0.1_Co_0.1_O_2_ with a stable interface as cathode active material and nano silicon as high-capacity anode, combined with ultra-thin Li_6_PS5Cl electrolytic layer, effectively reducing the ion-conduction resistance. The system achieved a reversible discharge capacity of 145 mAh g^−1^ at C/10 rate and an energy density of 266 Wh kg^−1^. It is due to the inhibition of interface side reactions by material optimization. The innovation of the bipolar stack design is that multiple single cells are directly connected in series to share the current collector, avoiding the mass loss caused by additional sealing and external connection in the conventional battery (Fig. 12f). The experimental data showed that the output voltage of the stacked double cells is increased to 8.2 V, and the battery level energy density is increased to 204 Wh kg^−1^ at the C/10 ratio, which was due to the optimization of the proportion of inactive materials, and the shortening of the electron transmission path also reduced the internal resistance in the parallel process. However, the promotion of bipolar stacking still faces challenges, such as the consistency of each cell is difficult to guarantee, and the volume expansion of the anode silicon in the process of charge and discharge threatened the mechanical integrity of the electrolytic layer. This needs to be optimized by developing more efficient adhesives and increasing the proportion of active materials. In short, this work emphasizes the key role of bipolar architecture in the commercialization of solid-state batteries and lays a foundation for the innovation of material engineering and preparation process in the future.

In addition, it is very important to design lithium-ion batteries to deal with extreme conditions and improve the safety and life of the system. For example, the local hot spot can reach up to 150 °C when the battery is fast charged or over-discharged. Therefore, the design of high-temperature-resistant lithium-ion batteries is a current development direction for the next-generation battery. Cras and team used the amorphous silicon anode prepared by the sputtering method and a new type of lithium titanium oxide sulfide Li_1.2_TiO_0.5_S_2.1_ cathode, combined with the lithium solid electrolyte, to build a high-temperature-resistant (> 230 ℃) and short-circuit-resistant micro-battery structure [209]. During the charge-discharge process, the lithium reaction of the silicon film presented two stages, which occured at 0.23 and 0.08 V, respectively. When the discharge depth exceeded 0.05 V, the crystalline Li_15_Si_4_ phase precipitated, which was limited by dynamics and needs a long time constant voltage polarization trigger. It is worth noting that the silicon anode had a "memory effect" when circulating in a specific charge-discharge interval. The residual lithium content in the electrode gradually accumulated, resulting in an increase in delithiation voltage and a decline in reversible capacity. This effect can be completely eliminated by deep discharge above 0.6 V, which is essentially due to the diffusion rearrangement of lithium ions in lithium-rich silicon alloys. More importantly, after three consecutive 260 ℃ heat treatments, the capacity, cycle stability and ultrafast charging capacity of the battery did not change significantly.

In the research and development of solid-state batteries, the optimization strategy for the overall electrode system constitutes the core research direction for enhancing the comprehensive performance of batteries. The fundamental objective lies in systematically addressing key challenges such as solid-solid interface contact, stress management, and transport dynamics caused by solid-state properties through multi-component and multi-scale collaborative design. The electrode system is no longer regarded as a simple mixture of active substances, conductive agents, and binders, but needs to be designed as an integrated functional unit, where the selection and interaction of each component directly determine the structural integrity and electrochemical behavior stability of the electrode. The function of the binder goes beyond the traditional bonding effect. It needs to have excellent mechanical toughness, certain ionic conductivity, and even self-healing properties to maintain the persistent physical contact between the alloy anode with drastic volume changes and the rigid solid electrolyte particles, as well as to prevent the degradation of the electrode structure during the cycling process. The optimization of conductive agents such as one-dimensional multi-walled carbon nanotubes aims to construct a stable and efficient three-dimensional electronic conduction network. This network not only ensures the rapid transport of electrons, but also its interwoven porous structure facilitates the uniform migration of lithium ions and enhances the structural stability of the electrode through its own mechanical properties. The surface engineering of current collectors is dedicated to enhancing their electrochemical and mechanical coupling with the active layer, optimizing the electronic distribution at the interface, and suppressing the uneven deposition of lithium. Ultimately, these strategies, through system integration, jointly aim to achieve the coordinated management of electrons, ions, and stress within the electrodes, constructing a composite electrode system that combines excellent transmission characteristics, interface stability and mechanical robustness, providing a crucial material and structural foundation for promoting the practical application of high-energy-density, long-life solid-state batteries.

## Solid Electrolytes in Solid-State Lithium-Ion Batteries

### Characteristics of Solid-State Electrolytes in Solid-State Lithium-Ion Battery

As the demand for lithium-ion batteries to achieve higher safety and energy density continues to increase, traditional liquid electrolytes are gradually exposing their limitations due to issues such as flammability, leakage, and inability to effectively suppress lithium dendrites. Against this backdrop, solid-state electrolytes have attracted widespread attention due to their excellent thermal and electrochemical stability.

As a key component of solid-state batteries, solid electrolytes have many advantages over traditional liquid electrolytes: 1) Solid-state electrolytes significantly improve battery safety primarily due to their inherent non-flammability, excellent mechanical stability, and ability to inhibit lithium dendrite growth. Compared to traditional liquid electrolytes, solid-state electrolytes are typically composed of solid materials such as ceramics or polymers. They are inherently less flammable, less volatile, and less leak-resistant, fundamentally reducing the risk of thermal runaway and explosions. Many solid-state electrolytes possess a high Young's modulus and mechanical strength, which physically resist the penetrating growth of lithium dendrites, effectively preventing internal short circuits. They also play a significant role in counteracting the volume expansion of anode materials. For example, alloy anodes such as silicon and tin undergo significant volume changes during lithiation. Solid-state electrolytes can, to a certain extent, suppress the structural damage caused by anode volume expansion. Solid-state electrolytes maintain structural stability and electrochemical inertness at high temperatures, avoiding the flammable gas generation that liquid electrolytes readily decompose at high temperatures or under overcharge conditions, further improving thermal stability and overcharge resistance [210]. 2) Solid-state electrolytes act as both electrolytes and separators in batteries, which can reduce the weight of batteries, give full play to the advantages of high specific capacity of lithium-ion batteries, and significantly improve the energy density of batteries [211]. 3) They broaden the usable temperature range of the battery: Most solid electrolytes have better thermal stability than organic electrolytes, enabling them to maintain high ionic conductivity and mechanical properties at high temperatures and maintain stable operation [212]. 4) In practical applications, solid electrolytes are usually required to remain stable under high voltage and not decompose, so as to be compatible with high-voltage electrode materials and improve battery energy density. For example, some solid electrolytes (such as Li_7_La_3_Zr_2_O_12_) can be stable in the 0–5 V range, supporting high-voltage cathode materials (such as NCM, LCO, etc.), while avoiding safety issues caused by electrolyte decomposition under high voltage [213]. 5) Solid electrolytes have a longer service life and better chemical stability. They do not decompose over time like liquid electrolytes, reducing the occurrence of side reactions, thereby extending the service life of the battery [214].

#### Mechanism of Solid Electrolytes

**Fig. 13:**
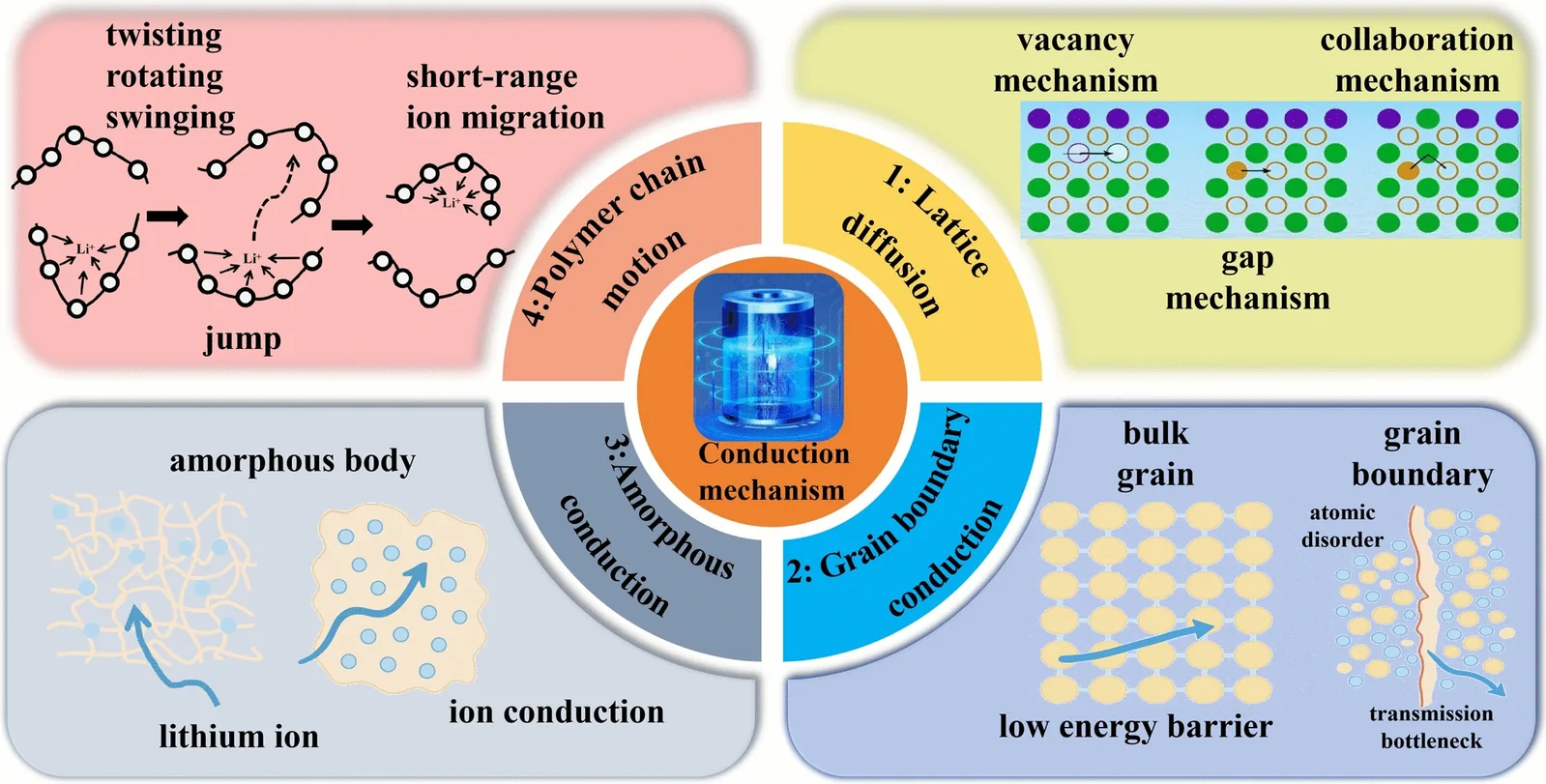
Schematic diagram of the four main conduction mechanisms of solid electrolytes [215–221, 223]. © The Author(s) 2024. © 2022 The Authors. © 2023 Muhammad Khurram Tufail et al. © 2023 The Authors. © 2024 the Author(s). Published by PNAS. © The Author(s) 2024. © the Owner Societies 2024. © 2022 American Chemical Society

Solid-state electrolytes are materials that can conduct ions in their solid state and are widely used in solid-state batteries, sensors, and other electrochemical devices. Unlike liquid electrolytes, where ions move freely through the liquid medium, solid-state electrolytes conduct current through the migration of ions within the solid material. It is essentially the migration of ions within the solid lattice or amorphous structure. The specific conduction modes mainly include lattice diffusion, grain boundary conduction, amorphous conduction, and polymer chain segment movement. To better understand the various conduction mechanisms of alloy-type anode solid electrolytes, this article sorts out the four most important mechanisms: lattice diffusion, grain boundary conduction, amorphous conduction, and polymer chain motion (Fig. 13) [215–223]. The following is a detailed explanation of the conduction mechanism of solid electrolytes:

##### Lattice Diffusion

Since most inorganic solid electrolytes such as oxides, sulfides and halides have highly ordered crystal structures, lattice diffusion is widely present in these materials [224]. Unlike liquid electrolytes where ions diffuse freely in the solvent in a Brownian motion, ions in solid electrolytes are restricted by the lattice structure and can only move by jumping from a stable lattice site to another vacant or interstitial site. This process usually requires overcoming a certain potential barrier, called the migration energy barrier, which depends on the type of ion, the crystal structure, the surrounding atoms, and the defect state in the crystal [225]. Due to the different jump destinations, lattice diffusion is further divided into vacancy diffusion and interstitial diffusion. There are a large number of vacant positions in the lattice that should be occupied by ions. The ions in the lattice can jump through these vacancies to achieve the purpose of movement, which is called vacancy diffusion [219]. The efficiency of this mechanism depends on the concentration of vacancies in the lattice and the energy barrier for ion migration. The more vacancies there are, the lower the energy barrier for migration, and the easier it is for ions to migrate within the lattice. In addition, since the ions in the lattice are not tightly packed, there must be gaps between different ions, so ions can move by jumping through the gaps in the lattice structure. This diffusion method is called interstitial diffusion [220]. Since the gaps between ions are small, interstitial diffusion can usually only occur in ions of smaller size. Generally, vacancies or gaps in the lattice are necessary channels for ion hopping. The way to optimize this channel is to artificially introduce an appropriate amount of doping elements to control the vacancy concentration, thereby increasing the diffusion rate and overall ionic conductivity. It is worth noting that the rate of lattice diffusion depends not only on the geometric properties of the diffusion path, such as ionic radius, bond length, and lattice constant, but is also closely related to temperature and follows the Arrhenius behavior, that is, the higher the temperature, the more thermal energy the ions obtain and the easier it is to overcome the diffusion barrier [218]. In practical applications, high-quality solid electrolytes should have low diffusion barriers, high ion mobility, and good structural stability, allowing ions to efficiently and directionally move through lattice channels, thereby meeting the battery's requirements for high conductivity and reliability. Therefore, understanding and optimizing the lattice diffusion mechanism is one of the core scientific issues in designing new high-performance solid electrolytes.

##### Grain Boundary Conduction

The grain boundary conduction mechanism of solid electrolytes refers to the complex physical and chemical process of the transmission of ion carriers through the grain boundary region in polycrystalline materials. This mechanism is usually a key factor limiting the overall ion conduction performance of solid electrolytes. Solid materials have grain boundaries as transition regions between different grains. The grain boundaries have a highly disordered atomic arrangement structure and have dislocations, dangling bonds, vacancies, and other defects. These structural features make the ion conduction environment in the grain boundary region significantly different from that inside the grain. When ions cross from one grain to other adjacent grains, they must overcome a higher activation energy barrier, which makes the grain boundary resistance usually 1–3 orders of magnitude higher than the resistance inside the grain [226]. The difficulties in ion conduction at grain boundaries mainly include the disorder of atomic arrangement, lattice distortion, the formation of a space charge layer and the existence of an interface energy barrier. These can be summarized as follows: 1) The disordered state of the atomic arrangement destroys the diffusion channels or "channel connectivity" in the crystal. In an ideal lattice, ions migrate along the periodically arranged potential wells, but at the grain boundaries, due to the disordered arrangement of atoms, the potential wells are of different depths, and ions must overcome higher uneven potential barriers to pass through [227]. It is like when the road becomes bumpy, it becomes more difficult for cars (ions) to drive, resulting in the ionic conductivity at the grain boundaries being generally lower than that of the inside of the grains. Dawson et al. used lithiated ore Li_3_OCl as a model and, through large-scale molecular dynamics simulation, and clearly found that the activation energy of lithium ion migration at the grain boundary was higher than inside the grain [228]. The grain boundary hinders the cross-boundary migration of ions, and its conductivity is significantly reduced. This confirms that the disordered arrangement between different atoms at the grain boundary and inside the grain acts as a bottleneck to limit the overall conductivity. 2) In the grain boundary region, lattice distortion (low atomic packing density, insufficient coordination number, and lattice parameter mismatch) causes changes in the ion conduction path. 3) At the grain boundary, due to the incompleteness of the atomic arrangement and the change of the chemical environment, a space charge layer effect will be formed, that is, the grain boundary captures carriers to form a depletion layer, generating a built-in electric field that hinders ion migration [221]. Quirk et al. [229] performed first-principles calculations on representative grain boundaries in four important solid electrolytes, namely, antiperovskite oxide Li_3_OCl and its hydrated counterpart Li_2_OHCl, thiophosphate Li_3_PS_4_, and halide Li_3_InCl_6_. The results showed that there were significant differences in the effects of grain boundaries on electronic structure and transport, ionic conductivity, and related ion dynamics. They also found that even if grain boundaries do not directly affect ionic conductivity, they still provide the prerequisites for potential lithium dendrite growth by disrupting the electronic structure. These findings reveal the different behavious of solid electrolytes than the bulk at the microscale as well as their potential impact and benefits on solid-state battery design. 4) Grain boundaries often enrich impurity elements or form secondary phases, further increasing the resistance to ion transport. In addition, different types of grain boundaries and grain boundary structures significantly affect the diffusion channels and migration efficiency of ions. Therefore, the physicochemical properties of grain boundaries play a key role in determining the overall ionic conductivity of solid electrolytes. A deep understanding of their conduction mechanism is of great significance for the design of high-performance solid electrolyte materials.

##### Amorphous Conduction

Amorphous conduction is one of the important modes of ion migration in solid electrolytes, and mainly exists in amorphous or glassy materials, such as sulfide glass and oxide glass [230]. The core feature of amorphous conduction in these materials is that they not only have short-range order but long-range disordered atomic arrangement patterns as well, but lack long-range ordered crystal structures, making the ion migration path more continuous, flexible, and diverse, and no longer subject to the periodic restrictions of the lattice potential well. This forms a "quasi-continuous diffusion channel", which causes the ion conduction path to ultimately show a high degree of non-uniformity and randomness, which is conducive to the rapid migration of ions across lower energy barriers [231]. Unlike ions in crystalline materials that migrate through regular lattice vacancies or interstitial sites, amorphous conduction relies on the dynamic rearrangement of the disordered structure of the material to form a continuous ion migration channel. In addition, there are usually more structural vacancies, defects, and soft molecular segments or coordination environments in the amorphous state, which enable ions to undergo thermal excitation hopping at lower temperatures, thereby achieving higher ionic conductivity. For example, metal cations jump and move through the local free volume or weakly bonded regions in the amorphous network [232]. Since the amorphous structure lacks long-range order, the energy barrier for ion migration is usually low, so the ion transport performance is optimized by adjusting the composition of the material, for instance, the S^2−^ network in the Li_2_S-P_2_S_5_ system. Dietrich et al. [233] studied the relationship between the local structure and lithium ion conductivity in the Li_2_S-P_2_S_5_ amorphous glass. Through various methods such as Raman spectroscopy, ^31^P MAS NMR and synchrotron X-ray total scattering, the trend of the content of different thiophosphate building blocks (such as PS_4_^3−^, P_2_S_7_^4−^) in the glass changing with the Li_2_S ratio was revealed. The study found that as the Li_2_S content increased, the proportion of PS_4_^3−^ units increased, the activation energy of ion migration decreased, and the conductivity increased. The 75:25 component had the highest conductivity and the best thermal stability. Through in situ structural evolution analysis, it was also found that internal redox reactions occurred during the crystallization process, affecting the final performance. In addition, amorphous materials often have a wider electrochemical window and better interfacial contact properties because their grain boundary-free structure can reduce interfacial impedance. However, the ionic conductivity of amorphous electrolytes is strongly dependent on the composition design and preparation process. For example, the introduction of LiI or Li_2_O can significantly improve the lithium ion mobility [234]. Therefore, the structural characteristics of the amorphous phase itself provide solid electrolytes with a controllable and efficient conduction mechanism that is different from traditional crystals, which is also an important research direction for the design and optimization of solid electrolyte materials.

##### Polymer Chain Motion

The movement of polymer chain segments in solid electrolytes is a complex multi-scale dynamic process, mainly involving local relaxation and cooperative motion of molecular chains. This mechanism mainly relies on the chain segment motion of the amorphous region of the polymer to transport metal ions. The core of its research is to achieve rapid ion transport [235]. At the microscopic level, the polymer’s main chain undergoes conformational changes through the internal rotation of the C–C bond. This local chain segment motion provides a dynamic conduction channel for ion transport. Specifically, the metal ions first coordinate with the polar groups (such as ether bonds and carbonyl groups) on the polymer chain to form a complex [223]. Under the action of an electric field and when the temperature rises above the glass transition temperature of the polymer, the amorphous polymer chain segments undergo local cooperative thermal motion, that is, chain segment relaxation. The movement of this chain segment provides a mobile "carrier" and free volume for the ions coordinated with it. Through the twisting, rotation and swinging of the chain segment, the ions can be transported in a jumping manner in the ever-changing polymer matrix, and the metal ions can "jump" from one coordination site to a new coordination site on the adjacent polymer chain or on the same chain, thereby achieving short-range ion migration [236]. The entire ion transport process is manifested as the continuous jumping of metal ions within and between polymer chains, accompanied by the continuous breaking and reforming of coordination bonds. In this process, the movement shows a significant temperature dependence, specifically following the Vogel-Tammann-Fulcher (VFT) equation, reflecting the close relationship between ion conduction in solid electrolytes and the glass transition behavior of polymers [237]. The macroscopic long-range transport of ions is the result of the accumulation of countless such microscopic short-range jumps driven by chain segment motion. Therefore, the flexibility and mobility of the polymer chain segments directly determine the level of ionic conductivity. On a macroscopic level, this chain segment motion mechanism determines the ionic conductivity, mechanical properties, and electrochemical stability window of solid polymer electrolytes. It is worth noting that the movement of polymer chain segments is not completely random, but is regulated by multiple factors such as intermolecular interactions, crosslinking degree, ion concentration and additives [24]. By precisely controlling the molecular structure design, crosslinking density and ion salt concentration of the polymer, the synergistic effect between chain segment motion and ion transport can be optimized, thereby achieving a balance between high ionic conductivity and good mechanical strength. This is the core strategy for developing high-performance solid polymer electrolytes.

#### Classification of Solid Electrolytes

**Fig. 14:**
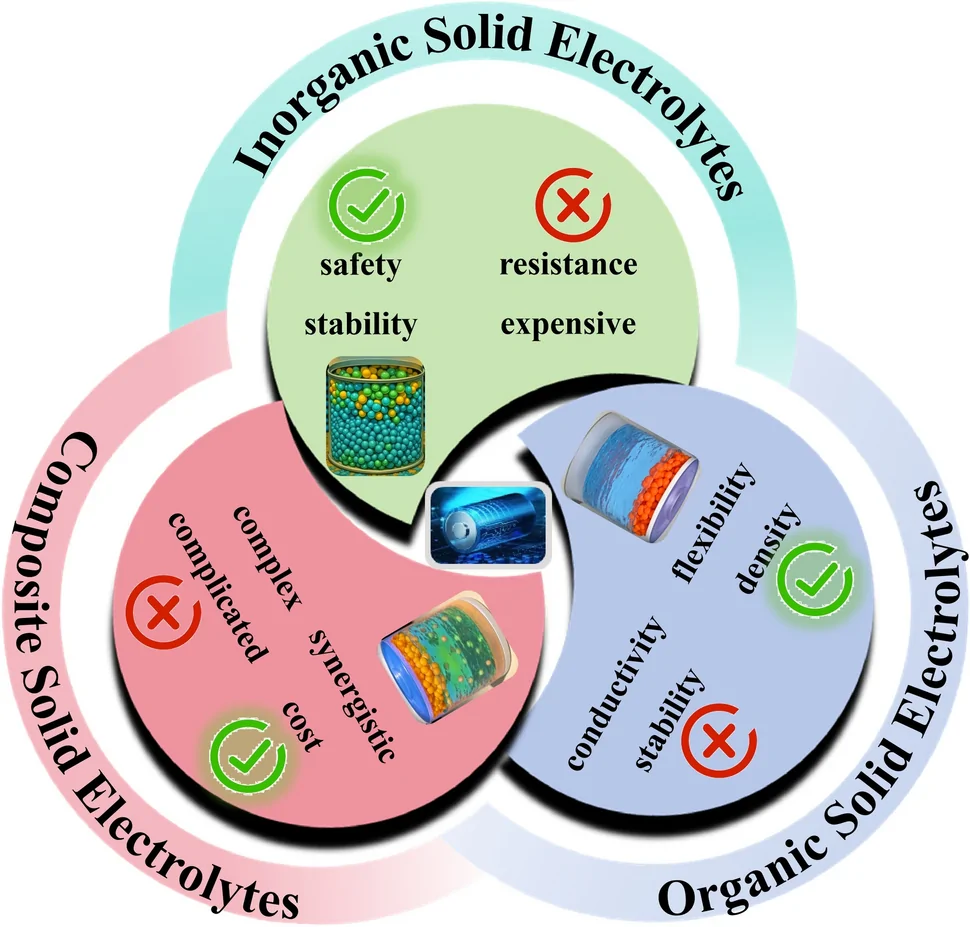
Schematic diagram of the three main classifications of solid electrolytes [238–245]. © The Author(s) 2023. © 2020 American Chemical Society. © 2018 Elsevier B.V. All rights reserved. © 2024 The Authors. Published by Elsevier Ltd. © 2021 Wiley–VCH GmbH. © 2024 Elsevier B.V. ©2020 American Chemical Society. © The Royal Society of Chemistry 2025

Currently, solid-state electrolytes are categorized into three types based on their host materials: inorganic, organic, and inorganic–organic composites. These three types of solid electrolytes are also being used in research on the adaptation of alloy-based anode materials for lithium-ion batteries. In order to clearly observe the various types of alloy-type solid electrolytes, the three most common solid electrolytes and their advantages and disadvantages are summarized in Fig. 14 [238–245].

Among them, inorganic solid electrolytes mainly include oxides, sulfides and halides. Typical representatives of oxide electrolytes are garnet-type Li_7_La_3_Zr_2_O_12_ (LLZO), NASICON-type Li_1+x_Al_x_Ti_2−x_(PO_4_)_3_ (LATP) and perovskite-type La_2/3−x_Li3_x_TiO_3_ (LLTO) [239]. The main advantages of oxide inorganic solid electrolytes are a wide electrochemical window (about 6.0 V), high elastic modulus and strong thermal stability. Ping et al. [246] used an LLZO garnet-type solid electrolyte to successfully prepare a silicon anode with a thickness of 1 µm, which is 10 times thicker than the thickness of silicon anodes in traditional organic electrolytes. The solid silicon anode exhibited excellent electrochemical performance, with a discharge capacity of 2685 mAh g^−1^ and a first coulombic efficiency of 83.2%. Through systematic morphological characterization and mechanical modeling analysis, the study found that the strong mechanical constraint of the garnet solid electrolyte can effectively limit the volume change of the silicon anode during the lithiation/delithiation process, preventing cracks and structural damage, so that the silicon anode can maintain structural integrity and good contact with the electrolyte. However, because the electrolyte is very rigid, the contact with the electrode is point-to-point contact, and the contact area remains small, making the interface impedance between the two large, resulting in the obstruction of lithium-ion transmission and a serious reduction in ionic conductivity. In addition, the synthesis of oxide electrolytes requires high temperature conditions, which has high energy requirements and production costs, making it difficult to further realize applications. As far as current research is concerned, typical representatives of sulfide electrolytes include glass-ceramic phase Li-P-S (Li_5_PS_4_, Li_7_P_3_Sn), Argyrodite-type Li_6_PS_5_X (X = Cl, Br, I), thio-LISICON Li_4_SnS_4_, and Li_11−x_M_2−x_P_1+x_S12 (M = Ge, Sn, Si) type Li_10_GeP_2_S_12_ [240]. Sulfide electrolytes are soft in texture, have a large contact area with the electrode, and have a small interfacial impedance, resulting in excellent ionic conductivity, which can reach the order of 10^−2^ S cm^−1^. It is currently the solid electrolyte that can best compare to liquid electrolytes in terms of ionic conductivity performance, and is also the most popular development direction for alloy-type lithium-ion batteries. However, the preparation conditions of sulfide electrolytes are harsh, their stability in air is poor, and they easily react with water to produce the highly toxic substance H_2_S. Therefore, large-scale production and application are difficult at this stage [241]. Park et al. [247] studied the performance of lithium-silicon alloy prepared by mechanical alloying as a anode material for all-solid-state lithium secondary batteries. The solid electrolyte selected was 70Li_2_S-30P_2_S_5_, and the anode used was a silicon-based anode material. In this study, they first mechanically alloyed lithium particles with silicon powder, and then further ball-milled the alloy powder to reduce the particle size. The experimental results showed that the alloy powder after the second ball milling had a smaller particle size and was evenly distributed, which significantly reduced the interfacial resistance of the battery and thus improved the electrochemical performance. In addition, it was found that reducing the particle size can increase the contact area between the electrode and the solid electrolyte and improve the alloying/de-alloying kinetics. In recent years, the development of halide solid electrolytes has received widespread attention due to their good ionic conductivity and wide electrochemical window. Common halide solid electrolytes include Li_3_YCl_6_, Li_3_YbCl_6_, and Li_3_YBr_6_, but halide electrolytes have obvious problems such as poor reduction stability and poor wet air stability [242]. They are still in the laboratory exploration stage and it is difficult to achieve their commercial application.

Compared with inorganic solid electrolytes, organic solid electrolytes exhibit good flexibility, simple preparation process, large contact area with active electrodes, and can adapt to volume expansion during cycling, therefore they have received widespread attention [243]. They are mostly polymeric compounds, where the common polymer solid electrolytes used are mainly the following: (1) PEO, which is the most common polymer solid electrolyte with good interfacial properties. For example, Zhang et al. [248] reported a silicon/vertical graphene (Si@VG) composite anode material for polymer-based all-solid-state batteries. Vertical graphene sheets were grown on the surface of silicon particles by thermal chemical vapor deposition. The material formed a three-dimensional conductive network, significantly improving the electronic conductivity of the electrode and alleviating the volume expansion problem of silicon during cycling. The flexible properties of the vertical graphene improved the interfacial contact between the anode and the solid polymer electrolyte (PEO-based) and reduced the interfacial impedance as well. In addition, this material had a higher lithium-ion diffusion coefficient and interface stability. (2) Polyvinylidene fluoride (PVDF), this polymer electrolyte has a strong electron-withdrawing effect, which can promote the transport of ions in the polymer electrolyte [243]. At present, many researchers have reduced the crystallinity of PVDF by introducing hexafluoropropylene (HFP), and the resulting PVDF-HFP and PVDF polymer electrolytes are widely used. For instance, Li et al. [249] designed a three-dimensional carbon interconnected micro-silicon (MSi-C) structure, which was prepared by carbonizing micro-silicon coated with PAN, in which the carbon network not only acted as a binder to maintain the integrity of the silicon film, but also provided an ion-electron conductive channel. (3) The advantage of PAN electrolyte is its strong antioxidant capacity, and it is often used to match high-voltage positive electrode materials. However, the interfacial compatibility between the cyanide group in PAN and the metal anode is poor, which seriously affects its application in the battery field [244].

Inorganic solid electrolytes have advantages such as high ionic conductivity, strong mechanical properties, and good thermal stability, but they also have disadvantages such as large interfacial impedance and brittleness. In contrast, polymer electrolytes have good flexibility and a wide electrochemical window, but they also have disadvantages, such as low ionic conductivity and poor mechanical properties at room temperature. At present, neither inorganic solid electrolytes nor polymer electrolytes can meet the practical application and production requirements of lithium metal batteries. Therefore, most current researchers use composite solid electrolytes that combine the two [245]. Composite solid electrolytes combine the high strength, high stability, and high ionic conductivity characteristics of inorganic solid electrolytes with the flexibility and easy processing advantages of polymer solid electrolytes through the synergistic effect of polymers and inorganic fillers. While significantly improving the ion transfer efficiency and electrochemical stability of the electrolyte, compositing also enhances its mechanical properties, making it better able to meet the needs of lithium metal batteries in practical applications. The addition of inorganic fillers to polymers not only reduces the crystallinity of the polymer, but also promotes the dissociation of lithium salts, thereby constructing an ion transfer network. Common inorganic fillers are divided into active fillers and inert fillers. Inert fillers include Al_2_O_3_, SiO_2_, and ZrO_2_, while active fillers include LLZO and LATP, etc. [238]. In addition, the interaction between polymer molecules can be weakened by adding plasticizers, thereby reducing the crystallinity of the polymer. Huo et al. [250] prepared a composite polymer electrolyte (PPCL-SPE) based on polypropylene carbonate (PPC) and garnet-type solid electrolyte (LLZTO) to construct a flexible interface between the silicon anode and the electrolyte to solve the problem of interface failure caused by the volume expansion of the silicon anode in all-solid-state batteries. The study confirmed through mechanical testing and electrochemical analysis that the flexible interface significantly improves interface stability and battery performance through the synergistic effect of the elastic deformation of the PPC matrix and the LLZTO filler, providing a new strategy for the application of silicon anodes in high-energy-density all-solid-state batteries.

### Challenges of Solid-State Electrolytes in Solid-State Lithium-Ion Battery

**Fig. 15:**
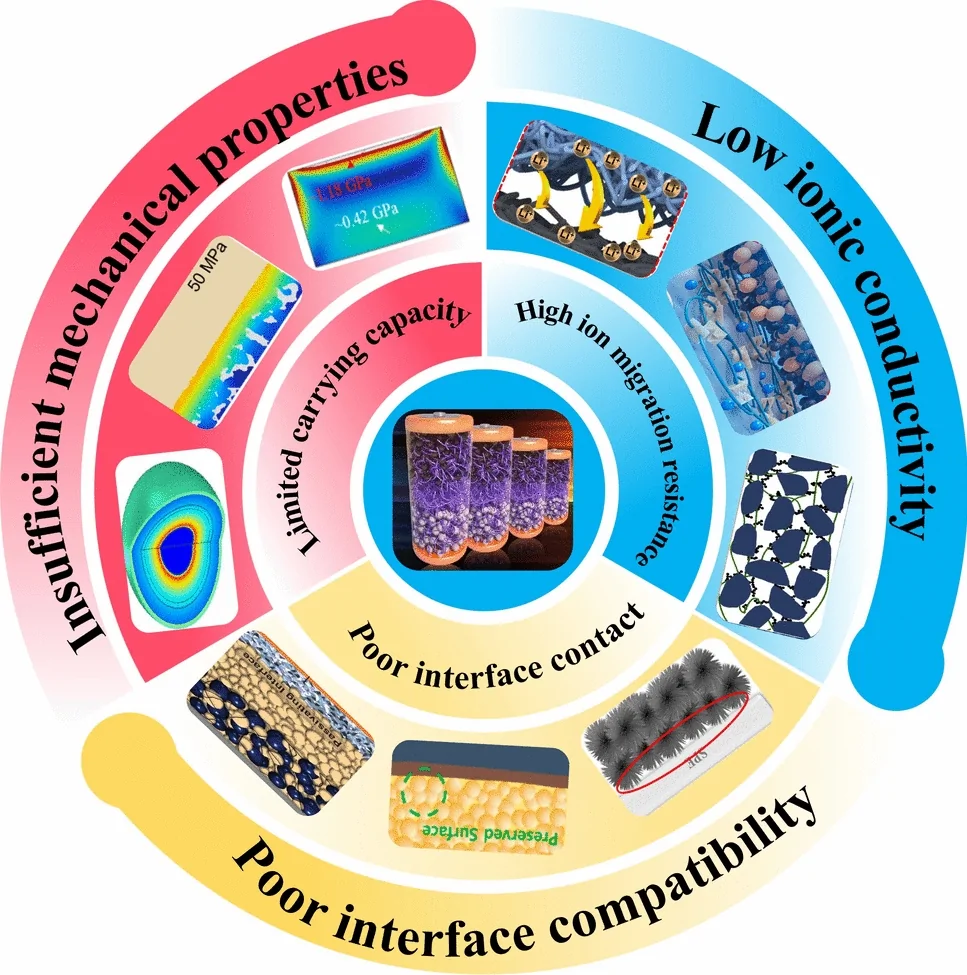
Schematic diagram of three main defects of solid electrolytes [248, 252–259]. © 2023 American Chemical Society. © 2021 The Authors. Published by Elsevier B.V. © 2025 Elsevier Inc. © 2024 Published by Elsevier Inc. © 2023 American Chemical Society 19. © 2021 American Association for the Advancement of Science. All rights reserved. © Youke Publishing Co., Ltd. 2023. © 2024 Elsevier B.V. All rights reserved. © 2023 Elsevier B.V. All rights reserved. © 2019 Elsevier B.V. All rights reserved

In the wave of next-generation battery technologies striving for higher energy density and improved safety, alloy-anode all-solid-state batteries hold great promise. However, solid-state electrolytes still face numerous challenges on the road to commercialization. Low ionic conductivity, inherent electrochemical instability, and stress-induced mechanical failure are currently recognized as key bottlenecks. Furthermore, the challenges of fabrication processes are also significant. Solid-state electrolytes must maintain high density while avoiding excessive grain boundary impedance, which requires precise control of sintering temperature, pressure, and atmosphere conditions. Different types of solid-state electrolytes require distinct fabrication routes. Cost control is also a significant concern. Many high-performance solid-state electrolyte materials suffer from high fabrication costs, scarce raw materials, or complex fabrication processes, hindering their commercial application. Furthermore, the assembly process for solid-state batteries differs significantly from that of traditional liquid-state batteries, requiring the development of novel battery manufacturing technologies and equipment, including electrode material pretreatment, interface modification, and interlayer composites. This requires significant time and resources to evaluate their practical application potential. In Fig. 15, we have systematically summarized the three main disadvantages of alloy-type anode solid electrolytes: insufficient ionic conductivity, poor interfacial compatibility, and weak mechanical properties [248, 252–259].

#### Low Ionic Conductivity

In alloy-type anode all-solid-state batteries, the low ionic conductivity of solid electrolytes is one of the serious challenges hindering their commercial application [259]. The root of this problem is that the inherent structural characteristics of solid materials limit the free migration ability of ions, which is specifically reflected in the fact that their ion transport efficiency is much lower than that of traditional liquid electrolytes. Compared with liquid electrolytes, where ions can move relatively freely and quickly under the solvation effect of solvent molecules, ion transport in solid electrolytes must rely on limited conduction paths in the solid matrix. These paths are often restricted by multiple factors such as grain boundaries, interfacial impedance, and space charge layers, resulting in their room temperature ionic conductivity being one to two orders of magnitude lower than that of liquid electrolytes [260]. Specifically, the reasons for the low conductivity of different types of solid electrolytes are different. For polymer solid electrolytes, the ion conduction mechanism mainly relies on the movement of polymer chain segments to provide conduction channels for ions, but the mobility of polymer chain segments at room temperature is limited, resulting in ionic conductivity usually only at the level of 10^−6^ to 10^−4^ S cm^−1^, which is much lower than the 10^−2^ to 10^−3^ S cm^−1^ of liquid electrolytes. For example, the ion transport capacity of polymers such as polyethylene oxide (PEO) at room temperature is much lower than that at high temperature. One study [261] explored the problem of low ionic conductivity of PEO-based solid polymer electrolytes at room temperature. It was found that due to its semi-crystalline structure, PEO limits the chain segment movement at room temperature, thereby inhibiting the migration of lithium ions, making its conductivity difficult to meet the needs of practical battery applications. However, under heating conditions, the crystal part of PEO gradually transforms into an amorphous state, and the activity of the polymer chain segment is significantly enhanced, thereby increasing the dissociation degree of lithium salts in it and the migration rate of ions, and the overall conductivity is significantly improved. The results show that PEO electrolytes exhibit good thermal stability and high ionic conductivity in the temperature range of 60–80 °C. In addition, the limited solubility of the polymer matrix for lithium salts and the strong coordination effect between lithium ions and polar groups on the polymer chain limit the concentration and migration rate of freely moving carriers. This low ionic conductivity directly leads to a significant increase in the internal resistance of the battery, limiting the rate performance and power density of the battery. What is more serious is that low ionic conductivity is often accompanied by uneven current distribution, which easily forms local current-intensive areas on the electrode surface and accelerates the degradation of the electrode material.

For inorganic solid electrolytes, there are specific ion-conduction channels in the internal crystal structure of certain oxides and sulfides, and sulfide-based electrolytes have been able to achieve ion conductivity levels close to those of liquid electrolytes [262]. However, the room temperature ion conductivity of most oxide solid electrolytes is also still 1–2 orders of magnitude lower than that of liquid electrolytes. In their case, it is mainly due to the fact that their rigid crystal structure limits the freedom of ion migration, and the high impedance at the grain boundaries further hinders ion transport. The grain boundaries that are prevalent in polycrystalline materials often become the main obstacle to ion transport. Zeng and team [196] constructed a 7 ± 2 μm thick garnet structure solid electrolyte LLZTO artificial interface layer on the surface of the silicon anode, and used the chemical cross-linking effect of PVA binder and PAA in the silicon electrode to form a stable ion channel, which significantly improved the interfacial ion conductivity. After 100 cycles, the interfacial impedance of the battery remained stable at around 100 Ω, which is much lower than the continuously increasing impedance of the untreated silicon electrode. The high ionic conductivity and high mechanical strength of LLZTO synergistically inhibited the volume expansion of silicon, reducing the expansion rate from 611% to 259%. Taking liquid batteries as a reference, this design in all-solid-state batteries further optimized the interface contact, reducing the impedance by 50%. After 1000 cycles at 1.2 A g^−1^, the capacity of 386 mAh g^−1^ was still maintained, confirming that the synergistic effect of the cross-linked binder and the LLZTO coating can stabilize the ion transport path and improve the electrochemical stability. It was also found that the atomic arrangement in the grain boundary region was chaotic and the structure was disordered, resulting in the grain boundary resistance being much higher than the grain body resistance, significantly reducing the overall ionic conductivity of the material. Although sulfide solid electrolytes have the highest room temperature ionic conductivity, where some can even be comparable to liquid electrolytes. However, they are extremely sensitive to air and moisture and are prone to react to generate low-conductivity byproducts, resulting in a decrease in conductivity. In addition, the interfacial chemical/electrochemical side reactions with the electrode materials also form an intermediate phase that hinders ion transport and increases the interfacial resistance [263]. The inherent low ionic conductivity directly leads to an increase in the internal resistance of the all-solid-state battery, which seriously affects the battery's rate performance and power density, making it difficult to meet the application requirements of fast charging and discharging. At the same time, in order to make up for the lack of ionic conductivity, it is necessary to increase the proportion of solid electrolyte in the electrode, which correspondingly reduces the content of active materials and thus reduces the overall energy density of the battery. Although oxide electrolytes have good stability: not being very sensitive to air and moisture and not prone to side reactions to produce low-conductivity byproducts either. However, their grain boundary impedance is still large, their texture is hard and brittle, and their solid-solid interface contact with the electrode is poor, which further increases the interface impedance. Therefore, how to effectively improve the room temperature ionic conductivity of solid electrolytes and improve their interface problems is a core scientific and technological challenge that needs to be solved in the current research field of all-solid-state batteries. Researchers have adopted a variety of strategies, including improving the intrinsic ionic conductivity of materials through doping modification, optimizing microstructure to reduce grain boundary impedance, constructing composite electrolyte systems that combine the advantages of different materials, and developing new ion conduction mechanisms. However, these improvement methods must significantly improve the ionic conductivity while maintaining the mechanical strength and chemical stability of solid electrolytes, which is still the core scientific and engineering challenge facing the current development of solid-state battery technology.

#### Intrinsic Electrochemical Instability

The interface between alloy anodes and solid electrolytes is a core technical bottleneck hindering the development of solid-state battery technology. Its complexity stems from the physical, chemical, and electrochemical interactions at the solid-solid interface, which far exceed those found in traditional liquid-based batteries. Specifically, solid materials cannot fully wet the alloy electrode surface and fill the porous structure of the electrode material like liquid electrolytes do, resulting in a significant lack of solid-solid interface contact area.

At the microscopic level, even the surface of a precisely processed solid material still has nanoscale roughness and unevenness. When a solid electrolyte contacts an alloy electrode material, the actual contact area is often only a small fraction of the nominal contact area, and there are microscopic gaps in a large area. These gaps become the main obstacle in the ion transport process, forcing ions to be transferred only through a limited number of real contact points, thus forming a significant "impedance bottleneck" at the interface. The generation of interface impedance involves multiple mechanisms such as geometric impedance, chemical impedance, and charge layer impedance: where, specifically, geometric impedance originates from the current congestion effect caused by the reduction of contact area [264]; chemical impedance comes from the influence of the sudden change of the chemical environment at the interface of different materials on the activation energy of ion transport [12]; and charge layer impedance is due to the formation of a space charge layer, which further changes the local electric field distribution and increases the difficulty of ion transport. Wang et al. [265] used in situ differential phase contrast scanning transmission electron microscopy (DPC-STEM) technology to realize the in situ visualization study of the space charge layer effect in all-solid-state lithium-ion batteries for the first time. The study found that at the interface between high-voltage LCO and the sulfide solid electrolyte LPSCI, lithium ions are enriched on the cathode side and depleted on the electrolyte side, forming a space charge layer that hinders lithium-ion transport. This is one of the main reasons for the slow interfacial ion transport. To address this problem, the authors proposed a strategy of coupling built-in electric field with chemical potential. By coating discontinuous ferroelectric BaTiO_3_ nanoparticles on the LCO surface, they successfully suppressed the formation of the space charge layer and promoted the redistribution of lithium ions at the interface, thereby significantly reducing the interfacial impedance and improving the battery's rate performance. The modified BTO-LCO cathode exhibited a discharge capacity of nearly 140 mAh g^−1^ at 0.2 C and a capacity of 92 mAh g^−1^ at a high rate of 1 C, far exceeding the discharge capacity of 60 mAh g^−1^ of the unmodified LCO cathode.

In addition, from a physical perspective, both solid electrolytes and alloy electrodes are rigid materials. The inevitable roughness between the surfaces makes it difficult for the two to achieve a perfect atomic-level fit, resulting in the actual contact between the two often being discrete point contacts [266]. This greatly limits the effective ion transfer area and generates huge interface impedance. More seriously, during the battery charge and discharge cycle, the electrode material undergoes significant expansion and contraction. This repeated volume change continues to destroy the already fragile physical contact. Generally, this repeated expansion and contraction process is anisotropic and may cause interface separation, the formation of holes or cracks, and thus the continuous accumulation of interface impedance and the rapid decline of battery performance. Temperature changes in the battery further exacerbate the complexity of this problem. Due to the difference in thermal expansion coefficients between different materials, temperature fluctuations generate thermal stress at the interface, which may lead to interface delamination or even cracking. At the same time, at high temperatures, the interface chemical reaction is accelerated to form a high-impedance reaction layer, while the hardness and brittleness of the material at low temperatures make interface contact more difficult. In sulfide solid electrolytes, the carbon additive content has an important influence on the interface properties. An appropriate amount of carbon additives can significantly improve the contact between the electrolyte and the electrode, reduce the interfacial impedance, and improve the ion conduction efficiency [267]. The conductivity of carbon materials helps to build an electronic conduction network and reduce the charge accumulation at the interface. However, excessive carbon additives may react adversely with the sulfide electrolyte, forming byproducts or causing a decrease in interfacial stability. In addition, the dispersion uniformity of carbon additives directly affects the consistency of the interface. Uneven distribution will lead to uneven local current density and produce hot spot effects. Tan and group explored the use of sulfide solid electrolytes to achieve stable cycling performance of high-loaded micron silicon anodes in lithium-ion batteries [255]. The study found that carbon additives significantly accelerated the decomposition of solid electrolytes, so a 99.9 wt% carbon-free micron silicon anode was used to effectively reduce interfacial side reactions. The SEI formed at the interface between the solid electrolyte and micron silicon has passivation properties and can inhibit continuous interfacial growth and lithium loss. In addition, micron silicon exhibits unique chemical and mechanical behaviors during charging and discharging, forming a dense lithium-silicon alloy during lithiation and maintaining contact after delithiation, thereby maintaining good interfacial stability. In terms of electrochemistry, the full cell achieves a high current density of 5 mA cm^−2^ at room temperature, a wide operating temperature range from − 20 to 80 °C, and a high areal capacity of 11 mAh cm^−2^, with 80% capacity retention after 500 cycles. These excellent performances are attributed to the interfacial passivation effect of SSE and the unique mechanical behavior of lithium-silicon alloys.

At the same time, at the chemical and electrochemical level, the interface is a highly active reaction area, and its stability directly determines the life and safety of the battery [268]. On the one hand, the electrochemical window of many solid electrolytes is narrow, and redox side reactions will occur when they come into contact with high-potential positive electrodes or low-potential anodes, generating an intermediate phase layer with complex chemical composition, usually with low ionic conductivity, and possibly high electronic conductivity. This unstable interface layer continuously consumes active materials and electrolytes and become another obstacle to lithium ion migration. On the other hand, the space charge layer formed by the redistribution of interfacial charges due to the chemical potential difference, when the electrode and the electrolyte are in contact, further increases the interfacial resistance and hinders the smooth passage of lithium ions. Klerk et al. [269] explored the effect of the space charge layer in all-solid-state batteries and evaluated its importance to battery performance. The research team analyzed the space charge layer characteristics at the interface between LCO and graphite electrodes and LLZO and LATP solid electrolytes by establishing a simple model that takes into account the Coulomb interaction between defects. Research has found that the thickness of the space charge layer is typically at the nanometer level, and the interfacial resistance and capacitance it causes have negligible effects on battery performance, unless a region of completely depleted lithium ions forms in the solid electrolyte. Model results indicate that the resistance of the space charge layer is less than 1 Ω cm^2^ in most cases, and only increases significantly under extreme conditions when lithium ions are completely depleted. To address the multi-scale and multi-physics coupling challenge of solid electrolyte interfacial contact, which combines physical contact, chemical stability, and electrochemical reactions, although solutions such as interface modification, buffer layer design, and composite electrolytes can alleviate the problem to a certain extent, it is often difficult to simultaneously address the challenges of both poor initial contact and deteriorating dynamic evolution. Therefore, continuing to build efficient and stable solid-solid interfaces through interface engineering, material innovation, and advanced manufacturing technologies is key to moving high-performance, high-safety all-solid-state batteries from the laboratory to practical application.

#### Mechanical Failure Caused by Stress

The high mechanical performance requirements of solid electrolytes stem from their dual functions of ion conduction and mechanical support in alloy-type solid-state batteries. The requirements for their mechanical properties go far beyond simply pursuing high hardness, but rather aim to achieve a delicate balance between rigidity, toughness, and flexibility. This multifunctional integration places extremely demanding and often contradictory demands on the mechanical properties of the material [270].

First, the solid electrolyte must have sufficient mechanical strength to effectively inhibit the tip of the lithium dendrite from penetrating the electrolyte, which is one of the core safety advantages of solid-state batteries. In addition, the solid electrolyte can also inhibit the expansion of the alloy anode. Studies have shown that the stress generated by the alloy anode when fully lithiated can reach several gigapascals or even tens of gigapascals. In this case, the solid electrolyte must have the corresponding mechanical strength and toughness to resist this stress without cracking or deforming [271]. Compared with liquid electrolytes, the solid-solid interface formed by the solid electrolyte and the electrode can provide uniform mechanical support, effectively limiting the expansion deformation of the alloy anode. However, although traditional inorganic solid electrolytes such as oxides and sulfide materials have good ionic conductivity, they often show brittle characteristics and are prone to brittle fractures when subjected to impact or stress concentration. Chen and team used the reaction molecular dynamics (ReaxFF-MD) method to systematically study the structural evolution and stress change mechanism of crystalline silicon and amorphous silicon during lithiation [272]. Stress analysis shows that crystalline silicon accumulates compressive stress of up to about 6 GPa at the lithium-silicon phase boundary, hindering the phase boundary from moving forward, thereby slowing down subsequent lithiation. Amorphous silicon can relieve stress through structural deformation and achieve rapid lithiation. Radial distribution function and coordination number analysis show that crystalline silicon generates more ordered Si clusters and lithium-silicon structures during lithiation, while amorphous silicon generates fewer clusters and more disordered structures. Gao et al. [273] constructed a Li|Li_7_La_3_Zr_2_O_12_ (LLZO) battery and observed the dynamic interface evolution process caused by lithium deposition, revealing the failure mechanism of LLZO during lithium deposition. This study found that under strong mechanical constraints and low current density, lithium can expand laterally on the LLZO surface and maintain a single crystal structure, exhibiting plastic creep behavior. However, at high current density (such as 3–4 A cm^−1^), local rapid deposition causes the interface stress to increase suddenly to the order of GPa or even 10 GPa, which is enough to crack the single-crystal LLZO without obvious defects, forming cracks, and ultimately causing short circuit. This "lithium eruption" deposition mode has been proven to be the root cause of electrochemical-mechanical coupling failure. By comparison, LLZO can remain intact even when deposited at high current under weak mechanical constraints, indicating that the stress release method is the key.

However, the pursuit of extreme rigidity leads to a second key requirement that conflicts with it: high fracture toughness or deformation capacity. High hardness does not mean good toughness. For example, many ceramic electrolytes are hard but as brittle as glass and easily crack under stress [274]. Any tiny defects inside the battery, uneven current distribution or defects introduced during the manufacturing process may become the starting point of cracks. Once the cracks begin to expand, they quickly penetrate the entire electrolyte layer, causing an internal short circuit in the battery. For example, during the actual charging and discharging process of the battery, the electrode materials, especially high-capacity metal lithium, silicon or tin-based anode materials, undergo drastic volume changes [275]. This requires the solid electrolyte to be able to follow the deformation of the electrode material while maintaining structural integrity. Otherwise, stress concentration will occur at the interface, leading to delamination or cracking, which directly damages the ion transport channel and may cause a series of subsequent safety problems. If the solid electrolyte is a completely rigid brittle material, it will not be able to adapt to this volume change of the electrode, resulting in huge stress concentration at the interface. This stress can cause interface separation, microcracks in the electrolyte itself, or even fracture, ultimately leading to loss of physical contact, a sharp increase in interface resistance, and loss of active materials, that is, the formation of dead lithium, which causes rapid deterioration of battery performance or even complete failure. Therefore, high toughness and deformability ensure that the electrolyte is not easily broken when subjected to internal stress, ensuring the long-term structural integrity and safety of the battery. However, for brittle ceramic materials, manufacturing large-area, defect-free films is a huge technical challenge, with high costs and low yields. Kalnaus et al. [214] took oxide-type solid electrolytes such as LLZO as an example to analyze the factors affecting the microstructure density during the sintering process, including temperature, pressure, and the precursor composition of the material, and pointed out that the thickness of the solid electrolyte film needs to be less than 10 microns to achieve high performance. The authors believe that insufficient density or improper sintering conditions can easily lead to the formation of grain boundary defects and microcracks, thereby reducing the mechanical strength and ionic conductivity of the electrolyte, and further exacerbating the risk of interface failure and lithium dendrite penetration during battery cycling. To make matters more complicated, the mechanical properties of solid electrolytes need to remain stable over a wide temperature range. At low temperatures, the material may become too rigid and lose the necessary deformation capacity, while at high temperatures, it may soften and be unable to effectively inhibit dendrite growth. This temperature dependence makes material design more difficult. Finally, solid electrolytes are also faced with complex loading conditions of multi-directional stress during manufacturing and use, including compression stress during manufacturing, assembly stress, and alternating stress caused by temperature cycling and electrochemical cycling during use [213, 276]. These complex stress states require the material to have not only good static mechanical properties, but also excellent fatigue resistance and long-term creep resistance.

In summary, it needs to have sufficient rigidity to resist the penetration of lithium dendrites, and sufficient flexibility and toughness to adapt to the volume changes of the electrode, maintain interface stability and withstand internal stress, while also being easy to process and shape. This also explains why current research focuses on the development of organic-inorganic composite electrolytes, attempting to combine the rigidity of ceramics and the flexibility of polymers in order to find the best balance between these mutually constrained performance requirements, thereby promoting the realization of safe, long-life, high-energy-density all-solid-state batteries.

### Modification Strategies of Solid-State Electrolytes in Solid-State Lithium-Ion Battery

#### Construction of Ion-Conducting Network

Solid-state electrolytes, as the core component of alloy-anode all-solid-state batteries, have diverse and evolving improvement strategies, primarily targeting challenges such as low ionic conductivity, unstable interfaces, and insufficient mechanical properties. These strategies are implemented through a multi-faceted approach, encompassing material design, interface engineering, and performance enhancement, aiming to achieve high energy density, safety, and long cycle life in battery applications. Firstly, to address the issue of low ionic conductivity, solid-state electrolytes can be categorized into four main types of materials design: oxide-based, sulfide-based, polymer-based, and thin-film.

##### Sulfide-Based Solid Electrolytes

Sulfide-based solid electrolytes, as key materials for the next generation of all-solid-state lithium-ion batteries, have attracted much attention due to their unique structural characteristics and excellent electrochemical properties. Sulfide solid electrolytes originate from oxide solid electrolytes and are formed by replacing oxygen ions with sulfur ions. Due to the low electronegativity of sulfur, the binding strength between sulfur and lithium ions is less than that between oxygen and lithium ions, which may lead to more freely moving lithium ions. This fundamental structural difference gives sulfide electrolytes excellent ion conductivity. Their excellent ionic conductivity and most favorable mechanical properties make them one of the most promising candidate materials for all-solid-state lithium metal batteries [277]. The most representative sulfide-based solid electrolytes are Li_3_PS_4_ (LPS), Li_6_PS_5_Cl (LPSC) and Li_10_GeP_2_S_12_ (LGPS) series electrolytes. LGPS is the first sulfide electrolyte to exhibit extremely high ionic conductivity, comparable to liquid organic electrolytes. This breakthrough achievement has laid an important foundation for the development of sulfide electrolytes [278]. However, the excessive sensitivity of sulfide solid-state electrolytes to moisture requires a processing environment that is incompatible with today's manufacturing infrastructure, which becomes one of the major technical obstacles to their large-scale industrial application.

**Fig. 16:**
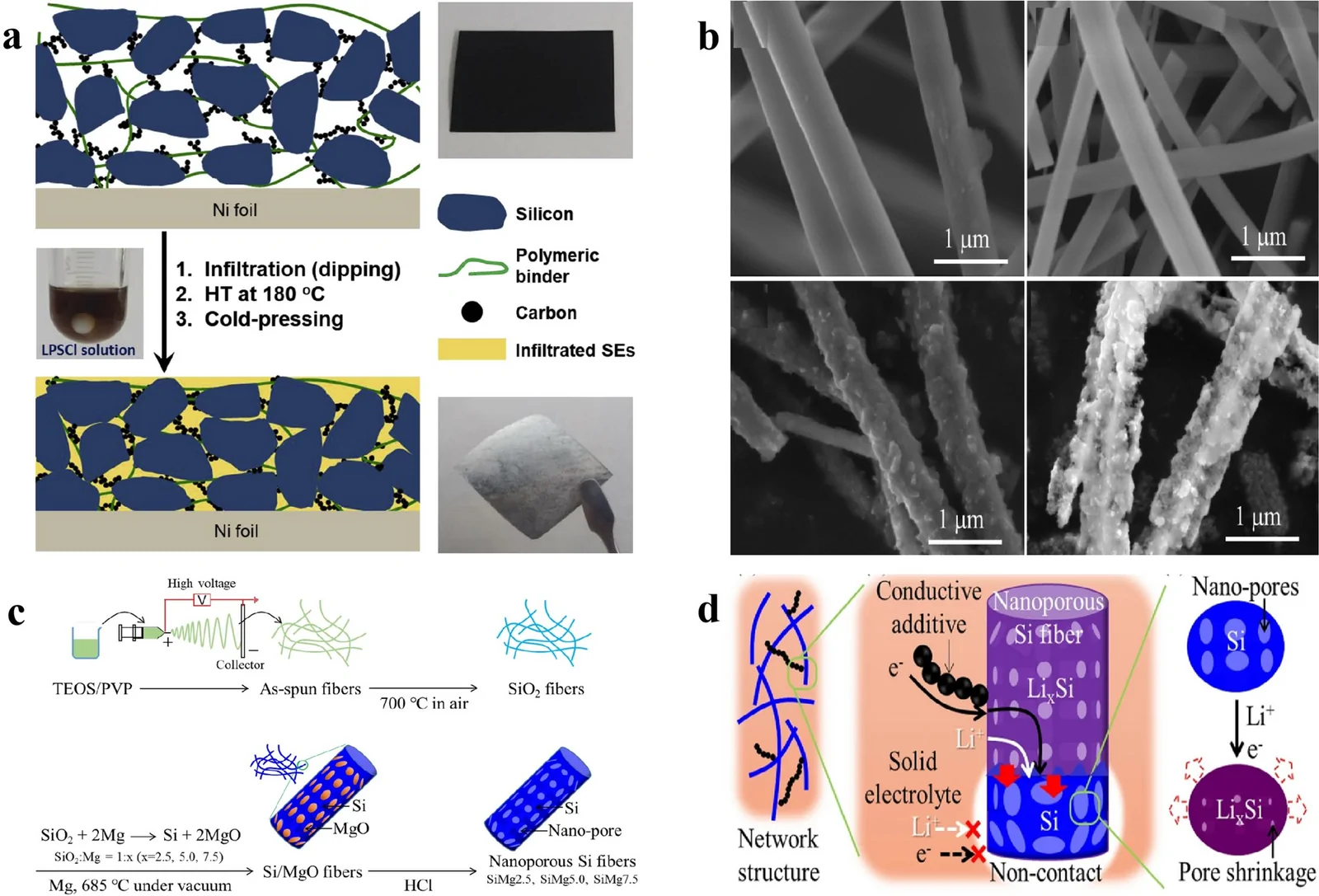
**a** A diagram depicting the infiltration method for traditional silicon composite electrodes using soluble solid electrolytes. Images capture the micro-silicon electrodes prior to and following the application of LPSCl, along with a view of the LPSCl solution in ethanol [280]. © 2019 Elsevier B.V. All rights reserved. **b** SEM images of as-spun fibers, the corresponding SiO_2_ fibers after calcination in air, SiMg5.0 before HCl etching, and the final SiMg5.0 product. **c** Diagram depicting the fabrication procedure for nanoporous silicon fibers. **d** Diagram outlining the approach to ensure consistent cycling performance [281]. © The Author(s) 2023

LPSCl was selected for its excellent ionic conductivity and mechanical deformability. It forms a two-dimensional ionic contact by cold pressing or solution processing, avoiding the flammability risk of liquid electrolytes. The ionic conduction mechanism of this electrolyte is derived from the disordered arrangement of anions and chlorine doping in its quartz structure, which reduces the lithium-ion migration barrier and promotes rapid lithium ion diffusion. Kim's research team developed a double-layered hybrid electrode consisting of a composite electrode layer (close to the solid electrolyte layer) and a diffusion-dependent electrode layer (close to the current collector) [251]. It presents 3D-modeled electrode configurations at 4 mA h cm areal capacity alongside the computed specific contact area, defined as the contact surface normalized by total graphite volume. Among all electrode architectures examined, the composite design achieved the maximum specific contact area of 1.92 × 10^5^ m^2^ m^−3^. It proposes the schematic representation of structural features and ion transport processes in composite, diffusion-controlled, and hybrid electrode systems. The synergistic effect of the two lithium ion transport mechanisms of ionic conduction of solid electrolyte particles and diffusion between active material particles is fully utilized. Li_6_PS_5_Cl is mixed with active materials by cold pressing to form a permeable ion network, which improves the lithium-ion transport efficiency. In the impedance analysis, the composite electrode shows a slope change in the mid-frequency range, indicating that the ionic impedance inside the electrode is 1035 Ω. In contrast, the hybrid electrode, due to its unique double-layered structure, only allows ionic conduction in the composite part, so it shows a lower impedance value of 522 Ω. The low ionic impedance is achieved by the relatively small thickness of the composite electrode part and the optimized solid electrolyte distribution. In addition, the contact area between graphite and LPSCl particles in different electrode structures was analyzed by 3D modeling, and it was found that the composite electrode had the largest specific contact area of 1.92 × 10^5^ m^2^ m^−3^. Therefore, the hybrid electrode also achieved better electrochemical performance. Electrochemical performance tests showed that the graphite-based hybrid electrode achieved a surface capacity of 4.12 and 2.44 mAh cm^−2^ at 0.1 and 1 C rates, and a volume capacity of 503 and 298 mAh cm^−3^, respectively, showing excellent rate performance and reasonable energy density. Compared with traditional dry-mixed electrodes, the solution infiltration method significantly improved the ionic conductivity. The ethanol solution containing LPSCl evenly penetrates into the pores of the alloy electrode to form a permeable ion network, reducing particle agglomeration and poor contact in the dry mix, resulting in a shorter ion transmission path and lower interface resistance [279, 280]. Unlike other researchers who directly applied electrolyte powder to battery preparation, Kim et al. [259] first dispersed the solid electrolyte into an ethanol solution and then infiltrated the solution into the silicon composite electrode to achieve scalable sheet electrode preparation to improve battery safety and energy density (Fig. 16a). In addition, this infiltration strategy optimized the ion contact area by liquefying LPSCl. The infiltration of LPSCl also broadened the electrochemical window, supporting compatibility with high-capacity silicon anodes and achieving an energy density of 338 Wh kg^−1^ for the LCO/Si full battery. The team members systematically investigated the factors affecting conductivity and performance: silicon particle size, polymer binder, and external pressure. The final experiment showed that micron-sized silicon and PVDF binder showed the best performance at 20 MPa and the least capacity decay. There are three reasons for this conclusion: although nano-sized silicon has a higher surface area, it is easy to agglomerate and increase resistance. The flexibility of PVDF alleviates the loss of ion contact caused by silicon volume expansion, while the rigidity of PAA/CMC leads to increased cracks and reduced conductivity. When the pressure dropped to 5 MPa, the performance decayed significantly, indicating that external pressure helps maintain close ionic contact, but excessive pressure may destroy the electrolyte structure.

In order to comprehensively solve the three major disadvantages of alloy-type anode solid electrolytes, each research team may not solve a single problem, but solve two or even three of them in a comprehensive way. In order to overcome the problems of low ionic conductivity and unstable interface at the same time, Yamamoto et al. [281] explored the important role of 75Li_2_S-25P_2_S_5_ in alloy-type anode solid electrolytes. The study showed that sulfide-based solid electrolytes can form stable and passivated SEI, which is a significant advantage over liquid electrolytes. In terms of strategies to increase conductivity, the study proposed an innovative network structure concept. The porous structure alleviated the volume change of silicon by shrinking the pores, thereby maintaining good interface contact between silicon and solid electrolytes or conductive additives. The fiber structure can easily form a network structure in the composite anode, and the partially formed Li_x_Si can continue to propagate ions and electrons to the partially delaminated area between the silicon fiber and SE or conductive additive, thereby improving the utilization of silicon. Figure 16b shows representative field emission scanning electron microscopy (FE-SEM) images of the products at each stage. The core of this design concept is that even when the contact between the silicon fiber and SE/conductive additive is partially disconnected, the partially lithiated silicon can still assist lithium-ion diffusion and electron transport through the fiber structure, thereby improving the utilization of silicon and increasing the initial coulombic efficiency. From Fig. 16c through a carefully designed nanoporous silicon fiber network structure, the ionic conductivity of the sulfide-based solid electrolyte is effectively utilized to construct and maintain ionic and electronic conductive pathways, ultimately achieving excellent electrochemical performance: an initial Coulombic efficiency of 71%, a reversible capacity of 1474 mAh g^−1^, a capacity retention of 85% after 40 cycles, and an areal capacity of 1.3 mAh cm^−2^, an industrially acceptable level. According to Fig. 16d, a new strategy for efficiently constructing and maintaining ionic and electronic pathways throughout lithiated silicon electrodes using porous and fibrous network structures is now clearly demonstrated. Furthermore, the mechanical properties of the solid electrolyte significantly influence its conductivity. Sulfide-based solid electrolytes exhibit a certain degree of elastic deformation, adapting to the volume changes of the silicon material during charge and discharge. Even with small outward volume changes of the fiber, the sulfide-based solid electrolyte can still deform elastically to a certain extent, maintaining close interfacial contact. This mechanical adaptability is crucial for maintaining stable ionic conductive pathways, especially when the silicon material undergoes large volume changes. During cycling, the charge transfer resistance of the solid electrolyte layer increases, but nanoporous silicon fibers exhibit a lower resistance growth rate than non-porous silicon. Compared to the resistance values before cycling, the resistance growth rate of SiMg 5.0 after cycling is significantly lower than that of the non-porous silicon. This difference suggests that the nanoporous silicon fibers are able to maintain close contact with the surrounding solid electrolyte, while non-porous silicon cannot do so. Although low ionic conductivity is the main challenge facing solid electrolytes, when its root cause is analyzed in depth, it is found that this problem is closely related to the microstructure and mechanical properties of the material. Improving ionic conductivity often requires increasing the porosity of the material, optimizing the grain boundary structure, or introducing defects to achieve more ion conduction channels. However, these improvement measures usually come at the expense of the mechanical strength of the material [282]. What is more complicated is that in the actual battery working environment, the solid electrolyte not only has to assume the function of ion conduction, but also must act as a physical barrier to prevent the penetration and growth of lithium dendrites. It creates an essential contradiction: the structural features that improve ionic conductivity often reduce the material's ability to resist mechanical stress. Conventional solid-state batteries usually require high pressures of tens or even hundreds of MPa to maintain good contact between the electrode material and the solid electrolyte to prevent the formation of internal voids and ion transport blockage caused by volume changes. However, this high-pressure requirement greatly increases the system complexity and cost, and reduces the energy density.

Different from traditional experimental methods, researchers successfully introduced elastic electrolytes into the porous electrode through vacuum infiltration and UV polymerization processes, so that they surrounded the active material particles and formed fast ion transport channels, and finally prepared solid-state batteries with excellent electrochemical performance. Pan and team first synthesized a soft-hard dimonomer copolymer using DMAM as the soft phase and AM as the hard phase, and then formed a deep eutectic mixture (DEM) by combining NMA and LiFSI in a 4:1 molar ratio [283]. Finally, an elastic solid electrolyte was developed from the two prepared substances. The DEM was selected because of its non-toxicity, high ionic conductivity, non-flammability and low vapor pressure, which was superior to conventional organic electrolytes. Raman spectroscopy confirmed that LiFSI was highly dissociated, with a strong free FSI^−^ anion peak at 731 cm^−1^ and a weak contact ion pair peak at 744 cm^−1^, which was not affected by dissociation after copolymerization. This elastic electrolyte exhibited a high ionic conductivity of 2 × 10^−3^ S cm^−1^, a lithium-ion transference number of 0.44, a high oxidation potential of 4.5 V, as well as non-flammability and self-healing properties at room temperature. It also had excellent mechanical properties, including an elongation at break of 1160%, a fracture strength of 1.7 MPa, shape memory ability and significant energy dissipation characteristics. Thanks to the excellent deformation recovery ability of the elastic electrolyte, the silicon material still maintained the integrity of the electrode after repeated expansion and contraction. Experimental results showed that the micronized silicon anode achieved a capacity retention rate of 90.8% after 300 cycles. The full battery and LFP achieved a 98.3% retention rate after 100 cycles. The assembled soft-pack battery could light up an LED, highlighting the synergistic effect of mechanical and ionic transport on increasing effective conductivity by preserving contact.

**Fig. 17:**
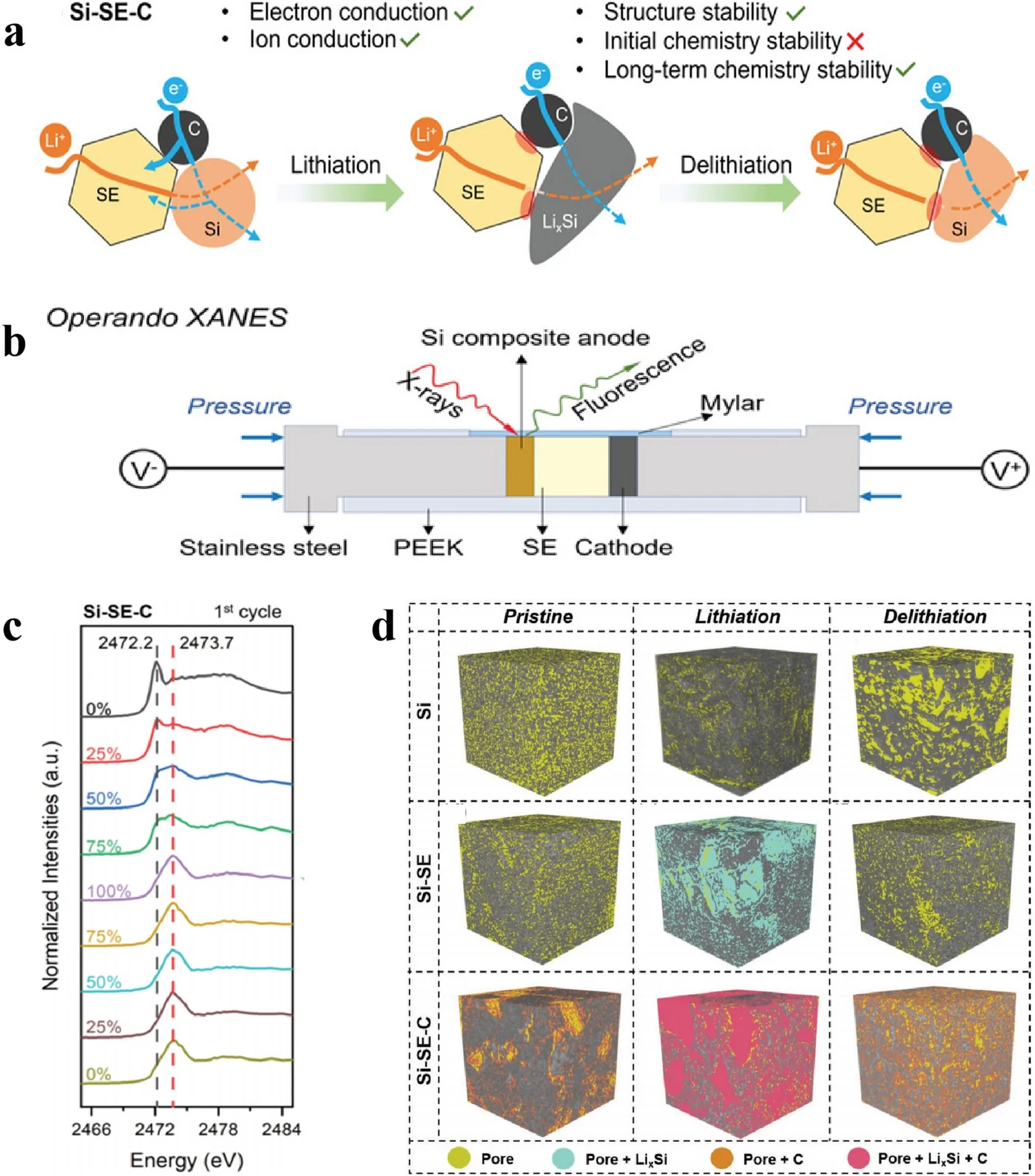
**a** Schematic illustrating the chemistry and structure evolution of Si–SE–C. **b** Schematic of the operando XANES. **c** Real-time assessment of the chemical durability of solid electrolytes within silicon composite anodes. **d** Analysis of structural changes using ex situ X-ray nanotomography [285]. © 2023 Wiley–VCH GmbH

Research on sulfides generally emphasizes optimizing the electrode-electrolyte interface contact to improve ionic conductivity, such as hybrid electrode design, solution penetration or microstructure control [284]. In addition, most researchers also enhance the electron and ion transport paths by adding conductive agents or binders, which reduce the interface resistance, and enhance the conductivity of the battery. For instance, Cao et al. [285] conducted an in-depth study on the application of sulfide solid electrolytes in silicon-based all-solid-state lithium batteries and their effects on ionic conductivity (Fig. 17a). The sulfide solid electrolyte Li_5.4_PS_4.4_Cl_1.6_ used in the study is a typical silver sulfide type sulfide electrolyte with an excellent ionic conductivity of up to about 8 mS cm^−1^. This value is far higher than the standard of 1 mS cm^−1^ at room temperature, making it an ideal choice for all-solid-state battery applications. The researchers adopted the strategy of adding solid electrolyte and carbon to silicon at the same time, which can be verified in detail by the results of electrochemical impedance spectroscopy analysis. In Fig. 17d, the team constructed a three-dimensional cubic model with a size of 10 × 10 × 10 µm^3^ for each reconstructed sample and studied the structural evolution through ex-situ XnT to track the structural evolution process. Before the battery test, the Si-SE-C composite anode showed the lowest impedance value, the pure silicon anode had a higher impedance, and the Si-SE composite anode showed the highest impedance. The Si-SE-C composite anode exhibited minimal overall impedance primarily due to the synergistic effect of carbon and the solid electrolyte. Si-SE exhibited a higher impedance compared to pure silicon because the solid electrolyte had a much lower conductivity than the silicon material itself. Equivalent circuit fitting analysis revealed that the impedance of various electrodes remains relatively low in the fully lithiated state, indicating that the decomposition of the solid electrolyte hade a negligible effect on the interfacial impedance and that the decomposition products retained good ionic conductivity. In addition, the X-ray absorption near-edge structure (XANES) spectrum showed no significant changes during the delithiation process, proving that the decomposition of the solid electrolyte was irreversible, but the decomposition products maintained relatively stable ionic conductivity during the cycle (Fig. 17b). Figure 17c shows the volume expansion and contraction of different anode materials during lithiation and the volume average of the effective plastic strain. This is the structural basis for the excellent electrochemical performance. From an electrochemical perspective, the addition of the solid electrolyte significantly improves the utilization and reaction kinetics of the silicon anode. The Si-SE-C composite anode achieved a maximum discharge/charge capacity of 3288/2917 mAh g^−1^ at a current density of 0.1 mA cm^−2^, with an initial Coulombic efficiency of 88.7%. This was primarily attributed to enhanced reaction kinetics and improved ionic conductivity. Notably, in situ XANES spectroscopy revealed that the sulfide solid electrolyte underwent partial electrochemical decomposition during the initial lithiation process. Theoretically, the final decomposition products were Li_2_S, Li_3_P, and LiCl, but this decomposition actually formed an ionically conductive passivation layer. Although this passivation layer created a certain resistance to ion and electron conduction, it also inhibited further degradation of the solid electrolyte, ensuring chemical stability during subsequent cycles. More importantly, the decomposed medium still had ionic conductivity.

##### Polymers-Based Solid Electrolytes

**Fig. 18:**
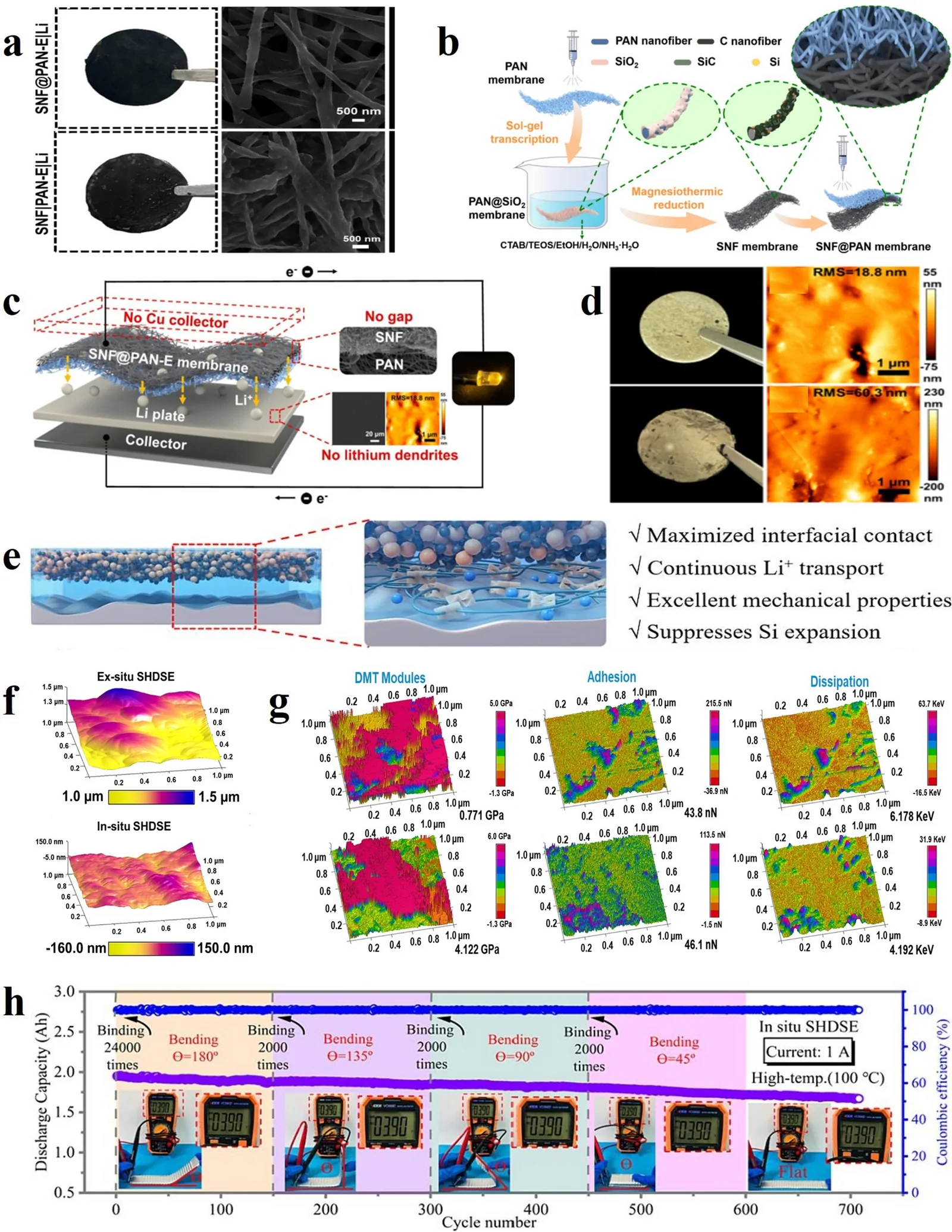
**a** Optical photos and FE-SEM images of SNF films. **b** Diagram of the manufacturing procedure for the integrated SNF@PAN. **c** Electron transport pathway of SNF@PAN. **d** Visual photographs and AFM images of lithium tablets [252]. © 2025 Elsevier Inc. **e** Structure of an integrated anode and polymer electrolyte for ASSLIBs, produced through in situ SHDSE. **f** AFM images depicting of the ex situ SHDSE/Si electrode and its in situ version after 100 h cycles. **g** DMT modulus mappings, adhesion mappings and energy-dissipation mappings of ex(in) situ SHDSE/Si electrodes. **h** Electrochemical cycling behavior of an in situ SHDSE Si|LiCoO_2_ pouch cell [253]. © 2024 Published by Elsevier Inc

Polymers such as PEO, PAN and PVDF can be formed into composite polymer electrolytes by adding inorganic fillers such as Al_2_O_3_, SiO_2_ or LLZTO, which significantly improves the lithium ion transference number and reduces the crystallinity to improve flexibility and ionic conductivity [238]. At the same time, cross-linked networks such as PEGDA and PC nanofiber structures can also introduce to enhance the mechanical modulus and achieve bending resistance and fire resistance. Following this strategy, Fang et al. [252] mainly focused on the design and optimization of solid electrolytes, especially by adding inorganic filler mesoporous silica nanofibers to improve ionic conductivity, lithium ion transference number and electrochemical stability, thereby realizing the application of high-performance lithium-ion batteries. The solid electrolyte they made was based on the electrospun PAN nanofiber membrane, coated with silica by sol–gel method, and further integrated into a self-supporting silicon/silicon carbide/carbon nanofiber anode to form an integrated anode@quasi-solid electrolyte membrane (SNF@PAN-E) (Fig. 18a, b). Figure 18c shows the electron transport pathway of SNF@PAN, where this design shows to comprehensively address the problems of low ionic conductivity, poor interfacial contact, and lithium dendrite growth in traditional solid-state electrolytes altogether, while also avoiding the leakage and safety hazards of liquid electrolytes. The advantage of quasi-solid-state electrolytes lies in combining the mechanical stability of solid electrolytes with the ion transport efficiency of liquid electrolytes, providing flexibility and interfacial compatibility through the polymer matrix, while introducing fillers to optimize the ion conduction path. Specifically, pure PAN nanofiber membrane, as the matrix of quasi-solid electrolyte, has an ionic conductivity of approximately 5.2 mS cm^−1^. However, by adding mesoporous silica nanofibers in different proportions (5, 10, 15, and 20 wt%), a composite membrane PANS-x (x represents the filler ratio) was formed, where the PANS-15 membrane with 15% of silica nanofibers exhibited the best performance. The electrolyte absorption rate was found to be as high as 765%, much higher than the 300−400% of the commercial Celgard 2325 separation membrane and 500% of the pure PAN membrane. This directly increased the ionic conductivity at room temperature to 8.0 mS cm^−1^, the lithium-ion transference number to 0.76, and the electrochemical stability window to 5.1 V. This improvement was primarily due to the unique structure of mesoporous silica nanofibers. Their helical pores, approximately 100 nm in diameter and several microns long, provided ample pore space, promoting electrolyte absorption and retention. Furthermore, the hydroxyl groups on the silica surface formed hydrogen bonds with fluoride ions, promoting lithium salt dissociation, reducing anion capture and increasing the free mobility of lithium ions. This, in turn, reduced polymer chain crystallinity and created a fast ion transport channel. An Arrhenius plot showed an exponential increase in ionic conductivity with increasing temperature, with a relatively low activation energy of approximately 0.2–0.3 eV, indicating that the filler reduced the ion migration barrier. In comparison, the ionic conductivity of pure PAN membrane was only 5.2 mS cm^−1^, with a lithium ion transference number of approximately 0.4–0.5. Upon addition of filler, the conductivity of the composite membrane initially increased and then decreased, ultimately dropping to 6.0 mS cm^−1^ for PANS-20. This was attributed to the excessive amount of filler causing nanofiber aggregation and increased transport resistance. Additionally, post-cycling analysis revealed that the Li foil from the SNF@PAN-E|Li configuration displayed superior surface smoothness compared to the SNF|PAN-E|Li system (Fig. 18d). AFM imaging confirmed reduced surface roughness, demonstrating that the integrated anode@electrolyte architecture enhanced lithium-ion transport stability while minimizing concentration polarization effects, consequently suppressing dendrite formation.

In addition to the common polymer substrates mentioned above, some materials such as polycarboxylates, polycarbonates, polynitriles, polyamides, polyimides, polysulfides and polyethylene glycols are currently also used as substrates for solid electrolytes. He and team have deeply explored the solid electrolyte design and ion conduction mechanism of self-healing dynamic supramolecular elastomer electrolyte (SHDSE), showing an important breakthrough in improving ion conductivity [253]. The research team constructed an integrated silicon anode/self-healing polymer electrolyte structure through in situ polymerization technology. The molecular structure of SHDSE contained multiple dynamic bonds, which enabled the polymer chain to reorganize and exhibit excellent electrochemical and mechanical properties, ultimately achieving high-performance solid-state lithium batteries. This molecular-scale architectural engineering facilitated seamless integration between the silicon anode and SHDSE electrolyte, establishing uninterrupted lithium ion conduction pathways across the anode-electrolyte interface while mitigating structural degradation resulting from silicon's volumetric expansion during cycling (Fig. 18e). The carefully designed SHDSE molecular structure in the study included a soft phase PEGDA to promote lithium ion transport, a hard phase cyclic compound 2-ureido-4-pyrimidinone (UPy) to provide strong supramolecular quadruple hydrogen bonds to enhance mechanical properties, and a deep eutectic solvent composed of LiTFSI and NMU to improve the dissociation ability of ions. Even more impressively, the soft-pack battery fabricated by the research team achieved a record-breaking volumetric energy density of 585.9 Wh L^−1^. Even under the harsher conditions of high temperature (100 ℃) and over 30,000 bend tests, it maintained a capacity retention of 86.2% after 700 cycles, with an average coulombic efficiency exceeding 99.9%. Based on three-dimensional surface topography analysis of the Si anode (Fig. 18f), both in situ SHDSE-Si and ex-situ SHDSE/Si electrodes exhibited comparable surface roughness values in their pristine state. Figure 18g presents the spatial distribution maps for DMT modulus, adhesion properties, and energy dissipation characteristics across both in situ SHDSE-Si and ex-situ SHDSE/Si configurations. The Si|LiCoO_2_ soft pack battery based on SHDSE electrolyte also exhibited excellent continuous bending performance at a high operating temperature of 100 ℃ (Fig. 18h). This excellent electrochemical performance was attributed to the significant dissociation of LiTFSI in SHDSE, which facilitated ionic conduction. The electrolyte utilized a deep eutectic solvent (DES) system consisting of LiTFSI and N-methylurea (NMU). Polyethylene glycol diacrylate (PEGDA) served as a soft phase, offering loose lithium-oxygen coordination and a low activation energy, effectively dissociating LiTFSI and promoting rapid lithium ion transport. Regarding electrical conductivity, the SHDSE electrolyte achieved an ionic conductivity of 3.54 × 10^−1^ S cm^−1^ at 25 °C. While slightly lower than the 4.20 × 10^−1^ S cm^−1^ of DES, this was due to the restriction of ionic movement by the cross-linked PEGDA segments. More importantly, compared to lithium-ion complexation via the ethylene oxide segment, complexation of lithium ions with the small molecule NMU significantly enhanced lithium-ion migration.

##### Thin-Film Solid Electrolytes

Thin-film solid electrolytes, on the other hand, are essentially thin-film forms of oxides, sulfides or polymer materials, and the basic ion conduction mechanism of the materials remains the same [286]. They all aim to solve the safety and stability problems of traditional liquid electrolytes and achieve the industrialization goal of all-solid-state lithium batteries. From the perspective of preparation technology, thin-film solid electrolytes are mainly prepared by thin-film technologies such as physical vapor deposition, chemical vapor deposition, and sputtering. The thickness is usually at the nanometer to micrometer level, which can significantly reduce the amount of material while maintaining good ion conduction performance [287]. The ion conductivity optimization strategy of thin-film solid electrolytes mainly focuses on material composition regulation, microstructure design and interface engineering. By precisely controlling the crystal structure, grain boundary density and connectivity of the ion conduction channel of the thin film, the migration ability of lithium ions in the thin film can be effectively improved. At the same time, the ultra-thin characteristics of the film greatly shorten the ion transmission path, which to a certain extent compensates for the lack of intrinsic ion conductivity of the material, thereby achieving significant improvement in the overall performance of the battery [288]. However, in practical applications, traditional thin-film solid electrolytes still face significant challenges. The ionic conductivity at room temperature is insufficient (10^−5^ ~ 10^−3^ S cm^−1^), which is significantly lower than that of traditional organic liquid electrolytes (10^−2^ S cm^−1^). At the same time, there are difficulties in forming an effective electrode-electrolyte interface. From the perspective of application characteristics, thin film electrolytes have advantages such as good interface contact, precise thickness control, and suitability for micro batteries, but the preparation cost is often higher than that of other types of solid electrolytes [289]. The relatively low ionic conductivity has long restricted the practical application of all-solid-state thin film batteries, prompting researchers to continuously explore new material systems and preparation processes to break through this technical bottleneck. Chen mainly focused on LiPON as a key component of all-solid-state lithium-ion batteries [290]. The battery used Li-Ni-Mn–O positive electrode and silicon anode thin film, prepared by sputtering deposition technology, to achieve high voltage operation of 4.55 V and excellent cycle stability. This study specifically optimized the sputtering process conditions and parameter settings to control the amorphous degree and nitrogen doping level of LiPON in real time, ultimately achieving the purpose of increasing conductivity. LiPON was chosen as a solid electrolyte because its amorphous structure could provide excellent lithium ion conductivity, higher electrochemical stability and lower self-discharge rate than liquid electrolytes, while avoiding the flammability and leakage risks of liquid electrolytes. Specifically, LiPON was deposited from a Li_3_PO_4_ ceramic target by RF magnetron sputtering in a nitrogen process gas. Although the nitrogen partial pressure exceeded that of oxygen, only a small amount of nitrogen replaced oxygen, which significantly affected the ionic conductivity and stability. The ionic conduction mechanism of LiPON relied on its amorphous network structure, in which phosphorus-oxygen–nitrogen bonds provided lithium-ion hopping paths. Low-temperature deposition ensured an amorphous phase and avoided crystallization leading to a decrease in conductivity. However, LiPON degraded after only 2 min of exposure to air, and flower-like pinholes appeared, indicating that the material was sensitive to environmental humidity, which could reduce the conductive performance, but its close contact with the electrode in all-solid-state batteries alleviated this problem. While LPON is a widely used thin-film solid electrolyte, sulfide-based thin-film solid electrolytes have made significant progress in improving ionic conductivity in recent years. Ultrathin, flexible Li_6_PS_5_Cl films prepared via a slurry casting strategy achieved a high ionic conductivity of 1.09 × 10^−3^ S cm^−1^ at room temperature, a value approaching that of liquid electrolytes. Furthermore, composite films prepared from a Li_6_PS_5_Cl-NBR composite exhibited an ionic conductivity of 0.18 mS cm^−1^, further demonstrating the feasibility of thin-film technology for maintaining high ionic conductivity. Hang's team studied the application of 70Li_2_S-30P_2_S_5_ glass-ceramic solid electrolyte in all-solid-state lithium batteries, supporting Li_2_SiS_3_ anode testing as an ion conductor [291]. Its sulfide-based electrolyte inhibited non-lithium ion migration through single-ion conduction, stabilized nanoscale Si particles, reduced capacity decay, and maintained the reversibility of conversion and alloying reactions. The room temperature ionic conductivity was about 1.5 × 10^−3^ S cm^−1^, thanks to the amorphous structure and the low barrier diffusion path provided by P_2_S_5_. It was verified by impedance spectroscopy and 0.01C cycling was demonstrated between −0.62 to 2 V. The electrolyte was pressed into 10 mm sheets and assembled with Li_2_SiS_3_ or Li_2_SiS_3_ + FeS electrodes and Li-In counter electrodes to ensure efficient ion transport and cycle stability. The conductive mechanism was derived from the high lithium content of Li_2_S and the P_2_S_5_ bridging network.

Annealing or halide doping can further improve the ion conductivity in these electrolyte systems. Optimization strategies may include atomic-scale doping and solution synthesis to enhance conductivity and uniformity and promote the commercialization of high-energy-density solid-state batteries. The effective construction of an ion-conducting network is also a core strategy for solving the key problem of low ionic conductivity in alloy-type anode solid electrolytes. It requires the coordinated promotion of multi-dimensional modification schemes to achieve efficient lithium-ion transport. Whereas, element doping modification is a basic strategy. By introducing appropriate doping elements, the geometric structure of the ion transport channel can be regulated. The ionic radius of the doping element should be larger than that of the host element to widen the ion transport channel. For example, Te doping and Al doping can significantly improve the bulk ionic conductivity. Microstructure regulation effectively maintains the ionic and electronic conductive pathways through innovative network structure design. The porous structure alleviates volume changes through pore shrinkage, and the fiber structure forms a continuous conductive network in the composite anode, which can maintain effective ion transport even if some contacts are disconnected. The development of elastic electrolytes provides a new approach to resolving the contradiction between mechanical properties and ionic conductivity. Through the design of soft-hard dual-monomer copolymers, while maintaining high ionic conductivity, excellent mechanical toughness and deformation adaptability can be achieved, thus effectively coping with volume changes during the alloying process. Inorganic filler modification introduces functional fillers to create fast ion transport channels. Mesoporous fillers provide ion transport space through their unique porous structure, and surface functional groups promote lithium salt dissociation, significantly improving ionic conductivity and the lithium-ion transference number. Thin-film technology significantly shortens the ion transport pathway to compensate for the material's inherent conductivity. Precisely controlling film structural parameters optimizes the connectivity of ion conduction channels, achieving efficient ion transport at ultra-thin thicknesses. Coating strategies establish a continuous ion transport interface by forming a conductive coating on the electrode surface. The coating material forms a chemical bond with the substrate, ensuring a tight bond and creating additional ion channels to enrich the transport pathway. Furthermore, surface modification techniques modify the material's surface properties to improve interfacial contact and ion transport kinetics, reduce interfacial impedance, and inhibit side reactions. The core goal of these modification strategies is to increase the number and quality of effective ion transport channels, lower the ion migration barrier, and reduce interfacial impedance, thereby constructing an efficient and stable three-dimensional ion conductive network. More importantly, several other modification strategies can be incorporated into the practical application of alloy-based anode solid electrolytes. The application of deep eutectic systems promotes the dissociation of lithium salts through intermolecular interactions, forming an efficient ion-conducting medium with good electrochemical stability and safety. Pressure-assisted strategies maintain good interparticle contact by applying appropriate mechanical pressure, but the pressure needs to be balanced to avoid damaging the material structure. Temperature regulation activates more ion transport channels by optimizing the operating temperature, improving ion mobility, but thermal stability limitations need to be considered. Synergistic modification strategies achieve synergistic effects by simultaneously introducing multiple functional components. For example, the composite of solid electrolytes and conductive carbon ensures both ion conduction and enhanced electron transport, achieving a significant improvement in overall performance. In practical applications, it is often necessary to select an appropriate modification strategy or a combination of multiple strategies based on the specific electrolyte system and electrode material properties to achieve optimal ion conductivity. With the continuous deepening of the understanding of the ion conduction mechanism of solid electrolytes and the continuous development of new material systems, the technology for constructing ion-conducting networks is developing in a more efficient, stable, and practical direction, laying an important technical foundation for the industrial application of high-performance all-solid-state lithium batteries.

#### Stable Interfacial Synergistic Effect

##### Thin-Film Solid Electrolytes

**Fig. 19:**
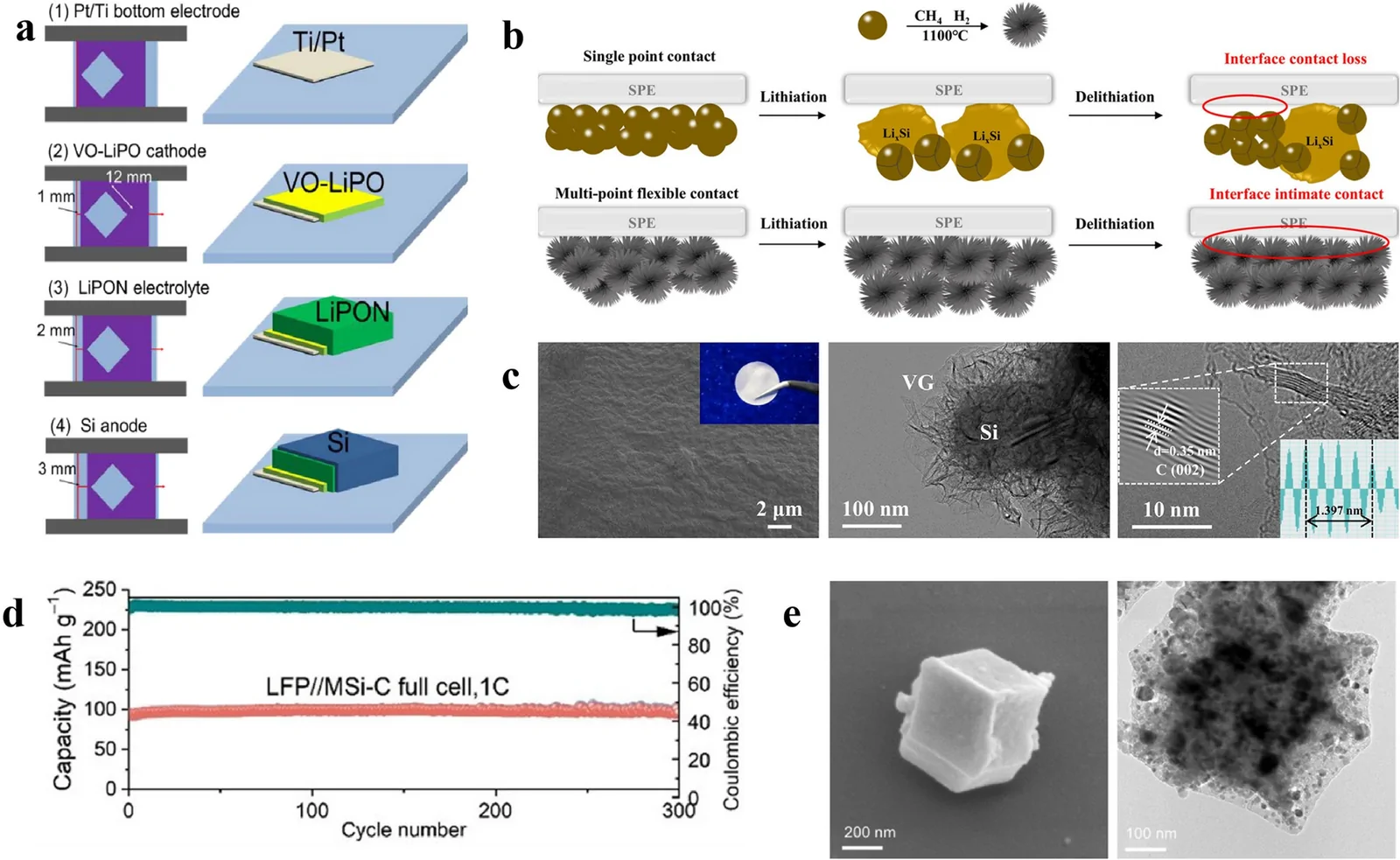
**a** Fabrication process for thin-film lithium-ion batteries [298]. © 2020 Elsevier B.V. All rights reserved. **b** Diagram illustrating the formation of Si@VG and mechanism of lithium insertion and extraction in Si and Si@VG; **c** SEM images of solid polymer electrolyte and TEM images of Si@VG [248]. © 2023 American Chemical Society. **d** Electrochemical cycling behavior of an LFP-Si full cell with a PVDF-HFP/LATP solid-state electrolyte [249]. © 2024 Published by Elsevier B.V. **e** SEM and TEM micrographs of the Si@MOF composite structure [304]. © 2022 American Chemical Society

In the construction of all-solid-state thin-film lithium-ion batteries, the interface characteristics and interface compatibility of solid electrolytes are key factors in determining battery performance, involving multiple complex physical and chemical processes. In the thin-film battery structure, the solid–solid interface formed between the solid electrolyte and the positive and anode materials have characteristics that are completely different from those of traditional liquid batteries. The properties of these interfaces directly affect ion transport, charge transfer, and the cycle stability of the entire battery [292]. The interface characteristics of solid electrolytes are mainly reflected in two aspects: interface structure and chemical composition. In terms of interface structure, due to the rigid characteristics of solid materials, solid-solid contact often has microscopic heterogeneity, resulting in the actual contact area being much smaller than the geometric contact area. This incomplete contact produces significant interface impedance [293]. At the same time, the lattice mismatch and thermal expansion coefficient differences between different materials also produce stress concentration at the interface, thereby affecting the mechanical stability of the interface. The complexity of the interface chemical composition stems from the chemical reactions that may occur between different materials. Especially within the operating voltage range of the battery, the solid electrolyte may undergo redox reactions with the electrode material to form an interface reaction product layer. The ionic conductivity of these reaction products is generally poor, which further increases the interface impedance [294]. Interfacial compatibility issues are particularly prominent in thin-film batteries, manifesting as multiple interrelated and complex phenomena. The extremely small thickness of each layer of thin-film battery material significantly amplifies the interfacial effect. The solid-solid contact between the solid electrolyte and the electrode material is microscopically heterogeneous, and the actual contact area is much smaller than the geometric contact area, resulting in significant interfacial impedance [295]. Chemical incompatibility manifests itself as irreversible chemical reactions at the interface, generating a reaction product layer with low ionic conductivity, which seriously affects ion transport in a confined space. Electrochemical incompatibility manifests itself as a mismatch between the electrochemical window of the solid electrolyte and the working potential of the electrode, making electrochemical decomposition more likely to occur under high electric field strength. Mechanical incompatibility manifests itself as differences in elastic modulus and thermal expansion coefficient between materials, leading to interfacial stress concentration and microcracks, which easily cause delamination under two-dimensional constraints [296]. Thermal incompatibility manifests itself as phase change or decomposition of the interfacial material caused by temperature changes. Local thermal stress in a rapid heat conduction environment accelerates interface degradation, ultimately leading to rapid battery performance degradation and shortened cycle life [297]. Kanazawa and team members used LiPON as the solid electrolyte layer thin film material, which is an amorphous electrolyte material that can be deposited by radio frequency magnetron sputtering at room temperature [298]. In order to obtain good interface contact with the LiPON electrolyte, the research team designed a multi-target sputtering system equipped with a movable shielding mask to prepare a multilayer structure by continuous vacuum deposition technology (Fig. 19a). The core of this method was to avoid direct contact between the layers while ensuring the integrity and compatibility of the interface. Specifically, a titanium adhesion layer and a platinum bottom electrode layer were first deposited on a borosilicate glass substrate in sequence, and then a lithium vanadium oxide (VO-LiPO) positive electrode layer, a LiPON electrolyte layer and a silicon anode layer were deposited in different areas by a movable shielding mask technology. The thickness of the LiPON electrolyte layer was 1.5 μm and was prepared by sputtering a Li_3_PO_4_ target in a nitrogen atmosphere of 0.3 Pa. It is worth noting that the study also demonstrated the stability of battery operation without a protective layer in an atmospheric environment, which is of great significance for practical applications. Although the discharge capacity dropped to 7.7 μAh cm^−2^ after 30 cycles, it showed better cycling stability than other similar amorphous thin film lithium-ion battery studies. This improvement was mainly attributed to the combination of amorphous vanadium oxide positive electrode and silicon anode, as well as the good interfacial compatibility with LiPON electrolyte. The realization of interfacial compatibility mainly relied on several key technical measures. First, all film layers were deposited at room temperature, avoiding interfacial reactions and thermal stress that might have been caused by the high-temperature treatment. Secondly, a continuous vacuum deposition process was used to prevent air exposure from damaging the interface quality. Thirdly, a movable shielding mask system ensured a clear interface boundary between the layers, avoiding internal short circuit problems caused by direct contact between the positive and anode materials [299]. The cross-sectional observation results of SEM confirmed the effectiveness of this preparation method, showing a dense film structure without cracks and voids, and a clear interface between the silicon/LiPON/VO-LiPO layers. This good interfacial structure was the basis for achieving stable electrochemical performance. From an electrochemical performance perspective, excellent interfacial compatibility was directly reflected in the battery's charge and discharge characteristics. The areal capacity reached 32.8 and 15.4 μAh cm^−2^ during the first charge and discharge cycles, respectively. This excellent performance was directly attributed to the good interfacial contact and ion conductivity between the LiPON electrolyte and the electrode materials. The success of this research not only demonstrated the superior performance of LiPON as a solid-state electrolyte, but more importantly, verified the feasibility of achieving high-performance all-solid-state batteries through precise control of interface engineering.

##### Polymers-Based Solid Electrolytes

In the development of all-solid-state batteries, the interface characteristics and interfacial compatibility between polymer composite electrolytes and electrode materials are key factors in determining battery performance. In the preparation of thin films or layers, vapor deposition technology has been fully applied, covering two major categories: Chemical Vapor Deposition (CVD) and Physical Vapor Deposition (PVD). CVD uses chemical reactions to generate solid materials such as metal oxides or carbides at high temperatures, while PVD directly vaporizes and deposits solid materials through physical processes such as evaporation or sputtering. It is widely used in many surface modification fields. The process conditions usually involve a vacuum environment or a specific gas atmosphere, and the temperature ranges from room temperature to thousands of degrees Celsius. In recent years, the combination of plasma or laser technology has further improved the deposition efficiency and film quality [300]. Specifically, TCVD (Thermal Chemical Vapor Deposition) technology deposits atoms or molecules by reducing or decomposing chemical vapor precursors at high temperatures, which can accurately construct an ultra-thin functional interface layer on the surface of the polymer composite electrolyte. The in situ deposited interface layer has good bonding strength and chemical compatibility with the polymer matrix [301]. The controllability of the TCVD process enables precise adjustment of the composition, thickness, and microstructure of the interface layer, thereby optimizing the ion conduction characteristics and electrochemical stability of the interface. The interface modification layer can effectively alleviate the chemical and electrochemical incompatibility between the polymer electrolyte and the electrode material, inhibit the occurrence of interface side reactions, and reduce the growth of interface impedance. At the same time, it also has good mechanical properties and thermal stability, which can withstand volume changes and temperature fluctuations during charge and discharge, and maintain long-term stability of the interface [302]. Zhang and research team used TCVD technology to grow vertical graphene sheets on the surface of commercial silicon nanoparticles to prepare Si@VG nanocomposites, which effectively improved the interface contact and impedance between the silicon anode and the PEO polymer solid electrolyte, and improved the electrochemical performance of the all-solid-state battery (Fig. 19b) [248]. The advantages of this polymer-based electrolyte are its flexibility, ease of processing, and good compatibility with the electrode. However, the interface between the traditional Si anode and SPE has significant challenges, including poor contact, high ion transfer impedance, and unstable interface during cycling, resulting in capacity fading and low coulombic efficiency. In Fig. 19c, the vertical array structure of VG creating a three-dimensional conductive network is shown, which increases the contact area between the SPE and the electrode. This reduces the interfacial impedance from 500 Ω cm^2^ to approximately 200 Ω cm^2^, ultimately enhancing ion transport efficiency and interfacial stability. In terms of interfacial structure, VG acts as a flexible buffer layer, similar to a bridge, connecting the rigid Si particles to the soft PEO matrix and preventing interfacial separation caused by Si volume expansion. Regarding compatibility, the hydrophobic surface of VG forms hydrogen bonds or van der Waals interactions with the hydrophilic segments of PEO, improving wettability and chemical compatibility. The team optimized the interfacial structure by encapsulating VG. The vertical arrangement of VG resembled "finger-like" protrusions, increasing the effective contact area by 2–3 times and forming a gradient interface. The core region near Si was a highly conductive VG layer, providing electron pathways. The outer layer, in contact with PEO, formed a porous composite interface, thereby promoting ion solvation/desolvation and enhancing interfacial compatibility. The enhanced C−O and Si−C bonds at the interface indicated that VG promoted chemical bonding and improved compatibility. The introduction of VG also reduced tortuosity through the 3D network, thus increasing effective conductivity by approximately 50%. The improved interfacial compatibility also manifested itself in mechanical stability.

Traditional solid electrolytes have poor fluidity and cannot effectively wet the surface of active materials, especially particle structures with large specific surface areas, which leads to the obstruction of the transmission dynamics of electrons and lithium ions and the generation of huge interface resistance. To solve this problem, Li et al. [249] used PVDF-HFP and LATP alloy anode composite solid electrolyte. The thickness of the electrolyte was about 50 µm and it showed excellent ionic conductivity. The interfacial compatibility between the solid electrolyte and the silicon anode is a key factor affecting battery performance. The researchers found that during the first discharge-charge process, the interface contains two semicircles. The high-frequency part corresponded to the solid electrolyte interface layer impedance (*R*_SEI_) and the low-frequency part corresponded to the charge transfer impedance (*R*_CT_). The R_SEI_ was found to be greater than *R*_CT_. During the lithiation process, the R_SEI_ decreased from 64 to 33 Ω. The reason for this kinetic enhancement was that, on the one hand, the Li_x_Si alloy had better ionic/electronic conductivity than pure silicon, which reduced the energy barrier between the solid electrolyte and MSi-C. On the other hand, the volume and thickness of the MSi-C anode increased during the lithiation process, which generated mechanical pressure at the interface and promoted the closeness of the physical contact of the interface. Through DRT model analysis, the researchers successfully separated the electrochemical processes with different time constants and gained a clearer understanding of the interface reaction mechanism. The improvement in interface compatibility was also reflected in the actual performance of the battery. The study showed that a close interface contact was formed between the MSi-C anode and the PVDF-HFP/LATP solid electrolyte. The cross-sectional SEM showed that there were no gaps or voids between the MSi-C and the SSE layer, confirming good interface compatibility. This excellent interface property enabled the battery to achieve high-performance cycling at room temperature without external pressure. It demonstrated the importance of optimizing interface design in improving the performance of solid-state batteries (Fig. 19d). The SEI layer formed in solid-state batteries was thinner and richer in inorganic components than traditional liquid batteries.

Although PVDF-based solid electrolytes have excellent mechanical strength and thermal stability, their relatively low room temperature ionic conductivity limits their practical application in high-performance solid-state batteries. In order to overcome this key technical bottleneck, researchers began to explore the strategy of combining PEO with excellent ionic conductivity with PVDF [303]. As a classic polymer electrolyte material, the ether oxygen groups in the molecular chain of PEO can form coordination with lithium ions, providing an effective channel for ion transport, thereby significantly improving the ionic conductivity of the composite system. By constructing a PVDF-PEO composite electrolyte system, not only can the excellent mechanical properties and chemical stability of PVDF be fully utilized, but also the high ionic conductivity of PEO can be obtained to achieve the synergistic effect of the two polymer materials, providing a new technical path for the development of solid electrolyte materials with both high ionic conductivity and good mechanical properties. Zhang et al. [304] developed a new type of PVDF fiber-supported polyethylene oxide/garnet composite electrolyte (PPG). This composite electrolyte was designed with careful consideration of interfacial compatibility. The PVDF fiber backbone not only enhanced the electrolyte's mechanical strength but also provided excellent structural support, while the addition of garnet filler significantly improved ion conductivity. Studies have shown that bare silicon electrodes experienced drastic volume changes during cycling, leading to interfacial failure with the PPG electrolyte, resulting in reduced contact area, increased impedance, and capacity decay. ZIF-67, as a cobalt-based zeolite imidazolate framework material, has a crystal structure constructed by Co^2+^ and 2-methylimidazole ligands through coordination bonds. It possesses a zeolite-like topological network and a controllable pore system. Compared to other MOFs materials, the significant advantage of ZIF-67 lies in its thermal stability and chemical stability, enabling it to maintain the framework integrity during the high-temperature carbonization process and form nitrogen-doped carbon-based composite materials with hierarchical pore channels. The structural advantage stems from the template effect of the ZIF-67 precursor, and its derivatives can inherit the high specific surface area and uniform metal distribution characteristics. In contrast, the silicon nanoparticles embedded within the MOF-derived carbon matrix in the Si@MOF effectively mitigated repeated deformation and provided charge transfer pathways. While bare silicon electrodes experienced severe expansion and cracking and voiding after cycling, the Si@MOF electrode exhibited controlled expansion and a stable interface (Fig. 19e). At an operating temperature of 60 °C, PPG formed a conformal interfacial contact with the Si@MOF anode and maintained this contact during cycling, eliminating the need for high external pressure throughout battery operation. This contrasts sharply with conventional oxide or sulfide solid electrolytes, which typically require high external pressures of 20–370 MPa to maintain interfacial contact with the silicon electrode and suppress morphological changes. The flexibility of the PPG composite electrolyte enabled operation without high external pressure, significantly simplifying operational requirements in practical applications. After activation cycling, the Si@MOF electrode exhibited lower charge transfer resistance than the bare silicon electrode, indicating that the MOF-derived carbon-based stabilized SEI layer facilitated more efficient charge transfer. Symmetrical cell testing showed that the Si@MOF symmetric cell could cycle stably for 1200 h at a current density of 0.2 mA cm^−2^ with virtually no change in overpotential, demonstrating the excellent interfacial stability between the Si@MOF anode and the PPG electrolyte. A full cell using an LFP cathode exhibited a capacity of 148 mAh g^−1^ at 0.2 C, with a retention of 91.9% after 100 cycles. This excellent cycling stability was directly attributed to the stable interfacial structure between the Si@MOF anode and the PPG electrolyte. The symmetric cell demonstrated stable operation over 1200 h with minimal overpotential variation, confirming robust interfacial stability between the Si@MOF anode and PPG electrolyte. The combination of the Si@MOF anode and the PPG electrolyte achieved excellent interfacial compatibility, consequently significantly improving the cycling stability and high-load performance of PEO-based electrolytes.

**Fig. 20:**
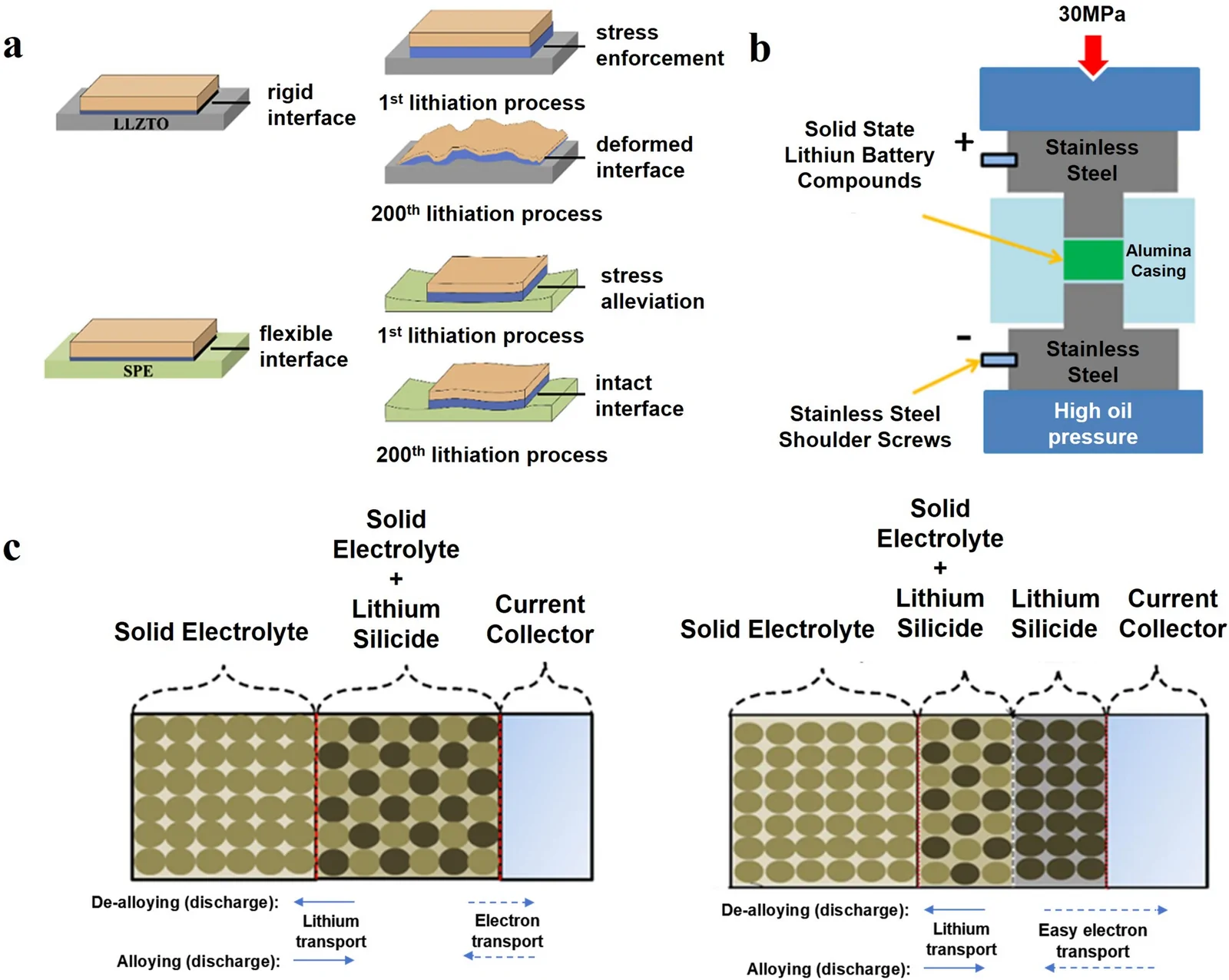
**a** Diagram of flexible and rigid interfaces during the 1st and 200th lithium insertion cycles [250]. © 2018 Elsevier B.V. All rights reserved. **b** Illustration of the test die designed for measuring the conductivity of solid electrolytes and conducting battery cycling experiments. **c** Illustrations depicting the transport of lithium ions and electrons through the solid electrolyte, composite anode, and current collector in conventionally(gradient) structured anodes [310]. © 2015 Elsevier Ltd. All rights reserved

Using LiTFSI and LLZTO as fillers in the polymer solid electrolyte, different approaches can lead to different battery performance outcomes. Huo and group reported an innovative strategy to construct a flexible interface between silicon anode and PPC and garnet composite electrolyte, aiming to develop high-performance solid-state lithium batteries [250]. The team used a mechanochemical method to prepare PPC/garnet/LiTFSI solid polymer electrolyte. PPC with a molar mass of 5 × 10^4^ g mol^−1^, 200-nm-sized LLZTO ceramic powder and LiTFSI lithium salt were uniformly mixed by high-energy ball milling to prepare a composite electrolyte membrane with a thickness of about 70 µm. The ionic conductivity of the composite electrolyte reached 4.2 × 10^−4^ S cm^−1^ at room temperature, which was 2.5 times higher than that of pure PPC electrolyte without LLZTO. It was mainly attributed to the percolation effect of LLZTO particles, which created a highly conductive path, promoted the complete dissociation of lithium salts, and suppressed the formation of ion clusters. The change in the percentage of free TFSI^−^ proved that the LLZTO additive significantly improved the ion dissociation degree. Furthermore, a 150-nm-thick amorphous silicon layer was deposited directly onto the composite electrolyte surface via DC magnetron sputtering at 25 ℃, followed by a 500-nm-thick copper layer as the current collector, forming a tightly integrated Si/SPE flexible interface. This resulted in silicon layer deformation and detachment from the underlying electrolyte support matrix (Fig. 20a). The Si/SPE/Li battery achieved a specific capacity of 1342 mAh g^−1^ at a 1 C rate, with a capacity retention of 86.1% after 200 cycles, demonstrating excellent rate performance and cycling stability. The full cell retained 82.6% of its capacity after 100 cycles at room temperature. The prepared flexible pouch cell was able to power an LED bulb, demonstrating its potential for application in flexible energy storage devices. Importantly, the tensile and compressive moduli of the PPCL-SPE were found to be 15.6 and 18.1 MPa, respectively, far lower than the 100 GPa of the LLZTO ceramic electrolyte. This allowed the PPCL-SPE to effectively mitigate the significant stress generated by the approximately 280% volume change of the silicon anode during charge and discharge through its own deformation.

##### Sulfide-Based Solid Electrolytes

Sulfide solid electrolytes have attracted much attention due to their high ionic conductivity and good formability. However, when in contact with electrode materials, chemical reactions easily occur to form an interface phase with high resistance, which affects ion transport. Through strategies such as surface coating, interface buffer layer and element doping, the interface stability and compatibility can be improved, thereby improving battery performance. Among the many sulfide solid electrolytes, Li_6_PS_5_Cl (LPSCl) is the most common. Huo and team members found that the composite Si/Li_6_PS_5_Cl anode has a rapid capacity decay due to the continuous growth of the SEI layer [305]. Although the silicon anode without the solid electrolyte has sufficient ionic/electronic conductivity and higher specific capacity, it will form micron-scale voids at the two-dimensional Si|Li_6_PS_5_Cl interface during the delithiation process, resulting in increased mechanical stress and poor cycle stability. LPSCl was chosen by them as the solid electrolyte due to its high ionic conductivity and mechanical rigidity. Its interface structure in the composite anode was characterized by direct contact between a ~ 20 nm thick SiO_x_ impurity layer on the Si particle surface and LPSCl. Although no new crystalline phase formed, elemental diffusion caused the ionic conductivity to drop from 2.5 × 10^−5^ to 1.9 × 10^−5^ S cm^−1^, while the electronic conductivity remained stable at approximately 8 × 10^−6^ S cm^−1^. This interfacial compatibility issue stemmed from the reductive decomposition of LPSCl at low potentials. The resulting SEI was comprised of Li_3_P, Li_2_S, and LiCl, while SiO_x_ reacted to form SiO_2_, Li_x_SiO_y_, and Li_2_O. These byproducts hindered the ion/electron percolation network, explaining the rapid capacity decay of the composite anode. It presents HAADF cryo-scanning transmission electron microscopy imaging of silicon and low-melting-point graphene (LPSCl) particles in interfacial contact following 100 cycles, with accompanying EDS mapping providing clear elemental distribution analysis. The complexity of the interfacial structure was further reflected in the three-dimensional composite interface. During lithiation, silicon expansion densified the layer, while delithiation caused submicron crack propagation. While the contact was good, SEI growth increased resistance and tortuosity. LPSCl exhibited poor interfacial compatibility in composite anodes due to SEI growth disrupting ionic contacts. While SEI-dominated silicon anodes without solid electrolytes presented no such issues, mechanical rigidity lead to void-induced stress concentration. Optimizing the interfacial structure involved surface modification with a PPC buffer layer to suppress the SEI and alleviate stress. In a full-cell Si|LPSCl|NCM@LBO, a PPC buffer layer improved stability to 71.9% and reduced stack pressure requirements. Compatibility was also be improved by replacing S with O or doping with other elements to broaden the electrochemical window and avoid low-potential decomposition. Overall, this study emphasized that interfacial SEI growth dominated the resistance increase of LPSCl in composite anodes. In contrast, the two-dimensional interface in solid-electrolyte-free silicon anodes was susceptible to mechanical failure. This study provided design guidance, such as optimizing the three-dimensional composite interface to balance ionic compatibility and mechanical stability, or introducing a flexible buffer layer to enhance compatibility, to achieve all-solid-state batteries with energy densities as high as 300 Wh kg^−1^. It presents ToF–SIMS (Time-of-Flight Secondary Ion Mass Spectrometry) mass imaging of silicon particles within the lithium sodium phosphate matrix in pristine and post-100-cycle states, respectively. Notably, cycling induced the formation of an intensified sulfur fragment layer surrounding the silicon particles.

Extending to similar sulfide solid electrolytes, interfacial issues generally stem from chemical instability and differences in mechanical rigidity. Compared to oxide solid electrolytes such as LLZO, LPSCl offers greater flexibility, but compatibility needs to be optimized to prevent excessive SEI growth. In full cells, variations in forward pressure can alleviate compatibility issues, but optimization is required to avoid high-voltage requirements. In summary, the interfacial structure and compatibility of LPSCl are performance bottlenecks for all-solid-state batteries. Multiscale characterization and simulation provide a framework for designing highly compatible interfaces. In solid-state battery systems, the interface characteristics between the electrode and the solid electrolyte determine the performance and service life of the entire battery. The interface must not only have excellent flexibility to adapt to the volume change and stress release of the electrode material during charging and discharging, but also have good mechanical stability to maintain long-term physical contact and structural integrity. The appropriate ratio of the two is often the key to the success of a battery. Flexibility ensures that the interface can withstand repeated mechanical deformation without breaking, especially for high-capacity materials such as silicon anodes, whose huge volume expansion and contraction require the interface to have sufficient elasticity to relieve stress concentration. At the same time, mechanical stability ensures that the interface may not undergo irreversible plastic deformation or interface separation during long-term cycling, thereby maintaining a stable ion transmission channel. When flexibility and mechanical stability reach the optimal balance, the interface can effectively buffer the volume effect of the electrode material and maintain long-term structural stability. Its synergistic effect is the core element for achieving high-performance solid-state batteries [306]. He and team studied the effect of different stacking pressures on the performance of silicon-based anode all-solid-state batteries and found that high pressure can inhibit the volume expansion and crack formation of silicon, reduce the decomposition of Li_6_PS_5_Cl solid electrolyte, and thus significantly improve the electrochemical performance [307]. First, from the perspective of the physical interface, the study found that applied pressure has a decisive influence on the interfacial contact quality between the silicon-based anode and the LPSCl solid electrolyte. Under low-pressure conditions, the silicon-based anode undergoes significant volume expansion and contraction during charge and discharge, leading to significant lateral cracks and contact failure between the electrode and the solid electrolyte. In contrast, under higher pressure conditions, the solid electrolyte and the silicon-based anode maintained close physical contact, ensuring good interfacial continuity even after the silicon material underwent lithiation and expansion. From the perspective of interfacial chemical stability, pressure conditions profoundly influenced the decomposition behavior of LPSCl at the silicon-based anode interface and the formation of the SEI. Under low-pressure conditions, LPSCl undergoes severe decomposition during long-term cycling, generating large amounts of decomposition products such as Li_2_S. These decomposition products accumulate on the silicon-based anode surface, forming a thick SEI layer, which not only consumes active lithium ions but also increases interfacial impedance, leading to rapid battery capacity decay. This decomposition behavior is closely related to the stress distribution and contact state at the interface. The good interfacial contact can reduce local stress concentrations, thereby suppressing the decomposition reaction of the electrolyte. In terms of interfacial structural evolution, after 100 charge-discharge cycles, the silicon-based anode under low pressure significantly increases in thickness, with numerous cracks and voids appearing internally. These structural defects further deteriorate the interfacial contact with the solid electrolyte. However, under high pressure, the thickness decreases. This is because the high pressure effectively suppresses the expansion of the silicon material, while the volume of the LPSCl was also compressed under pressure, resulting in a shrinkage of the overall electrode volume while maintaining the integrity of the electrode structure. Finally, from the perspective of overall interfacial compatibility, pressure conditions determined the long-term stability and electrochemical reversibility of the interface between the silicon-based anode and the LPSCl solid electrolyte. Under a pressure of 300 MPa, the silicon-based anode maintained a high specific capacity of 2268 mAh g^−1^ after 100 cycles, with a capacity retention rate of 86.62% and a coulombic efficiency stable above 99.5%. These excellent electrochemical performances were directly attributed to the high stability and good compatibility of the interface under high pressure. Due to the rigid contact and mismatched mechanical properties of the materials, complex stress distribution and interface failure problems often occur. In this context, the application of alloy-base materials in solid-state batteries still faces unique chemical-mechanical coupling challenges [305]. The huge volume change of alloy-base materials during lithiation/delithiation not only causes severe mechanical stress, but also triggers complex interfacial chemical reactions and forms a SEI layer. These factors jointly determine the cycle stability and electrochemical performance of silicon-based solid-state batteries [308].

The continuous growth of SEI film in liquid electrolyte lithium-ion batteries lead to severe impedance increase and rapid capacity decay. This problem is particularly prominent in high-capacity alloy anode materials. In order to fundamentally solve this challenge, all-solid-state battery technology has emerged. It uses solid electrolytes to replace flammable organic liquid electrolytes, which can not only eliminate the problems caused by SEI film formation, but also provides better safety and stability [309]. However, all-solid-state batteries face new technical challenges such as poor solid-solid interface contact and large ion transport resistance. Therefore, it is necessary to optimize the interface performance through innovative electrode design strategies to achieve the practical application of high-performance all-solid-state lithium-ion batteries. Jin and team members improved the electrochemical performance of lithium-silicon alloy anodes by optimizing electrode design [310]. Specifically, they used secondary ball milling to reduce the particle size to increase the contact area between the solid electrolyte and the anode active material, and designed a gradient structure anode to promote the transfer of lithium ions between the anode and the electrolyte and the transfer of electrons between the anode and the current collector. The experimental results showed that these two methods significantly improved the charge and discharge capacity and cycle performance of the battery (Fig. 20b). Research indicates that the fundamental reason for the inferior performance of all-solid-state batteries compared to traditional liquid electrolyte batteries lies in the high impedance at the interface between the solid electrolyte and the electrode. This is due to the difficulty in forming an ideal wet and soft interface between the solid materials, resulting in significantly increased resistance to lithium-ion transport at the interface. To address this interfacial compatibility issue, the study employed two strategies to improve the interfacial properties between the anode and solid electrolyte, and between the anode and current collector. A gradient anode structure was designed, consisting of a composite layer of silicon-lithium alloy and solid electrolyte, and a pure silicon-lithium alloy layer (Fig. 20c). This design effectively balanced ion and electron transport: the solid electrolyte in the composite layer provided more pathways for lithium ions to move between the anode and electrolyte, while the pure silicon-lithium alloy layer ensured excellent electronic conductivity, reaching 10^−4^ S cm^−1^, significantly higher than that of a composite layer containing an insulating solid electrolyte. This improved interfacial compatibility was evident in the electrochemical performance: the battery using secondary ball milling achieved an initial discharge capacity of 200 mAh g^−1^, more than double that of the untreated battery. The discharge and charge capacities of the gradient structure battery far exceeded those of traditional single-layer designs. It is particularly noteworthy that when the two strategies of secondary ball milling and gradient structure were combined, the battery still maintained a capacity of 70–90 mAh g^−1^ even at a high current density of 400 mA g^−1^. This performance is comparable to that of the traditional design at a low current density of 200 mA, fully demonstrating the importance of interface engineering in all-solid-state batteries. The gradient structure accelerated the alloying and dealloying kinetics, with obvious oxidation peaks appearing at 1.5 and 1.7 V, indicating that the reduction in interfacial impedance promoted the electrochemical reaction. The same ball milling operation is adopted, but the silicon prepared by PVD has the advantages of dense and uniform surface, controllable particle size, strong bonding force and high purity. It can significantly alleviate the problem of powdering and shedding caused by volume expansion, improve cycle stability and Coulombic efficiency, and achieve excellent conductivity and structural stability through layered or composite deposition design, which is better than traditional silicon particles. It is well known that sulfide electrolytes are regarded as ideal electrolytes for ASSLBs because of their high ionic conductivity, good plasticity and close contact with electrode materials. However, when in contact with silicon anodes, chemical/electrochemical reactions were prone to generate insulating byproducts such as Li₂S, Li_3_P, and LiCl as also discussed above, which lead to increased interfacial impedance and cycle attenuation. At the same time, the volume change of silicon during charging and discharging was as high as 300%, which caused powdering, shedding and interface peeling, thereby accelerating battery failure [311].

Compared with liquid electrolytes, solid electrolytes can form dense two-dimensional interfacial contacts on the surface of silicon wafers, thus offering several advantages. Their rigid structure prevents the electrolyte from penetrating into cracks and defects within the electrode, helping to maintain interfacial stability and inhibits the formation of harmful SEI. The interface can also continuously coat the silicon surface during the cycle, alleviating mechanical failure and pulverization caused by volume change, and reducing stress concentration during the lithiation process by dispersing local stress. However, it is very difficult to achieve ideal conformal interface contact on a flat silicon surface. The uneven interface leads to uneven distribution of the lithiation reaction, with some areas preferentially expanding and squeezing the electrolyte along a specific crystal direction, making the contact points denser, but also exacerbating the gap expansion in other areas, and ultimately causing peeling between the electrode and the electrolyte. To solve this problem, in the study of specific crystal orientations of silicon anode materials, it was found that different crystal orientations have a significant effect on the diffusion behavior of lithium ions in silicon. Among them, the < 110 > direction has the largest gap space, providing the fastest diffusion path for lithium ions, while the diffusion rate of the < 111 > and < 100 > directions is relatively slow [312]. The choice of crystal orientation directly affects the lithiation depth, volume expansion characteristics and cycle stability of the silicon anode. Among them, the < 110 > crystal orientation silicon wafer can achieve a more uniform lithium ion distribution and a deeper lithiation depth during the lithiation process. Na and team members found that the surface groove structure of the < 110 > crystalline silicon wafer, after KOH etching, significantly improved the interface contact with the solid electrolyte, effectively inhibiting the cracking and pulverization during the cycle [254]. Finally, a single-chip 100% silicon wafer anode for all-solid-state batteries was developed. The surface groove structure of the < 110 > crystalline silicon wafer after KOH etching significantly improved the interface contact with the solid electrolyte, effectively inhibiting the cracking and pulverization during the cycle. The groove could penetrate the electrolyte with good ductility under the pressure of battery manufacturing, forming a more conformal and tighter interface contact, which not only increased the reaction area but also optimized the uniformity of the lithiation current distribution. It demonstrates the preferential expansion pathways for silicon wafers of varying crystallographic orientations: < 100 > , < 110 > , and < 111 > substrates possess four, two, and six in-plane [110] directions, respectively. Among these configurations, the bare < 111 > wafer experiences the most pronounced in-plane expansion at the silicon-substrate interface, while bare < 100 > and bare < 110 > wafers showed progressively reduced expansion. The substantial volumetric growth along < 110 > directions generated considerable compressive stress release. Electrochemical impedance spectroscopy analysis showed that the grooved silicon wafer had a low and consistent contact impedance, while the flat silicon wafer had a high impedance at the beginning of the cycle, while it gradually densified afterward through expansion in the thickness direction, whereas the impedance slowly decreased. Grooved silicon maintained low impedance and interface integrity during the cycle, which was reflected in the voltage curve as a lower overpotential and more stable charge and discharge behavior. Furthermore, interfacial behavior was significantly influenced by the crystal orientation of the silicon wafer. The < 110 > orientation facilitated lithium ion diffusion along the thickness, minimizing in-plane expansion, reducing stress, and maintaining interfacial stability. However, the < 111 > orientation favored the formation of high-concentration lithium alloys near the interface, triggering stress concentration and compromising interfacial integrity. This finding demonstrates that the interfacial stability depends not only on surface morphology control but also on the intrinsic crystal properties of the material, providing important insights into interfacial design for solid-state batteries.

**Fig. 21:**
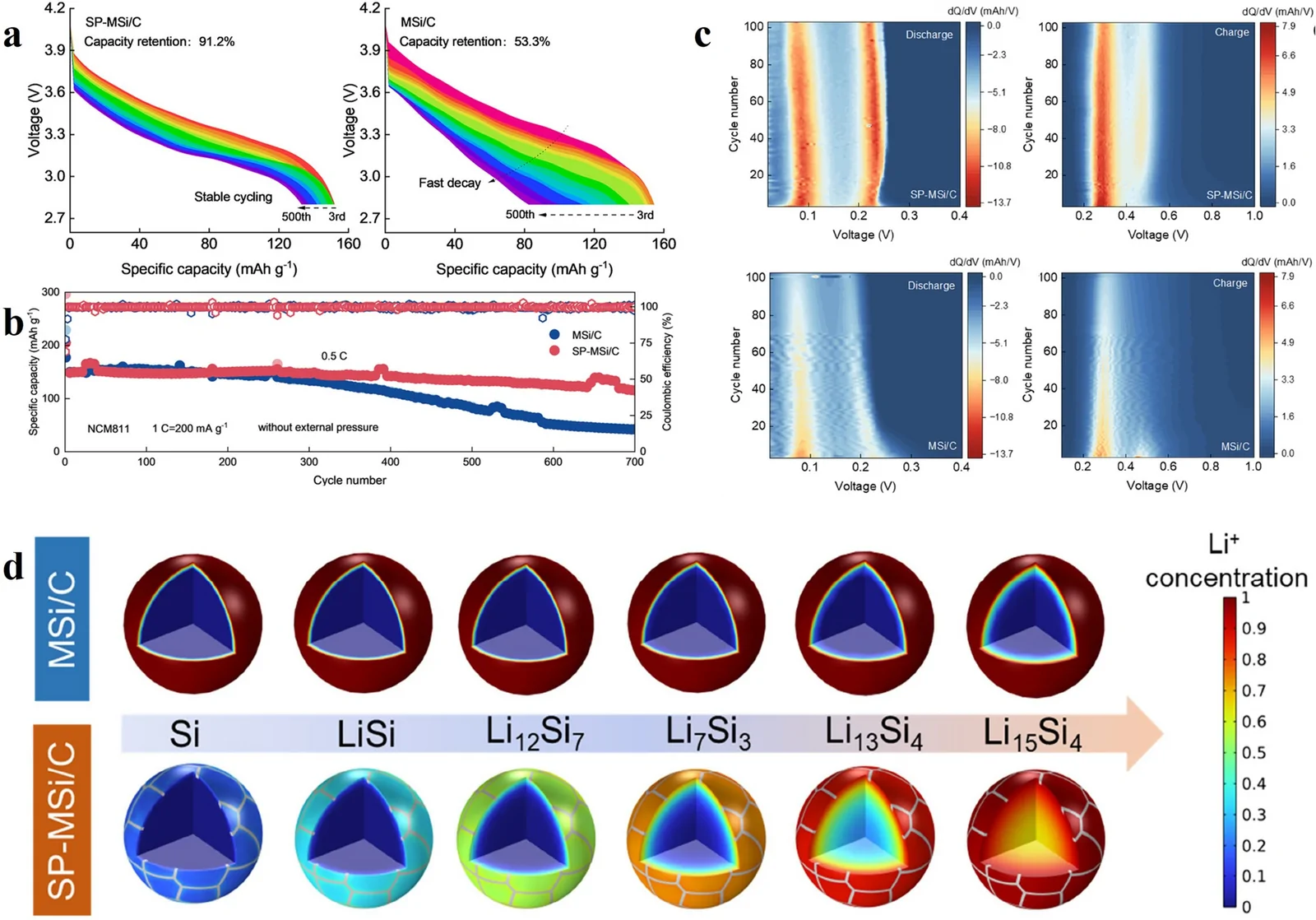
**a** Discharge curves for SP-MSi/C and MSi/C full cells over the initial 500 cycles. **b** Extended cycling behavior of SP-MSi/C/SSE/NCM811 and MSi/C/SSE/NCM811 full cells. **c** Contour plots of differential capacity (dQ/dV) versus voltage for SP-MSi/C and MSi/C at various cycle counts. **d** Modeled profiles of Li^+^ concentration for SP-MSi/C and MSi/C composites [315]. © 2025 American Chemical Society

In the development of solid-state batteries, the structural design and performance optimization of the interface between solid electrolytes and electrodes have always been the key technical bottlenecks that restrict their practical application. Different types of solid electrolyte systems exhibit completely different interface behaviors and electrochemical performances when in contact with alloy-type anode materials due to their unique chemical composition, physical properties, and interface characteristics. Polymer-based solid electrolytes usually have good flexibility and interface contact, and can adapt to the volume changes of electrode materials during charging and discharging to a certain extent, but their ionic conductivity is relatively low [313]. Sulfide solid electrolytes are known for their excellent ion conductivity, but interface stability and mechanical matching often face greater challenges [314]. In order to gain a deeper understanding of these differences and guide the optimization design of interface engineering, researchers began to adopt a comparative study strategy, systematically comparing the interface behaviors of different solid electrolyte systems under the same experimental conditions, thereby revealing the intrinsic correlation mechanism between interface structure and electrochemical performance. Qin et al. [315] used two different types of solid electrolyte systems: PVDF-HFP/LLZO composite polymer electrolyte and Li_6_PS_5_Cl sulfide solid electrolyte, and deeply explored the relationship between interface structure and performance. In a PVDF-based composite solid electrolyte system, these researchers fabricated a composite electrolyte film containing PVDF-HFP, PVDF, LiTFSI, and LLZO nanoparticles via solution casting. The film exhibited an ionic conductivity of 3.07 × 10^−4^ S cm^−1^, and its appropriately porous structure provided excellent pathways for ion transport. Importantly, the composite electrolyte formed a stable interfacial contact with the silicon-carbon anode, maintaining close interfacial bonding even under no external pressure (0 MPa) after 10 cycles, thus avoiding the interfacial separation and contact failure associated with conventional silicon anodes due to volume changes. Interfacial impedance analysis revealed that the self-pressure structure significantly improved the solid-solid interfacial transport dynamics. This enhanced interfacial compatibility was primarily attributed to the unique electron-ion hybrid conductive network formed by the compressed carbon nanotube (CNT) network encapsulated by the ion-conductive PEO/LiTFSI in the self-pressure structure. This structural design enabled dynamic adaptive contact at the interface. When the silicon particles expanded, the compressed CNTs provided mechanical buffering while maintaining electron and ion transport pathways. Compared with MSi/C, the SEI film accumulation and lithium ion trapping in the SP-MSi/C system remained relatively stable, indicating that the self-pressure structure not only improved the mechanical contact but also enhanced the chemical stability of the interface. This stable interface structure enabled the full battery to achieve long-term stable operation for 700 cycles without external pressure, with a capacity retention rate of 79.2% (Fig. 21a, b). Figure 21c presents capacity differential analysis for SP-MSi/C and MSi/C spanning cycles 3 through 100. Conversely, SP-MSi/C maintained consistent peak intensity and positioning throughout charge-discharge cycling, attributed to superior electrode stability and enhanced reaction kinetics uniformity. Figure 21d illustrated lithium-ion distribution patterns during initial lithiation of MSi/C and SP-MSi/C electrodes. Within the SP-MSi/C architecture, lithium-ion concentration remained minimal at the outer shell, while achieving uniform dispersion throughout the self-pressing network and internal MSi particles. This demonstrates that relative to MSi/C, the SP-MSi/C configuration facilitates accelerated lithium-ion flux transfer from the external self-pressing framework to active materials under volumetric fluctuations, resulting in enhanced lithiation efficiency.

Unlike traditional liquid battery architectures, solid electrolytes cannot penetrate porous micro-silicon electrodes, so that the interfacial contact area between solid electrolytes and micro-silicon electrodes is limited to a two-dimensional plane. The unique interfacial geometry can remain unchanged even if the volume expands during the silicon lithiation process, thereby preventing the generation of new interfaces [316]. The key to forming a stable passivation SEI on the surface of micro-silicon anodes lies in controlling the extent of interfacial reactions and the properties of the products. In traditional liquid electrolyte systems, continuous side reactions occur between silicon and electrolytes, forming an unstable and growing SEI film, which leads to lithium ion inventory loss and increased impedance. In the sulfide solid electrolyte system, a highly passivating SEI layer can be formed on the silicon surface. This SEI has good ionic conductivity and electronic insulation, which can effectively prevent further interfacial reactions, thereby achieving stable cycle performance and avoiding the problem of continuous growth of SEI in traditional liquid electrolytes [255]. However, there are still many challenges to achieve true industrial application, especially the carbon additives commonly used in traditional silicon anode design significantly promote the decomposition reaction of the solid electrolyte and destroy the interfacial stability. Tan and team completely eliminated the carbon additives, not only avoiding the problem of solid electrolyte decomposition caused by carbon, but also making full use of the electronic conductivity of silicon itself and the interfacial passivation characteristics of the sulfide solid electrolyte, and achieved stable and efficient operation of the micron silicon anode in the all-solid-state battery system [255]. Carbon additives have a significant negative impact on the interfacial stability of the solid electrolyte. By comparing silicon-solid electrolyte composite anodes with and without carbon additives, the experiment showed that the initial voltage platform of the carbon-containing electrode during the first lithiation process was only 2.5 V, which indicated that a large amount of solid electrolyte electrochemical decomposition occurred before reaching the lithiation potential above 3.5 V. XRD (X-ray Diffraction) analysis further confirmed this phenomenon. The solid electrolyte diffraction signal in the carbon-containing electrode almost disappeared, indicating severe electrolyte decomposition and the formation of a product dominated by nanocrystalline Li_2_S. In contrast, the carbon-free electrode retained the crystalline structure of the electrolyte and unreacted silicon, with only a small amount of amorphous lithium-silicon alloy signal appearing at approximately 20°. More Li_2_S formed in the S 2*p* region, while the intensity of the PS_4_^3−^ thiophosphate unit signal decreased significantly. Regarding interfacial contact and mechanical compatibility, the study revealed a unique chemomechanical behavior between the sulfide solid electrolyte and the silicon anode. During lithiation, the microsilicon particles maintained direct ionic and electronic contact with the solid electrolyte through the lithium-silicon alloy. This contact allowed rapid lithium-ion diffusion and electron transport throughout the electrode, unhindered by any electronically insulating components such as the SEI or electrolyte. Morphological evolution studies revealed that the originally discrete microsilicon particles formed a dense, interconnected lithium-silicon alloy structure after lithiation. The electrode porosity decreased from an initial 40% to less than 10%, and the particle boundaries completely disappeared. After delithiation, although the electrode did not return to its original discrete microparticle structure, a large particle structure with voids was formed. Importantly, good contact was maintained between the porous lithium-silicon structure and the solid electrolyte layer after delithiation. More importantly, Tan et al. conducted a detailed exploration of the self-discharge performance of the battery, a performance that is often not mentioned or valued in other literature. Compared with liquid electrolyte batteries, solid electrolyte batteries can maintain about 99% of the charge even at 55 ℃.

##### Others

In addition to the common LPSCl sulfide-based solid electrolyte, Li_10_SnP_2_S_12_ (LSnPS) has also been widely used in alloy-type anode solid electrolytes. Studies have found that although the ionic conductivity of the two electrolytes is similar, LSnPS is 1.4 × 10^−3^ S cm^−1^ and LPSCl is 1.7 × 10^−3^ S cm^−1^, there are significant differences in their interfacial reactivity with silicon anodes. The LPSCl electrolyte exhibits better interfacial stability. Grandjean and team focused on comparing the interfacial behavior of LSnPS and LPSCl, two solid electrolytes with similar ionic conductivity, with silicon materials of different sizes [317]. The initial Coulombic efficiency of LSnPS with micron-sized silicon particles reached 90%, while that of LSnPS was only 40%, indicating that LSnPS underwent a more serious decomposition reaction at the interface, forming a thicker SEI. EIS analysis revealed a characteristic frequency of the SEI layer of approximately 0.5 Hz, significantly lower than the typical frequencies above 1 kHz in liquid electrolyte systems, indicating that the SEI layer formed on the silicon surface has poor conductivity. Silicon nanowires, due to their high surface area and unique morphology, enabled better dispersion within the LPSCl matrix, forming a more uniform composite interface. EDX elemental mapping confirmed the colocalization of Si and S, indicating a closer interface between the solid electrolyte and the nanowires. In contrast, the interface between micronized silicon particles and the electrolyte exhibited a mutually repulsive distribution pattern, resulting in a limited contact area. Interfacial impedance studies indicated that the initial SEI impedance of silicon nanowire cells was much lower than that of micronized silicon cells. It was attributed to the larger interfacial contact area provided by the nanowires, which reduced the current density at the interface and formed a thinner but more stable SEI layer. Furthermore, the interfacial impedance of the micronized silicon cell increased tenfold due to the particle comminution effect, while the nanowire cell only increases sevenfold, demonstrating better interfacial stability. The difference in interfacial compatibility was also reflected in the different lithiation mechanisms. Nanowire systems tend to form amorphous Li_x_Si phases, while micron silicon can achieve deeper lithiation to form crystalline Li_15_Si_4_ phases. However, this deep lithiation is accompanied by greater volume expansion and interfacial stress, leading to interface damage and performance degradation. The team also found that solid electrolytes cannot penetrate into the interior of nanowire aggregates, which limits the contact between some nanowire surfaces and the electrolyte, but overall maintains good interfacial compatibility. The improvement in interfacial stability allowed nanowire batteries to maintain 63% of their capacity after 15 cycles at a C/20 rate, while micron silicon batteries only maintained 53%. In long-term cycling at higher rates (C/10), the nanowire system still had silicon contributions after 100 cycles, demonstrating excellent interfacial durability and cycle stability.

**Fig. 22:**
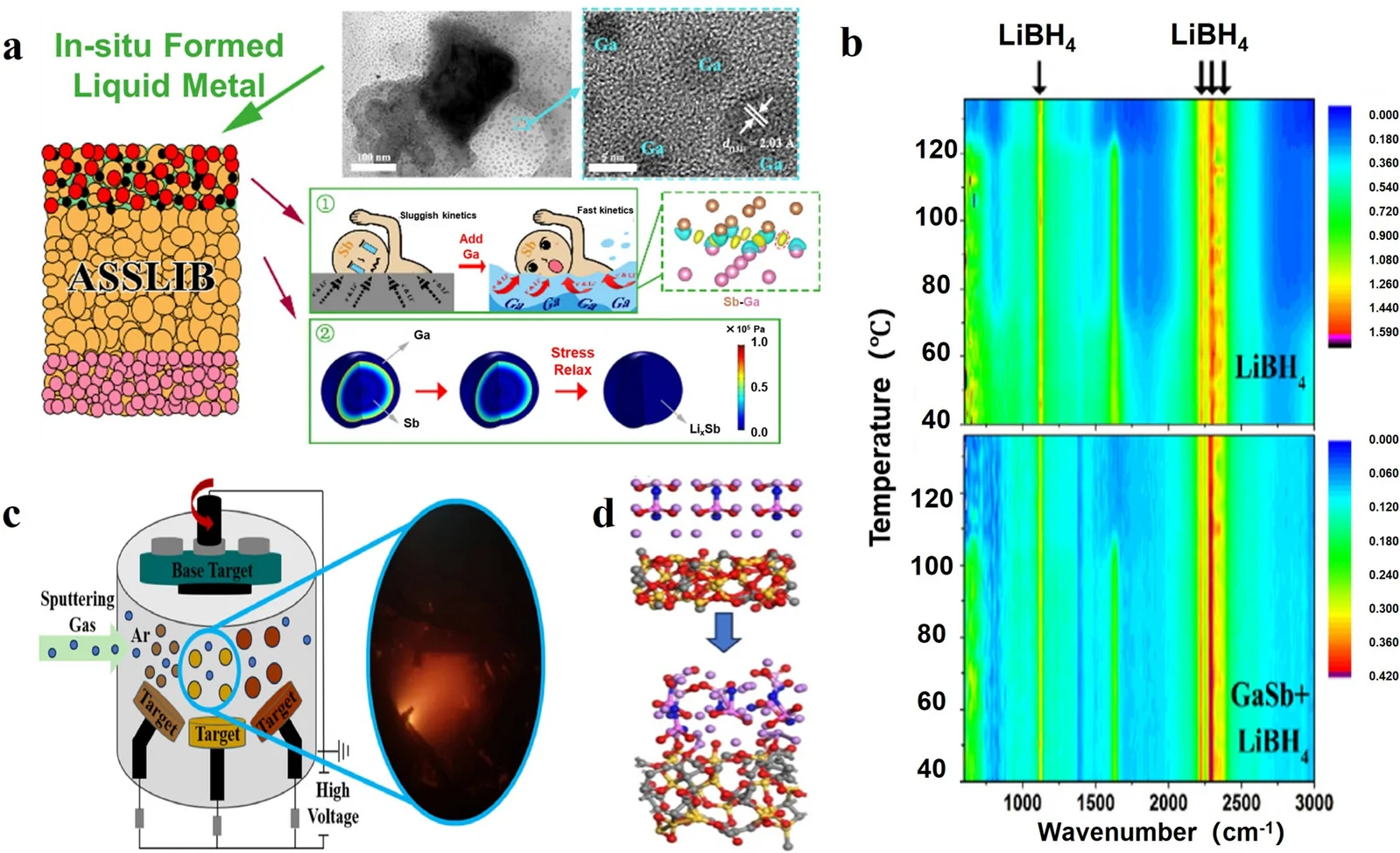
**a** Morphology and mechanism of ASSLIB. **b** In situ FTIR spectra of LiBH_4_ and the GaSb/C/LiBH_4_ composite across a temperature range of 40 to 136 °C [318]. © 2020 American Chemical Society. **c** Schematic diagrams of preparation of ASSTFB. **d** Atomic arrangements and charge density variations for LiAlPON/SiCO composites [322]. © 2025 Elsevier B.V

In the study of alloy-type anode solid electrolytes, in addition to the common silicon-based materials, other alloy-type anodes also have many interface problems between electrodes and solid electrolytes. Studies have found that traditional Sb electrodes have serious interface contact problems during the cycle with LiBH_4_ solid electrolytes. This is mainly due to the huge volume change during the alloying/de-alloying process, which leads to insufficient contact between the solid electrode and the electrolyte, thereby hindering the diffusion of lithium ions. On this basis, Mo and team introduced liquid metal Ga into Sb-based anode materials to solve the key problem of poor interface contact between solid electrolyte and electrode in all-solid-state lithium ion batteries (Fig. 22a) [318]. In situ FTIR analysis confirmed that GaSb/C and LiBH_4_ electrolyte had good thermodynamic stability at an operating temperature of 125 ℃ and would not undergo harmful chemical reactions (Fig. 22b). The introduction of liquid metal Ga played a multiple interface improvement role: first, as a wetting agent, it improved the solid electrode/electrolyte interface contact. Secondly, its fluidity was used to achieve the characteristic of self-repair, and when the interface integrity was mechanically damaged due to volume change, it could instantly self-heal. Third, finite element analysis proved that liquid Ga, as a stress release phase, could promote stress relaxation and alleviated the mechanical degradation of anode materials. Electrochemical impedance spectroscopy tests showed that the internal resistance of the GaSb electrode remained stable throughout the charge-discharge process, maintaining approximately 23 Ω, indicating close contact between the electrode and the LiBH_4_ electrolyte. In contrast, the internal resistance of the pure Sb electrode fluctuated between 18 and 29 Ω. This improved interfacial compatibility significantly reduced the charge transfer impedance of the GaSb electrode at the electrode/electrolyte interface, ensuring excellent rate performance. Furthermore, this liquid metal-assisted technology was universally applicable and compatible with various solid-state electrolyte systems. In addition to Ga, the liquid metal material for alloy anodes often also utilizes self-healing Ga-In liquid metal. Pure Ga has a melting point of approximately 30 ℃ and requires temperatures slightly above room temperature to remain liquid. However, the addition of In to form a Ga-In eutectic alloy lowers the melting point to below room temperature, allowing it to remain stable in a liquid state without the need for additional heating. Therefore, in terms of ease of application, Ga-In alloys are more practical in room-temperature applications due to their lower melting point. Furthermore, traditional silicon- and tin-based solid electrode materials undergo significant volume expansion and contraction during lithiation/delithiation, leading to deteriorated interfacial contact with the solid electrolyte, which in turn affects ionic conduction and electrochemical performance. To address this interfacial compatibility issue, Wang and team employed self-healing Ga-In liquid metal as the anode material [319]. The study found that the Ga-In liquid metal exhibited a contact angle of 134.9° with the Li_6_PS_5_Cl solid electrolyte. While its wettability was not ideal, its fluidity and self-healing properties enabled it to maintain stable interfacial contact with the solid electrolyte. Compared to conventional solid anode materials, which require stacking pressures of up to 200 MPa to maintain interfacial contact, Ga-In liquid metal maintained excellent electrochemical performance at lower pressures (1–20 MPa), effectively alleviating the mechanical stress caused by volume change and improving interfacial stability and cycling performance.

As an important multifunctional solid electrolyte interface material, Li_3_PO_4_ has good chemical and electrochemical stability and is often used as a protective layer or buffer layer in lithium-ion batteries. The lithium ion conductivity of this material is about 10^−7^ S m^−1^, which is relatively lower but sufficient to maintain ion transport [320]. The Li_3_PO_4_ layer can effectively prevent direct contact between the electrode material and the electrolyte, preventing adverse interfacial reactions, while allowing the transport of lithium ions. In all-solid-state batteries, Li_3_PO_4_ is often used as an artificial SEI layer, which can provide mechanical buffering, reduce the stress caused by volume change, and protect the active material from the effects of electrolyte decomposition. Li et al. [321] used an innovative method of electrochemically converting micron-sized BiPO_4_ precursors to in situ generate a Li_3_PO_4_ transition layer to protect bismuth nanoparticles. This transition layer had mechanical stability, interface stability as well as good ion conductivity. As a common solid electrolyte, Li_2_S-P_2_S_5_ (LPS) has the defect of a limited electrochemical stability window, especially when it is prone to the reduction/decomposition reactions at low potentials, which seriously hinders the application of low-potential anode materials such as Li, Si, and graphite in LPS-based solid-state batteries. To solve this interface compatibility problem, the researchers proposed a strategy of in situ generation of a Li_3_PO_4_ transition layer by electrochemically converting micron-sized BiPO_4_ raw materials. This Li_3_PO_4_ transition layer played multiple functions at the interface. First, it acted as a solid glue to bond Bi nanoparticles together, buffering the mechanical strain caused by volume expansion/contraction and maintaining the stability of the electrode structure. Secondly, it acted as a protective layer between the highly active Li-Bi alloy and LPS, providing better interface stability. Thirdly, as a part of the solid electrolyte membrane, it provides an ion conduction channel for the electrochemical lithiation/delithiation process. In lithium symmetric battery tests, the Bi@Li_3_PO_4_ interface layer significantly improved the critical current density and extended the stable cycle time from 160 h to over 1200 h. It effectively suppressed lithium dendrite penetration and LPS side reactions, demonstrating the versatility and effectiveness of in situ transition layer construction in improving the stability of solid-state electrolyte interfaces.

Thin-film alloy anode materials are typically prepared in thin-film form through vapor deposition techniques such as sputtering, evaporation, or pulsed laser deposition. These thin-film materials are then combined with LiPON solid electrolytes to form high-performance all-solid-state thin-film lithium battery systems. This relationship stems from the unique advantages of LiPON as an amorphous electrolyte, which provides a stable solid-solid interface and alleviates the mechanical stress and interfacial instability caused by volume expansion of the alloy anode during lithiation/delithiation. LiPON is a nitrogen-doped lithium phosphate glass electrolyte prepared by RF magnetron sputtering from a Li_3_PO_4_ target in a nitrogen atmosphere. Its amorphous structure ensures close contact with the thin-film alloy anode, preventing lithium dendrite growth induced by grain boundary defects. Compared with the permeable SEI growth of liquid electrolytes, the solid interface of LiPON reduces side reactions and the capacity decay rate is also reduced by about 50%. Based on the material design framework combining first principles and experiments, Xu and team deeply explored the interface phenomena and interface compatibility issues between SiCO anode and LiPON solid electrolyte doped with different elements [322]. The study constructed a LiXPON/SiCO interface model with six doping elements (Al, Si, C, Sn, Y, Ta) through magnetron sputtering technology (Fig. 22c). The interface stability was evaluated by parameters such as interface formation energy and interface adhesion energy. It was found that the LiPON system doped with Al and Si had an interface formation energy and interface adhesion energy significantly higher than that of the pure LiPON system (Fig. 22d). After structural relaxation, the LiXPON structure underwent a certain degree of deformation when combined with SiCO. The oxygen atoms in the interface area could also adsorb lithium atoms to form Li_2_O complexes. This phenomenon was more obvious in the LiCPON/SiCO system, which was consistent with the experimental observation of the LiCPON/SiO_x_C interface behavior. Importantly, charge density difference analysis revealed that the LiAlPON/SiCO and LiSiPON/SiCO systems exhibited greater electron cloud overlap and significant charge transfer at the interface. This phenomenon is associated with the formation of lithium compounds, indicating the disappearance of a space charge insulating layer, leading to significantly improved ion transport at the interface. Analysis of interfacial electronic properties, using density-of-charge (DOS) calculations, revealed the underlying mechanism of doping's impact on interfacial performance. The introduction of doping elements significantly reduced the band gap of LiPON, particularly Al doping, which significantly decreased the band gap from 6.3 to 1.2 eV. This reduction in the band gap directly enhanced the interfacial conductivity, pushing the density of states of the interfacial system generally above the Fermi level, indicating superior interfacial conductivity compared to pure SiCO and most electrolyte monolayers. The LiYPON/SiCO system, on the other hand, retained a band gap of 1.3 eV, exhibiting semiconducting properties, consistent with its relatively poor interfacial performance calculations, further validating the effectiveness of Al and Si doping in improving interfacial performance. The diffusion behavior of lithium ions at the electrode/electrolyte interface was also investigated using Cl-NEB and MSD analysis. Interfacial lithium-ion diffusion pathways can be divided into two types: migration along the interface and diffusion into the electrode interior. Calculations indicate that most lithium ions tend to diffuse along the interface. Of particular note, the LiAlPON/SiCO interface exhibited the lowest lithium ion diffusion barrier, at only 0.2 eV. This indicates that Al and Si doping significantly reduced the resistance to lithium ion migration at the interface and enhances the diffusion rate. Importantly, interfaces with higher calculated interfacial energies typically exhibit lower interfacial impedance in experiments, attributed to the formation of a stable interfacial phase. Battery performance tests echoed theoretical predictions. Al doping significantly enhanced the capacity of the LiPON electrolyte, with the SiCO/LiAlPON/Li battery achieving an initial discharge capacity of 130.2 mAh g^−1^. The introduction of the Si layer boosted the initial discharge capacity of the SiCO/LiPON battery by 54.7%, reaching 190.1 mAh g^−1^. The capacity decrease during the initial 20 cycles was attributed to decomposition of the electrode and electrolyte due to interfacial reactions, which resulted in the formation of interfacial phases and contributes to battery capacity decay. Rate performance tests showed that although the specific capacity of the battery decreased with increasing current rate, the capacity decrease in the Al and Si doped systems was significantly smaller than that of the intrinsic system, which was attributed to the enhanced stability of the doped LiPON interface, fully demonstrating the effectiveness and accuracy of the first-principles calculation framework in interface design.

Based on the existing research results, interfacial synergistic stabilization has been identified as a key strategy for addressing interfacial compatibility issues between alloy-type anodes and solid-state electrolytes. This synergistic effect has significantly improved interfacial performance through the synergistic action of multiple mechanisms. Regarding interfacial structure optimization, TCVD technology precisely constructs an ultrathin functional interfacial layer on the surface of the polymer composite electrolyte, achieving excellent bonding strength and chemical compatibility between the in situ deposited interfacial layer and the polymer matrix. This effectively mitigates chemical and electrochemical incompatibilities between the polymer electrolyte and electrode materials, inhibits interfacial side reactions, and reduces interfacial impedance growth. In terms of improving interfacial chemical stability, the in situ construction of the Li_3_PO_4_ transition layer performs multiple functions: acting as a solid glue to bind the nanoparticles together, buffering the mechanical strain caused by volume expansion; acting as a protective layer between the highly active alloy and the solid electrolyte, providing improved interfacial stability. The self-pressure structure design achieves dynamic adaptive contact at the interface. The compressed carbon nanotube network was coated with ion-conductive PEO/LiTFSI to form an electron-ion hybrid conductive network. When the active material expanded in volume, it provided mechanical buffering and maintained electron and ion transport channels. Liquid metal-assisted technology introduced Ga or Ga-In alloys with fluidity and self-healing properties, utilizing their wetting agent effect to improve the solid-state electrode/electrolyte interface contact. When the interface integrity was mechanically damaged by volume changes, it could instantly self-heal. At the same time, it acted as a stress release phase to promote stress relaxation and alleviated the mechanical degradation of the anode material, so that the interface impedance could remain stable throughout the charge and discharge process. Surface morphology manipulation involved forming a groove structure on the silicon wafer surface through KOH etching, thereby significantly improving the interfacial contact with the solid electrolyte. The grooves could penetrate the highly ductile electrolyte under battery manufacturing pressure to form a more conformal and intimate interfacial contact. This not only increased the reaction area, but also optimized the uniformity of the lithiation current distribution, giving the grooved silicon a low and consistent contact impedance and maintaining low impedance and interfacial integrity throughout the cycle.

#### Mechanical Strength Optimization

**Fig. 23:**
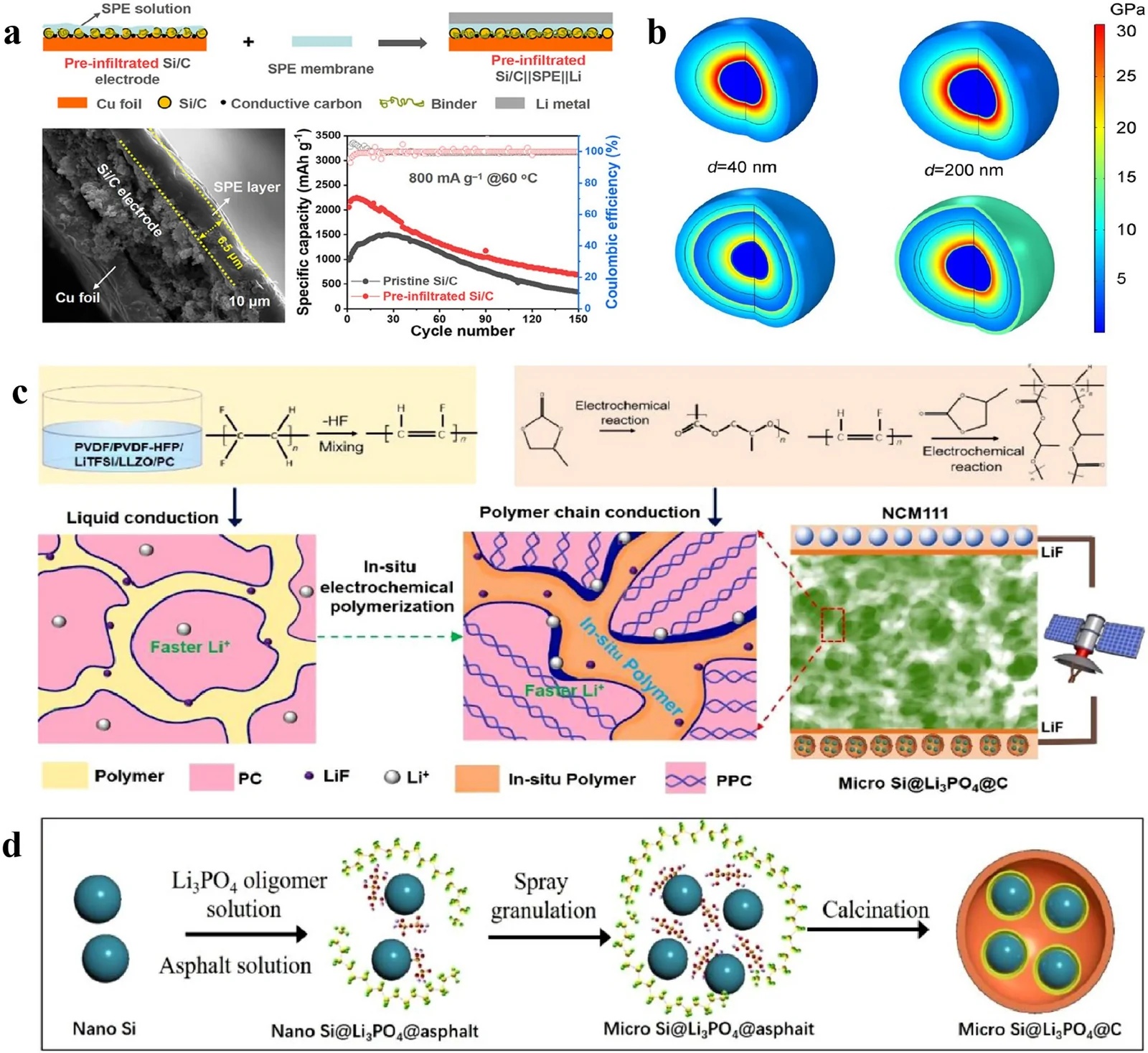
**a** Structure of solid-state lithium batteries incorporating a pristine Si/C electrode and SPEs, the HRTEM of SPE layer and cycling behavior of fresh and pre-treated Si/C electrodes [325]. © 2024 American Chemical Society. **b** Simulation and stress analysis of Si and Si@MgO particles with diameters of 40 nm and 200 nm [256]. © Youke Publishing Co., Ltd. 2023. **c** Illustration of the structure of solid-state batteries. **d** Diagram depicting the micro Si@Li_3_PO_4_@C fabrication procedure [329]. © 2021 Published by Elsevier B.V

The mechanical properties of solid polymers are mainly determined by factors such as their molecular structure, crystallinity, molecular weight, and degree of crosslinking [323, 324]. Solid polymers have moderate mechanical strength, with tensile strength usually ranging from a few MPa to tens of MPa, which is much lower than that of metal materials but higher than that of gel polymers. Their elastic modulus is relatively low, usually between tens of MPa and several GPa, showing good flexibility and bendability. Solid polymers have good ductility, with elongation at break reaching 100–1000%, which makes them less prone to brittle fracture when subjected to mechanical deformation. The mechanical strength of crystalline polymers is usually higher than that of amorphous polymers, but their flexibility is relatively poor. Although amorphous polymers have lower strength, they have better flexibility and processing properties. Increasing the degree of crosslinking can significantly improve the mechanical strength and dimensional stability of the polymer, but will reduce its flexibility. Temperature has a significant effect on the mechanical properties of solid polymers, with increasing temperature leading to a decrease in strength but an increase in flexibility. In general, solid polymers have good flexibility while maintaining a certain mechanical strength. The property gives them unique advantages in applications such as flexible electronic devices and thin film materials. Dong and research team deeply explored the key influence of the mechanical properties of PEO-based solid electrolytes on the performance of all-solid-state lithium-ion batteries, especially in silicon-based anode applications [325]. The specific experiment prepared two PEO-based electrolytes, a pure PEO-LiTFSI electrolyte and a PEO-IL electrolyte added with ionic liquid plasticizer. At an operating temperature of 60 ℃, the mechanical stability of the two electrolytes showed completely different characteristics. The PEO-LiTFSI membrane was able to maintain its original shape and structural integrity, while the PEO-IL electrolyte became viscous and melted. This difference in mechanical stability directly affected the electrochemical performance of the battery. Although the PEO-IL electrolyte performed better in terms of ionic conductivity, its poor mechanical stability severely limited its application performance at high current density. In contrast, the PEO-LiTFSI electrolyte, with its superior mechanical stability, maintained a reversible specific capacity of 1300 mAh g^−1^ at a high current density of 800 mA g^−1^, demonstrating excellent electrochemical performance (Fig. 23a). Through anatomical analysis of the cycled battery, the researchers further verified the critical role of mechanical stability in battery performance. After rate testing, the electrolyte membrane of the battery using PEO-LiTFSI retained its original shape, while the PEO-IL electrolyte membrane was almost unobservable. It was due to deformation caused by its poor mechanical stability at 60 °C. Cross-sectional morphology observations using a scanning electron microscope revealed a large gap between the PEO-IL electrolyte and the lithium metal, providing direct evidence of deformation caused by insufficient mechanical stability during cycling, which in turn led to increased interfacial impedance. This study revealed that at an operating temperature of 60 °C, the mechanical stability of the PEO-based electrolyte, rather than ionic conductivity, is a more dominant factor in the electrochemical performance of silicon-carbon composite electrode solid-state batteries. This discovery has important guiding significance for the design of solid-state electrolytes, indicating that while pursuing high ionic conductivity, the mechanical properties of the material must be fully considered, especially when facing active materials such as silicon-based anodes that undergo significant volume changes.

Electrolytes with insufficient mechanical strength are prone to deformation and structural damage during battery cycling, leading to interruption of ion conduction paths and deterioration of interfacial contact, which ultimately seriously affects the long-term cycle stability and rate performance of the battery. This provides important design criteria and performance balance considerations for the subsequent development of solid electrolyte materials [326]. Unlike traditional liquid batteries, the low permeability of solid electrolytes can avoid side reaction problems and continuous SEI film formation, but the huge stress generated by alloy materials during the lithiation-delithiation process, resulting in particle breakage and conductivity loss, becomes a major problem [327]. To this end, Han and group designed a unique Si@MgO@C composite structure, in which the MgO coating plays a key role as a stress release layer [256]. The researchers found that the MgO coating can effectively improve the stress distribution of silicon particles (Fig. 23b). At 50% charge state, the stress of uncoated silicon particles at the lithiation edge was as high as 30 GPa, while the MgO coating made the stress more evenly distributed in the LiSi_x_ region and the MgO-LiSi_x_ interface, reducing the stress of 40 nm Si@MgO particles to below 20 GPa and the stress of 400 nm particles to 25 GPa. The MgO layer effectively released stress from silicon and reduced the volume expansion of silicon particles during repeated lithiation. The in situ EIS allowed real-time recording of very sensitive interfacial chemical stability and dynamics. The PEO/LATP/NCF composite solid electrolyte used by the researchers achieved an ionic conductivity of 1.68 × 10^−4^ S cm^−1^ at 50 °C and a lithium-ion transference number of 0.43. Good interfacial compatibility was demonstrated in lithium-lithium symmetric battery tests, with stable cycling for 1000 h at a current density of 0.05 mA cm^−2^. The solid electrolyte effectively suppressed the volume expansion of the Si@MgO@C electrode, significantly reducing it from 49% in the liquid battery to 23%. Meanwhile, the SEI impedance remained relatively stable, decreasing from an initial 192 to 156 Ω at the end of delithiation. A uniform and dense SEI layer, approximately 20 nm thick, formed in the solid-state battery. This layer was primarily composed of LiF and LiTFSI salts, with a LiF content of up to 63.4%. This LiF-rich SEI exhibited excellent mechanical flexibility, effectively releasing mechanical stress and maintaining structural stability.

##### Polymer-Based and Oxide-Based Solid Electrolytes

PEO and PVDF are both the main substrates of polymer-based solid electrolytes, which play the role of conducting ions and isolating positive and anodes in lithium batteries. Both belong to polymer electrolyte systems and require the addition of lithium salts to provide ionic conductivity. They have good mechanical flexibility and processing properties and can form stable electrolyte membranes. However, the difference is that PEO has polar ether oxygen groups, which can dissolve lithium salts and coordinate with lithium ions. The ionic conductivity is relatively high, but there are problems such as high crystallinity and insufficient room temperature ionic conductivity. Although PVDF has better mechanical strength and thermal stability, due to the lack of polar groups in the molecular chain, it is almost non-ion conductive. It usually requires the addition of a large amount of plasticizer or preparation into a gel electrolyte to obtain sufficient ionic conductivity [328]. Therefore, PEO is more suitable as the main material of solid electrolytes, while PVDF is more commonly used as a supporting matrix or in combination with other conductive components. Both have their own advantages in application, and the choice depends on the specific performance requirements. Pan and research team mainly focused on the mechanical strength optimization and stress management of PVDF-based composite polymer electrolytes (Fig. 23c) [329]. The main reason why the team chose PVDF/PVDF-HFP as the polymer matrix was its excellent mechanical strength and dielectric constant, which are conducive to ion transport. At the same time, PVDF-based materials have excellent hydrophobicity, which makes their environmental stability excellent. However, the high crystallinity of PVDF limits its ionic conductivity, so the researchers reduced the crystallinity by copolymerizing with hexafluoropropylene (HFP), improving the ionic conductivity while maintaining good mechanical strength. To further improve the mechanical properties, the researchers introduced fast ion conductor garnet-type LLZTO ceramic filler into the PVDF/PVDF-HFP matrix. The addition of this inorganic filler not only provided a transmission channel for lithium ions, but also improved the ionic conductivity by reducing the crystallinity of PVDF without losing its good mechanical strength and contact performance with the electrode. The improvement in mechanical tensile strength was attributed to the uniform distribution of LLZTO, which reduced agglomeration, and the synergistic effect of adding LLZTO and PC plasticizer. In terms of mechanical properties, traditional plasticizers such as propylene carbonate (PC), while significantly improving ionic conductivity, can lead to a significant loss of mechanical strength and pose safety risks. This is because the presence of plasticizers as free solvents weakens the mechanical integrity of the polymer matrix. To address this conflict, researchers designed an in situ polymerization mechanism. In situ FTIR spectroscopy and MNR confirmed that electrochemical polymerization occurs between PC and PVDF/PVDF-HFP during the first discharge. PVDF undergoes dehalogenation at open circuit voltage to form C = C double bonds. Subsequently, an addition reaction with PC occurs when voltage is applied, while PC undergoes ring-opening and complete self-polymerization. The key advantage of this in situ polymerization process is that it maintains a high ionic conductivity of 2.9 × 10^−4^ S cm^−1^ while significantly improving mechanical strength and eliminating the safety risks of liquid plasticizers. The main advantages of polymer solid electrolytes over other types of solid electrolytes are their excellent flexibility and improved electrode contact properties, which can effectively alleviate the mechanical stress caused by volume changes in the electrodes during charge and discharge. Manufacturing workflow for Micro-Si@Li_3_PO_4_@C components in solid-state battery applications is depicted in Fig. 23d. Especially when paired with a silicon anode, the silicon material undergoes a huge volume change during the lithiation-delithiation process. Traditional rigid inorganic electrolytes have difficulty adapting to this deformation, while flexible polymer electrolytes can relieve interfacial stress through their own deformation and maintain good interfacial contact. In addition, the LiF-rich interfacial layer not only has good chemical stability, but also excellent mechanical flexibility, which can effectively inhibit the growth of lithium dendrites and maintain the mechanical integrity of the interface. Unlike previous studies of PVDF-garnet-based composite electrolytes that are prone to severe chemical dehydroflourination, when cycling with lithium metal, the in situ polymerization mechanism can inhibit the unlimited dehydrofluorination reaction of PVDF/PVDF-HFP, generating a stable LiF layer interface, thereby maintaining the mechanical stability and electrochemical performance of the electrolyte.

Different from other methods, electrospinning can stretch polymer solutions or melts into continuous fibers with diameters ranging from tens of nanometers to several micrometers. These fibers have a large aspect ratio, and the fiber membranes formed have high porosity and a three-dimensional interconnected porous network structure [330]. The biggest advantage of electrospinning is that it can produce nanofiber materials with ultra-high specific surface area. The unique micromorphology allows the specific surface area of the material to reach tens or even hundreds of square meters per gram, which is several orders of magnitude higher than that of materials prepared by traditional methods. The advantages brought by ultra-high specific surface area include providing more active sites for catalytic reactions, enhancing adsorption capacity for separation and purification, improving electrochemical performance for energy storage, and promoting cell adhesion for biomedical applications [331]. The feature makes electrospun nanofibers exhibit excellent performance in many fields such as catalysis, adsorption, filtration, sensing, battery electrodes, and tissue engineering. This is also the fundamental reason why electrospinning technology has attracted much attention and has been widely used. Liu et al. [332] prepared PAN or PMMA-based polymer solid electrolytes by liquid phase polymerization and electrospinning technology for use in silicon anode energy storage systems to achieve high energy density and excellent reliability. The polymer-based material exhibited a low Young's modulus and high ductility, enabling it to better accommodate the volume change of the silicon anode during ion intercalation, preventing electrode fragmentation and interfacial separation. The nanofiber membrane, fabricated via electrospinning, formed a three-dimensional interconnected framework. The porous fiber morphology significantly enhanced tensile strength and released stress through elastic deformation between fibers, ensuring a capacity retention greater than 90% after 500 cycles. It enhanced mechanical strength was attributed to the flexible crosslinking of the polymer chains. During liquid-phase polymerization, the coordination of the chain segments with the lithium salt formed a semicrystalline structure that balanced ion transport efficiency and mechanical toughness. Compared to the planar structure of conventional cast membranes, the stress-strain curves of the fiber-spun membranes showed no significant plastic deformation at strains of 10%–20%, with a recovery rate exceeding 95%. This helped suppress mechanical failure of the silicon anode, such as crack propagation and particle delamination. Importantly, the research team demonstrated the excellent damage resistance of the polymer solid electrolyte through extreme tests such as heating, external short circuiting, overcharging, nail penetration, and cutting. First, the battery could still light up the LED lamp normally at a high temperature of 100 °C, indicating that its thermal stability and structural strength were significantly better than those of liquid electrolyte batteries. Secondly, in the external short circuit and nail penetration experiments, only a slight local temperature rise occurred without thermal runaway or electrolyte leakage, indicating that the polymer solid electrolyte has high puncture resistance and high modulus support. More importantly, the battery could still cycle after being cut or even directly immersed in water, indicating that the polymer solid electrolyte could not only maintain electrode bonding, but also ensured the continuity of the conductive phase and interfacial bonding after external mechanical fracture. The above phenomena verified its ability to maintain ion conduction and structural robustness under stress, strain, and mechanical damage environments. Overall, the mechanical properties of polymer materials are better than the rigidity of ceramic materials, but particle optimization is required to balance strength and flexibility. Future improvements may include cross-linking networks to further increase the modulus to 5 GPa or introducing self-healing polymers to repair cycle-induced microcracks.

**Fig. 24:**
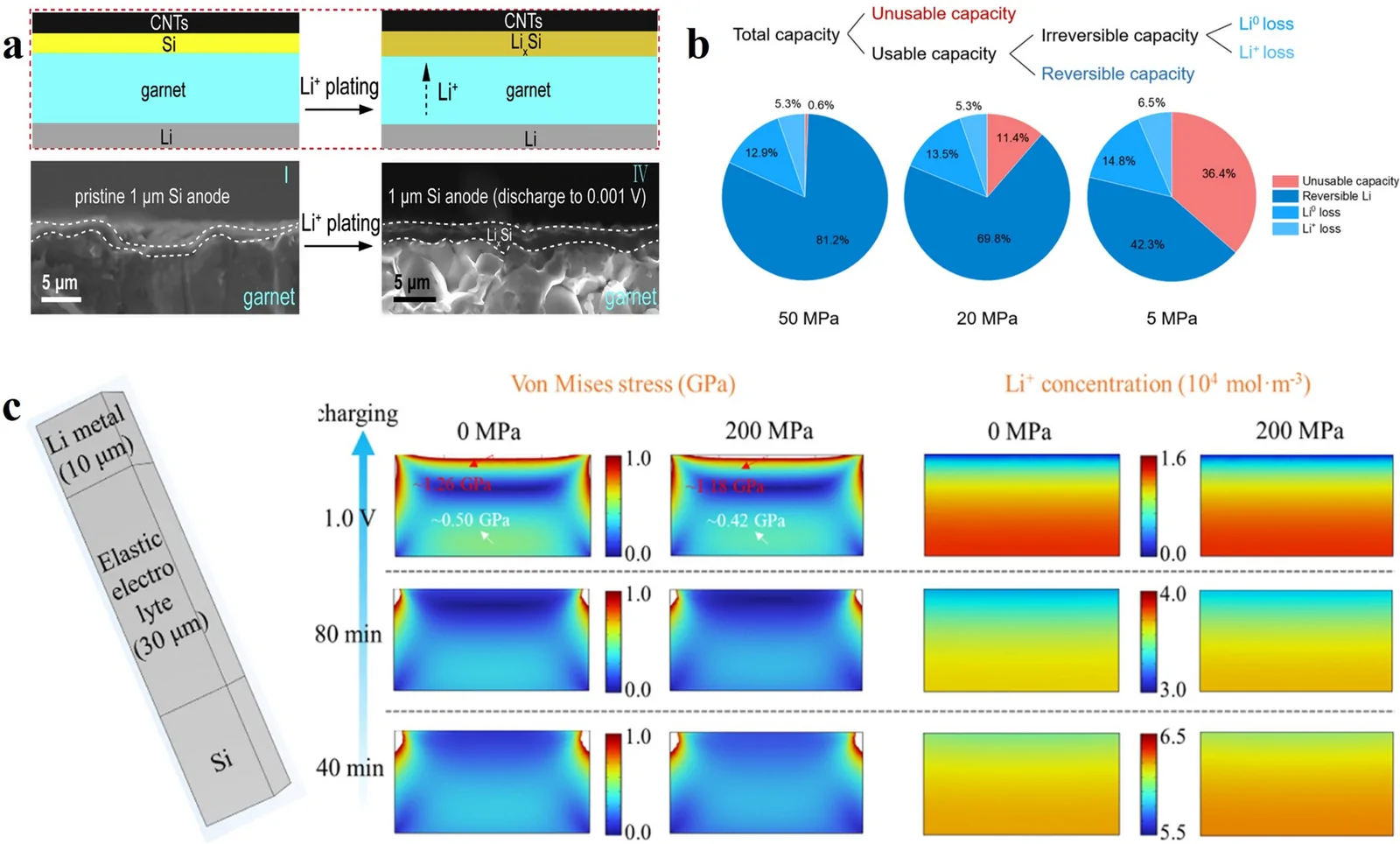
**a** Structural evolution of the Li/garnet/Si cell during discharge and its associated discharge profile and voltage-capacity curve of the solid-state Si anode during discharge [246]. © 2019 Elsevier B.V. All rights reserved. **b** Capacity distribution of μ-Si under stacking pressures of 50, 20, and 5 MPa, derived from TGC experiments [257]. © 2024 Elsevier B.V. All rights reserved. **c** Profiles of Li^+^ concentration and Von Mises stress at 40 min, 80 min, and 1.0 V, under no external pressure or with 200 MPa, for 10 μm Si anodes during charging [258]. © 2023 Elsevier B.V. All rights reserved

As a typical ceramic material, oxide solid electrolytes have mechanical properties characterized by high strength, high hardness but poor toughness. Their compressive strength is usually in the range of several hundred MPa to more than 1 GPa, and their flexural strength is generally in the range of 100–300 MPa, showing excellent load-bearing capacity [333]. The elastic modulus is relatively high, usually between 100 and 200 GPa, far exceeding that of polymer and sulfide electrolytes, showing high rigidity and dimensional stability. However, the biggest disadvantage of oxide solid electrolytes is their obvious brittleness, extremely low elongation at break, poor impact toughness, and easy brittle fracture rather than plastic deformation under external force. The brittle characteristic makes it challenging in battery applications, especially during the charge and discharge cycle, when the volume change of alloy electrode materials will generate stress on the electrolyte, which may lead to deterioration of interfacial contact or cracking of the electrolyte [334]. Different types of oxide electrolytes have slightly different mechanical properties: garnet type has relatively high mechanical strength, perovskite type has medium strength, and nasicon type has slightly better toughness under certain conditions. In order to improve mechanical properties, strategies such as particle refinement, composite, and interface engineering are often used, or composite electrolytes are prepared by combining with polymers to balance the relationship between strength and toughness. Ping and team members revealed the nanomechanical confinement effect of solid electrolytes through experiments and theoretical modeling [246]. The mechanical confinement effect of garnet electrolyte was reflected in two key aspects: First, the lithium-ion concentration and stress distribution inside the silicon anode remain relatively uniform in the thickness direction, ensuring the stability of the overall structure. Secondly, although the stress in the silicon anode surface changed periodically during charging and discharging and produced fatigue fracture risks on the surface, the mechanical confinement of garnet effectively inhibited such damage. Under garnet confinement, the maximum crack opening displacement of the silicon anode when it is completely delithiated was much smaller than the corresponding value in the liquid battery, which proved the significant advantage of solid electrolytes in reducing structural damage to the silicon anode. During the delithiation process, the crack driving force of the garnet-constrained silicon anode was smaller, which is the key evidence of its higher fracture resistance. The strong and tough silicon/garnet interface effectively protected the structural integrity of the silicon anode by suppressing the volume change during charging and discharging. Theoretical analysis showed that tunnel cracking was the primary failure mode of silicon anodes, and the steady-state energy release rate increased linearly with thickness, making thicker silicon anodes susceptible to cracking during the delithiation phase. However, a comparison of the mechanical states of silicon anodes with and without garnet confinement confirmed that garnet nanomechanical confinement significantly enhanced electrode durability. A 1-micron-thick silicon anode exhibited a high discharge capacity of 2685 mAh g^−1^ and an initial Coulombic efficiency of 83.2% in garnet-based solid-state batteries, significantly outperforming its performance in organic electrolytes. As the silicon anode thickness increases to 2 and 3 μm, although it initially maintained good contact with the garnet electrolyte, it gradually separated and pulverized during deep discharge, confirming the existence of an optimal thickness range (Fig. 24a). This study not only experimentally demonstrates the feasibility of thick silicon anodes in solid-state batteries but, more importantly, reveals from a mechanical perspective how the mechanical confinement effect of solid-state electrolytes fundamentally changes the design criteria for electrode materials.

##### Traditional Sulfide-Based Solid Electrolytes

The biggest advantage of sulfide solid electrolytes is their relatively good plasticity and processing properties. Compared with oxide electrolytes, sulfides have better deformation ability and can undergo plastic deformation under a certain pressure. Although the elongation at break is still small, it is significantly better than that of oxide materials. This property enables them to obtain better inter-particle contact through cold pressing, which is beneficial to reducing interface impedance [278]. There are differences in the mechanical properties of different types of sulfide electrolytes: the Li_2_S-P_2_S_5_ series is relatively soft and easy to process but has lower strength; halide sulfides such as Li_6_PS_5_Cl have slightly higher strength; and materials such as Li_3_PS_4_ are between the two. The main challenge of sulfide electrolytes is that they are easily decomposed in a humid environment to produce toxic H_2_S gas, which limits their processing and application conditions [335]. Overall, sulfide electrolytes have both certain mechanical strength and processing properties and are important candidate materials for the preparation of all-solid-state batteries. Cao and team members used Li_6_PS_5_Cl sulfide solid electrolytes and focused on studying the mechanical response characteristics of solid electrolytes under different stacking pressures (5, 20, and 50 MPa) (Fig. 24b) [257]. Research has found that solid-state batteries require high stacking pressures of tens of MPa to fully utilize the capacity of the silicon anode, a pressure requirement far exceeding the acceptable range for practical applications. The researchers observed that increasing the stacking pressure from 5 to 50 MPa significantly reduced the impedance at the solid electrolyte-electrode interface, indicating that high pressure improves solid-solid interfacial contact. However, they found that this impedance improvement alone is insufficient to explain the significant capacity difference, as at a low current density of 0.1 mA cm^−2^, 200 Ω impedance difference only produces a polarization change of approximately 16 mV. A more critical finding is the effect of stacking pressure on the electrochemical-mechanical coupling effect. During the lithiation process of silicon particles, a volume change exceeding 300% generated intrinsic stress. However, simulations showed that the local stress accumulation caused by silicon expansion is still far smaller than the effect of the applied stacking pressure, indicating that local stress is primarily determined by the applied pressure. The stress-induced overpotential at 50 MPa is an order of magnitude greater than that at 5 MPa. This overpotential difference directly affects the local reaction current, suppressing the reaction current in the high-pressure region, thereby preventing the growth of Li_x_Si protrusions and maintaining a smooth interface between the solid electrolyte and the anode material. At a pressure of 50 MPa, the lithiation reaction was more uniform, with a smoother and denser interface. However, at a pressure of 5 MPa, the reaction exhibited significant inhomogeneity, resulting in a heterogeneous distribution with both high and low concentrations. Phase-field modeling further revealed that at low pressure, the interfacial contact impedance dominated the current distribution, and the stress overpotential was negligible. This accelerated the growth of Li_x_Si protrusions, making the lithiation reaction increasingly non-uniform, with a standard deviation of the current distribution reaching 2.1 mA cm^−2^. At high pressure, however, stress not only enhanced the interfacial contact between the electrolyte and the electrode but also suppressed the reaction current at stress accumulation points, resulting in a more uniform current distribution with a standard deviation of only 0.9 mA cm^−2^. The core of this mechano-electrochemical coupling effect lied in the fact that high stacking pressure generated a sufficiently large local overpotential, forcing the lithiation reaction to propagate to the surrounding area, thereby flattening the entire silicon electrode surface.

**Fig. 25:**
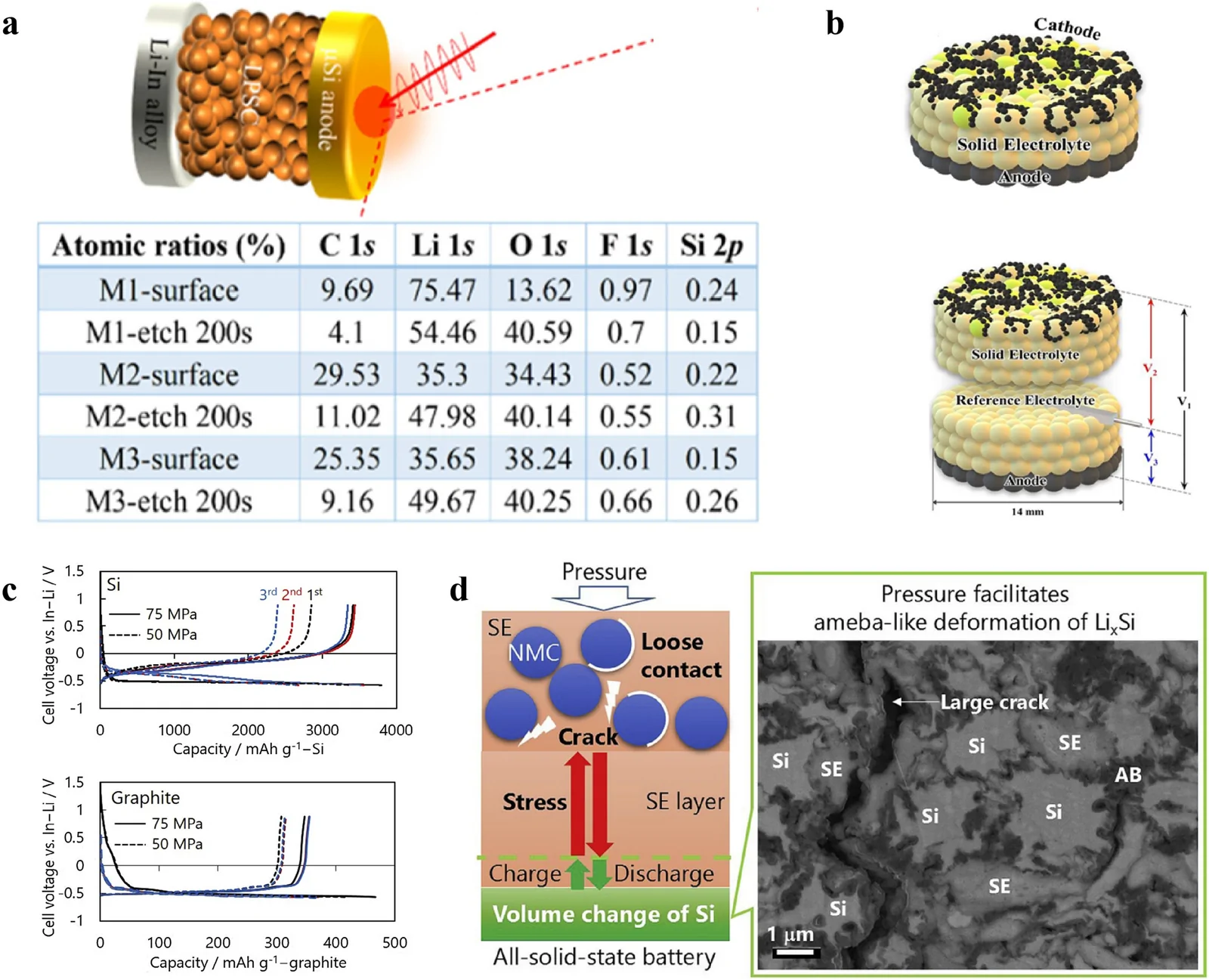
**a** Overall atomic ratios of elements in the electrodes and high-resolution XPS spectra for C 1*s* and Si 2*p* [258]. © 2023 Elsevier B.V. All rights reserved. **b** Diagram and SEM micrograph of constructed two-electrode ASSB (without RE) and three-electrode ASSB (with RE) [337]. © 2022 Elsevier B.V. All rights reserved. **c** Voltage-capacity curves for the first three cycles of Si/Li–In and graphite/Li–In half-cells. **d** Pressure mechanism of all-solid-state battery; [340] © 2020 Elsevier B.V. All rights reserved

Studying the mechanical response characteristics of solid-state electrolytes is a key step in the development of all-solid-state battery technology. Differently though, Tao and research team explored in detail the mechanical behavior of solid electrolyte Li_6_PS_5_Cl under different mechanical loads and external pressure conditions and its impact on battery performance [258]. The team members systematically analyzed the stress-strain evolution law in the solid electrolyte system, when the thickness of the silicon anode changed from 5 to 15 μm by combining experiments and simulations. This study focused on two key stress types: hydrostatic stress and Von Mises stress. The former determines the stress-induced chemical potential, and the latter is a deformation evaluation criterion. When it exceeded the yield strength of the material, it leads to plastic failure. Research has found that the lithium-ion diffusion path lengthened with increasing silicon anode thickness, forming a significant lithium-ion concentration gradient within the electrode. It directly leads to a hydrostatic stress gradient proportional to the concentration (Fig. 24c). Specifically, when discharged to 0.01 V, the lithium-ion concentration and hydrostatic stress near the center of the current collector-electrolyte boundary for a 15 μm-thick silicon electrode were 21.32 × 10^5^ mol m^−3^ and 3.40 GPa, respectively, while on the electrolyte side, they reached 1.44 × 10^5^ mol m^−3^ and 10.67 GPa, respectively. This uneven lithium-ion concentration and hydrostatic stress distribution lead to significant spatial variations in the Von Mises stress in thicker electrodes. The Von Mises stress in thick electrodes tended to reach a minimum at the center, while the stress values at positions 1/4 and 3/4 of the distance from the current collector boundary to the electrolyte boundary became important indicators for evaluating overall electrode performance. In short, the effect of applied pressure on the mechanical properties of solid-state electrolytes exhibited complex characteristics. This study compared battery performance under applied pressures of 100, 200, and 300 MPa and found that applied pressure primarily reduced cracking by lowering the Von Mises stress, thereby ensuring the stability of electron/ion transport pathways. Under an applied pressure of 200 MPa, a 10 μm silicon anode was able to discharge for 133.8 min and charge to 1.0 V in 116.9 min. However, compared to the hydrostatic stress of up to 10 GPa, the effect of an applied pressure of 200 MPa was relatively limited. This suggests that applied pressure has little intrinsic effect on lithium-ion diffusion, primarily maintaining particle connectivity to ensure low ohmic impedance. Maintaining efficient ion/electron conduction in the anode to ensure low concentration gradients is key to achieving optimal battery performance at low applied pressures of less than 10 MPa. This differs from the most effective modification methods for silicon anodes in liquid batteries. According to the difference in XPS before and after etching, it can be seen that there is a thick layer of Li–Si–O coating on the surface of M1-μSi (Fig. 25a). Based on the premise of not destroying the fusion of silicon boundaries, the study suggests adopting strategies such as a three-dimensional interconnected silicon and solid electrolyte hybrid structure that shortens the ion-electron transmission path, improving the ionic and electronic conductivity of silicon or introducing high-conductivity additives, and reducing the volume expansion rate of silicon or limiting the lithiation capacity to improve cycling performance. These findings provide in-depth insights into the electrochemical-mechanical coupling mechanism in solid-state batteries and emphasize the importance of minimizing the lithium-ion concentration gradient for achieving low-applied-pressure solid-state batteries.

##### Sulfide-Based Solid Electrolytes with Different Raw Material Ratios

Sulfide solid electrolytes prepared with different molar ratios of Li_2_S and P_2_S_5_ show significant differences in mechanical properties. When the P_2_S_5_ content is high, more P-S bonds and polyanion units such as P_2_S_7_^4−^ are formed in the material, resulting in a more rigid network structure, relatively high mechanical strength, and increased elastic modulus, but at the same time the material becomes more brittle and its plastic deformation ability decreases [336]. Introducing a lower content of P_2_S_5_ will reduce the proportion of sulfide solid electrolytes in the system, thereby weakening the passive buffering capacity of traditional sulfide electrolytes for the volume expansion of active materials. However, this low-content design instead enhances the mechanical restraint effect of the rigid framework, promoting higher local stress gradients in the active materials during lithiumization. The creep effect, through time-dependent plastic deformation, dynamically fills the pores inside the electrode and forms a tight solid-solid contact interface, thereby alleviating stress concentration and inhibiting particle fragmentation. Insufficient P_2_S_5_ content will reduce the number of non-bridging sulfur atoms in the Li^+^ conduction path, lowering the ionic conductivity. Therefore, it is necessary to precisely control the proportion of P_2_S_5_ to balance the ionic conductivity and interface stability, which is crucial for the long-cycle performance of all-solid-state batteries. Oh and team have deeply explored the mechanical properties of Li_2_S-P_2_S5 glass electrolytes in all-solid-state batteries, especially the mechanism of the influence of its redox activity on chemical-mechanical failure [337]. Sulfide solid electrolytes have unique mechanical advantages over oxide electrolytes, including lower electronegativity and good toughness mechanical properties, which make them feasible in battery construction, while exhibiting high ionic conductivity and low grain boundary resistance at room temperature. However, in practical applications, the mechanical stability of LPS electrolytes is seriously challenged by multiple factors. When the battery was cycled in a wide voltage range of 0.5–3.7 V, the oxidative decomposition of LPS not only lead to chemical degradation of the electrolyte itself, but more importantly, it triggered a series of mechanical failure problems. The team conducted detailed studies using a three-electrode system and found that when the charge voltage reached 3.7 V, LPS underwent significant oxidative decomposition, forming highly insulating products such as P_2_S_5_. These decomposition products significantly increased the charge transfer resistance of the positive electrode. More seriously, this electrolyte oxidative decomposition induced the growth of dendritic lithium metal on the anode side. These dendritic lithium metals penetrated the electrolyte layer, forming micro-shorts and causing persistent abnormal voltage fluctuations within the battery. This micro-short phenomenon not only disrupted the normal operation of the battery but also generated significant mechanical stress within the electrolyte layer. With cycling, the continued growth of lithium dendrites and the further decomposition of LPS formed a vicious cycle, leading to significant crack propagation in the electrolyte layer. The generation of mechanical stress involved multiple factors. Firstly, the high-capacity active material underwent significant volume changes during charge and discharge, generating periodic mechanical stress on the electrolyte layer. Secondly, the oxidative decomposition products of LPS had different densities and volumes, resulting in uneven stress distribution within the electrolyte. Thirdly, the growth of dendritic lithium metals exerted localized mechanical stress on the electrolyte material, causing plastic deformation of the matrix and the initiation of microcracks. Finally, the formation of reduction products such as Li_2_S was accompanied by volume expansion, which generated additional tensile stress inside the electrolyte. The study also found that mechanical failure has a progressive characteristic. Figure 25b depicts the construction blueprint and scanning electron microscopy visualization of the assembled power source, in that order. In the first few cycles, the redox activity of LPS showed a certain reversibility, and the impedance spectrum after discharge was able to basically return to the initial state. However, with the increase in the number of cycles and the repeated occurrence of abnormal voltage fluctuations, the mechanical integrity of the electrolyte gradually deteriorated, eventually leading to irreversible capacity loss and battery failure. The progressive failure mode indicates that even within the actual stability window of the electrolyte, long-term cycling may still lead to cumulative mechanical damage. Overall, the study clearly showed that there is a strong coupling relationship between the redox activity and mechanical stability of the LPS solid electrolyte.

Under voltage conditions outside the theoretical stability window, chemical decomposition, dendritic growth, and mechanical stress form a complex network of interactions, ultimately leading to chemical-mechanical failure of all-solid-state batteries. This failure mechanism is particularly significant for all-solid-state batteries containing high-capacity electrode materials, emphasizing the importance of considering both electrochemical stability and mechanical stability in solid-state battery design [338]. As the Li_2_S content increases to a moderate ratio (such as 75Li_2_S-25P_2_S_5_), the material reaches a relatively balanced state, maintaining a certain mechanical strength while also having moderate plasticity. This component generally exhibits good compressibility and inter-particle bonding ability. A good balance between strength and plasticity can be achieved in terms of mechanical properties. Compared with components with high P_2_S_5_ content, its brittleness is significantly reduced, and it has moderate deformation ability, which is convenient for cold pressing and processing; compared with components with high Li_2_S content, it maintains sufficient mechanical strength and is not too soft to affect structural stability [339]. This balance makes it easier to obtain a dense electrolyte layer and good interface contact in practical applications. Yamamoto and team members used 75Li_2_S-25P_2_S_5_ (LPS) sulfide solid electrolyte, which has a Young's modulus of about 24 GPa [340]. Its relatively low mechanical strength enables it to provide a certain buffering effect through elastic deformation during volume change. In the traditional solid electrolyte layer configuration with a thickness of 500–1000 μm, this elastic deformation can effectively relieve the mechanical stress generated by the volume change of the electrode, thereby preventing the stress from being transmitted to the opposite electrode. However, when the thickness of the solid electrolyte layer is reduced to about 75 μm, this buffering effect is significantly weakened. Compressive stress has a complex and critical effect on the mechanical behavior and ion conduction performance of solid electrolytes. The study found that in the pressure range of 15–75 MPa, higher compressive stress can maintain close contact between solid electrolyte particles, thereby maintaining the continuity of the ion conduction network. The resistance of the solid electrolyte drops sharply with increasing pressure until it stabilizes at about 70 MPa, indicating that 70 MPa is the threshold pressure required to maintain an effective ion conduction path. At a pressure of 75 MPa, the solid electrolyte exhibited sufficient mechanical strength to withstand the stress changes during cycling, with a relatively small normalized resistance increase of only 1.3–1.5 times, indicating that the solid electrolyte structure remained relatively stable under moderate pressure. However, the mechanical limitations of the thin solid electrolyte layer became apparent in the face of the large volume change of the silicon anode. The silicon composite electrode expanded by approximately 55% during lithiation, corresponding to a thickness increase of approximately 12 μm. This significant volume change transmitted stress to the cathode through the thin solid electrolyte layer, resulting in a sharp increase of 4.8–5.2 times in the cathode NCM/solid electrolyte interface resistance. In contrast, the graphite anode, due to its smaller volume change, experienced a corresponding interface resistance increase of only 3.3–3.7 times. This phenomenon revealed that the thin solid electrolyte layer could not provide sufficient mechanical buffering to isolate the volume changes of the anode from the positive electrode. The solid electrolyte also exhibited important plastic deformation behavior and self-healing properties under high pressure. Under a pressure of 75 MPa, the solid electrolyte was able to repair fine cracks formed in the silicon electrode through plastic deformation, promoting close contact between the Li_x_Si and the solid electrolyte. This plastic deformation ability is crucial for maintaining electrochemical activity at the electrode-electrolyte interface (Fig. 25c). In contrast, a lower pressure of 50 MPa was insufficient to induce sufficient plastic deformation to repair the cracks, resulting in the accumulation of more fine cracks and deterioration of the interfacial contact. Furthermore, under high pressure, the synergistic plastic deformation of the solid electrolyte and Li_x_Si resulted in the silicon particles exhibiting a unique amoeba-like morphology. This morphological change reflected the mechanical interaction between the materials during repeated lithiation and delithiation (Fig. 25d).

When the Li_2_S content was further increased (such as 80Li_2_S-20P_2_S_5_ or higher), the lithium ion concentration in the material increased, the PS_4_^3−^ tetrahedral structural units increased, the network connectivity decreased, resulting in a decrease in mechanical strength and the material became softer, but the plasticity and processability were significantly improved [341]. The mechanical properties of 80Li_2_S-20P_2_S_5_ showed more outstanding flexibility and plasticity. The high Li_2_S content significantly reduced the brittleness of the material, making it easier to achieve good contact and densification between particles during the pressing process. The compressibility of the material was significantly improved, and a high-density electrolyte sheet could be obtained at a relatively low pressure, which was of great significance for reducing manufacturing costs and improving interface contact [342]. However, excessive flexibility also brings the problem of relatively low mechanical strength, and the material needs to be handled and used with more care. In the study of all-solid-state lithium batteries with porous amorphous silicon thin film anodes, 80Li_2_S-20P_2_S_5_ glass exhibited key mechanical performance characteristics, which directly affected the battery's cycle stability and electrochemical performance. The solid electrolyte has a relatively low elastic modulus of about 20 GPa. The mechanical property enables it to undergo reversible deformation under high pressure, providing an important mechanical buffer for the huge volume change of the silicon anode during the lithiation/delithiation process [343]. This low elastic modulus property of the solid electrolyte is in sharp contrast to traditional rigid ceramic materials, enabling it to maintain the mechanical integrity of the anode/electrolyte interface through compression deformation under uniaxial pressure. This deformation ability is crucial for adapting to the quasi-one-dimensional volume expansion of the silicon film during the lithiation process. Sakabe et al. [344] found that the mechanical response behavior of the solid electrolyte showed significant differences in silicon films of different thicknesses. For a thin silicon film with a surface capacity of 0.6 mAh cm^−2^, the solid electrolyte can effectively withstand and disperse the mechanical stress generated by the silicon volume change, so that the porous and non-porous silicon films show similar characteristics in the electrochemical impedance spectroscopy, indicating that the solid electrolyte has sufficient mechanical toughness to maintain interface stability within this thickness range. However, when the areal capacity increased to 2 mAh cm^−2^, the increased thickness of the silicon film lead to a greater absolute volume change, severely challenging the mechanical load-bearing capacity of the solid electrolyte. Non-porous silicon films exhibited significantly increased diffusion and charge transfer resistances due to the loss of structural integrity, while porous silicon films effectively mitigated mechanical stress on the solid electrolyte through their inherent stress relaxation mechanism. The mechanical behavior of the solid electrolyte during cycling was directly influenced by the evolution of the silicon film's microstructure. Non-porous silicon films were prone to through-thickness cracks during repeated lithiation/delithiation. The formation and propagation of these cracks imposed localized mechanical stress concentrations on the solid electrolyte, hindering ion transport pathways at the electrolyte-electrode interface. In contrast, porous silicon films partially accommodated this volume expansion through their internal pore structure. Although the pore volume could only accommodate approximately 20% of the volume expansion, this structural design significantly reduced the mechanical stress transmitted to the solid electrolyte, allowing the electrolyte to maintain relatively stable mechanical integrity. The mechanical properties of the solid electrolyte not only excelled during slow cycling but also maintained structural stability under the transient mechanical shock of rapid charge and discharge. This mechanical stability was of great significance for achieving high-power density all-solid-state batteries, as it could fully resist the more severe mechanical impact of the rapid volume change of silicon on the solid electrolyte during high-rate charge and discharge.

##### Others

Apart from the Li_2_S-P_2_S_5_ series mentioned above, the LPSI (LiI-Li_3_PS_4_) system solid electrolyte is also a composite electrolyte material formed by introducing the halide LiI into the Li_3_PS_4_ matrix, representing an important research direction for the modification of sulfide electrolytes. This system fully utilizes the synergistic effect of the two components and exhibits unique performance advantages. From the perspective of ionic conductivity, the introduction of LiI significantly improves the ionic conductivity of the system. As an ionic conductor, the I^−^ ions of LiI have a large ionic radius and good polarization ability, which can effectively regulate the conduction environment of lithium ions. Studies have shown that the addition of an appropriate amount of LiI can increase the room temperature ionic conductivity of the composite electrolyte to the order of 10^−1^ S cm^−1^ or even higher, which is mainly attributed to the fast ion conduction channel formed at the interface between LiI and Li_3_PS_4_ and the optimization of the overall structure [345]. In terms of mechanical properties, the LiI-Li_3_PS_4_ system exhibits improved plasticity and processing properties. The softening effect of LiI reduces the brittleness of the material, making it easier to obtain a dense electrolyte sheet by cold pressing. At the same time, the system maintains the relatively good interfacial adaptability of sulfide electrolytes [346]. Electrochemical stability is another advantage of this system. The presence of LiI helps to improve the interfacial stability between the electrolyte and the electrode material, especially the compatibility with high-potential positive electrode materials. However, the system still faces the common challenges of sulfide electrolytes, including sensitivity to moisture and the complexity of the preparation process. The mechanical properties and stress-strain characteristics studied by Maresca and team are mainly reflected in the exploration of LPSI, especially its application in all-solid-state lithium-ion batteries [347]. During the battery assembly process, Maresca et al. prepared electrolyte particles by applying pressure through a hydraulic press. The specific operation was to press 200 mg of LPSI under 1 ton pressure for 10 s, then place the composite electrode material on the LPSI particles, apply 5 or 6.5 tons of pressure for 20 s, and finally place a lithium sheet on the other side and press under 1 ton pressure for 10 s. They ensured that the final thickness of the entire particle is controlled within the range of 750–800 μm. The research team emphasized the important effect of mechanical pressure on the performance of solid-state batteries. After comparing the battery performance under different pressure conditions, it was found that when cycling at a 0.1 C rate, the irreversible capacity was significantly reduced by increasing the battery packaging pressure, indicating that appropriate mechanical pressure can improve solid-solid interface contact and reduce interface resistance. Although the surface roughness of the SnMAG composite electrode increased after 30 cycles, this may be due to the small but repeated volume changes of Sn during the charge and discharge cycle, unlike the more obvious volume changes in liquid batteries, no significant capacity decay was observed in the all-solid configuration. It means that although volume changes do occur, they are fully controlled and almost no pulverization occurs. In addition, due to the pressure applied during the battery preparation process, the contact between the solid electrolyte and the electrode is maintained during the cycle. The effect of mechanical stress on the performance of electrode materials is particularly prominent in the application of Sn-based anode materials [348]. To overcome these problems, researchers such as Maresca adopted the strategy of dispersing Sn in a graphite/LPSI buffer matrix [347]. The reduction of Sn size plays an important role in suppressing the pulverization phenomenon, which is achieved by reducing the strain of lithiation/delithiation, while carbon as a framework and buffer space for Sn further alleviates the mechanical stress. From the perspective of mechanical stability, solid electrolytes show significant advantages over liquid electrolytes. In liquid battery configurations, the volume change of Sn is one of the most critical disadvantages. By using solid electrolytes and graphite, the volume expansion of Sn is physically controlled, which has a good effect on the uniformity of the composite anode. The mechanical stability of the LPSI electrolyte was also reflected in its compatibility with the lithium metal anode. Lithium-lithium symmetric cell plating/stripping cycle testing showed that the battery exhibited stable lithium plating/stripping behavior over 80 h, with a constant overvoltage value, indicating a stable interface between LPSI and lithium and the absence of lithium dendrite formation. Overall, the amorphous properties of the solid electrolyte, and the composite electrode structure can effectively control the mechanical stress and volume changes of the material during cycling, thereby achieving stable and efficient all-solid-state lithium-ion battery performance.

**Fig. 26:**
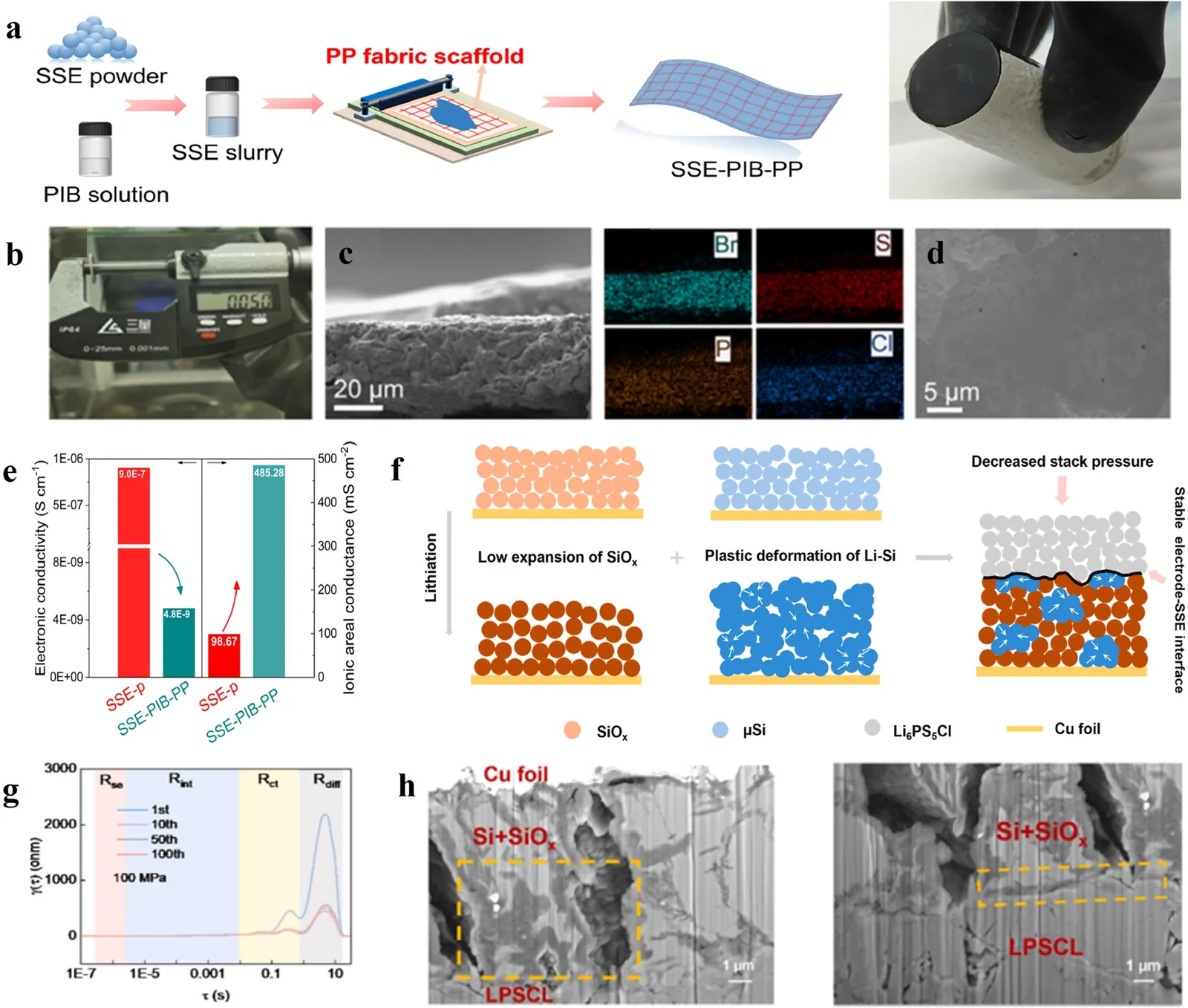
**a** Diagram of the fabrication process and optical photograph. **b** Thickness measurement; **c** Cross-sectional SEM micrograph and EDS elemental mappings; **d** Top-view SEM image of the SSE-PIB-PP membrane. **e** Evaluation of electronic conductivities and ionic areal conductance for SSE-p and SSE-PIB-PP [349]. © 2025 Elsevier B.V. **f** Diagram illustrating the interface between the SiO_x_ and μSi hybrid electrode and the SSE. **g** DRT analysis of the SiO_x_ and μSi hybrid anode across various cycles under pressures of 100 MPa. **h** Magnified images of SiO_x_ and μSi hybrid particles and the interface between the SiO_x_ and μSi hybrid anode and LPSCl [354]. © 2025 Elsevier B.V

However, the study found that both the traditional dry-processed SSE-PTFE membrane and the wet-processed SSE-PIB membrane had serious mechanical performance defects when matched with electrolyte-free silicon-carbon anodes. The SSE-PTFE membrane was due to the defluorination reaction of the PTFE binder in the electrochemical environment to generate a conductive amorphous carbon network, which lead to direct short circuit failure of the battery. Although the SSE-PIB membrane had good chemical stability, it was mechanically weak and could not withstand the huge volume change stress generated by the alloy-base anode during the lithium insertion and removal process, resulting in microscopic voids and delamination at the two-dimensional Si–C|SSE-PIB interface, which ultimately caused rapid capacity decay. To solve these mechanical performance problems, Ji and research team developed an innovative SSE-PIB-PP composite membrane, which achieved excellent mechanical performance improvement through the rigid-flexible coupling design of the PIB elastic binder and the PP non-woven fabric support (Fig. 26a) [349]. Specifically, the SSE-PIB-PP membrane exhibited a tensile strength of 34.7 MPa, an elastic modulus of 411 MPa, and an elastic recovery of approximately 30%. These excellent mechanical properties enable it to effectively withstand and disperse the internal stresses generated during the cycling of the silicon anode. In situ pressure monitoring experiments revealed that, compared to an SSE-p membrane approximately 800 microns thick, a 50-micron-thick SSE-PIB-PP membrane redistributes approximately 35.5% of the internal stress generated by the silicon volume change, significantly reducing interfacial mechanical damage caused by stress concentration (Fig. 26b-d). Mechanical property testing results demonstrated that the design concept of the SSE-PIB-PP membrane leveraged the rubbery elastic properties of the PIB binder to provide stress buffering capacity, while simultaneously relying on the PP fiber scaffold for mechanical reinforcement, forming a synergistic stress-dissipating mechanism. During cycling, when the silicon anode undergoes volume expansion, the maximum stress change within the membrane was only approximately 0.6 MPa, significantly lower than the 1.1 MPa stress change associated with thicker SSE-p membranes. This stress-dissipating capability effectively prevented mechanical fracture and chemical decomposition at the interface. SEM observations confirmed that batteries using SSE-PIB-PP membranes could maintain close electrode-electrolyte interface contact after deep charge and discharge cycles, without observable cracks or voids, ensuring the continuity of the charge carrier transport channel (Fig. 26e). This optimization of mechanical properties not only solved the problem of interface stability, but also significantly improved the electrochemical performance of the battery. It emphasizing that in high-energy-density all-solid-state batteries, the electrolyte membrane must not only have good ionic conductivity and chemical stability, but also sufficient mechanical strength and elasticity to adapt to the volume changes of the electrode material. In particular, for alloy-type anode materials, the optimization of mechanical properties is a key factor in achieving long-term stable cycling.

Adding polymers and oxides to sulfide-based solid electrolytes to form a multi-component composite system is currently an important strategy to improve the comprehensive performance of sulfide electrolytes. This composite method can effectively combine the advantages of each component and make up for the shortcomings of a single material. The addition of polymers mainly plays the following roles: first, it improves mechanical flexibility. Polymers have good elasticity and bendability, which can significantly reduce the brittleness of sulfides and improve the impact resistance and cycle stability of composite electrolytes [350]. Secondly, it enhances interfacial stability. Polymers can form a continuous bonding phase between sulfide particles, improve the bonding strength between particles, and act as a buffer layer at the interface between electrolyte and electrode, alleviating stress concentration during charging and discharging. In addition, some polymers also have certain ionic conductivity and can provide additional conduction paths for lithium ions [351]. The introduction of oxides mainly plays the role of structural stability and performance regulation. Oxides such as Al_2_O_3_ and SiO_2_ have excellent chemical stability and mechanical strength, and can serve as rigid supporting phases to improve the overall strength and dimensional stability of the composite system. Oxides can also regulate the microstructure of the composite electrolyte and affect the distribution of ion conduction paths through interfacial interactions. More importantly, some functional oxides such as LLZO and LAGP are excellent ion conductors in themselves. Their addition can not only provide structural support but also contribute additional ionic conductivity [352]. The three-component composite system achieves performance optimization through synergistic effects: sulfide provides high ionic conductivity, polymer imparts flexibility and interfacial compatibility, and oxide provides structural stability and chemical inertness. This design concept provides an effective way to develop practical solid electrolytes with high conductivity, good mechanical properties, and long-term stability. Dunlap et al. [353] mainly focused on Li_6_PS_5_Cl as an ion transport medium in all-solid-state lithium-ion batteries. The interfacial compatibility and mechanical interaction with coal tar pitch-derived silicon-carbon composite anodes directly determine the stability of the battery. LPSCl was selected as a solid electrolyte due to its high ionic conductivity and mechanical deformability. Its mechanical properties in the composite anode are a high Young's modulus and excellent ductility. A dense interface is formed by cold pressing under 100 MPa to adapt to the volume expansion of silicon particles during lithiation and avoid electrode pulverization and ion contact loss. Dunlap emphasized that the mechanical strength of LPSCl is further enhanced by the addition of polymer binders such as PVDF, achieving a tensile strength of approximately 50 MPa, ensuring structural integrity during cycling. The key to the mechanical strength of the LPSCl glass-ceramic structure lies in the flexibility provided by the amorphous phase and the rigidity enhanced by the crystalline phase. Under stresses of 10–20 MPa, the strain recovery rate exceeded 95%, helping to dissipate local stresses induced by the expansion of the silicon anode and prevent crack propagation. More notably, LPSCl's high viscoelasticity maintained continuous contact at the interface. Even when the silicon anode shrinked due to delithiation, it maintains the ion transport path through elastic rebound, minimizing interfacial impedance growth. Another key to mechanical strength was the addition of conductive carbon derived from coal tar or oil pitch. These carbon particles not only enhanced the modulus of the composite electrolyte but also stabilized the polymer chains through carbon–sulfur bonds, preventing creep deformation at temperatures above 60 °C. The team found that the mechanical properties of the LPSCl composite membrane were stable over a wide temperature range from −20 to 80 °C, with fluctuations in the Young's modulus below 10%, ensuring battery reliability under certain extreme operating conditions. The stress-strain characteristics of LPSCl were further reflected in its interfacial compatibility with the Si−C anode. The flexibility of the sulfide chain allowed LPSCl to penetrate into the electrode micropores during the hot-pressing assembly process, forming a gradient interface structure and alleviating the radial stress during the lithiation of the silicon anode. It was observed that LPSCl underwent 5%–10% elastic strain when the silicon particles expanded, without obvious plastic deformation. Compared with the brittle fracture of rigid oxide materials, the viscoelastic modulus of LPSCl ensured the integrity of the interface, and there was no obvious crack after 100 cycles. The glass transition temperature of the LPSCl film was only −20 °C, ensuring flexibility at low temperatures.

Yang and team paid special attention to the effect of different types of solid electrolytes on battery performance [354]. The team used two main solid electrolyte systems: organic/inorganic composite solid electrolyte PVDF/LLZO and sulfide solid electrolyte Li_6_PS_5_Cl. For the PVDF/LLZO composite solid electrolyte, the study found that volume expansion was the main factor determining the cycle stability and electrode kinetics. The PVDF/LLZO SSE system with good interfacial compatibility exhibited relatively stable electrochemical performance. By employing a low-expansion chemical vapor deposited carbon-coated silicon oxide (SiO_x_@C) anode, researchers achieved stable cycling performance and high areal capacity, reaching up to 23 mAh cm^−1^, under zero external stacking pressure (Fig. 26f). The carbon coating layer distributes the stress generated by the SiO_x_ particles to the flexible and elastic carbon layer via the C−Si contact, thereby buffering the volume expansion of the electrode during charge and discharge. Low volume expansion alone is not sufficient to achieve good cycling stability at low stacking pressure; the plastic deformation capacity of the silicon particles is also crucial. In sulfide electrolytes, despite exhibiting greater volume expansion, the μSi anode exhibited better interfacial stability and lower impedance than the low-expansion SiO_x_ anode due to its excellent plastic deformation capacity. Because the hardness of the lithium-silicon alloy decreased significantly after lithiation, allowing the lithiated lithium-silicon alloy to have sufficient plastic deformation capacity to maintain good contact with the solid electrolyte. According to Fig. 26g, distribution of relaxation times (DRT) measurements are employed to evaluate the electrochemical dynamics of SiO_x_ and μSi composite anodes under 100/30 MPa mechanical stress conditions. FIB-SEM observations revealed that the μSi particles partially fuse after cycling, forming a more tightly fitting two-dimensional interface, while more micrometer-scale voids exist between the SiO_x_ particles and the solid electrolyte (Fig. 26h). Based on this understanding, SiO_x_ and μSi were successfully combined in a ratio of 7:3, successfully combining the low expansion properties of SiO_x_ and the good plastic deformation ability of μSi, achieving excellent cycle stability in sulfide solid-state batteries. The capacity retention rate reached 81% after 100 cycles at 100 MPa, and the retention rate was 50% after 30 cycles at 30 MPa. Even at an extremely low stacking pressure of 10 MPa, it could still provide a high discharge specific capacity of 947 mAh g^−1^. This study refreshes the understanding of the design principles of low-stack-pressure solid-state battery anodes and provides important design guidance for practical solid-state batteries using organic/inorganic composite solid electrolytes and sulfide solid electrolytes.

To optimize the mechanical strength of alloy-based anode solid electrolytes, researchers have developed a variety of effective modification strategies to address the mechanical challenges posed by the significant volume change of electrode materials. Molecular structure manipulation is the most fundamental optimization approach, balancing strength, and flexibility by adjusting crystallinity, molecular weight, and degree of crosslinking of polymers. For example, copolymerization of PVDF with hexafluoropropylene reduces crystallinity to improve ion conductivity while maintaining good mechanical strength. Composite filler modification strategies significantly enhance the mechanical properties of materials by introducing inorganic ceramic fillers such as LLZTO and Al_2_O_3_. These fillers not only provide pathways for lithium ion transport but also synergistically improve ionic conductivity and mechanical strength by reducing polymer crystallinity and agglomeration. The innovative application of in situ polymerization mechanisms completely addresses the problem of traditional plasticizers weakening mechanical integrity. PC and PVDF undergo in situ polymerization through electrochemical reactions, maintaining high ionic conductivity while eliminating the safety risks of liquid plasticizers. Advanced fabrication techniques such as electrospinning enable the construction of three-dimensional interconnected nanofiber networks, significantly enhancing tensile strength and stress release capabilities through ultra-high surface area and interfiber elastic deformation. A multi-component composite design leverages the synergistic effects of sulfide, polymer, and oxide to achieve a combination of high ionic conductivity, good flexibility, and structural stability. Specifically, a rigid-flexible coupled SSE-PIB-PP composite membrane design, combining a PIB elastic binder with a PP non-woven scaffold, achieves excellent stress dissipation. Stacking pressure optimization and electrochemical-mechanical coupling control improve solid-solid interfacial contact by applying appropriate external pressure, while leveraging the stress-induced overpotential effect to achieve uniform reaction current distribution and interface flattening. Interface engineering strategies enhance mechanical flexibility and chemical stability by constructing gradient interface structures and LiF-rich interfacial layers, effectively inhibiting lithium dendrite growth and maintaining mechanical integrity at the interface. Future mechanical strength optimization will inevitably evolve toward more intelligent and multifunctional approaches. Self-healing capabilities introduced through dynamic chemical bonds, such as hydrogen bonds, coordination bonds, or reversible covalent bonds, can enable electrolytes to self-heal cracks and automatically repair mechanical damage during cycling. Prestressed design introduces appropriate pre-compressive stress during the fabrication process, enabling the material to better withstand cyclic stresses during operation. More importantly, multi-scale structural manipulation is a key approach to improving mechanical stability. The implementation of these forward-looking strategies will advance the mechanical performance optimization of solid electrolytes to new heights, laying a solid foundation for the industrial application of next-generation high-energy-density, long-life all-solid-state batteries.

## Advanced Characterizations for Alloy-based SSBs

Due to the unique solid-state-electrolytes between cathode and anode, and the volume effect of alloy-based anode during cycling, alloy-based all-solid-state batteries (alloy-based SSBs) possess a nature of interfacial complexity and intricate lithium-ion migration mechanism. Thereby, conventional characterization methods effective on liquid-electrolyte systems, are incapable of detecting the electrochemical process occurring in the alloy-based SSBs accurately. For instance, the anisotropy of solid-state samples incurs spectral line broadening when it comes to traditional NMR, which is not competitive to characterize the migration behavior of lithium-ion in detail, and thus negatively influences the analysis of the failure mechanism. To better find out the thorny problems and understand the distinct mechanisms existing in the alloy-based SSBs, it is of great necessity to adapt advanced characterizations correspondingly. In this section, the introduction will be mainly divided into three parts: morphology and structure characterization, surface and internal component analysis, and emerging digital simulations.

### Morphology and Structure Characterization

#### In situ* Techniques*

**Fig. 27:**
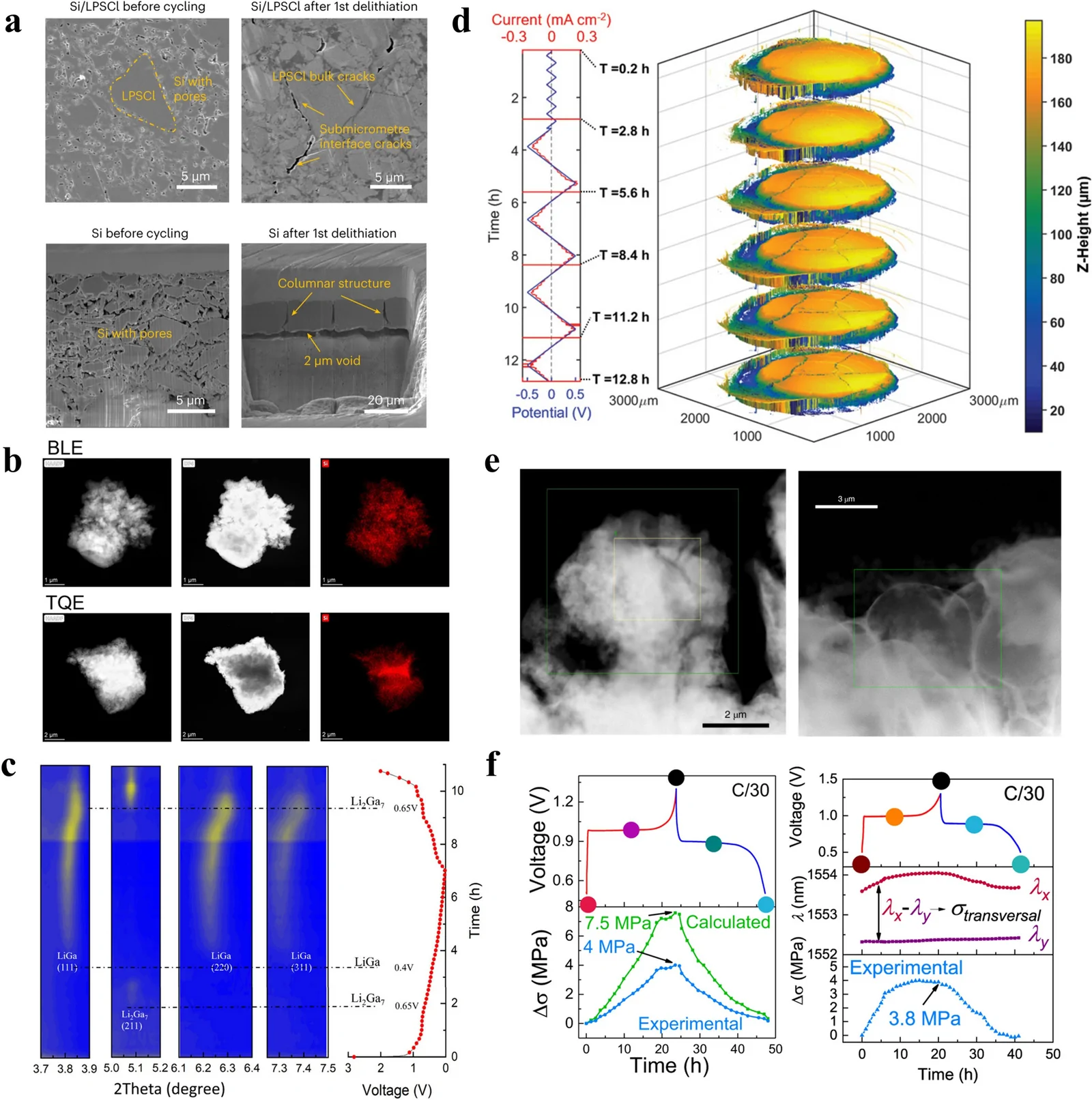
**a** In situ SEM images of the heterointerfaces of both composite Si/Li_6_PS_5_Cl and SE-free Si anode in different stages. [355] © 2023 Springer Nature Limited. **b** In situ TEM images of the silicon particles in batteries with BLE and TQE after cycling test. [356] ©2023Published by Elsevier B.V. **c** In situ XRD patterns.[319]. © 2024 American Chemical Society. **d** 3D renderings of the LGPS pellet in the SSB during cycling. [357] © 2020 Wiley–VCH GmbH. **e** Cryo-TEM images of the interfaces between Li^0^ anodes and SPE and FEC-SPE with EDS maps. [358] © The Author(s), under exclusive licence to Springer Nature Limited 2022, corrected publication 2022. **f** Internal and external stress changes of the InLi_x_ anode and the InLi_x_|Li_3_PS_4_ interface separately captured by external force sensor and FBG. [359] © The Author(s) 2022

The dynamic interfaces between the electrodes and SSE determine that the electrochemical process and structural changes might be subtle and instantaneous, only happen during the reaction and cannot be observed after certain stages, such as the temporary existence of side reactions, which conventional characterizations can barely capture and therefore analyze their effect on the performance of battery. In order to cope with situations mentioned above, in situ techniques, including in situ SEM, TEM, XRD, and X-ray tomography, have come to the fore and been widely applied in the research of SSB [355]. Electrochemical in situ techniques, focusing on real-time tracking of electrochemical behaviors and interface changes under battery working conditions (e.g., charging/discharging), are characterized by dynamic monitoring and direct correlation with reaction processes. Compared with standard SEM, in situ SEM is competent to characterize the microstructure evolution across the heterointerfaces while the battery is charging/discharging, since it integrates with a controlled-atmosphere sample chamber and other equipment, which provides us with substantial evidence to understand the failure mechanism. For example, Huo et al. [305] used in situ SEM to study the interaction between the heterointerfaces of both composite Si/LPSCl and SE-free Si anode in SSBs. As is shown in Fig. 27a, the LPSCl|Si/LPSCl interface still kept close contact after 100 cycles even though there are submicrometer-scale cracks formed by the expansion of silicon particles and these cracks propagated and widened after further cycling. While a 2-μm-wide void, which indicated irreversible plastic deformation, came into being at the LPSCl|Si interface (interface between SE-free Si anode and the SSE) after the first delithiation, and the void was widened to 10 μm after the 100th delithiation, causing the failure contact. By characterizing the interfaces between different electrodes and SSE through the in situ SEM, it is possible to reveal the distinct failure mechanisms behind them.

Same principle applies to in situ TEM, rather than capturing the three-dimensional surficial morphology as SEM does, TEM techniques can gain the 2D image of the internal structure and analyze the atomic changes during cycling, since it could reach a maximum magnification of 1,500,000 × via the Aberration-Correction (that is, AC-TEM) or even 230,000,000 × by leveraging ultra-stable electron optics, monochromatized electron sources, and multi-dimensional aberration correction to achieve sub-angstrom resolution (which is a specific TEM called AC-STEM). A case in point is work done by Zhao et al. [356], they used Talos F200X in situ STEM to separately characterize the silicon particles in batteries with based liquid electrolyte (BLE) and TXE-based quasi-solid electrolyte (TQE) after cycling test. As a result, shown in Fig. 27b, there were cracks and voids that appeared on the Si particle with BLE because of the inherent volume effect of silicon during the lithiation/delithiation process. Meanwhile, the Si particle cycled with TQE maintained integrity attributed to a three-dimensional network structure, which tightly wrapped the active material particles and thus mechanically limited their intense expansion caused by the volume effect. The difference above implies the fact that the polymer skeleton synthesized in situ effectively controls the volume evolution, which supports the structure of electrodes from falling apart. The unparalleled resolution of TEM techniques renders observing the electrochemical process from a sub-angstrom perspective possible.

Besides applying the above powerful characterization methods which are developed on the basis of electron, scientific tools derived from the X-ray play essential roles as well in terms of observing the intricate process inside the SSBs. As one of the most important tools, in situ XRD has been prevalently used in the range of substance synthesis to monitor the evolution of various processes. When X-rays irradiate the sample, the regularly arranged atoms in the sample will diffract the X-rays, forming a specific diffraction pattern. By analyzing information such as the peak positions and intensities of the diffraction pattern, the crystal structure, phase composition, and lattice parameters can be determined during battery operation. The in situ XRD technique was utilized to track the structural evolution of gallium-indium (Ga-In) liquid metal alloy during the lithiumization/delithiumization process in real time, revealing its dynamic reaction mechanism as a anode for solid-state lithium-ion batteries (Fig. 27c) [319]. The authors observed that in the electrochemical cycling, only gallium (Ga) participated in the lithium storage reaction of the Ga-In alloy, while the role of indium (In) was to maintain the low melting point characteristic of the alloy and ensure that the material could recover to a liquid state after de-lithiation. Specifically, during the discharge process, the XRD spectra showed the sequential formation of Li_2_Ga_7_ (5.07° diffraction peak) and LiGa (3.87°, 6.3°, 7.4° diffraction peaks), and the solid solution mechanism of lithium intercalation was confirmed through the lattice expansion phenomenon. Additionally, no Li_2_Ga phase was detected under high current conditions, indicating that the kinetic limitation affected the formation of the final lithiumized product. These results not only verified the self-repairing characteristic of Ga-In alloy through the reversible liquid-solid transformation to achieve stable interface contact, but also excluded the direct electrochemical activity of indium. Additionally, this study provided a rational strategy to characterize the alloy-based SSBs that could help us better figure out the complicated mechanism and thus come out with a more stable and excellent battery design.

Unlike in situ SEM, where the 3D perception is simulated through the differences in grayscale distribution signals on a 2D plane, the X-ray tomography, which is usually called as CT in medicine field, is able to scan and present the 3D model of samples directly through a rotating X-ray source and detector array capture differential X-ray attenuation by materials from multiple angles, which computational algorithms reconstruct into cross-sectional images of internal structures. Madsen et al. [357] applied Operando X-ray tomography to get the 3D renderings of an LGPS pellet in the SSB during cycling as shown in Fig. 27d, and demonstrated that topologically heterogeneous regions (highlighted in yellow) form at the LGPS/Li interface. These regions are products of electrochemical reduction and decomposition, mainly composed of Li_2_S, a small amount of Li_3_P and Li_3.75_Ge, and will expand from the interface to the bulk phase with cycling. This causes the reductive decomposition reaction of LGPS to propagate from the interface into the bulk LGPS and hinders a stable interfacial passivation layer from forming. As a result, the bulk LGPS undergoes continuous decomposition, which further exacerbates the damage to the bulk structure. And it also reveals that in all-solid-state batteries, low-density domains form at the LGPS-Li interface due to reductive decomposition, generating stress that causes electrolyte cracking. In batteries with interlayer modification, the liquid electrolyte inhibits such decomposition and reduces cracks. It indicates that solid electrolyte interfacial decomposition is the main cause of mechanical failure, confirming that interface modification is crucial for the stability of solid-state batteries.

#### Cryogenic Electron Microscopy

As one part inside every single battery, SEI originates from the contact of electrolyte and anode, and plays an indispensable role since the various states of its formation could lead to significant differences in the occurrence of side reactions, the ICE and the cycling stability of the battery. Especially for alloy-based SSBs here, whose electrolytes and anodes are both solid-state, the SEI formed across the heterointerfaces always faces issues like failure contact and frequent side reactions caused by the poor solid-solid contact or the volume effect of alloy-based anodes, which then negatively impact the continuous transport of ions between interfaces. Therefore, studying the construction and evolution of SEI in such an all-solid-state system enables us to better comprehend the mechanism and further propose adequate strategies to improve. Even though the electron microscopies mentioned previously endow us with the capability to spot the ongoing process from a high-resolution perspective, the electron beam sensitivity of battery materials precludes such analysis. To tackle this problem, cryo-TEM (cryogenic transition electron microscopy) is put forward and it has become a hit in recent reports of studying interfaces of SSBs because it could observe the samples steadily without damaging them for basically two reasons. The first one is that some samples could be sensitive to radiation emitted by regular TEM, which might induce crystal structure damage and even chemical decomposition. Meanwhile, the cryo-TEM operates under the temperature of −196 °C, which significantly suppresses the thermal motion and chemical activity of molecules inside the samples, so the samples become more durable to electron radiation. The other is protecting the samples from reacting with air, since most materials inside SSBs like lithium are extremely active with air and thus destroy the original morphology. Unfortunately, there are few reports about applying this technique to alloy-based SSBs, but here is another example that could demonstrate its supremacy. Lin et al. [358] separately characterized the interfaces between Li^0^ anodes and different solid-polymer-electrolytes (SPE) and found out that cryo-STEM characterization of the baseline SN-incorporated PolyEA SPE (SN-SPE) revealed specific morphological features of lithium filaments. As shown in Fig. 27e, the lithium filaments plated in SN-SPE under a current density of 0.1 mA cm^−2^ for 60 min exhibited distinct features in HAADF-STEM images: the filaments displayed bright contrast in most regions with only small dark domains or stripes, indicating the occurrence of side reactions and the formation of cracks or voids inside. Moreover, no conformal SEI films were observed on these filaments. In contrast, the introduction of 5 wt% FEC (FEC-SPE) brought about remarkable changes: cryo-STEM imaging showed densely packed Li^0^ domes with a thin, conformal SEI coating, which is 20–30 nm thick, featuring a mosaic structure. EDS maps confirmed that elements like O, C, N, S, and F were enriched only on the surface of these domes (constituting the SEI) rather than distributing throughout the Li^0^ deposits, unlike the case in SN-SPE, collectively demonstrating the formation of a stable passivation layer. What’s more, it paves the way for broader applications of cryo-TEM in studying complex heterointerfaces, including those in alloy-based SSBs, where clarifying SEI construction and evolution is crucial for addressing contact failure and side reaction issues.

#### Fiber Bragg Gating

Chemo-mechanical stress induced by the volumetric and morphological change in electrodes emerging when the battery is working, will always affect its performance significantly, and such an issue is even more pronounced when it comes to the alloy-based SSBs, as it is accompanied by problems like unstable contact between the SSE and the electrodes, as well as the volume effect of the alloy. However, the existing detection methods to monitor stress are basically placed outside the battery system, which merely provide data with limited accuracy and incomplete coverage. Conversely, the fiber bragg gating (FBG) optical sensor could give more precise data without influencing the battery performance, since the fibers made of specific materials could be embedded inside the electrodes or the interface between them and the SSEs, continuously monitoring the optical signal and then converting it into data reflecting the relation between stress and voltage. The study conducted by Marchini et al. [359] is a good case to demonstrate, where they first assembled the all-solid-state cell with the configuration of InLi_x_|Li_3_PS_4_|Li_4_Ti_5_O_12_ and then applied the FBG sensor to investigate the stress change of the InLi_x_ anode and the InLi_x_|Li_3_PS_4_ interface discretely. As it is illustrated in Fig. 27f, the sensor embedded inside the InLi_x_ measures a stress of 4–7 MPa during charging, and the stress nearly returns to zero after discharging, which indicates mechanical reversibility, while the external force sensor only detects a stress of < 0.5 MPa. Similarly, under an external pressure of 8 MPa, the sensor placed at the InLi_x_|Li_3_PS_4_ interface measures a peak stress of 3.8 MPa during charging, which approaches zero after discharging. In contrast, the value recorded by the external force sensor (< 0.5 MPa) was lower than that obtained by the internal sensor. By comparing the measurement results of external pressure sensors and FBG sensors, it was observed that external pressure sensors are insensitive to the internal pressure changes of batteries during operation, which may lead to misjudgment of the internal mechanism of batteries. In contrast, FBG sensors can accurately capture the pressure fluctuations on the anode surface and at the interface between the electrode and solid-state electrolyte during battery cycling. This technique enables us to better understand the chemo-mechanical coupling effects on the electrode surfaces and at the interfaces between electrodes and SSEs inside SSBs during cycling, thereby opening up new possibilities for further optimizing battery design and improving battery performance.

### Surface and Internal Component Analysis

#### X-ray Spectroscopies

**Fig. 28:**
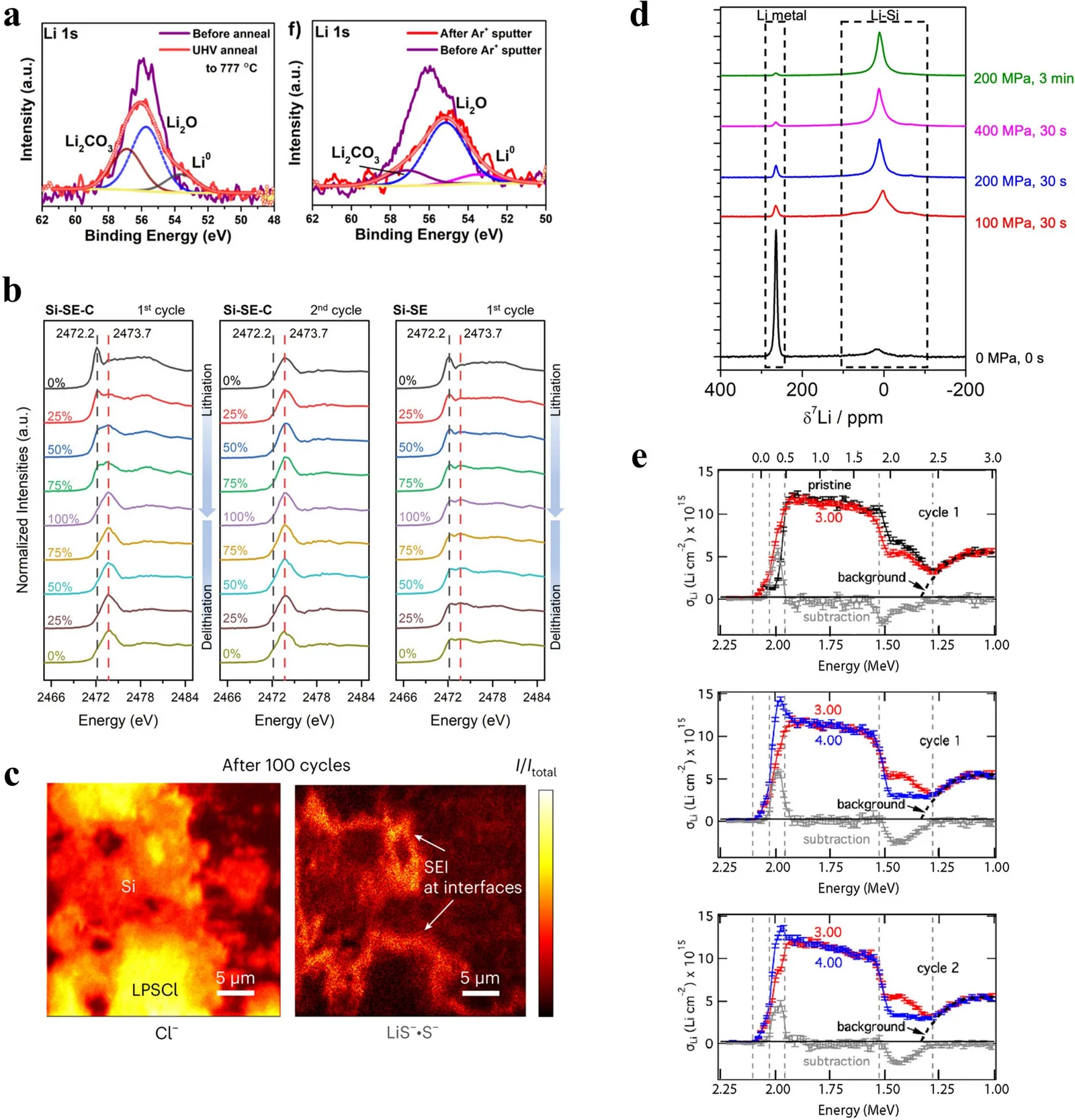
**a** In situ XPS of Li 1 s after UHV annealing to 777 °C, and Li 1*s* after sputter cleaning at 27–227 °C. [360] ©2021AmericanChemicalSociety. **b** Sulfur K-edge XAS spectra of those of the Si-SE-C composite anode at various states of charge and depth of discharge across the first two cycles, and those of the Si-SE composite anode at different lithiation and delithiation states during the first cycle. [285] © 2023 Wiley–VCH GmbH. **c** Images from TOF–SIMS surface analyses of SEI components at Si|LPSCI interfaces after 100 cycles. [355] © 2023 Springer Nature Limited. **d** ^7^Li ssNMR spectra of Li_1_Si under different pressure and time conditions. [361] ©The Author(s) 2024. **e** NDP spatial distribution profiles of Li^+^ in Si-LiPON-LCO solid-state batteries under different SOCs in the first and second cycle. [362] ©2021 American Chemical Society

Alloy-based anode SSBs frequently encounter critical bottlenecks rooted in surface and bulk chemistry: for instance, the spontaneous formation of impurity phases on solid electrolyte surfaces and the dynamic compositional evolution of alloy anodes during charge-discharge cycles. These issues directly impair ionic conductivity at solid-solid interfaces and compromise battery cycle stability, yet conventional characterization techniques often fail to accurately capture surface-specific elemental composition and chemical states, which creates an urgent need for specialized methods to resolve these compositional ambiguities from a chemical perspective. XPS emerges as a pivotal solution to this challenge, it operates on the basis of the photoelectric effect, excites the inner-shell electrons of atoms on the sample surface using X-rays and analyzes the surface elemental composition and chemical states by measuring the energy distribution of photoelectrons. Furthermore, there comes the in situ XPS, when it is combined with ultra-thin inert film window for vacuum-liquid isolation, vacuum-compatible electrochemical three-electrode system and so on, which could further address the demand for real-time compositional monitoring. In one study [360], the contamination made up primarily of Li_2_CO_3_ on the surface of LLZT pellets was cleaned by annealing or sputtering under ultrahigh vacuum condition in an Argon ion atmosphere, and in situ XPS was applied to check if the contamination was removed clearly. The schemes and results are shown in Fig. 28a, where the Li 1 s spectrum demonstrates that the characteristic peaks of Li_2_CO_3_ (~ 56.8 eV) and LiOH (~ 55.8 eV) are weakened after the two treatments, revealing the Li^0^ (~ 53.6 eV) and Li_2_O peaks of the LLZT bulk, which proves that the surface impurities are effectively removed. The in situ XPS used in this case is a key method to confirm the complete removal of impurities, as it can avoid the recontamination caused by sample transfer, which is a problem with ex situ XPS. In summary, XPS is gradually attracting attention in the field of solid-state batteries. Its excellent component detection capability enables us to better optimize the design of materials for solid-state batteries from the perspective of chemical composition, alleviating problems like low ionic conductivity caused by solid-solid contact in batteries, thereby improving the performance such as working capacity and cycle stability.

Like XPS introduced previously, XAS is another profiling technique that is competent to analyze the element composition and chemical state via X-rays, but their operating principles differ. When the energy of incident X-rays approaches the binding energy of an element's inner-shell electrons, they are strongly absorbed by the element's atoms, making the absorption coefficient rise sharply, which is called an absorption edge. Fine oscillations appear on both sides of this edge: the low-energy near-edge region corresponding to the XANES reflects the absorbing atom's electronic state (e.g., oxidation state, coordination symmetry), the high-energy region called Extended X-ray absorption Fine Structure (EXAFS) analyzes the type, distance, and coordination number of surrounding coordinating atoms. Different from XPS which focuses on compositional analysis of the surface (1–10 nm), however, XAS can penetrate the bulk phase of alloy-based anodes and track the decomposition of sulfide SE inside silicon particles. For alloy-based anodes, which are prone to agglomeration and undergo severe volume expansion, this is an unparalleled technique capable of simultaneously monitoring the electrolyte status across both the surface and bulk phases. Operando XANES can real-time monitor the changes in material composition in alloy-based SSBs, so as to clarify factors affecting battery performance such as ion transport mechanisms. For instance, Cao et al. [285] used operando XANES to detect the state of sulfide SE in Si–SE–C and Si–SE composite anodes respectively (Fig. 28b). Their research initially captured the dynamic decomposition process of sulfide SE during the electrochemical cycling of silicon-based anodes in real time. It was found that the sulfide SE undergoes electrochemical decomposition during the first lithiation process of the nanosilicon-based anode, and the decomposition was irreversible, but the decomposition products remained stable in subsequent cycles. In addition, the addition of C intensified this decomposition. The generated products still had ionic conductivity, enabling the Si-SE-C composite anode to maintain a relatively high capacity compared with other materials after the first 50 cycles. Moreover, combined with XnT (X-ray Nanotomography) analysis, it revealed the stability and ionic conductivity of SE decomposition products, explaining why the composite anode with SE and carbon added (Si-SE-C) could still maintain excellent performance when SE was partially decomposed.

#### Time of Flight Secondary Ion Mass Spectrometry

A significant issue hindering the commercialization of SSB is the interface between the SSE and the electrodes. Unlike liquid electrolyte systems, where electrolytes can wet interfaces, penetrate electrodes, form extensive contact areas, and establish stable ion diffusion channels, with SEI preventing solvent co-intercalation to enhance cycle life, SSBs suffer from point contact rather than surface contact, resulting in much smaller effective contact areas. This issue is exacerbated for alloy-based composite anodes, whose significant volume expansion often leads to contact failure after cycling, severely degrading battery lifespan and capacity. Chemically, while SSBs avoid the repeated SEI formation that plagues liquid batteries, SSEs still react with anodes to form SEI-like interfacial layers that profoundly impact performance. For instance, LLZO electrolytes react with Li-metal anodes to form substances that affect or even hinder ion diffusion, and a similar phenomenon occurs with alloy-based anodes.

Therefore, monitoring the composition analysis of the SSE, the electrodes, and the interface formed by their reaction during cycling is crucial for a better understanding of the mechanism of solid-state batteries and the design of battery components. Time-of-Flight Secondary Ion Mass Spectrometry is uniquely suited for this task. Its principle involves bombarding the sample surface with primary ions to eject charged secondary ions and then by measuring differences in their flight times, the mass-to-charge ratio is calculated to analyze surface composition. A key advantage of TOF–SIMS over XAS and XPS is its submicron spatial resolution, which captures the compositional heterogeneity of alloy-based interfaces caused by severe volume expansion. For example, Huo et al. [355] characterized the Si|LPSCl interface using TOF–SIMS before cycling and after 100 cycles. The results, as shown in Fig. 28c, revealed a large number of various anions such as LiP^−^, LiS^−^, LiCl^−^, and SiO^−^ at the interface after cycling. This confirmed that the decomposition reaction of the sulfide SSE occurs at the interface, and its decomposition products are also the main components of the SEI. Furthermore, it was found that the SiO_x_ impurities on the surface of silicon particles are also involved in the formation of the SEI, making the decomposition reaction path more complex. Complementary in situ SEM observations further highlighted structural disparities: pure Si anodes exhibited widening interfacial gaps from 2 μm after 1st delithiation to 10 μm after 100th delithiation, whereas Si-LPSCl composite anodes maintained tight contact despite submicron cracks induced by Si expansion stress. Their work clarified part of the reaction mechanism at the anode interface of alloy-based SSB. Future studies may need to further figure out the reaction process or determine the impact of these reaction products on battery performance, providing the possibility to control or even adjust the reactions to mitigate issues or improve capacity and cycle life.

#### Solid State Nuclear Magnetic Resonance

As mentioned above, SSEs can effectively inhibit the disordered growth of the SEI film and significantly enhance the cycle life of alloy-based anodes. The core mechanism lies in the fact that the reactivity between SSEs and alloy-based anodes is much lower than that of liquid electrolytes, which fundamentally suppresses lithium loss caused by side reactions. A variety of commonly used SSEs can effectively alleviate the volume expansion of alloy-based anodes through different mechanisms and reduce interface failure. On the one hand, for example, sulfide electrolytes restrict the expansion of alloy particles by virtue of their high Young's modulus, making alloy-based anodes undergo plastic deformation instead of brittle fracture after lithiation, thereby maintaining solid-solid interface contact. Composite polymer electrolytes (CPEs), on the other hand, buffer the volume change of alloy-based anodes by utilizing their flexible characteristics. However, sulfide electrolytes have a narrow electrochemical stability window (1.7–2.1 V vs. Li^+^/Li) and are prone to irreversible decomposition during lithiation, resulting in the loss of lithium inventory and hence a low ICE of alloy-based SSBs. For instance, the ICE of LPS and LPSI-based silicon-based SSBs are only 75.9% and 77.6%, respectively [363].

To improve ICE for further extending cycle life and enhancing rate performance, the prelithiation strategy has shown remarkable effectiveness since its proposal and has achieved a certain degree of commercial application. However, in alloy-based SSBs, since both the anode and the SSE are in a solid state, their molecular motion is significantly restricted, and effects such as dipolar interactions between nuclear spins and chemical shift anisotropy are enhanced. As a result, signal broadening occurs when using traditional nuclear magnetic resonance (NMR) to characterize the prelithiation degree of alloy-based anodes. In contrast, solid-state nuclear magnetic resonance (ssNMR), processed through techniques like magic angle spinning and cross-polarization, has become the only characterization method capable of accurately analyzing the chemical state of the atoms to be measured. For example, Ham et al. [361] used ^7^Li ssNMR to probe the chemical state of lithium in prelithiated silicon anodes, successfully distinguishing and quantifying lithium metal (265 ppm signal) and lithium-silicon alloy phases (broad signal around 0 ppm) to study the impact of different pressure and time conditions on the prelithiation degree of silicon. The results showed (Fig. 28d) that in the unpressurized Li_1_Si sample, the proportion of the lithium metal signal was higher, and the lithium-silicon alloy signal at 0 ppm accounted for only 27.8% of the total ^7^Li ssNMR signal intensity, while in the pressurized Li_1_Si sample, the lithium-silicon alloy phase became the main component, with the 0 ppm signal accounting for more than 92%. Moreover, the higher the pressure and the longer the time, the higher the proportion of the alloy phase. This result confirmed that the unpressurized sample continued to undergo lithium-silicon alloying reaction during the ssNMR measurement, while the composition of the pressurized sample remained stable without significant changes. It provides direct chemical state evidence for the effectiveness of the pressure-induced prelithiation strategy and also offers strong references for the regulation of prelithiation parameters and the design of industrialization processes.

In addition to accurately monitoring the chemical state changes of ^7^Li in the Li–Si alloy, the structural stability of the electrolyte can also be indirectly characterized by monitoring the changes in the chemical environment of ^1^H in CPE. For example, Pan et al. [329] applied ^1^H ssNMR to characterize the hydrogen chemical environment of 3D-PPLLP-CPEs under different SOC (open circuit voltage OCV, charged to 4.0 V, discharged to 3.0 V) to verify the in situ electrochemical polymerization reaction between PC and PVDF/PVDF-HFP. The results indicated that under open circuit voltage (OCV), the hydrogen chemical shifts in PVDF and PC were close, making it difficult to distinguish the hydrogen peaks in the ssNMR spectrum. When charged to 4.0 V, the hydrogen peaks changed significantly, indicating that PC underwent a ring-opening reaction and an addition reaction with the elimination products of PVDF/PVDF-HFP, i.e., the occurrence of the in situ electrochemical polymerization reaction. When discharged to 3.0 V, the hydrogen peaks remained consistent with those when charged to 4.0 V, confirming that the polymerized electrolyte structure was stable without additional side reactions. The characterization results of ^1^H ssNMR precisely described the polymerization reaction mechanism of PCE during the electrochemical process and provided a new technical approach for subsequent studies on in situ electrochemical reactions of other SSEs.

#### Neutron Depth Profiling

For LIBs, the diffusion rate of Li^+^ determines how close the actual capacity upper limit is to the theoretical capacity during battery cycling. If the diffusion rate is low, Li^+^ may not reach the electrodes to participate in reactions before the charge-discharge state switches, resulting in a lower actual capacity. This issue is particularly prominent in alloy-based SSBs. Take silicon-based anodes as an adequate example to illustrate, on one hand, compared with the rapid diffusion of lithium ions in liquid electrolytes, in SSBs, since the electrolyte is solid, currently, except for sulfide solid electrolytes, whose Li^+^ diffusion rate can be comparable to that of liquid electrolytes, the diffusion rates of other types of solid electrolytes such as oxide solid electrolytes are generally limited, not to mention the diffusion performance of polymer solid electrolytes is even more ordinary due to their high crystallinity at room temperature [364]. On the other hand, the tight lattice arrangement and small spacing of silicon lead to a significantly lower diffusion efficiency of lithium ions in silicon-based anodes compared to other anode materials [365]. The combined effect of these factors makes the actual cycle capacity performance of Si-based SSBs unsatisfactory, hindering their commercialization process. If the spatial distribution characteristics of Li^+^ inside the battery under different operating states can be clarified, it will help to deeply understand the diffusion mechanism of Li^+^ and the key factors affecting its diffusion rate. However, techniques like XPS and XAS can only detect the surface composition distribution. Even though TOF–SIMS can achieve depth analysis, it is a destructive detection technique. In contrast, Neutron Depth Profiling (NDP), as a non-destructive nuclear reaction technique, can generate α particles and tritium particles through the reaction of cold neutrons with ^6^Li in the sample. By detecting the number and emission energy of these particles, combined with simulation calculations, the concentration of lithium and its depth distribution can be obtained. For example, Strelcov et al. [362] used NDP technology combined with Monte Carlo simulation to study the spatial distribution profiles of Li^+^ in Si-LiPON-LCO solid-state batteries under different SOCs, including the pristine state and when charged to different OCVs. The results showed (Fig. 28e) that when charging from the pristine state to OCV = 3.00 V, there was a net loss of Li^+^ in LiCoO_2_ (LCO) and at the LCO-LiPON interface, while there was a net increase in Li^+^ at the Si-LiPON interface, and the Li^+^ content in the small area close to the Cu current collector was consistent with that in the pristine state. When charged to OCV = 4.00 V, a lithium enrichment peak appeared at the Si-LiPON interface, while the entire Si layer was not fully lithiated, indicating that this interface region is preferentially lithiated and dominates the open-circuit voltage of the battery. The above experimental results confirm that the low lithium-ion diffusivity of silicon leads to uneven lithium distribution, making the Si-LiPON interface a limiting region for battery performance, and the large potential drop in this region is directly related to its high impedance. This provides an experimental basis for improving the performance of solid-state batteries by optimizing the interface lithium enrichment/depletion phenomenon.

### Advanced Computer Simulation

Alloy-based anodes, especially the silicon-based anodes, have emerged as a key candidate material for next-generation SSBs due to their ultra-high theoretical specific capacity. However, they suffer from severe volume expansion during cycling, which easily causes electrode cracking. And elevated impedance from interfacial side reactions, which degrades cycle stability. Moreover, key processes in experimental systems, such as the evolution of material microstructure and multi-physics field coupling effects, are difficult to accurately capture. Coupled with uncontrollable factors like pollution control and operational errors, the research on battery performance optimization and failure mechanisms has fallen into a predicament of "difficulty in reproducing experiments and extracting laws." Against this backdrop, digital simulation technology, by constructing virtualized experimental scenarios, can not only avoid the environmental load and interference factors of physical experiments, but also achieve precise regulation and quantitative analysis of the dynamic evolution process of alloy-based electrodes. With the explosive growth of modern computing power and the iterative innovation of machine learning algorithms, the refined simulation that was once difficult to achieve due to the complex multi-scale characteristics of alloy-based materials has become possible. Digital simulation technology is gradually becoming a core tool to break through the research and development bottlenecks of alloy-based anode solid-state batteries.

**Fig. 29:**
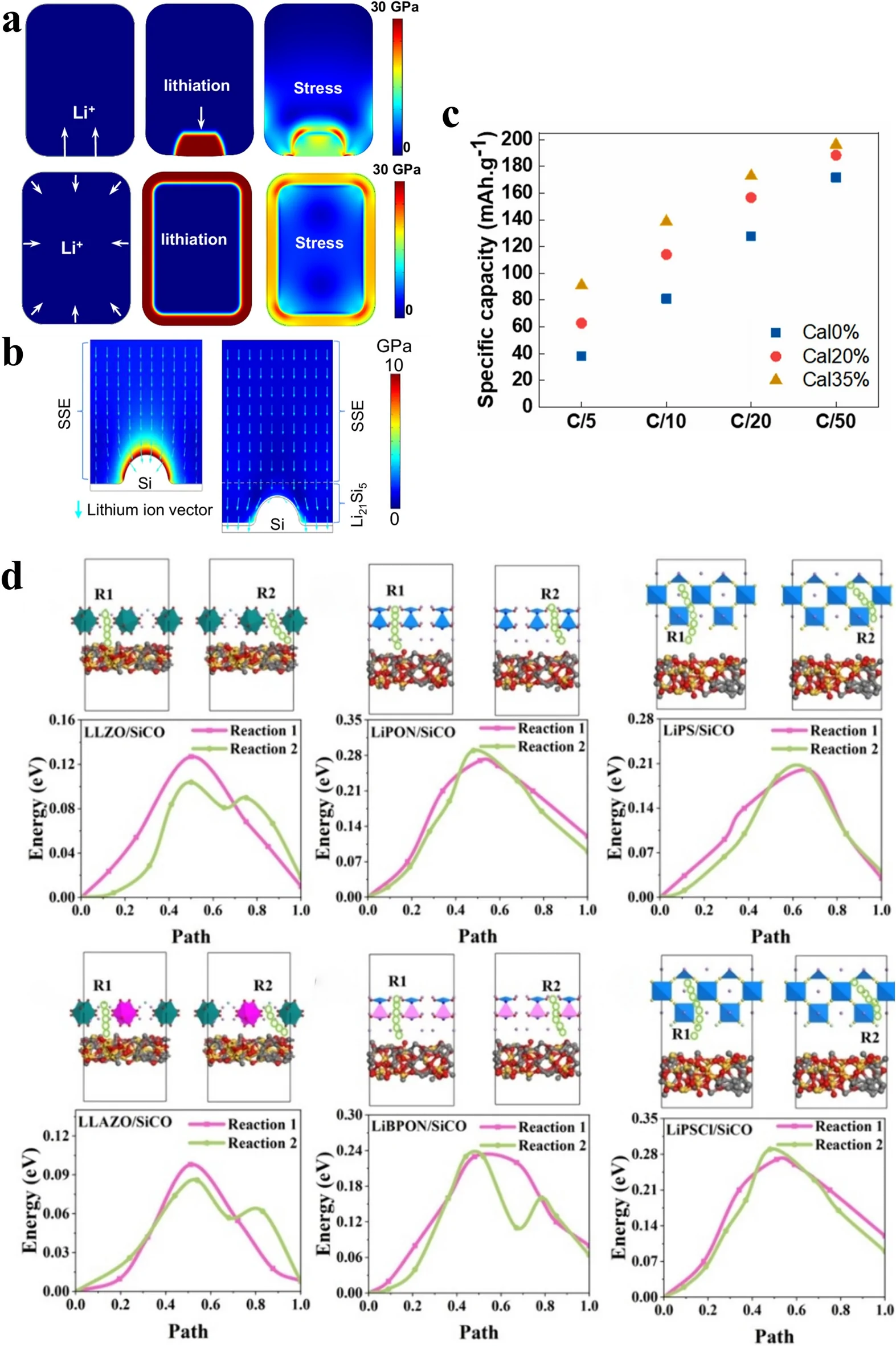
FEM simulation of **a** current and stress distribution under Si|SSE model and Si|Li_21_Si_5_|SSE model, and **b** stress distribution of pure Si particles and Si particles coated with Li–Si@Si during lithiation. [130] ©The Author(s) 2025. **c** Molecular dynamics simulation of the specific capacity and rate performance of SSBs with different electrode calendering degrees. [367] © 2024 The Authors. Published by Elsevier B.V. First-principles calculation of **d** Diffusion barriers of lithium in the interfaces to verify the simulation outcomes of machine learning method. [368] © 2024 Elsevier Inc

Finite element modeling (FEM) based on multi-physics field simulation, benefiting from the improvement of modern computer resources, has developed from a theoretical exploration stage in the last century to an important research method in the field of materials science at present. Its core principle is to discretize a continuous physical system into a finite number of interconnected elements and approximate the solution of the overall system by solving the local equations of the elements. For example, Liu et al. [366] adopted the finite element method at the continuum scale to construct a thermal-mechanical-electrochemical coupling model to analyze the elasto-plastic behavior of Si-based SSBs during charge-discharge cycles, and verified the reliability of the model through experimental data. The study found that the mechanical degradation of the silicon anode mainly stems from the accumulation of irreversible plastic strain during cycling. Appropriate application of external pressure can effectively inhibit deformation and extend battery life, but excessive pressure will increase the overall stress and raise battery safety risks. Another perfect case that can illustrate the application of FEM in the alloy-based SSBs field is the work of Zhang et al. [130], where they established two models respectively for complementary verification with the experimental results. The first model simulated the current and stress distribution at the interface between different anodes and electrolytes, and the results are shown in Fig. 29a. It can be observed that in the Si|SSE model, the interfacial stress increases significantly during the lithiation process, and the current distribution is concentrated, leading to excessive local current density and inducing the growth of lithium dendrites. In contrast, the Si|Li_21_Si_5_|SSE model benefits from the high ionic/electronic conductivity of the Li_21_Si_5_ layer, resulting in uniform current distribution and the almost complete disappearance of stress concentration, which fundamentally reduces the mechanical damage at the electrolyte interface. And the second model, on the other hand, establishes an axisymmetric model to compare the stress distribution differences between pure Si particles and Si particles coated with Li–Si alloy (Li–Si@Si) during lithiation (Fig. 29b). For pure Si particles, during lithiation, the stress at the interface between Li_x_Si and Si accumulated up to 30 GPa, directly causing the particles to fracture along the lithiation direction. This simulation result was consistent with the fracture of Si particles after 180 s observed via in situ TEM. During the lithiation of Li–Si@Si particles, lithium ions diffused rapidly and uniformly to the Si core through the Li-Si alloy layer, so the expansion stress was evenly distributed inside the particles, with a maximum stress of only 20 GPa. Moreover, the particles did not fracture but only undergo uniform volume expansion, which was consistent with the phenomenon of stable expansion of Li–Si@Si particles after 70 s observed. This multi-physics field coupling model provides quantitative guidance for the design of silicon-based all-solid-state batteries, which can alleviate mechanical degradation by optimizing the yield strength or structural design of electrode materials, thereby improving cycle performance.

Molecular dynamics (MD), based on the principles of classical mechanics, describes the interactions between particles by constructing force fields, which can simulate the structural evolution during the preparation and operation of battery materials, providing a theoretical basis for optimizing the electrode manufacturing process of alloy-based all-solid-state batteries. Alabdali et al. [367] simulated the wet manufacturing process of NMC622/Li_6_PS_5_Cl-based composite electrodes through Coarse-Grained Molecular Dynamics (CGMD), focusing on the interaction mechanisms between active materials, solid electrolytes, and carbon-binder domains. For different calendering degrees (0%, 20%, 35%), the corresponding electrode microstructures were generated through simulation, and the variation laws of volume fraction, thickness, and porosity of each component were analyzed. The results which are presented in Fig. 29c shows that a high calendering degree can significantly improve ion and electron transport efficiency, enhancing the specific capacity and rate performance of SSBs, while the uncalendered electrodes exhibit performance degradation due to uneven microstructure, high porosity, and poor interfacial contact.

In addition to the improvement of computing power, the rise of artificial intelligence technology has opened up a new path for material design and performance prediction of alloy-based anode solid-state batteries. The traditional trial-and-error method is difficult to achieve efficient screening in the huge parameter space of electrode-solid electrolyte interfaces, which seriously limits the development process of high-performance electrode and electrolyte materials. Machine learning (ML) methods can extract characteristic laws from known materials and their performance data, construct mathematical models, and after training and optimization, perform performance prediction and efficient screening of unstudied materials. Yang et al. [368] proposed a new computational strategy combining machine learning and first-principles, which realized high-throughput screening of oxide and sulfide electrolytes suitable for high-stability SiCO-based all-solid-state batteries. The simulation outcomes of CI-NEB method illustrated in Fig. 29d have confirmed that element doping (such as Al, B, Cl, and Si) can effectively improve the interface performance between electrolytes and SiCO. This research provides a new idea for the rapid screening of new functional interface materials and promotes the transformation of all-solid-state battery material design from the traditional trial-and-error method to an efficient computation-driven mode.

**Fig. 30:**
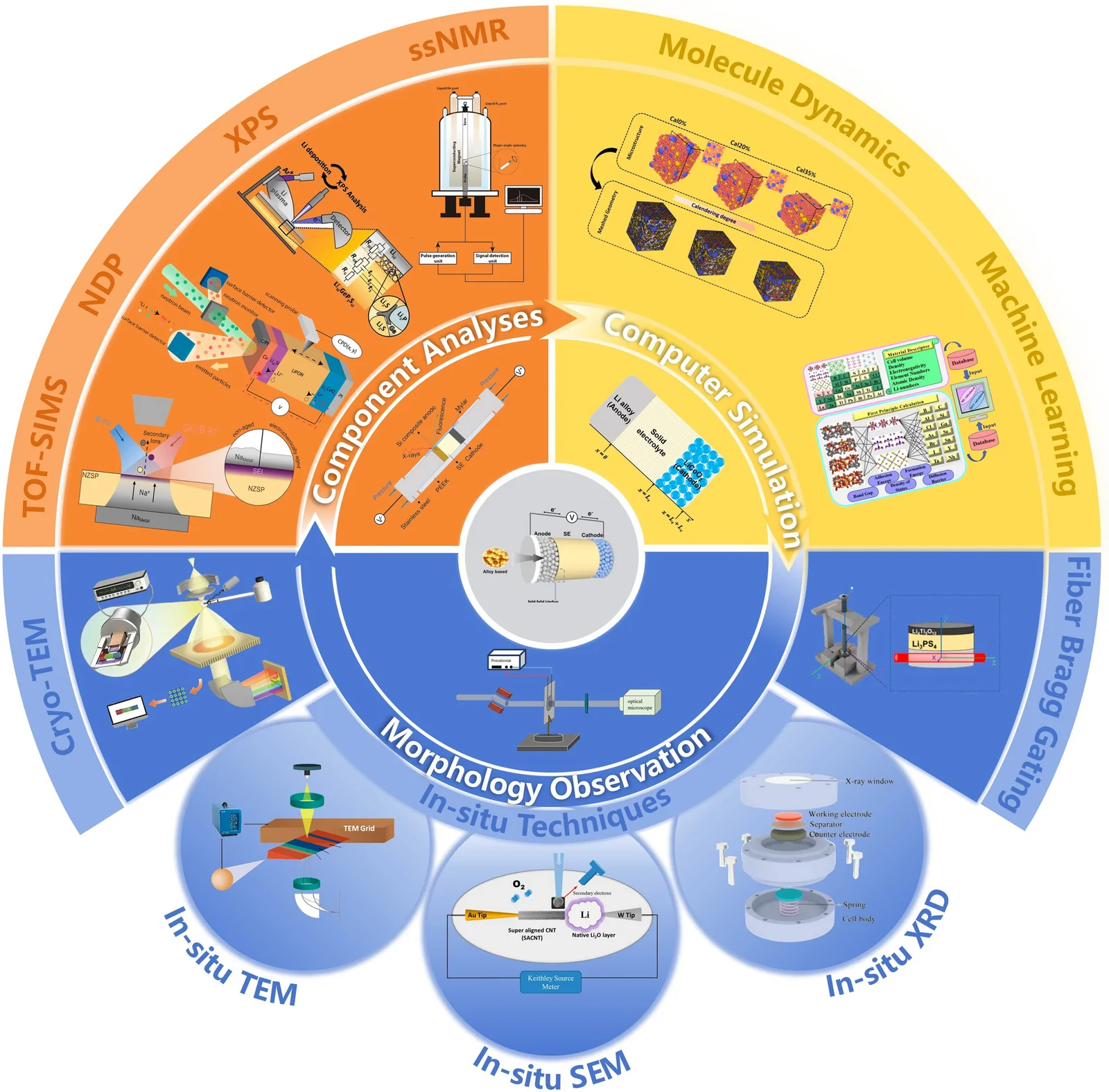
Schematic diagrams of advanced characterization methods for alloy-based SSBs. [285, 359, 362, 367–378] © 2023 Wiley–VCH GmbH. © 2022 Springer Nature Limited. ©2021 American Chemical Society. © 2024 The Authors. Published by Elsevier B.V. © 2024 Elsevier Inc. All rights are reserved. © The Korean Institute of Metals and Materials 2021. © 2017 WILEY–VCH Verlag GmbH & Co. KGaA, Weinheim. © 2017 by Annual Reviews. © 2025 Elsevier Ltd. © 2025 The Author(s). Published by Elsevier Inc. © 2016 American Chemical Society. © 2021 The Author(s). This is an open access article under the CC BY-NC-ND license. © 2016 American Chemical Society. © 2019 WILEY–VCH Verlag GmbH & Co. KGaA, Weinheim. © 2014 American Chemical Society

This section has systematically reviewed a suite of advanced characterization techniques, which collectively offer an unprecedented multi-dimensional and multi-scale perspective for deeply understanding the complex chemo-mechanical behaviors within alloy-based SSBs (Fig. 30). The core value of these techniques does not lie in the capability of any single instrument, but in the profound insights brought forth by their synergistic application. By integrating in situ dynamic morphology observations with operando high-resolution component analyses, it is now possible to effectively correlate the macroscopic structural evolution of the electrode during cycling, the chemical side reactions at the interface, and the internal stress distribution, thereby constructing a complete failure picture that spans from the atomic to the device scale.

Meanwhile, digital simulation technologies, represented by FEM and MD, are transitioning from being mere tools for experimental validation to crucial platforms for prediction and rational design. This research pattern, which tightly couples experimentation with simulation, not only reveals intrinsic mechanisms inaccessible to conventional methods such as the coupling between stress concentration and ion transport pathways, but also provides critical guidance for the rational design of high-performance electrode architectures and stable solid-solid interfaces. Despite these significant advancements, there still remain challenges in characterization. Key frontiers that require breakthroughs include deeply integrating vast, multimodal characterization data with machine learning algorithms to enable rapid performance prediction and the inverse design of materials. Looking forward, the development of correlative techniques that integrate multiple detection modalities into a single platform will be a critical step in advancing alloy-based SSBs from laboratory-scale research toward commercialization.

## Conclusions and Prospects

The advantage of the alloy anode lies in its enhancement of energy density and improvement of inherent safety. Compared to graphite, its high-capacity characteristic directly increases the quality energy density of the battery. However, its more profound commercial value lies in providing a new approach to addressing the widespread safety concerns associated with lithium metal anodes with extremely high energy density. This enables the pursuit of higher energy density without sacrificing the safety reliability of the system, thereby reducing the design cost and weight of excessive protection at the battery pack level. In the current research process, the modification strategy for alloy anode solid-state lithium-ion battery mainly focuses on the core scientific issues such as alleviating its huge volume strain, stabilizing the solid-solid interface and improving the overall ion transport efficiency. It is effective to reserve space for the volume expansion of alloy particles and inhibit their powdering through ingenious nanostructure design, such as porous skeletons or three-dimensional conductive networks. The artificial interface protection layer is constructed to enhance the mechanical stability and electrochemical compatibility of the interface, while inhibiting harmful side reactions, such as Li_3_PO_4_ or a gradient interface layer. At the electrode phase level, a ductile three-dimensional conductive network is constructed by using conductive additives with high mechanical strength, which not only ensures efficient electronic transmission, but also provides an auxiliary channel for ion migration and enhances the structure of the electrode. The function of the binder has also shifted from inert bonding to the active role of the ion-electron hybrid conductor. Its flexibility and self-healing ability further maintain the durable contact of the multiphase interface. In addition, the innovative design at the system level improves the energy density and power output of the overall battery by optimizing the integration mode. The researchers developed a flexible composite solid electrolyte that adapted to the volume change of the alloy anode. The flexibility of the polymer was used to buffer the cyclic stress, and the inorganic filler was used to improve the overall ionic conductivity and inhibit the dendritic penetration. In addition, the gradual transition from electrode to electrolyte modulus can be realized by constructing a gradient electrolyte structure, so as to alleviate the interface stress concentration. The research in this field will increasingly rely on multi-scale collaborative design and precise regulation. Ultimately, it will drive the practical application of the new generation of solid-state batteries, which feature high energy density, long cycle life and high safety. Although significant progress has been made in improving its performance, in order to accelerate the commercialization of alloy-based anode solid-state batteries, the following aspects should be considered.

To achieve the long-term cycling stability of alloy-type anode solid-state lithium-ion batteries, the forward-looking strategies should focus on a multi-scale collaborative interface and structural engineering design. At the microscopic scale, constructing a three-dimensional porous anode structure with preset buffer space can effectively absorb volume changes and prevent the agglomeration and failure of active substances, such as nano-porous metal skeletons and carbon confinement systems. At the interface level, artificial interface layers with gradient modulus, high ionic conductivity and mechanical adaptability are designed, such as LiF-rich and organic-inorganic hybrid layers. The interface layer needs to have excellent fracture toughness to suppress crack initiation, and at the same time possess good electrochemical passivation properties to block side reactions with the electrolyte. The modification of the electrolyte itself is equally crucial. The development of composite solid electrolytes with intrinsic flexibility and self-healing properties can achieve dynamic conformal contact with the volume changes of the anode, maintaining a continuous low-impedance ion channel. It is also necessary to conduct in-depth research on its interface compatibility and repair kinetics with different types of alloy anodes, ultimately achieving the ability to respond in real time to changes in electrode volume and continuously maintain low-impedance ion channels.

To achieve the theoretical high energy density, it is necessary to fundamentally solve the problems caused by alloying reactions, such as low utilization rate of active substances, excessive interface impedance and high proportion of inactive components. Through multi-level material and structural innovation, the energy density contribution at the electrode and electrolyte levels is collaboratively optimized. At the anode level, it is necessary to construct a densified electrode structure with a high proportion of active substances, low porosity, and the ability to effectively adapt to volume expansion. For instance, by developing porous micron-scale structures or gradient modulus designs, the lithium storage sites can be maximized within a limited space, thereby enhancing the initial coulombic efficiency and cycling stability. The optimization of the electrolyte layer is of vital importance. It is recommended to focus on developing ultra-thin, high-ionic conductivity and mechanically tunable solid electrolyte membranes, by reducing the thickness of inactive media to lower the internal resistance of the system and increase the volumetric energy density. Interface engineering needs to achieve precise control at the atomic/nanometer scale, and construct ultra-thin interface layers with both high ionic conductivity and electronic insulation to minimize energy loss caused by interface polarization.

Future material innovation needs to go beyond the traditional silicon and tin-based systems and explore new alloy compounds with higher theoretical capacity, lower volumetric strain and better interfacial compatibility, such as designing multi-metal intermetallic compounds (such as M_x_Si_y_Sn_z_, Li-Mg-Si systems) or metal nitrides with topological structure regulation. The lithiation reaction pathway and volume change amplitude were regulated through element doping and phase engineering. For solid electrolytes, there is an urgent need to develop new material systems that possess high ionic conductivity, wide electrochemical windows and intrinsic flexibility. For instance, superionic conductor halides with disordered structures or porous ceramic electrolytes with nanocrystalline boundary engineering to simultaneously achieve rapid ion transport and stress dissipation. The construction of new material systems will increasingly rely on the collaboration of cross-scale computing and high-throughput experiments.

In the development of solid-state lithium-ion batteries with alloy anodes, it is necessary to construct the matching system of high-performance anode and cathode and electrolyte. For example, by developing a functional solid electrolyte with high ionic conductivity and interface compatibility, and coupling it with pre-lithiated high-capacity alloy anode and high-voltage anode, the output voltage and capacity can be synchronously improved. In addition, it is necessary to innovate the integrated process of electrode and electrolyte, and develop advanced technologies such as dry electrode molding, multi-layer co-sintering, and in situ solidification of electrolyte, so as to prepare a compact and close interface integrated battery stack, reduce the proportion of inactive volume, and improve the volumetric energy density and mechanical stability. In order to achieve the optimal effect of lithium supplement, it is necessary to develop an accurate, controllable and solid-state system compatible pre-lithium method. The pre-lithium reagent with high stability and controllable lithium removal capacity was developed, and its decomposition kinetics was regulated by micro-nano structure design, so that it could release a lithium source on demand at the initial stage of battery activation. A new pre-lithiation reagent with interface compatibility was designed to make the residual phase formed after decomposition have ionic conductivity and not increase the interface impedance.

Using advanced in situ/working condition characterization technology and multi-physical field simulation is the key to reveal its complex electrochemical mechanical coupling behavior and guide the rational design of materials and interfaces. At present, it is urgent to use these technologies to deeply analyze the core scientific issues such as the volume strain dynamics of alloy anode, the evolution of SEI, the bottleneck of lithium-ion transport, and the stress distribution and relaxation mechanism. It is suggested to vigorously develop in situ technology with high spatial and temporal resolution suitable for the solid-state battery system. For example, synchrotron radiation X-ray tomography and diffraction (XRD-CT) have been used to visualize the lithiation/delithiation process of alloy particles and the interface contact failure with electrolyte. In situ scanning electrochemical microscope (SECM) and atomic force microscope (EC-AFM) have been used to analyze the ion current distribution and mechanical property evolution at the interface. In situ NMR and XPS were also used to track the dynamic changes in interface chemical composition and spatial gradient. With the advancement in new techniques, new techniques as well as combination or coupling of existing techniques can also be used to model innovative experiments for in situ analysis of battery structures, electrochemistry and interface reactions. The integration of characterization and simulation technology will expand to a higher dimension. Combining artificial intelligence and machine learning to study alloy-based anode solid-state lithium-ion batteries holds great potential. By integrating multimodal in situ data through machine learning, a prediction model from microstructure to macro performance is established. Machine learning can, by learning from a vast database of known material structures and properties, reverse-engineer new solid-state electrolytes or interface buffer layers that possess high ionic conductivity, electrochemical stability, and adaptive flexibility. More importantly, AI models can predict the formation of spatial charge layers at the interface between unknown solid electrolytes and active materials, as well as the Gibbs free energy of interface reactions, thereby pre-selecting the most promising interface combinations before synthesis. Additionally, reinforcement learning algorithms are applied to optimize electrode manufacturing process parameters, such as slurry ratio, compaction density, or heat treatment conditions.

In the process of developing and industrializing alloy-type anode solid lithium-ion batteries, mere breakthroughs in material properties are no longer sufficient to drive them toward practical applications. It requires researchers to deeply reflect and restructure the entire technological path from material synthesis, electrode processing to battery integration [379]. To achieve large-scale application of alloy-type anode solid-state lithium-ion batteries, it is significant to fundamentally solve the problems of large-scale amplification existing in their material systems, manufacturing processes and system integration [380]. In terms of materials, it is necessary to develop low-cost and scalable synthetic pathways for the preparation of high-performance alloy anodes and solid electrolytes. For instance, silicon-based composite anode precursors can be produced on a large scale through wet mechanical alloying, or ultrathin sulfide-polymer composite electrolyte films can be prepared by solution casting to replace the high-energy-consuming vapor deposition and high-temperature sintering processes. This has driven materials scientists not only to synthesize particles with excellent lithium storage properties, but also to shift their focus to designing powders suitable for specific advanced processes. In terms of manufacturing processes, it is necessary to innovate the electrode-electrolyte integration technology, develop technologies compatible with large-scale roll-to-roll production, avoid interface contamination and improve packaging efficiency. It is also valuable to develop high-precision pre-lithiation equipment and processes suitable for solid-state batteries to achieve quantitative and uniform supplementation of active lithium. Ultimately, through the coordinated upgrade of materials, processes and systems, the large-scale application of alloy-type anode solid-state batteries under the requirements of high energy density, high safety and low cost can be achieved, promoting the development of major fields such as electric vehicles and large-scale energy storage.

## Data Availability

Data will be made available on request.
